# Outlook for Implementation of Genomics-Based Selection in Public Cotton Breeding Programs

**DOI:** 10.3390/plants11111446

**Published:** 2022-05-29

**Authors:** Grant T. Billings, Michael A. Jones, Sachin Rustgi, William C. Bridges, James B. Holland, Amanda M. Hulse-Kemp, B. Todd Campbell

**Affiliations:** 1 Bioinformatics Graduate Program, North Carolina State University, Raleigh, NC 27695, USA; gtbillin@ncsu.edu (G.T.B.); jim.holland@usda.gov (J.B.H.); 2 Department of Crop and Soil Sciences, North Carolina State University, Raleigh, NC 27695, USA; 3 Pee Dee Research and Education Center, Clemson University, Florence, SC 29506, USA; majones@clemson.edu (M.A.J.); srustgi@clemson.edu (S.R.); 4 Department of Mathematical and Statistical Sciences, Clemson University, Clemson, SC 29634, USA; wbrdgs@g.clemson.edu; 5 Plant Sciences Research Unit, The Agricultural Research Service of U.S. Department of Agriculture, Raleigh, NC 27695, USA; 6 Genomics and Bioinformatics Research Unit, The Agricultural Research Service of U.S. Department of Agriculture, Raleigh, NC 27965, USA; 7 Coastal Plains Soil, Water, and Plant Research Center, The Agricultural Research Service of U.S. Department of Agriculture, Florence, SC 29501, USA

**Keywords:** cotton breeding, GWAS, genomic prediction, fiber quality, cotton yield

## Abstract

Researchers have used quantitative genetics to map cotton fiber quality and agronomic performance loci, but many alleles may be population or environment-specific, limiting their usefulness in a pedigree selection, inbreeding-based system. Here, we utilized genotypic and phenotypic data on a panel of 80 important historical Upland cotton (*Gossypium hirsutum* L.) lines to investigate the potential for genomics-based selection within a cotton breeding program’s relatively closed gene pool. We performed a genome-wide association study (GWAS) to identify alleles correlated to 20 fiber quality, seed composition, and yield traits and looked for a consistent detection of GWAS hits across 14 individual field trials. We also explored the potential for genomic prediction to capture genotypic variation for these quantitative traits and tested the incorporation of GWAS hits into the prediction model. Overall, we found that genomic selection programs for fiber quality can begin immediately, and the prediction ability for most other traits is lower but commensurate with heritability. Stably detected GWAS hits can improve prediction accuracy, although a significance threshold must be carefully chosen to include a marker as a fixed effect. We place these results in the context of modern public cotton line-breeding and highlight the need for a community-based approach to amass the data and expertise necessary to launch US public-sector cotton breeders into the genomics-based selection era.

## 1. Introduction

Plant breeders play a crucial role in the development of new cultivars by selecting characteristics of contrasting traits in order to ultimately obtain rare individuals that are recombinant for otherwise opposing phenotypes. For example, in cotton (*Gossypium* spp.) breeding there is a strong negative association between total fiber yield and major fiber quality traits, especially fiber length and strength [1]. This negative association is usually attributed to pleiotropy, linkage, or both [2]. To overcome these negative associations, breeders screen large population sizes for the “needle in the haystack”—a plant or line (depending on the stage in the breeding process) that has sufficient fiber quality characteristics with an overall acceptable yield [3].

Two allotetraploid (2n = 4x = 52) species comprise the vast majority of the cotton fiber and cottonseed production worldwide. The two predominant species are *Gossypium hirsutum* (Upland cotton, 97% of acreage) and *G. barbadense* (Pima, Sea Island, Egyptian, or Extra-Long Staple cotton—3% of acreage) [4]. Although the two species can hybridize relatively easily, much of the Upland cotton breeding is confined to the domesticated, elite gene pool due to poor plant performance following hybridization and inbreeding (“hybrid breakdown,” see Dai et al. [5]). The key difference between the two species is that *G. barbadense* has a higher quality (longer, stronger, finer) fiber, whereas *G. hirsutum* has a shorter growing season, average fiber quality, and substantially elevated yield [6].

Currently, public-sector cotton breeders in the US screen phenotypic characteristics on tens of thousands of early generation progeny (F3 and F4) for fiber quality, yield, and yield components, as well as smaller numbers (10’s or ~100’s of entries) of advanced progeny (F5 and forward). This annual screening process is expensive, laborious, and requires the collection of a large volume of hand-picked and/or machine-harvested seed cotton samples. Following harvest, this large volume of samples must be individually ginned on a laboratory gin to separate seeds and fibers to obtain information on the gin turnout (ratio of fiber to combined weight of seed and fiber) and physical fiber properties (e.g., length, strength, fineness, etc.), measured using standardized equipment such as the High Volume Instrument (HVI) and/or the Advanced Fiber Information System (AFIS). Little is known about the trait architecture of many complex traits in cotton due to the low co-occurrence of identified significant loci shared between studies [7]. Since most cotton breeding programs operate in a largely closed, inbreeding-based manner, further knowledge is needed at the breeding-program scale to dissect complex traits and devise genomic approaches to make cotton breeding easier, faster, and more efficient.

The Pee Dee (PD) Germplasm Enhancement Program has been a long-term cotton improvement project with USDA-ARS in Florence, SC, USA, since 1935. Over time, breeders have maintained genetic diversity while combining fiber quality traits, especially fiber strength and length, with insect/disease resistance and acceptable levels of fiber yield and yield components [8]. It has been noted that a majority of the commercial cultivars in the US have a PD line somewhere in the pedigree [9], with similar trends observed in commercial cultivars developed in China [9], usually attributed to PD lines’ exceptional fiber strength. Due to the long-term emphasis on enhancing diversity, Campbell et al. (2013) [9] also found that the PD breeding program will continue to be a useful source of genetic diversity in the future, although how to efficiently utilize this diversity in a breeding program is an open question.

Selection for fiber quality and yield is complicated by the contribution of genotype × environment (G × E) interactions on these traits [1,10]. Generally speaking, breeders select for trait stability across their candidate environments, although yearly fluctuations in weather, precipitation, disease pressure, and random field variation can confound a breeder’s ability to make direct selections on observed phenotype means and relative ranks [11,12]. Understanding how the genetic underpinnings of key traits in cotton vary with respect to the loci of variable effect sizes (small to high) or quantitative trait loci (QTL), and the cumulative effect of many loci (the polygenic effect) in multiple years and locations is crucial for designing more efficient selection schemes [13]. The stability of these effects across time is also essential to utilizing genomics-based prediction and downstream selection programs, where phenotypes are predicted using genotypic information [14].

Research assessing genomic selection across multiple environments in cotton is limited. A group in Australia studied the utility of genomic selection for cotton fiber length and strength on historical data and found a similarly favorable performance using a range of statistical models with a wide variance in prediction accuracy in different environments [15]. Little research has been conducted to explore the potential for genomic selection within the public-sector US Upland cotton gene pool, except a single study in a structured population looking at six fiber quality traits with trials in one location [16]. There is substantial opportunity to build on this work, especially with regard to seed composition traits and yield.

The goal of this study was to evaluate the genetic underpinnings of critical quantitative traits, including seed composition, yield, and fiber quality measured with both HVI and AFIS, across multiple environments in a set of historical cotton breeding materials (the Pee Dee breeding program), with the longer-term goal of conducting a preliminary analysis for implementing a genomics-based selection in public cotton breeding programs. We aimed to explore loci with estimable effects using association mapping and the polygenic background based on realized genomic relationships.

## 2. Results and Discussion

### 2.1. Genomic Relationships between Lines

**Figure 1:**
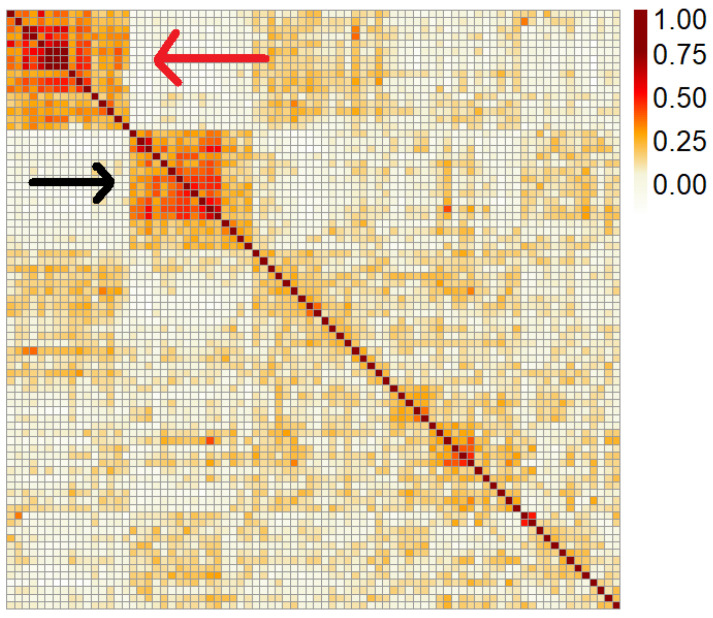
Genomic relationship matrix among the 80 lines in this study. The deeper red colors indicate a higher pairwise relationship between lines at the whole-genome level. The two large blocks in the top left consist of (1, red arrow) half- and full-sib relatives with PD695 or PD875 parents; and (2, black arrow) lines selected out of the initial gene pool from the Pee Dee breeding program in the first two breeding cycles. Breeding cycles are outlined in Campbell et al. (2011) [8].

A total of 15,177 SNPs remained after filtering to retain only polymorphic SNPs from the CottonSNP63K array. Based on historical pedigree records, this set of 80 lines is related mostly within four or so cycles of breeding, with dozens of rounds of successive recombination resulting in admixture between groups [17]. Based on the investigation of relationships from the SNP data, it does appear that recombination has broken relationships, with the most significant exceptions occurring within two big genotype blocks, where most individuals in each of the blocks of the heatmap are (approximately) related at the full- or half-sib level (Figure 1). Other smaller blocks (2–5 lines) along the diagonal correspond to parent–child relationships. Previous analysis [17], together with the GRM here, show that this panel is fairly weakly structured overall, indicating the suitability of this dataset for a genome-wide association study, as long as the existing population structure is accounted for when estimating allele effects and *p*-values.

### 2.2. Trait Heritabilities

In line with prior estimates [18], the fiber quality traits generally have higher broad-sense heritabilities than yield and its components (Table 1). For seed traits, seed oil % was more heritable (comparable to most fiber quality) than seed protein % (closer to most yield components), supporting a previous genetic analysis on the Pee Dee breeding program germplasm [19]. Immature fiber content has markedly lower heritability than all other fiber quality traits examined here (H^2^ = 0.22, compared with H^2^ ≥ 0.75 for all others). Gin turnout (the ratio of lint to seed and lint) and seed index (seed weight) were the more heritable yield components, whereas bolls per square meter and boll weight were the least heritable yield components. Lint yield, the multiplication product of seed cotton yield and gin turnout, was highly heritable (H^2^ = 0.96).

The average single year–location environment broad-sense heritabilities were consistently lower than the overall heritabilities, often having high standard errors (Table 1, Supplemental Table S1). This suggests that, in general, averaging over more observations provides more benefit than the penalty accumulated from the added genotype × environment variance. The overall heritabilities are essentially the ratio of genotype variance divided by the sum of genotype variance and proportions of genotype × environment and residual variance components that contribute to standard errors of genotype mean comparisons. Lower heritability in certain traits could be associated with (a) a lower genetic contribution to the trait; (b) differential genotype response by environment (G × E); or (c) increased residual variance due to measurement or experimental error (difficulty in measuring the phenotype accurately). Lower heritability translates to a decreased ability to detect genetic loci affecting the trait, as well as complicates the implementation of phenotypic or genotypic selection due to high standard errors for the genotype effects.

### 2.3. Genome-Wide Association Study

We performed an association analysis between each of the 14 replicated field trials (by environment) and the filtered genome-wide SNP data on the panel of 80 historical cotton lines from the Pee Dee breeding program (results shown in Supplemental Table S2). The genomic inflation factor and QQ-plots showed PCA adequately reflected the population structure (Supplemental Data S1). The genome-wide association study revealed hundreds of trait-associated SNPs. Significant SNP-trait associations were identified for all 20 traits examined (Table 2, Supplemental Table S2). A combination of four individual field trials (Hartsville 2004, Tifton 2006, Blackville 2004, and Rocky Mount 2006) accounted for 45% of the SNP-trait associations discovered.

**Figure 2:**
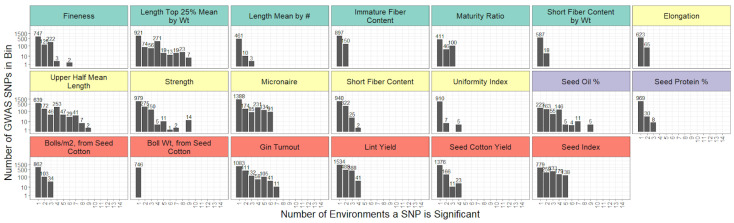
The number of year–location environments each SNP-trait association was discovered in. Results are shown for markers identified with at least 5 of 100 resamples of the association test with FDR-corrected *p*-value < 20%. The *y*-axis is plotted with a log 10 transformation. AFIS traits are highlighted in teal, HVI traits in yellow, seed traits in purple, and yield traits in red.

Two key findings arose from the GWAS of the 14 field trials. First, some traits (AFIS length mean by number, HVI elongation and uniformity index, seed protein %, boll weight and bolls per square meter, and lint yield) yielded little to no marker–trait associations in 5+ environments, indicated as “-” in Table 3. Second, when marker–trait associations were detected, the same marker–trait associations were usually detected in two or fewer environments (Figure 2, Supplemental Figure S1), with the exception of a handful of marker–trait associations for fiber strength, length, oil, gin turnout, and seed index Supplemental Table S3. 

Traits with below-median heritability (H^2^ < 0.85) were slightly less likely to have at least one significant GWAS hit in at least three environments (Fisher’s exact test one-sided *p*-value = 0.070; also see Supplementary Figure S2 for a visualization). An increase in the number of lines present in a field trial would make it easier to detect loci that contribute to these difficult to dissect traits in a wider range of environments by increasing statistical power. Considering previous findings, that genotype × environment interactions exhibit substantial control over many of these traits [13,20], variable effects of the same allele in different environments could have contributed to the inability to detect many stable marker–trait associations for most traits.

The results for cotton fiber quality (length/strength) are more promising, with hundreds of marker–trait associations discovered in multiple environments. The markers discovered here (especially markers detected in 3+ field trials, Supplemental Table S3) will be immediately useful to breeders, particularly while high-throughput tools for genome-wide marker profiling in cotton are not feasible on a program-wide scale (due to cost or other factors). A potential application of these markers is using Pee Dee lines as the non-recurrent parent in a high yielding elite × high fiber quality backcross scheme for marker-assisted selection. Screening the fiber quality is notoriously difficult due to the high time and monetary cost involved with sampling fibers from individual plants, the preparation of samples, and testing with the HVI or AFIS. There was ~50% overlap in markers between the fiber length measurements from AFIS and HVI, although many of them were still only specific to fiber length parameters from one of these systems. Others have noted the biological and practical differences between AFIS and HVI and the difficulty in choosing a single parameter (or a small number of parameters) to predict which fibers will ultimately have the best spinning quality [21]. Breeders select on fiber quality through indirect measures of spinning quality, such as fiber bundle strength/length (HVI), or individual fiber measurements (AFIS), so a careful evaluation of a breeder’s specific goals is needed before applying any of these markers into a marker-assisted selection program.

### 2.4. Genomic Prediction Evaluation through Cross Validation

Although marker-assisted selection, based on markers originating from GWAS or QTL mapping, has demonstrated success in cotton breeding for nematode resistance [22], it is generally accepted in quantitative genetics and plant breeding that a genomic selection scheme is an efficient alternative for complex, highly polygenic traits [23] such as yield and fiber quality. To be most effective, a genomic selection scheme should be built with a model that can be used to predict performance across environments. For example, prediction model training data would be collected from one or more year–location environments and used to predict performance in one or more different year–location environments. In some cases, a breeder has data collected from one or more year–location environments and would be interested in using all of that collected data for prediction model training to predict the performance of breeding lines not phenotyped in the same year–location environments in an effort to decrease phenotyping costs. In this study, we compared the ability to predict the performance between environments and as a baseline compared it to the ability to predict performance within the same year–location environment(s).

Various genetic models exist to connect genotyping information with phenotypic observations [24]. One such model is the infinitesimal model, which states that variation in complex traits is due to the cumulative effect of many small effect loci [25]. In conjunction with the typical desire of a line development breeder, an additional restriction can be enacted to exclusively model additive genetic effects. To assess the application of the infinitesimal model in an additive framework, we used the filtered set of 15,177 SNPs to estimate realized genomic relationships between all pairs of 80 lines, using the correlation matrix method. We then fit a GBLUP model using a linear kernel with RKHS [26] to test the agreement between genomic relationships and the observed phenotype means for different within- and between-environment scenarios. Correlations between the predicted phenotype value and observed phenotype mean for the testing set were used to estimate the prediction ability. Lastly, we evaluated the potential for adding stable GWAS hits as fixed effects, to see if incorporating additional information from loci with estimable effects could improve the prediction ability.

#### 2.4.1. Cross-Validation within Overall and Environment Means

**Figure 3:**
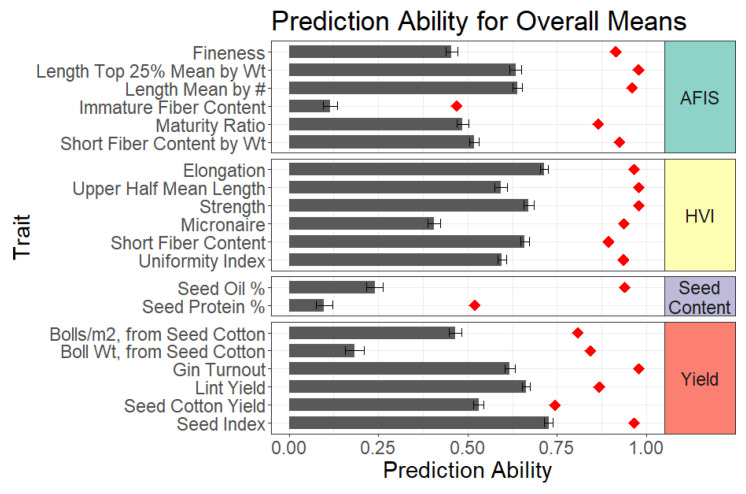
Prediction ability for the genomic best linear unbiased predictor (GBLUP) models trained and tested on the overall means. Error bars are standard errors. The red diamond corresponds to the theoretical maximum prediction ability, estimated as the square root of the trait heritability. AFIS: Advanced Fiber Information System; HVI: High Volume Instrument. AFIS traits are highlighted in teal, HVI traits in yellow, seed traits in purple, and yield traits in red.

For the models trained and tested on the overall means, prediction abilities for fifteen of the twenty traits were in the moderate to high range (*r* ≥ 0.5), with the exception of AFIS immature fiber content, HVI micronaire, seed protein/oil, and boll weight from seed cotton (Figure 3, Supplemental Figure S3, grey bars). The differences in prediction ability among traits could partially be attributed to differences in heritability, which makes sense since the square root of the heritability is the theoretical maximum genomic prediction ability (Figure 3, Supplemental Figure S3, red diamond).

**Figure 4:**
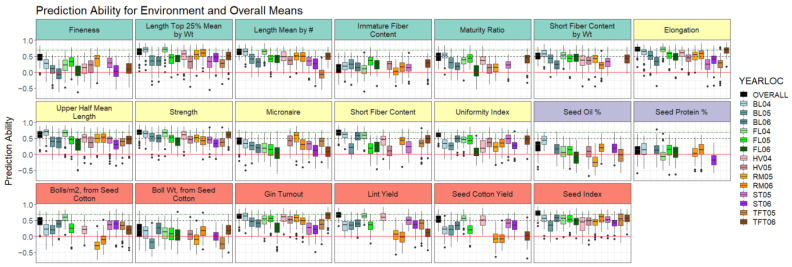
Prediction ability within 14 individual year–location environment means and overall means for 20 fiber, agronomic, and seed traits. The red solid line corresponds to *r* = 0, the black dashed line is *r* = 0.5, and the green dashed/dotted line is *r* = 0.7. AFIS traits are highlighted in teal, HVI traits in yellow, seed traits in purple, and yield traits in red. Wt = weight.

To compare the consistency of prediction ability estimates across the 100 cross-validation runs, the standard deviation for the 100-point estimates of the prediction ability for each trait were calculated (Supplemental Table S4). The traits with a low prediction ability (*r* < 0.4), immature fiber content, seed oil, seed protein, and boll weight prediction abilities also had the highest standard deviations, indicating large variability in the prediction ability, depending on how the data were randomly partitioned during cross-validation (Figure 4, Supplemental Figure S4, black box and whiskers). The HVI micronaire prediction ability had a comparable standard deviation to other more easily predicted traits, indicating micronaire would likely be difficult to predict regardless of the training population composition. Together, these results indicate the importance in the careful selection of a training population to drive improved predictions in practice for most traits with a low prediction ability. In comparison, among the traits studied here, HVI elongation and lint yield prediction abilities had the two lowest standard deviations, suggesting that the exact composition of the training population relative to the target population appears less important in this panel. Experiments with larger numbers of individuals are needed to further parse out these differences in training population design and optimization in reference to genomic selection in cotton breeding.

Disaggregated environment-specific means were extracted for each line to determine if the prediction ability differed based on whether the overall or the environment means were used to train and evaluate the model. We hypothesized that the prediction ability would differ for a trait depending on whether the trait means were modeled from all of the data compared to a single year’s worth of data due to either an additional replication across environments (i.e., more precise estimates of line means in the overall means) or differences in the phenotype distribution (i.e., some outliers in certain environments that are always difficult to fit accurately in an additive genetic model).

Some traits varied substantially depending on the year–location of the trait means analyzed in the environment dataset. For example, lint yield prediction ability ranged from close to zero for both the Rocky Mount 2005 and 2006 means all the way up to 0.64 in Florence 2006, and topping out at 0.66 for the overall means. Especially for yield, these results indicate the importance of replicated, multi-location, multi-year trials to build models for employing genomic selection in highly complex traits, such as yield in cotton. For other traits, model implementation based on one year (or a small number of year–locations) of data seems more plausible. The prediction ability for fiber strength and length was more uniform across different subsets of the data, with prediction ability >0.29 in all of the single environment models (single environment mean prediction ability of 0.44 for fiber strength, 0.34 for fiber length).

The two seed composition traits, however, proved to be more challenging in genomic prediction. Seed protein, which had the second lowest heritability of the traits evaluated here (H^2^ = 0.27), had a uniformly poor prediction ability (0.10 for overall means, 0.03 for single environment). The seed protein % had a strong environmental effect and a small genetic component [19], meaning that inherent variability in field trials can make it very difficult to predict a line’s performance on the basis on genomic relationships. Seed oil, on the other hand, had high heritability (H^2^ = 0.88), but a long tail in the phenotype distribution, with some lines having very high and very low seed oil content (Supplemental Figure S5). For non-continuously distributed traits, an additive genetic model (such as GBLUP) is unable to capture effects associated with extreme phenotypes when those extreme values are not genetically distinct from the other lines in the model. To predict seed oil and protein, further careful selection of germplasm specifically for that purpose and experimental design would be necessary to drive an improved prediction ability.

Our findings show that even with a modest number of genetic markers and small training population size, encouraging levels of prediction ability can be achieved for many traits within a breeding program. Almost uniformly, the best cross-validation prediction ability was achieved when using the least squares’ means for each line from the overall dataset compared to single year–locations in the environment dataset, likely due to the ability to more accurately capture a line’s breeding value with more replication (Figure 4, Supplemental Figure S4). Further research is needed to determine if this trend holds up with a larger, more diverse panel in an applied cotton breeding program, aiding breeders in deciding how to allocate limited field resources and time.

#### 2.4.2. Cross-Validation between Environments

We also examined the possibility of training a model in one environment and then predicting the unobserved lines’ performance in other environments, i.e., between environments. This analysis solely utilized the environment dataset, where we trained the model for each of the 14 field trials, and for each we tested the model with data from each of the 14 field trials (196 combinations for each trait, including 14 self-comparisons equivalent to Figure 4). Seed cotton yield, fiber yield, boll weight by seed cotton yield, and bolls per square meter were much more difficult to predict across environments, suffering a ~30–60% decrease in prediction ability, with larger decreases when predicting between years than between locations (Table 3, Supplemental Table S5; data from the same year was easier to predict between). Since these were all dryland trials, and overall weather across the experimental locations was likely similar between years, these results could be due to the confounding effects of weather on crop productivity and the resulting genotype × year interactions. These results also highlight the need for highly replicated trials with a variety of weather conditions to estimate the genetic effects associated with yield and its substituent components, if the goal is to predict for dryland locations.

For most other traits, however, there was a small penalty associated with training the model in one environment and then trying to predict the phenotype means of unobserved individuals in a different location or year. Encouragingly, the HVI length and strength prediction models transferred to other environments with a minimal penalty (roughly ~10% decrease in the prediction ability for across location/same year predictions, no penalty for predicting across years at the same location). For fiber length and strength, in contrast to yield, it may be better to perform trials at multiple locations regardless of the year, since this is the situation that seems to be most challenging to the model examined here. These results further support the immediate application of genomic selection to these high-value fiber quality traits.

#### 2.4.3. Inclusion of GWAS Hits as Fixed Effects

The GBLUP model is designed to capture the additive genetic component for a trait by connecting overall genetic relatedness to the lines’ breeding value. The model operates under the assumption of the infinitesimal model, which states that an individual’s phenotype is due to the additive effects of many loci across the genome, each with a very small effect. GBLUP can have a poor prediction ability in the presence of violations to this assumption—for example, if dominance, epistasis, or a small number of high-effect loci exert substantial effects on the phenotypes. Since GBLUP is only capturing the additive component of genetic variance for a trait, it is important to investigate how alternative genetic models (kernels) can be used to improve the prediction ability (and eventual prediction accuracy).

**Figure 5:**
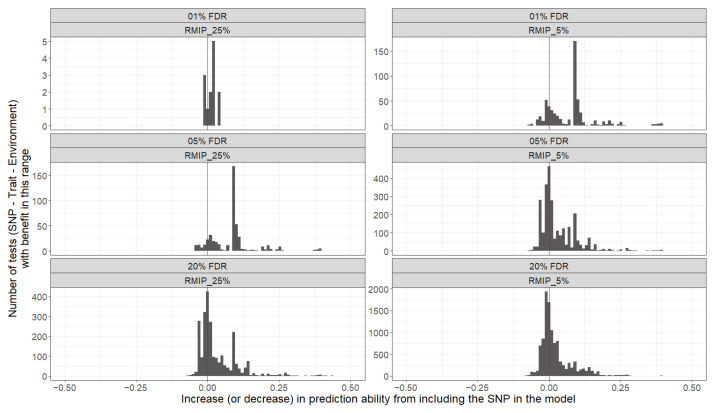
Histograms of change in prediction ability due to inclusion of genome-wide association study (GWAS) hits as fixed effects in the genomic prediction model at differing significance thresholds. FDR: Benjamini–Hochberg False Discovery Rate *p*-value correction. RMIP: resample model inclusion probability.

We investigated the possibility of integrating individual GWAS hits into the GBLUP model by comparing models that include and do not include one significant SNP. This procedure was repeated for each of the SNPs that were stable (detected in ≥3 environments). For each GWAS hit, we calculated the change in prediction ability observed from including that GWAS hit as a fixed effect in the model (only in the environments the SNP was detected in). At the threshold established and used in the preceding portion of this study (BH FDR < 20%, RMIP ≥ 5), most (%) GWAS hits improved the model prediction ability (Figure 5). With stricter significance level filtering, a higher proportion of GWAS hits improved the model prediction ability, although many GWAS hits which provided small prediction improvements were lost. This analysis highlights the need to carefully select a threshold for incorporating markers as fixed effects, as there is a fine line between overly- and underly-conservative. Future experiments with large population sizes will enable more separation as to which variants should or should not be directly incorporated into the prediction model, and/or used independently for marker-assisted selection.

### 2.5. Outlook for Genomic Prediction in Public Cotton Breeding

Genomic prediction has demonstrated success in many economically important crop species. The vast majority of cultivated varieties of cotton in the United States are bred by private companies, carrying patented transgenes for insect resistance and herbicide tolerance. Although the role of public-sector cotton breeders is constantly evolving, public breeders have always played key roles in pre-breeding for germplasm improvement, basic and applied genetic studies, and the introgression of alleles from uncultivated lines or specific genotypes into elite backgrounds. Genomic selection presents an opportunity for cotton breeders to accelerate genetic gain in the cotton gene pool by increasing the efficiency and accuracy of selection.

As a first step, we used GWAS to identify SNPs associated with the 20 traits in this study. Many loci significant in one environment were not significant in other environments, a frequent problem in GWAS that can be due to a number of practical or statistical matters, potentially including a lack of power to detect small-effect loci in this small panel, normal difficulties in obtaining accurate trait measurements in field trials, and genotype × environment interactions. The GWAS results, especially those stable associations discovered in 3+ environments, can be utilized by breeders to develop genetic screens for marker assisted selection.

In this study, we evaluated the prediction ability of a standard genomic prediction model in 20 traits that cotton breeders frequently evaluate in their breeding materials. The analysis of AFIS and HVI fiber quality traits, seed oil/protein, and yield and its substituent components revealed heterogeneity in the prediction ability between traits and scenarios. Generally, heritability was a decent predictor for prediction ability, as revealed by cross-validation, although seed oil % stood alone in having a high heritability but low prediction ability. A poor genomic prediction performance can occur when the genetic model differs from that of the underlying genetic architecture. Seed oil is an oligogenic trait with a small number of major genes in some crop species such as *Brassica* spp. and soybean [27], suggesting further research is needed to identify the underlying allelic variation controlling cotton seed oil accumulation. These results serve as a reminder that a one-size-fits-all approach is not possible for genomic selection in cotton breeding, and breeders need to carefully develop training populations and GS algorithms to accurately model the underlying genetics and phenotype distribution for their target traits.

More field trials with a larger training population and high replication are needed before the genomic prediction for yield or yield components can be feasible in practice. Public-sector cotton breeders usually limit replicated field trials to <100 entries at the F_5_ stage and forward due to challenges in management and logistics for larger sizes of field trials, as well as increased residual error due to field heterogeneity. However, with a well-designed training set, perhaps using historical data and lines from within the breeding program, genomic prediction could be implemented for fiber length and strength immediately. However, the current genotyping platforms available to cotton breeders are cost-prohibitive to use at any early stage in the breeding cycle because of the amount of plants that need to be screened (thousands to tens of thousands), so a low-density, high-throughput system for assessing genomic relationships is crucial to make genomic selection a reality for cotton breeders.

For fiber length, one group used direct measures of expression and single nucleotide variants in 400 known fiber length genes to predict fiber length (measured using upper half mean length from HVI) in a biparental population [28]. With a training set of ~100 individuals and testing set of ~350 individuals, they were able to achieve prediction accuracy >0.80 within the family examined, demonstrating that the use of many genetic variants distributed across the genome was able to capture the heritable component of fiber quality. Our results correlate well with these findings using a distributed marker set, indicating that genomic prediction within environments, even with a relatively small number of markers and small training set, seems feasible. Given the high linkage disequilibrium in cotton and long haplotype blocks often spanning many millions of bases [29], a carefully chosen set of variants should be able to roughly approximate the tested and proven gene-by-gene approach.

Others have modeled fiber quality using a marker × location interaction term to predict cotton fiber quality, finding that adding this information to the model significantly improves the prediction accuracy when trying to predict performance across environments [15]. They were able to model marker × location interactions since they had 8+ years of data from each site, so the separation of a line’s performance between environments was more clear. Directly accounting for G × E is promising for genomic predictions in cotton, although the biggest challenge is accumulating the amount of data necessary to accurately capture these high-level interactions (thousands of markers by many environments, plus the shared effect of a marker across environments). Rogers and Holland (2022) [30] found that such high-complexity models improve the prediction accuracy only when environments overlap between the training and testing set, further demonstrating our inability to capture genotype × environments based on the measurable environmental variables.

## 3. Materials and Methods

Eighty historical cotton lines from the PD breeding program were genotyped with the CottonSNP63K array [31], as reported in Billings et al. (2021) [17]. Field trial data corresponding to these eighty cotton lines was collected between 2004–2006 at six locations across the mid-south and southeast US cotton belt [32]. In brief, eighty released cotton lines were planted in an alpha-lattice, randomized incomplete block design, in two-row plots with two to four replicates depending on the field space availability. Data collected in these trials included yield, yield components, fiber quality (HVI and AFIS), and seed protein and oil (Table 4).

Statistical analysis was performed using the method of Campbell et al. (2009) [32] with PROC MIXED in SAS v9.4 (SAS Institute, Cary NC). The line means for all 20 traits were estimated for the dataset overall (genotype fixed effects across all environments) and for each environment individually (calculated from the genotype × environment interactions). Means were calculated with LSMEANS by fitting the appropriate model with genotype, location–year, and genotype × location–year interaction as fixed effects, and replicate nested in location–year and incomplete block nested in replicate as random effects. (Data were collected and analyzed on eight additional traits which for simplicity will not be discussed in the remainder of the paper, but will be provided in supplemental materials, including AFIS 2.5 percentile length by number, 5 percentile Fiber length by number, Fiber length by weight; HVI yellowness and reflectance; plant height; and boll density and boll weight calculated from lint.).

Family-mean broad-sense heritability (H^2^) and the associated standard errors were calculated for the overall and per environment datasets using a random-effects model fitted with PROC MIXED in SAS v9.4 [33]. Briefly, a random-effects model is fitted and the proportion of phenotypic variance, excluding errors associated with environments, replicates, or incomplete blocks, explained by the lines, was calculated. Traits having a very low heritability (H^2^ < 0.01), indicative of a very low genotypic effect relative to the error component in the model, were excluded from the corresponding single environment analysis.

Genome-wide association study (GWAS) was performed in PLINK v1.9 [34,35] for the overall and environment means, with SNPs having a minor allele frequency (MAF) > 0.05 using the linear command. The genomic inflation factor and QQ-plots were used to select the first five principal components to correct for population structure. Results were filtered using the 20% Benjamini–Hochberg discovery rate threshold. Due to this population’s small sample size and known family structure of this population, the resample model inclusion probability was used as suggested in Bian and Holland (2017) [36]. It allows for cross-validation and filtering of identified significant SNPs. One hundred random subsets of the data were generated, each consisting of 80% (64 of 80 lines) of the data. Filtering on each of the random subsets of SNP data was performed using the maf command in PLINK to maintain MAF > 0.05 for each marker in the subsetted data. GWAS was run on each data subset, and the number of GWAS runs detecting each marker–trait association was recorded. Using the thresholds suggested by Bian and Holland (2017), hits from GWAS were only retained if they were significant in at least 5 of 100 runs; hits significant in at least 25 of 100 runs were also noted, as well as those SNP-trait associations that exceeded the more stringent 5% and 1% false discovery rate (FDR) thresholds, and their combinations with resample model inclusion probability (RMIP) of 5% and 25%—6 total thresholds. GWAS hits were classified as stable if they were discovered in at least three environments on the basis of the 5% RMIP and 20% FDR threshold level.

The cross-validation accuracy of the genomic best linear unbiased predictor (GBLUP) was estimated using the Reproducing Kernel Hilbert Spaces (RKHS) model for the overall and environment datasets in an 80% train, 20% test arrangement over 100 iterations in BGLR statistical package [37]. The realized, additive genomic relationship matrix (GRM) was the correlation matrix with the tcrossprod(G)/ncol(G) commands in R v4.0.5, with the centered, scaled genotype matrix produced using recode A in PLINK. Only markers with MAF > 0.05 in the training set were retained for construction of the GRM. For the overall and environment means, phenotypes were centered to mean 0 and scaled to variance 1 using the scale function in R. Prediction ability was calculated using the Pearson correlation coefficient between the predicted phenotype value from GBLUP and the observed phenotype value from the rescaled phenotypic means. For each stable GWAS hit, an additional model was fit that included the same GBLUP component as above, with one GWAS hit added as a fixed effect. The change in prediction ability was calculated by subtracting the model’s prediction ability of the model without the GWAS hit from the model’s prediction ability of the model that included the GWAS hit.

## 4. Conclusions

In total, our results support the development of further genomic resources to enable breeders to tailor genetic marker development and utilization within their own breeding program’s gene pool and outline the need for more field trial data with larger numbers. Multiple years or locations of data should be used to ensure the chosen model can account for genotype × environment interactions, either directly or indirectly. Genomic selection on fiber quality should be possible to implement in the near term, as long as a suitable genotyping platform for estimating genomic relationships can be identified. Although the utilization of these resources will come down to the scope and scale of each individual breeding program, uniform phenotyping and genotyping will allow breeders to share some information to better estimate marker effects and empower more accurate predictions, hopefully reducing the number of early generation progeny needed to screen for these complex traits. More data for fiber and seed cotton yield, as well as seed protein and oil content, will help determine whether or not a genomic prediction for these traits is possible in cotton. Other important traits in cotton, such as abiotic and biotic stress, theoretically have the potential for genomic prediction, although the selection efficiency will be limited by trait heritability and other practical limitations of large-scale field experiments with controlled stress conditions. To enable genomic prediction in cotton breeding at scale, the community needs a low-cost genotyping system that can be used to accurately estimate relatedness between selection candidates. Shared phenotyping methods and data tracking systems will also enable data exchange and integration, bringing genomic prediction to fruition in public cotton breeding programs sooner.

## Figures and Tables

**Table 1:** Broad-sense heritabilities and their standard errors for 20 cotton traits. Overall is calculated using data from all 14 year–locations, and Environment is the mean of heritabilities and standard errors calculated for all environments separately.

		Overall		(Mean from up to 14 Tests in Separate Environments) Environment		Difference in H^2^ (Overall-Environment)
Trait Type	Trait	Broad-Sense Heritability (H^2^)	Standard Error	Broad-Sense Heritability (H^2^)	Standard Error	
Advanced Fiber Information System (AFIS)	Fineness	0.84	0.03	0.37	0.11	0.47
	Length Top 25% Mean by Wt	0.96	0.01	0.79	0.05	0.17
	Length Mean by Number	0.92	0.01	0.47	0.11	0.45
	Immature Fiber Content	0.22	0.13	0.28	0.11	−0.06
	Maturity Ratio	0.75	0.04	0.32	0.12	0.43
	Short Fiber Content by Wt	0.86	0.03	0.41	0.09	0.45
High Volume Instrument (HVI)	Elongation	0.93	0.01	0.40	0.11	0.53
	Upper Half Mean Length	0.96	0.01	0.64	0.08	0.32
	Strength	0.96	0.01	0.69	0.07	0.27
	Micronaire	0.88	0.02	0.68	0.07	0.20
	Short Fiber Content	0.80	0.03	0.47	0.10	0.33
	Uniformity Index	0.88	0.02	0.40	0.11	0.48
Seed Composition	Seed Oil %	0.88	0.02	0.50	0.09	0.38
	Seed Protein %	0.27	0.14	0.12	0.14	0.15
Yield	Bolls/m^−2^, from Seed Cotton	0.65	0.06	0.35	0.15	0.30
	Boll Wt, from Seed Cotton	0.71	0.05	0.27	0.13	0.44
	Gin Turnout	0.75	0.01	0.69	0.07	0.27
	Lint Yield	0.75	0.04	0.52	0.12	0.23
	Seed Cotton Yield	0.55	0.08	0.48	0.12	0.07
	Seed Index	0.93	0.01	0.60	0.08	0.33
**Average**		**0.78**	**0.04**	**0.47**	**0.10**	**0.31**

AFIS traits are highlighted in teal, HVI traits in yellow, seed traits in purple, and yield traits in red. Wt = weight.

**Table 2:** Number of significant marker–trait associations discovered by genome-wide association study. “-” indicates no hits from GWAS or GWAS was not run due to near-zero heritability.

Trait Type	Trait	Overall Means	Blackville			Florence			Hartsville		Rocky Mount		Stoneville		Tifton		Average per Trait
			2004	2005	2006	2004	2005	2006	2004	2005	2005	2006	2005	2006	2005	2006	
Advanced Fiber Information System (AFIS)	Fineness	657	292	8	30	34	238	96	-	2	240	1	35	18	-	36	**112**
	Length Top 25% Mean by Wt	212	762	41	-	407	57	59	280	197	419	2	64	123	9	242	**192**
	Length Mean by Number	-	74	-	5	24	9	-	-	53	174	1	-	-	9	141	**33**
	Immature Fiber Content	-	88	-	39	17	187	199	164	10	52	58	78	9	184	112	**80**
	Maturity Ratio	3	4	58	18	-	300	32	41	-	110	9	65	13	-	150	**54**
	Short Fiber Content by Wt	1	1	5	72	21	19	112	168	44	125	-	-	9	9	37	**42**
High Volume Instrument (HVI)	Elongation	-	18	-	201	434	8	-	-	3	19	-	-	15	-	55	**50**
	Upper Half Mean Length	471	163	10	76	585	54	-	451	193	12	278	203	108	18	281	**194**
	Strength	284	41	15	30	112	15	570	35	41	47	-	364	535	31	80	**147**
	Micronaire	769	280	10	152	18	-	66	-	353	743	591	48	192	681	228	**275**
	Short Fiber Content	58	40	10	4	123	307	269	231	-	-	3	167	29	426	-	**111**
	Uniformity Index	17	30	3	-	26	726	-	-	3	-	-	1	26	112	-	**63**
Seed Composition	Seed Oil %	180	89	-	155	161	33	151	-	89	129	156	-	230	96	-	**98**
	Seed Protein %	104	21	-	16	-	299	119	-	13	-	454	-	3	24	-	**70**
Yield	Bolls/m^2^, from Seed Cotton	91	38	-	2	150	-	-	9	-	6	-	6	381	47	440	**78**
	Boll Wt, from Seed Cotton	9	105	-	10	29	-	3	33	7	22	34	30	-	4	460	**50**
	Gin Turnout	301	276	2	-	133	3	53	628	27	349	897	100	9	-	603	**225**
	Lint Yield	1007	9	38	201	192	-	-	1372	-	-	-	29	628	28	334	**256**
	Seed Cotton Yield	116	75	22	161	92	-	-	785	-	3	-	5	469	38	67	**122**
	Seed Index	605	831	11	15	1	-	278	-	168	-	732	237	8	1	815	**247**
**Average per Environment**		**244**	**162**	**12**	**59**	**128**	**113**	**100**	**210**	**60**	**123**	**161**	**72**	**140**	**86**	**204**	

Location abbreviations—BL: Blackville, SC; FL: Florence, SC; HV: Hartsville, SC; RM: Rocky Mount, NC; ST: Stoneville, MS; TFT: Tifton, Georgia. AFIS traits are highlighted in teal, HVI traits in yellow, seed traits in purple, and yield traits in red. Wt = weight.

**Table 3:** Comparison of prediction ability building the model using the overall means and predicting individual environment means.

		Prediction Ability Compared to within Year and Location (% of within Prediction Ability)			
		Within Location, Different Year	Within Year, Different Location	Different Year and Location	Interpretation
Advanced Fiber Information System (AFIS)	Fineness	95%	84%	94%	Within location is easier
	Length Mean by Number	100%	88%	96%	Within location is easier
	Length Top 25% Mean by Wt	100%	90%	98%	Within location is easier
	Immature Fiber Content	95%	63%	92%	Within location is easier
	Maturity Ratio	93%	67%	91%	Within location is easier
	Short Fiber Content by Wt	84%	72%	82%	Within location is easier
High Volume Instrument (HVI)	Elongation	95%	92%	94%	Within location is easier
	Upper Half Mean Length	100%	90%	98%	Within location is easier
	Strength	100%	95%	97%	Within location is easier
	Micronaire	67%	65%	62%	Within location is easier
	Short Fiber Content	36%	71%	47%	Within year is easier
	Uniformity Index	96%	88%	93%	Within location is easier
Seed Composition	Seed Oil %	100%	76%	100%	Within location is easier
	Seed Protein %	56%	30%	5%	Within location is easier
Yield	Bolls/m^2^, from Seed Cotton	1%	48%	29%	Within year is easier
	Boll Wt, from Seed Cotton	18%	100%	81%	Within year is easier
	Gin Turnout	98%	96%	95%	Within location is easier
	Lint Yield	49%	68%	59%	Within year is easier
	Seed Cotton Yield	13%	44%	34%	Within year is easier
	Seed Index	96%	99%	99%	Within year is easier

“Within Location, Different Year”—training and testing the model in different field trials, restricted to only those comparisons that took place at the same location but in different years; “Within Year, Different Location”—training and testing the model in different field trials, restricted to only those comparisons that occurred in the same year but in different locations; and “Different Year and Location”—training and testing the model in different field trials, restricted to only those comparisons that took place in both different years and locations. AFIS traits are highlighted in teal, HVI traits in yellow, seed traits in purple, and yield traits in red. Wt = weight.

**Table 4:** Overview of data available for each year–location.

Location	Year	Comments
Blackville, SC	2004	✔
	2005	No seed oil % or seed protein %
	2006	✔
Florence, SC	2004	✔
	2005	✔
	2006	No bolls m^−2^, boll weight, lint, or seed cotton yield
Hartsville, SC	2004	No seed oil % or seed protein %
	2005	No bolls m^−2^, boll weight, lint, or seed cotton yield
Rocky Mount, NC	2005	No high-volume instrument short fiber content
	2006	✔
Stoneville, MS	2005	No seed oil % or seed protein %
	2006	✔
Tifton, GA	2005	✔
	2006	✔

“✔” observations available for all traits.

## Data Availability

All datasets and scripts for this study are included in the Supplementary Materials or can be found in the GitHub Repository https://github.com/USDA-ARS-GBRU/PeeDeeCottonBreedingProgram_GSOutlook. Raw genotypic data are available at the CottonGen database https://www.cottongen.org/.

## Supplementary Materials

The following supporting information can be downloaded at: https://www.mdpi.com/article/10.3390/plants11111446/s1, Table S1: Table of single environment and overall heritability estimates for the extended trait set; Table S2: All GWAS results for the extended trait set; Table S3: Stable GWAS hits for the extended trait set; Table S4: Standard deviation for the point estimates of prediction ability over 100 runs of cross validation, for the extended trait set; Table S5: Difference in prediction ability for prediction between environments, for the extended trait set; Figure S1: GWAS histogram, similar to Figure 2, for the extended trait set; Figure S2: Figure showing relationship between heritability and number of stable GWAS hits for the 20-trait set; Figure S3: Prediction ability for Overall means for the extended trait set; Figure S4: Prediction ability variability for overall and environment means for the extended trait set; Figure S5: Phenotype distribution among lines for the extended trait set; Data S1: PDF containing all QQ-plots and genomic inflation factors.
