# Supplementary material for: Outlook for Implementation of Genomics-Based Selection in Public Cotton Breeding Programs
Source: Plants (Basel). 2022 May 29;11(11):1446. doi: 10.3390/plants11111446 (PMC9182660; doi:10.3390/plants11111446)
Supplement: Supplementary file 1 [file plants-11-01446-s001.zip › plants-1707252-supplementary/Supplemental_Data S1.pdf]

Trait	Mean_VIF
TFT05_L_N_MM	1.0024872
BL06_L_N_MM	1.003305
TFT05_ELO	1.0049984
BL05_B	1.0054914
OVERALL_IFC	1.005982
TFT05_L_W_MM	1.0077398
TFT05_OIL	1.0077546
HV05_GBOLLT	1.0082995
ST06_GBOLLSD	1.0090007
ST05_BOLLM2L	1.0090511
HV05_UI	1.0095377
ST05_BOLLM2S	1.0097416
RM06_UI	1.0100687
FL05_SYLD_KG	1.0103983
RM06_SFC_W	1.0106605
FL05_LYLG_KG	1.0108599
FL06_OIL	1.0110301
BL06_L5_N_MM	1.0112319
BL06_L_W_MM	1.0115292
ST06_ELO	1.0124098
HV05_SFC_W	1.0124791
ST05_RD	1.0135408
HV05_GBOLLSD	1.0137965
TFT06_B	1.0147832
RM06_IFC	1.0148859
FL05_INDEX	1.0150911
BL06_SFC_W	1.0168659
TFT05_L5_N_MM	1.017269
FL06_ELO	1.0172692
FL05_BOLLM2S	1.0186678
TFT05_UQL_W_MM	1.0189858
TFT06_SFC	1.019169
ST05_SYLD_KG	1.0208844
RM05_BOLLM2L	1.0211744
ST06_GBOLLT	1.0214013
BL06_UQL_W_MM	1.0219318
BL05_IFC	1.0222127
RM05_BOLLM2S	1.0223535
BL05_UI	1.0231544
TFT06_GBOLLT	1.0239634
TFT06_UI	1.0255349
HV05_RD	1.025558
FL04_OIL	1.0262977
FL05_BOLLM2L	1.0263097
HV05_ELO	1.0270892
ST05_SFC	1.0272893

BL06_RD	1.0278263
RM06_SFC	1.0287699
BL06_GIN	1.0296015
BL05_ELO	1.0296383
RM05_UI	1.0307096
HV04_UI	1.0307408
RM05_PROTEIN	1.0312926
RM05_ELO	1.0314008
OVERALL_OIL	1.0316467
BL04_B	1.032214
BL06_ELO	1.0322622
TFT05_INDEX	1.0353324
BL04_LYLG_KG	1.0362958
RM05_GBOLLSD	1.0366584
TFT05_GIN	1.0383087
HV05_SFC	1.0389361
BL05_BOLLM2L	1.0395614
BL05_BOLLM2S	1.0396788
HV05_L_N_MM	1.0400101
BL05_MAT_RATIO	1.0411568
ST06_L_N_MM	1.0412907
ST05_SFC_W	1.0414374
TFT05_GBOLLSD	1.0417412
BL05_GBOLLSD	1.0422305
FL05_ELO	1.0435932
ST05_GBOLLT	1.0440411
FL04_B	1.0442862
TFT06_SFC_W	1.0448
HV04_SFC	1.044835
BL06_OIL	1.0450905
FL06_MIC	1.0456233
FL06_UI	1.0464738
HV05_B	1.0470538
TFT06_GBOLLSD	1.0470935
TFT05_UHM_MM	1.0476132
BL05_RD	1.0479274
FL05_RD	1.0481167
FL06_B	1.048394
BL04_ELO	1.0506495
TFT06_IFC	1.0507595
ST06_RD	1.0513355
HV04_SFC_W	1.0514553
OVERALL_ELO	1.0526141
FL05_GIN	1.0528399
HV04_STR_KG	1.0531387
HV04_FINE_MTEX	1.0542326
BL05_FINE_MTEX	1.0542845

FL04_PROTEIN	1.0548011
RM06_OIL	1.0548709
BL06_BOLLM2S	1.0552222
OVERALL_SFC_W	1.0557307
ST05_IFC	1.0557351
BL06_BOLLM2L	1.0557576
FL06_MAT_RATIO	1.0562597
ST05_GIN	1.0563897
FL05_GBOLLSD	1.056734
OVERALL_PROTEIN	1.0569683
RM05_OIL	1.0570127
ST05_MAT_RATIO	1.0582101
TFT06_RD	1.0584608
BL06_SYLD_KG	1.0585019
BL04_OIL	1.058658
BL05_UQL_W_MM	1.0587914
FL05_SFC	1.059037
FL04_SFC_W	1.0591415
FL06_GBOLLSD	1.0591615
TFT05_STR_KG	1.0598997
ST05_L_N_MM	1.0599572
RM05_SYLD_KG	1.0603237
BL06_B	1.0604001
HV05_MAT_RATIO	1.0605566
RM05_SFC_W	1.0605961
RM06_STR_KG	1.0615483
HV04_INDEX	1.0619073
RM05_INDEX	1.0624898
HV05_OIL	1.0632215
ST05_UI	1.063307
TFT06_STR_KG	1.0657164
BL04_SYLD_KG	1.066148
RM05_IFC	1.0686358
FL06_SFC	1.069208
RM06_ELO	1.0694215
BL05_L_W_MM	1.070009
FL04_GBOLLT	1.070145
RM06_BOLLM2S	1.0706108
RM06_BOLLM2L	1.070659
OVERALL_L_N_MM	1.0707274
RM05_MAT_RATIO	1.0718692
RM06_SYLD_KG	1.0725958
BL06_LYLG_KG	1.0726523
BL05_UHM_MM	1.0732003
TFT06_L_N_MM	1.0732619
FL06_HT	1.0734686
ST06_INDEX	1.0738936

BL05_SYLD_KG	1.0758253
BL05_GBOLLT	1.0764696
TFT05_BOLLM2L	1.0768856
HV04_ELO	1.0772096
BL05_SFC_W	1.0774187
FL05_STR_KG	1.077421
BL06_UHM_MM	1.0775813
BL06_INDEX	1.07848
TFT05_BOLLM2S	1.0785576
BL05_INDEX	1.0786296
ST05_LYLG_KG	1.0790792
FL05_UQL_W_MM	1.0796191
ST06_BOLLM2L	1.0798777
HV04_MAT_RATIO	1.0799502
BL05_L5_N_MM	1.0801546
ST06_BOLLM2S	1.0801655
FL04_L_N_MM	1.0806515
ST05_L_W_MM	1.0811558
FL05_L5_N_MM	1.0817456
RM06_L_N_MM	1.0818701
BL06_SFC	1.0820693
BL04_HT	1.0821414
BL06_GBOLLT	1.0823746
BL05_L_N_MM	1.0835469
FL04_IFC	1.0861934
BL04_GBOLLT	1.086195
RM05_GBOLLT	1.0862332
RM06_PROTEIN	1.0868818
BL06_GBOLLSD	1.0870739
RM05_L_N_MM	1.0873509
BL05_LYLG_KG	1.0885023
FL04_UI	1.0888035
FL05_FINE_MTEX	1.0894737
FL05_MIC	1.0910733
BL04_BOLLM2L	1.0920027
RM06_INDEX	1.0921823
TFT06_UHM_MM	1.0928787
RM05_L_W_MM	1.0931439
RM06_GBOLLT	1.093284
HV05_L_W_MM	1.0945836
ST06_OIL	1.0958362
FL05_OIL	1.0970014
BL05_GIN	1.0976509
ST06_GIN	1.0992494
FL05_GBOLLT	1.0996392
ST06_B	1.099803
BL06_STR_KG	1.0999755

HV05_GIN	1.1006279
FL04_HT	1.1009653
BL06_HT	1.1012476
FL06_UHM_MM	1.1019889
OVERALL_MAT_RATIO	1.1024961
OVERALL_UI	1.1045961
HV05_STR_KG	1.1047626
RM06_LYLG_KG	1.1054299
BL04_IFC	1.105651
BL04_RD	1.1060524
ST06_FINE_MTEX	1.1065748
ST05_INDEX	1.1072909
HV05_IFC	1.1095589
ST05_ELO	1.1108493
ST06_PROTEIN	1.1122805
HV05_INDEX	1.1126845
ST05_UQL_W_MM	1.1140565
TFT05_LYLG_KG	1.1147723
BL06_PROTEIN	1.1148862
TFT06_L_W_MM	1.1152049
OVERALL_GBOLLSD	1.1158831
BL06_UI	1.1164021
OVERALL_RD	1.1165989
FL05_SFC_W	1.1172729
HV05_FINE_MTEX	1.1177177
HV05_UQL_W_MM	1.1178556
FL04_GIN	1.1182762
FL06_FINE_MTEX	1.1185897
TFT06_FINE_MTEX	1.1203235
BL04_UI	1.1211439
TFT06_UQL_W_MM	1.121689
RM05_LYLG_KG	1.1221334
FL05_UHM_MM	1.1239596
ST05_FINE_MTEX	1.1256397
FL05_L_N_MM	1.1266379
HV04_BOLLM2L	1.1271869
BL05_STR_KG	1.1277953
HV05_L5_N_MM	1.1280077
HV04_L_N_MM	1.128095
FL05_L_W_MM	1.1285695
OVERALL_BOLLM2L	1.1299499
TFT06_SYLD_KG	1.1307713
BL04_GBOLLSD	1.1310084
RM06_RD	1.1314781
RM06_B	1.131675
ST06_L_W_MM	1.1325519
FL04_MAT_RATIO	1.1342652

BL04_PROTEIN	1.1344984
RM06_L5_N_MM	1.1354155
FL06_IFC	1.1356893
RM06_L_W_MM	1.1360109
HV05_UHM_MM	1.1371168
FL04_FINE_MTEX	1.1375465
BL06_FINE_MTEX	1.1376407
RM05_FINE_MTEX	1.1376568
ST05_MIC	1.1390594
RM05_UQL_W_MM	1.1412748
ST05_UHM_MM	1.1420778
OVERALL_HT	1.142289
FL06_UQL_W_MM	1.142903
RM05_B	1.1444754
FL06_L_W_MM	1.1490302
TFT05_GBOLLT	1.1490537
TFT06_MIC	1.1492985
BL04_BOLLM2S	1.1493422
OVERALL_L_W_MM	1.1499643
BL05_MIC	1.1506043
FL04_SFC	1.1506687
RM06_UQL_W_MM	1.1513794
TFT06_INDEX	1.1514623
FL05_IFC	1.1517159
RM05_L5_N_MM	1.1518012
BL04_MAT_RATIO	1.1520899
BL06_MAT_RATIO	1.1565958
TFT06_ELO	1.1575446
FL06_PROTEIN	1.1582035
RM06_UHM_MM	1.158748
ST05_L5_N_MM	1.1588168
RM06_MIC	1.159147
ST06_MIC	1.1613234
ST06_SYLD_KG	1.1619148
BL06_MIC	1.1623966
HV04_BOLLM2S	1.1633046
FL06_L5_N_MM	1.1637499
FL06_INDEX	1.1644288
FL06_GBOLLT	1.1665753
BL04_STR_KG	1.1667326
FL04_L5_N_MM	1.1672309
TFT06_LYLG_KG	1.1673262
FL04_L_W_MM	1.1679305
FL04_L25_N_MM	1.169045
TFT06_L5_N_MM	1.1690779
TFT05_SYLD_KG	1.1694511
FL05_PROTEIN	1.1700269

FL04_BOLLM2L	1.1702925
TFT05_RD	1.1707624
RM05_GIN	1.17168
FL04_INDEX	1.1734736
TFT05_SFC	1.1749529
BL05_SFC	1.1784157
FL04_UQL_W_MM	1.1787115
OVERALL_B	1.1800014
OVERALL_UQL_W_MM	1.1815788
ST06_L5_N_MM	1.1836398
OVERALL_BOLLM2S	1.1847728
ST06_LYLG_KG	1.1853262
BL04_L_W_MM	1.1858686
FL06_GIN	1.1862026
BL06_IFC	1.1873082
FL06_L_N_MM	1.1884963
ST05_STR_KG	1.1897746
FL04_GBOLLSD	1.1906302
BL04_L_N_MM	1.1911903
BL04_SFC	1.1923541
TFT06_MAT_RATIO	1.1925964
BL04_SFC_W	1.1926505
ST06_UQL_W_MM	1.1926595
FL05_MAT_RATIO	1.1937789
ST06_UI	1.1940139
FL05_B	1.1959009
OVERALL_GBOLLT	1.1962197
FL04_MIC	1.1975131
RM06_GBOLLSD	1.1990957
ST06_UHM_MM	1.2101256
OVERALL_GIN	1.2106886
FL06_STR_KG	1.2107108
BL04_MIC	1.2113032
FL04_STR_KG	1.2118851
BL04_L25_N_MM	1.2157559
OVERALL_L5_N_MM	1.2197423
TFT06_GIN	1.2216089
HV04_GIN	1.2229001
FL05_UI	1.2233506
FL04_UHM_MM	1.2265901
ST06_STR_KG	1.2295236
FL04_LYLG_KG	1.2338351
FL04_ELO	1.2369882
HV04_UQL_W_MM	1.2374405
HV04_L_W_MM	1.2385827
TFT05_MIC	1.2392305
OVERALL_INDEX	1.2425476

ST05_B	1.242991
BL04_UQL_W_MM	1.2433608
OVERALL_UHM_MM	1.243739
OVERALL_STR_KG	1.249369
BL04_INDEX	1.2532996
OVERALL_SYLD_KG	1.2534643
RM05_UHM_MM	1.2537098
FL04_RD	1.2558153
BL04_L5_N_MM	1.257557
FL04_SYLD_KG	1.2626972
RM06_GIN	1.2628952
FL06_SFC_W	1.2643487
HV04_L5_N_MM	1.2675018
RM05_MIC	1.2686336
HV04_SYLD_KG	1.2724866
HV04_L25_N_MM	1.279154
OVERALL_L25_N_MM	1.2808041
TFT05_B	1.2811961
OVERALL_MIC	1.2841634
BL04_FINE_MTEX	1.2852295
OVERALL_FINE_MTEX	1.2865485
TFT06_BOLLM2S	1.2907824
TFT06_BOLLM2L	1.2916159
OVERALL_SFC	1.29764
HV04_UHM_MM	1.3079806
BL04_UHM_MM	1.3112512
OVERALL_LYLG_KG	1.3280639
HV04_LYLG_KG	1.3352244
RM05_STR_KG	1.3380136
HV05_MIC	1.338553
BL04_GIN	1.3477448
FL04_BOLLM2S	1.3735503

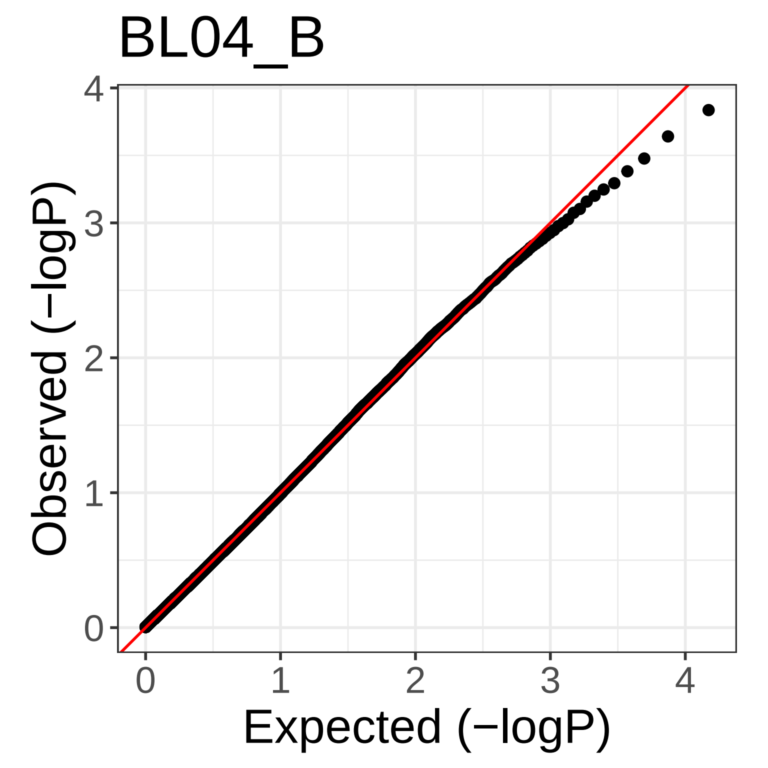
BL04_B
Observed (−logP)
Expected (−logP)
0
1
2
3
4
0
1
2
3
4

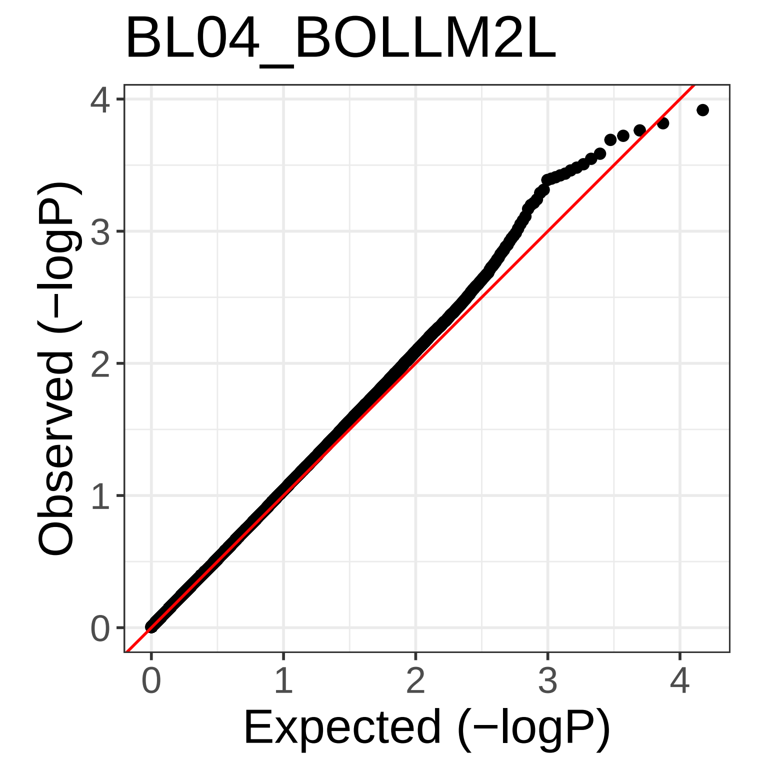
BL04_BOLLM2L
Observed (−logP)
Expected (−logP)
0
1
2
3
4

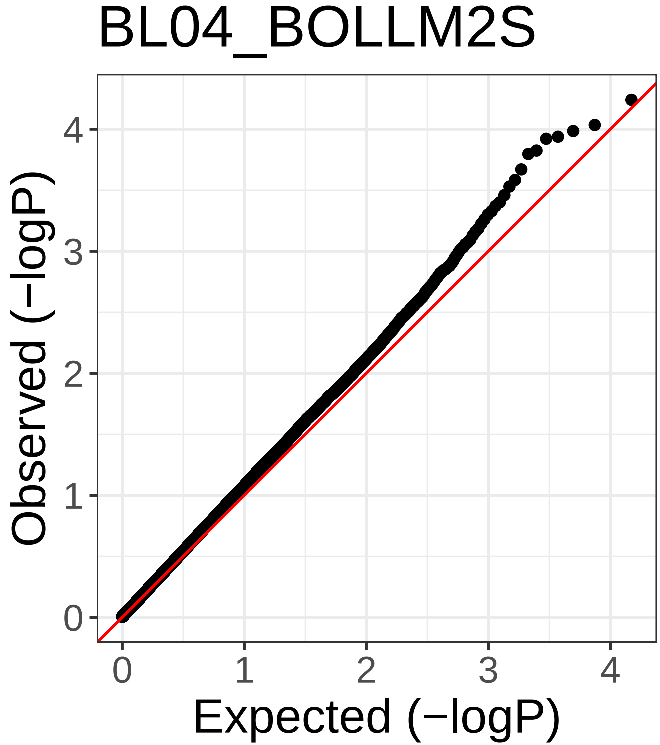
BL04_BOLLM2S
Observed (−logP)
Expected (−logP)
0
1
2
3
4
0
1
2
3
4

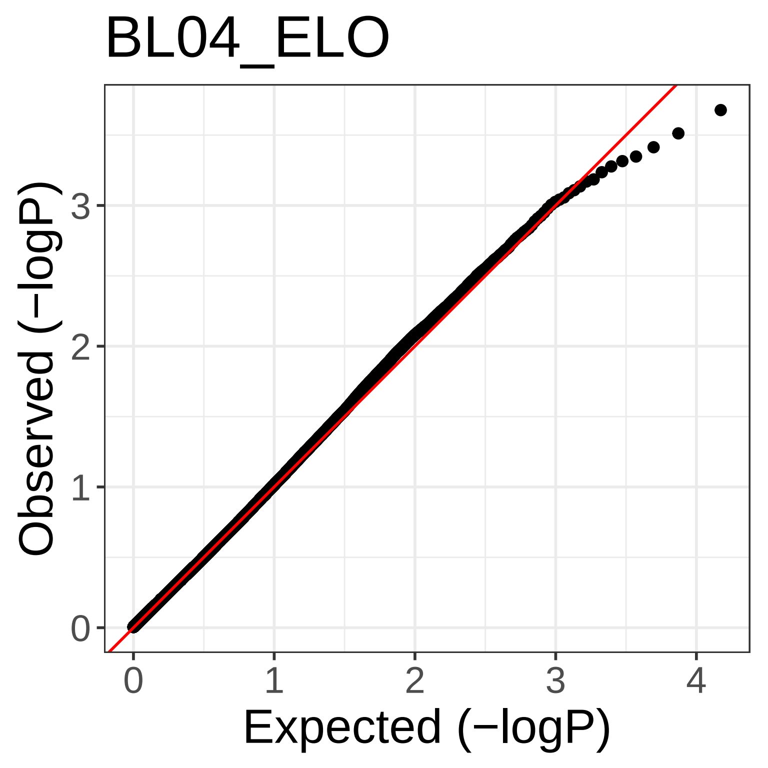
BL04_ELO
Observed (−logP)
Expected (−logP)
0
1
2
3
0
1
2
3
4

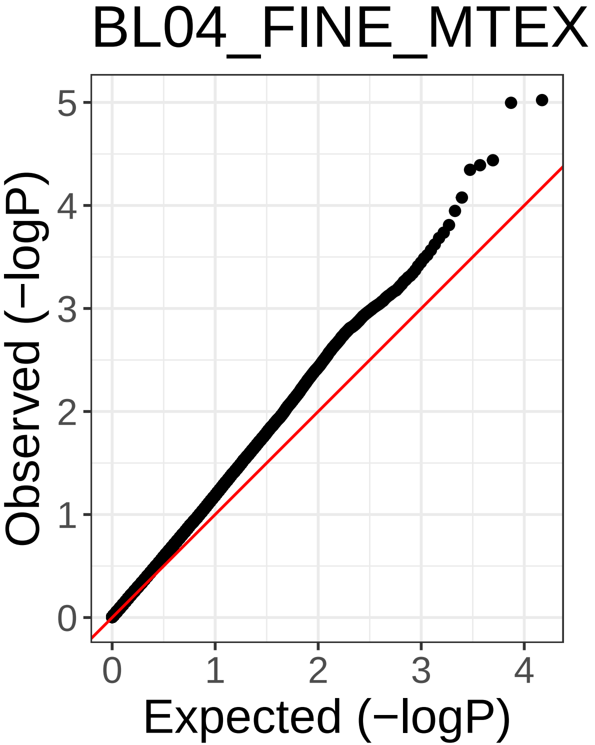
BL04_FINE_MTEX
Observed (−logP)
Expected (−logP)
0
1
2
3
4
5
0
1
2
3
4

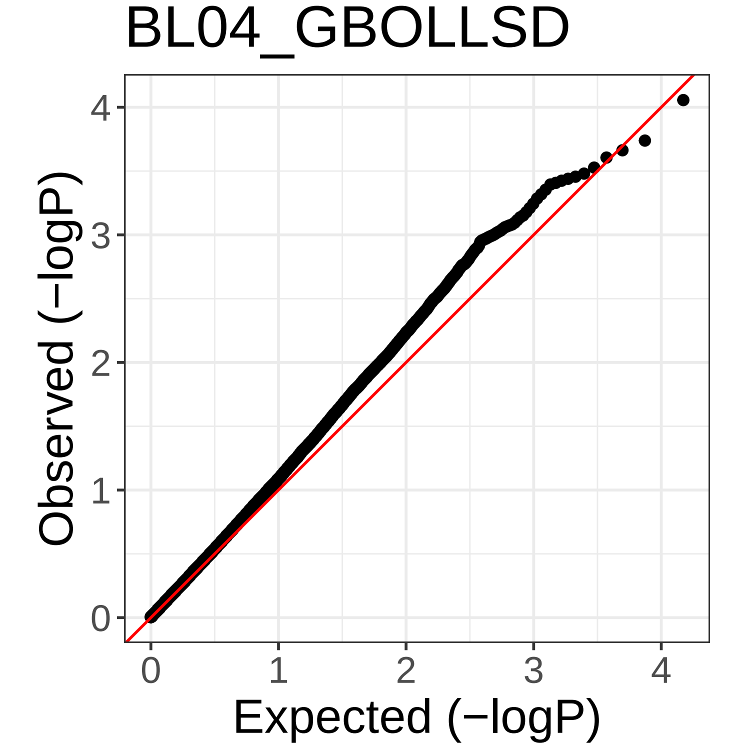
BL04_GBOLLSD
Observed (−logP)
Expected (−logP)
0
1
2
3
4
0
1
2
3
4

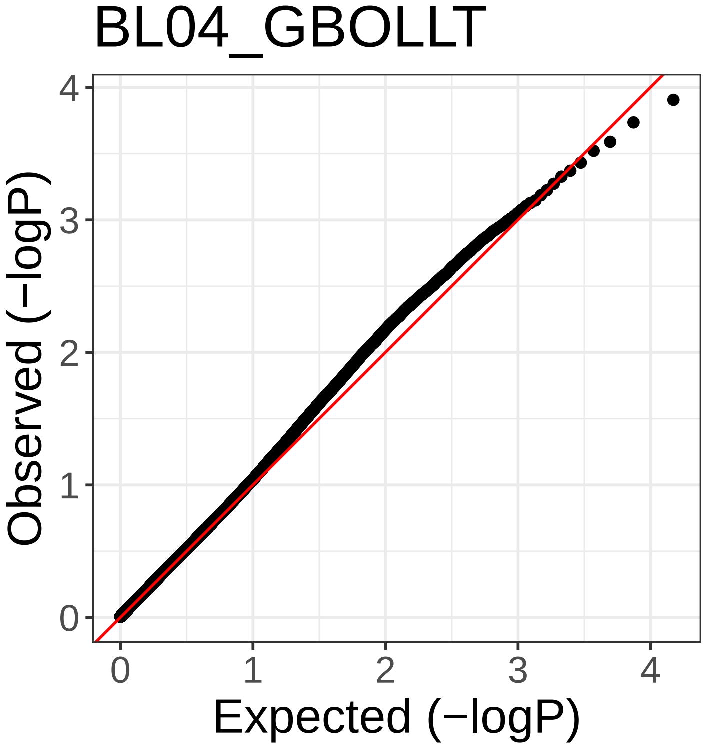
BL04_GBOLLT
Observed (−logP)
Expected (−logP)
0
1
2
3
4
0
1
2
3
4

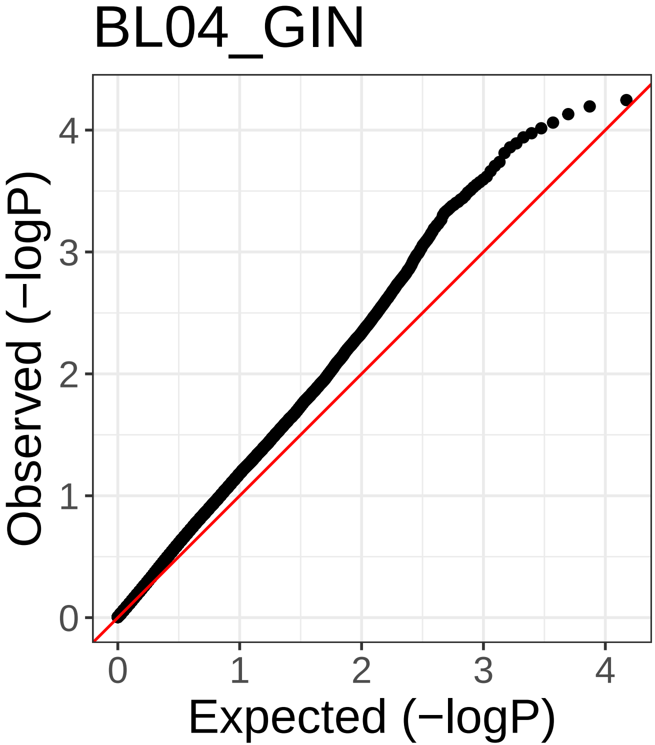
BL04_GIN
Observed (−logP)
Expected (−logP)
0
1
2
3
4
0
1
2
3
4

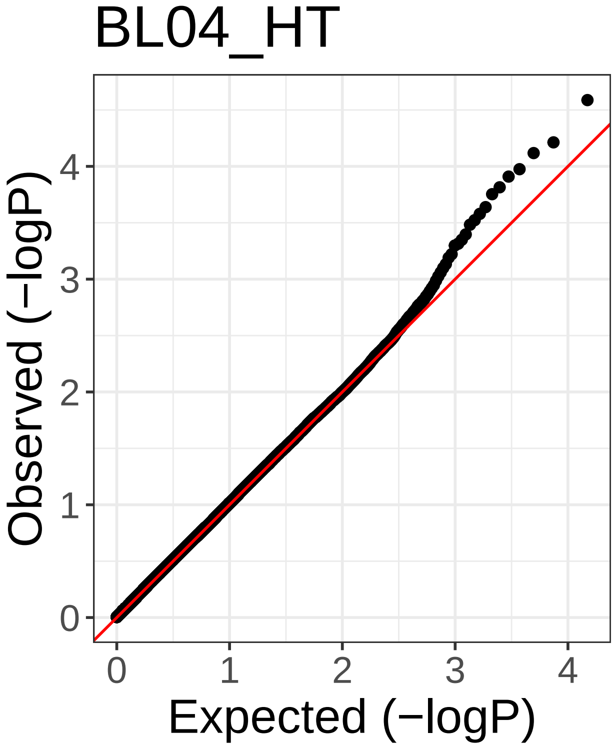

BL04_HT
Observed (−logP)
Expected (−logP)
0
1
2
3
4
0
1
2
3
4

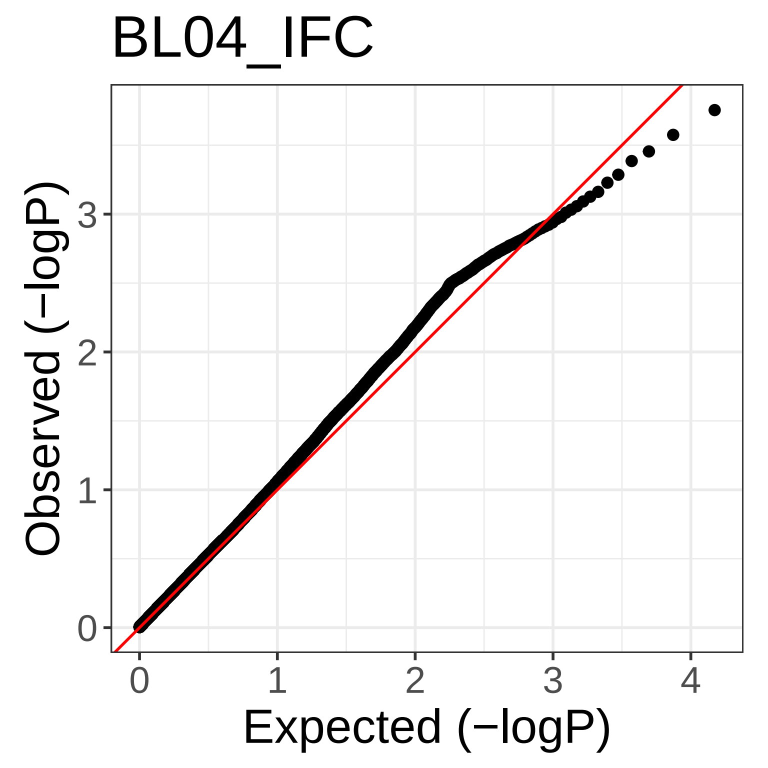
BL04_IFC
Observed (−logP)
Expected (−logP)
0
1
2
3
4

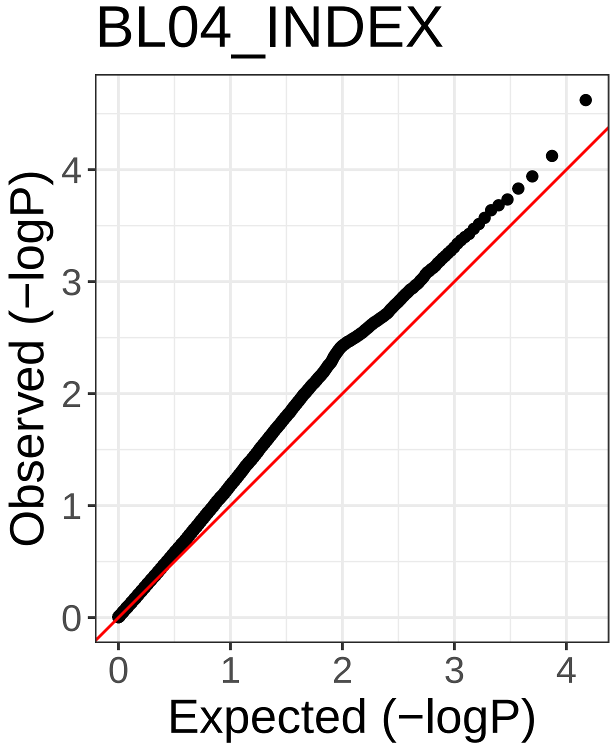
BL04_INDEX
Observed (−logP)
Expected (−logP)
0
1
2
3
4
0
1
2
3
4

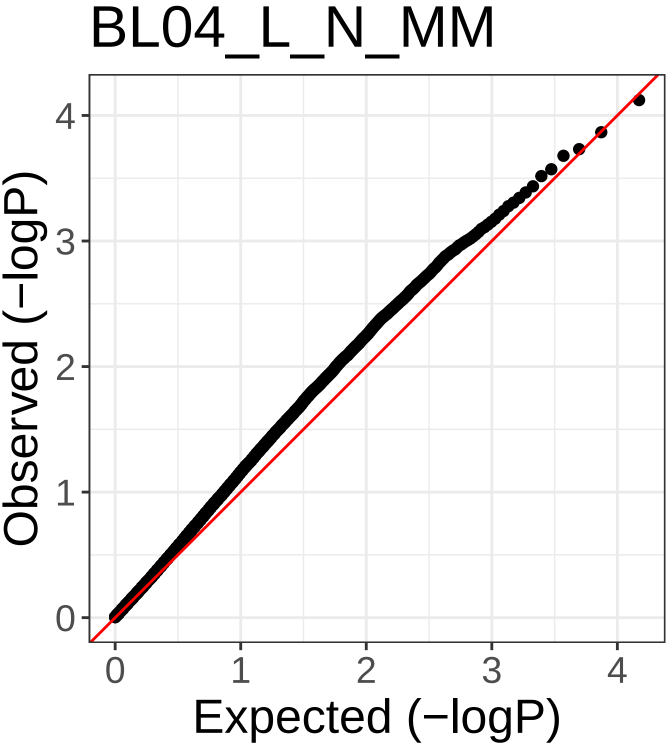
BL04_L_N_MM
Observed (−logP)
Expected (−logP)
0
1
2
3
4
0
1
2
3
4

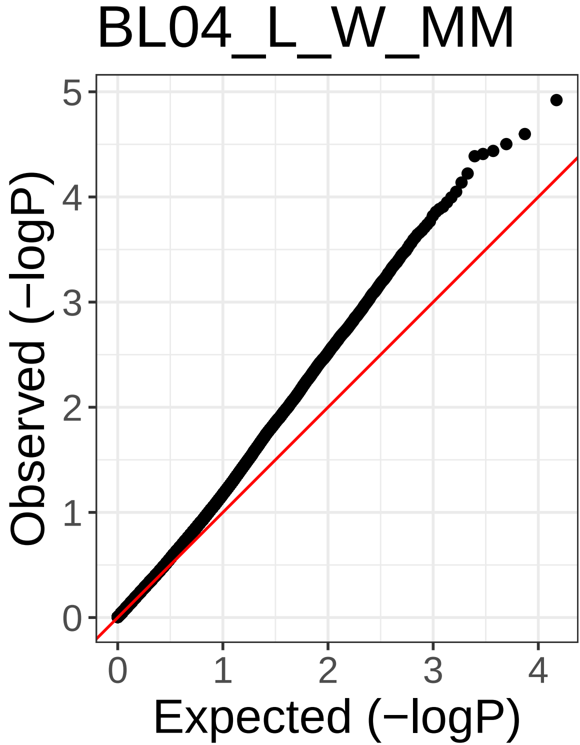

BL04_L_W_MM
Observed (−logP)
Expected (−logP)
0
1
2
3
4
5
0
1
2
3
4

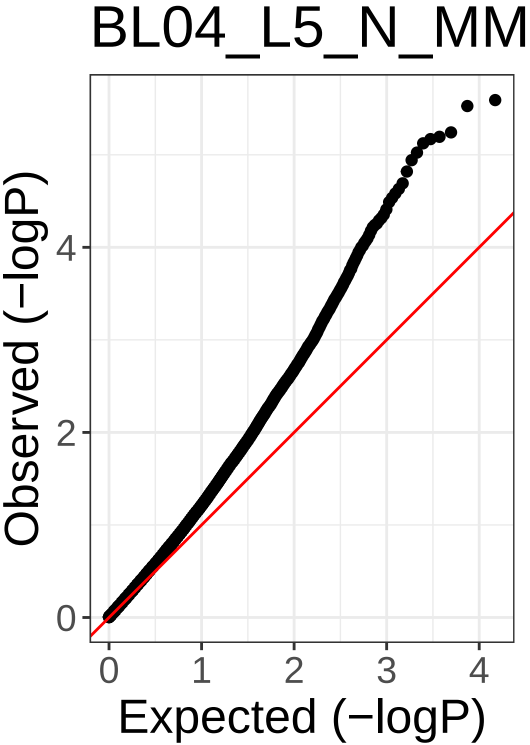

BL04_L5_N_MM
Observed (−logP)
Expected (−logP)
0
2
4
0
1
2
3
4

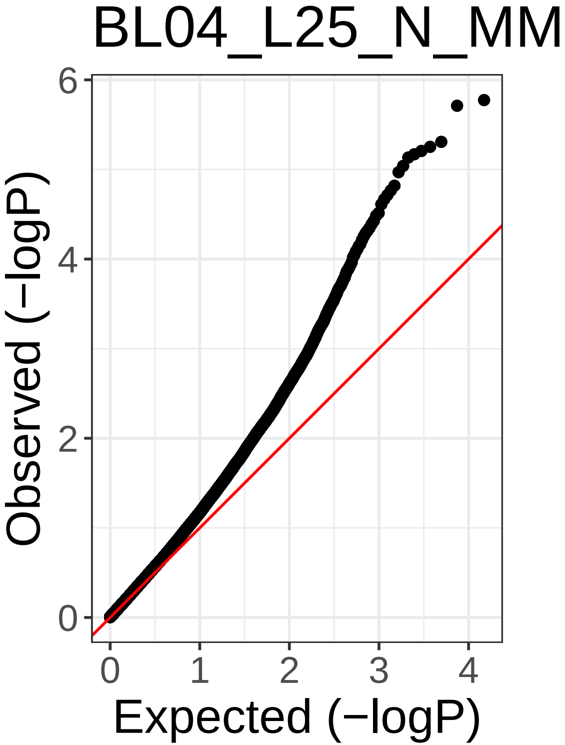
BL04_L25_N_MM
Observed (−logP)
Expected (−logP)
0
2
4
6
0
1
2
3
4

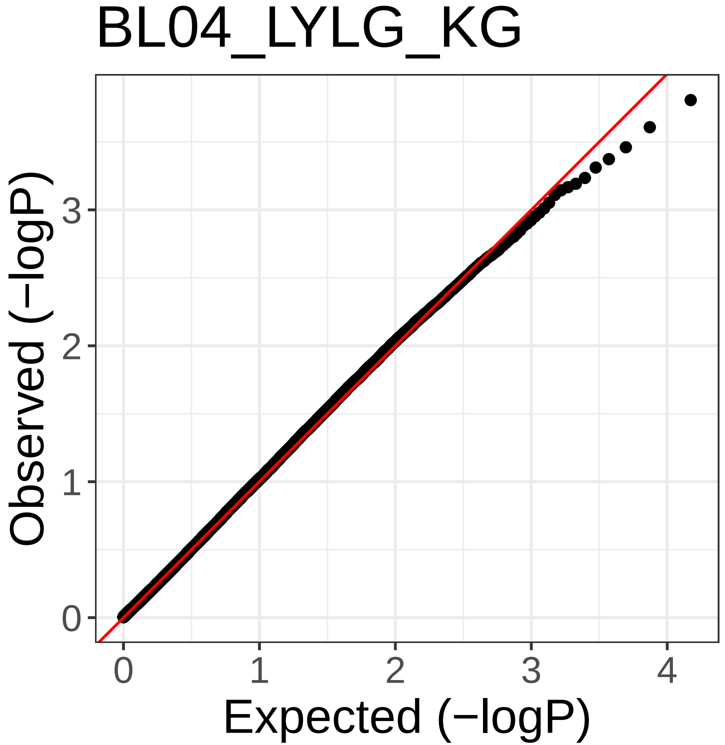

BL04_LYLG_KG
Observed (−logP)
0
1
2
3
Expected (−logP)
0
1
2
3
4

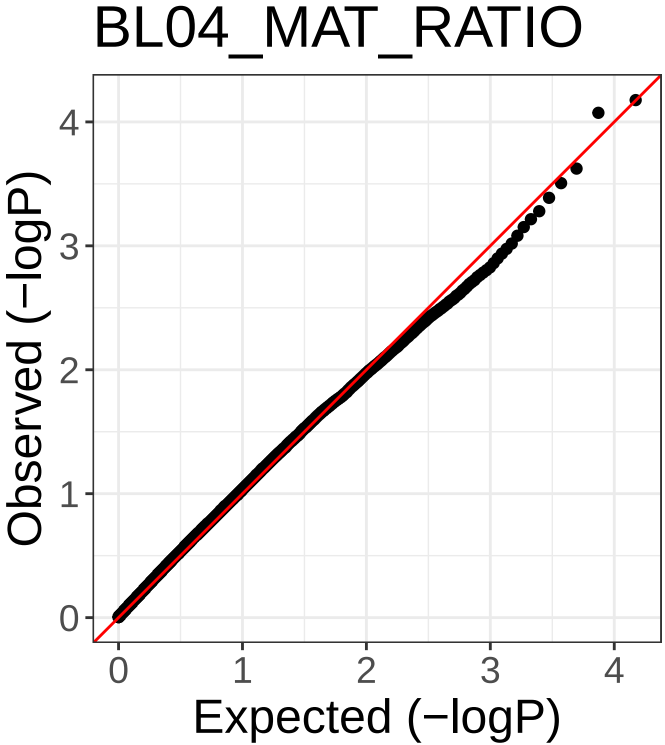
BL04_MAT_RATIO
Observed (−logP)
Expected (−logP)
0
1
2
3
4
0
1
2
3
4

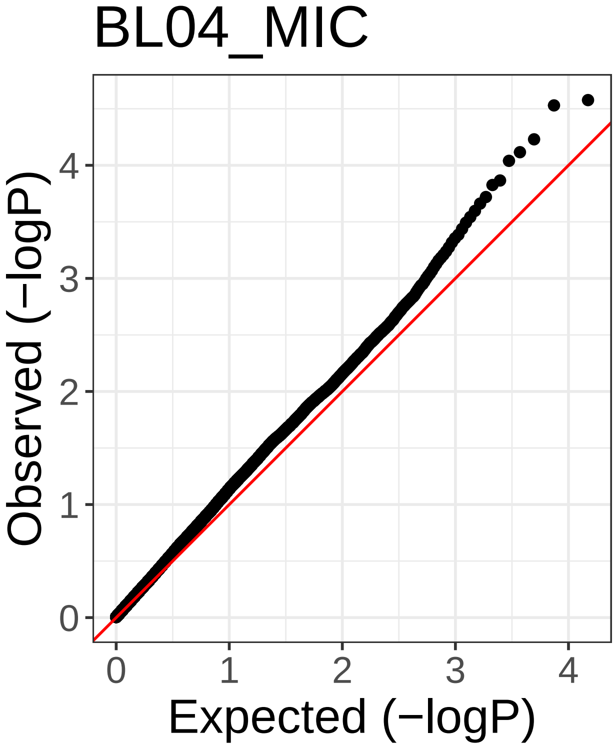
BL04_MIC
Observed (−logP)
Expected (−logP)
0
1
2
3
4
0
1
2
3
4

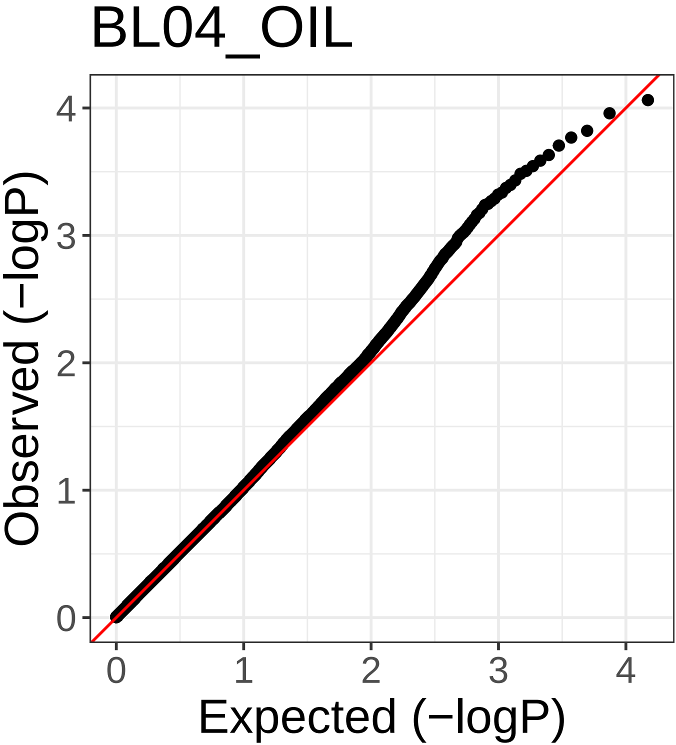
BL04_OIL
Observed (−logP)
Expected (−logP)
0
1
2
3
4
0
1
2
3
4

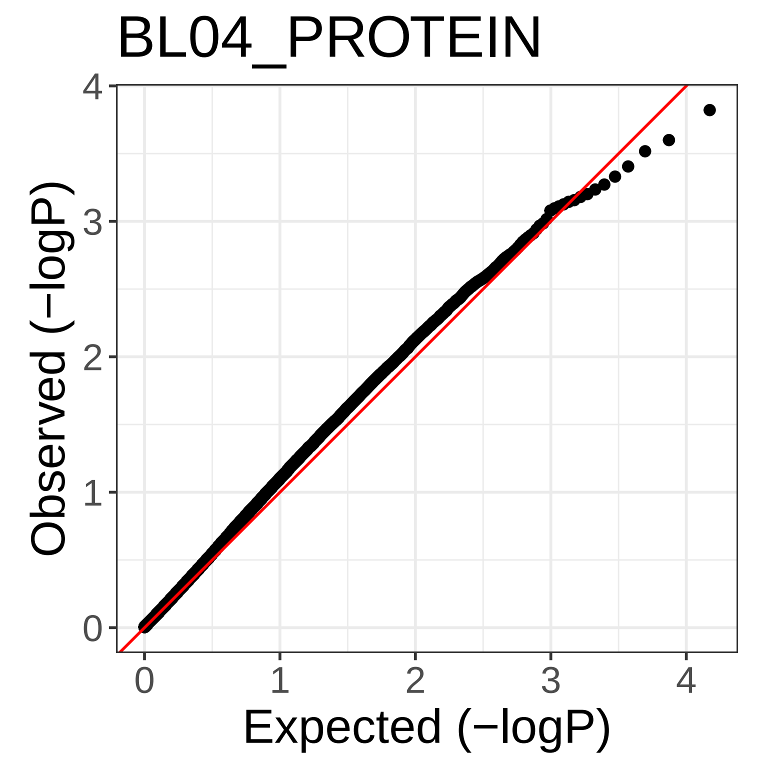
BL04_PROTEIN
Observed (−logP)
Expected (−logP)
0
1
2
3
4
0
1
2
3
4

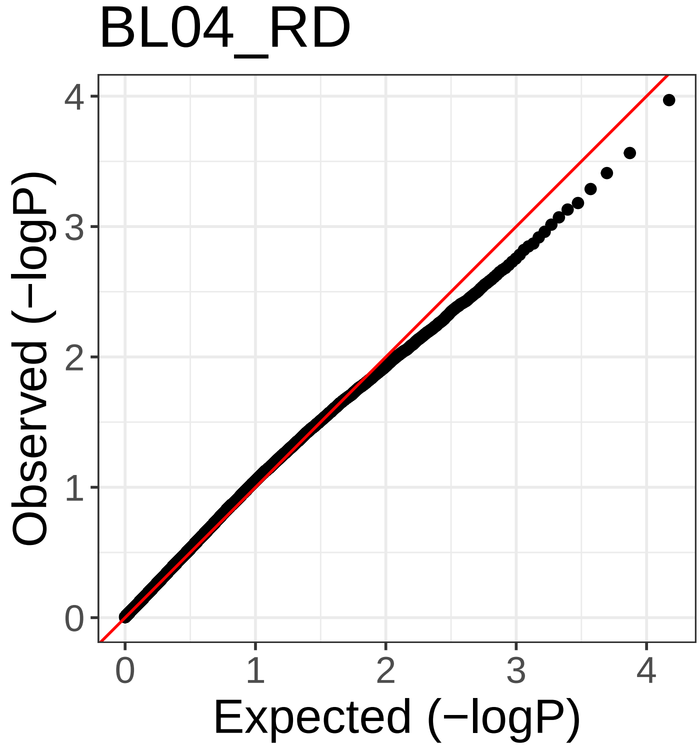
BL04_RD
Observed (−logP)
Expected (−logP)
0
1
2
3
4

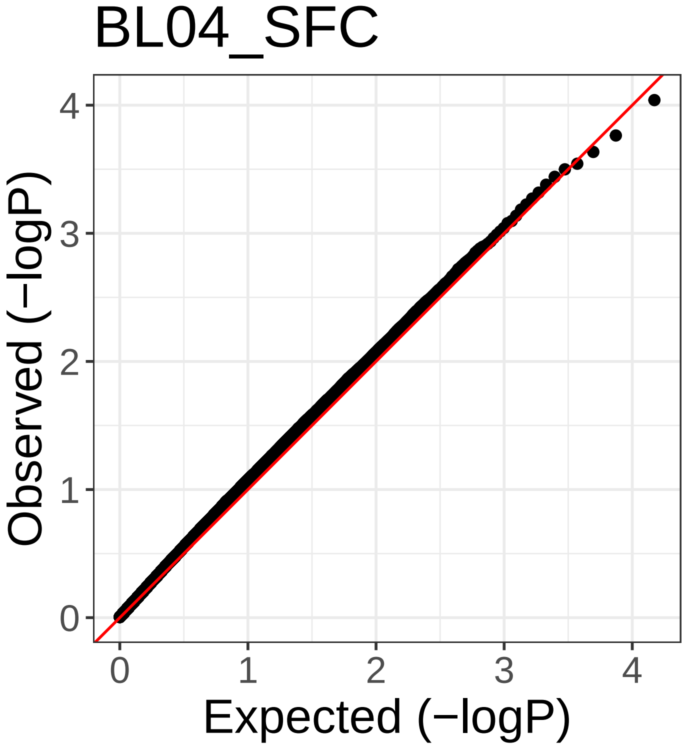
BL04_SFC
Observed (−logP)
Expected (−logP)
0
1
2
3
4
0
1
2
3
4

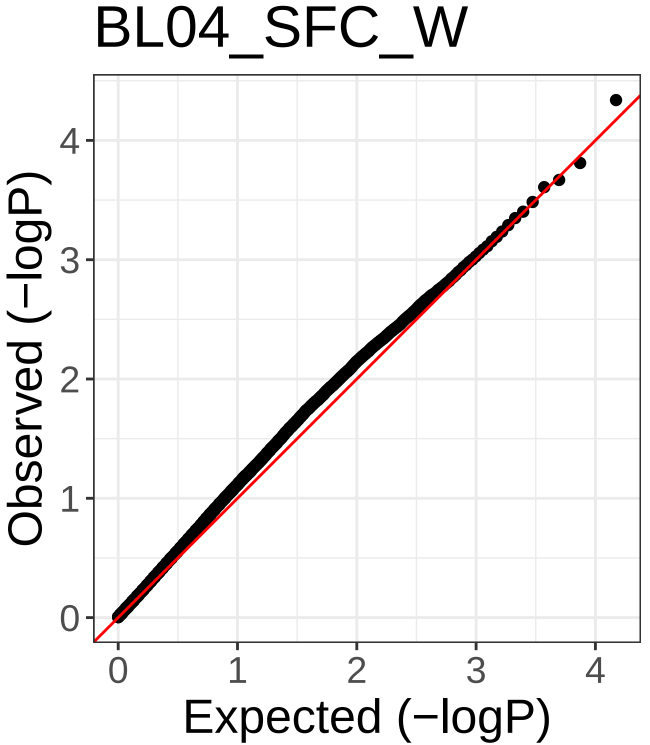
BL04_SFC_W
Observed (−logP)
Expected (−logP)
0
1
2
3
4

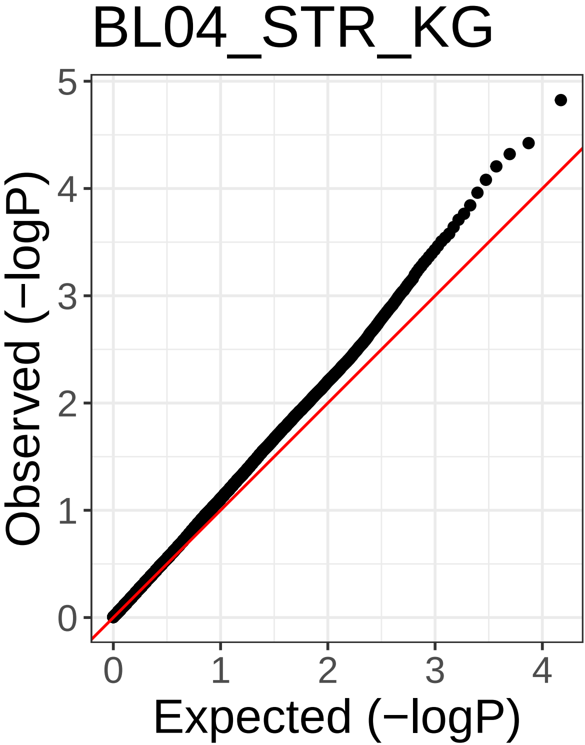

BL04_STR_KG
Observed (−logP)
Expected (−logP)
0
1
2
3
4
5

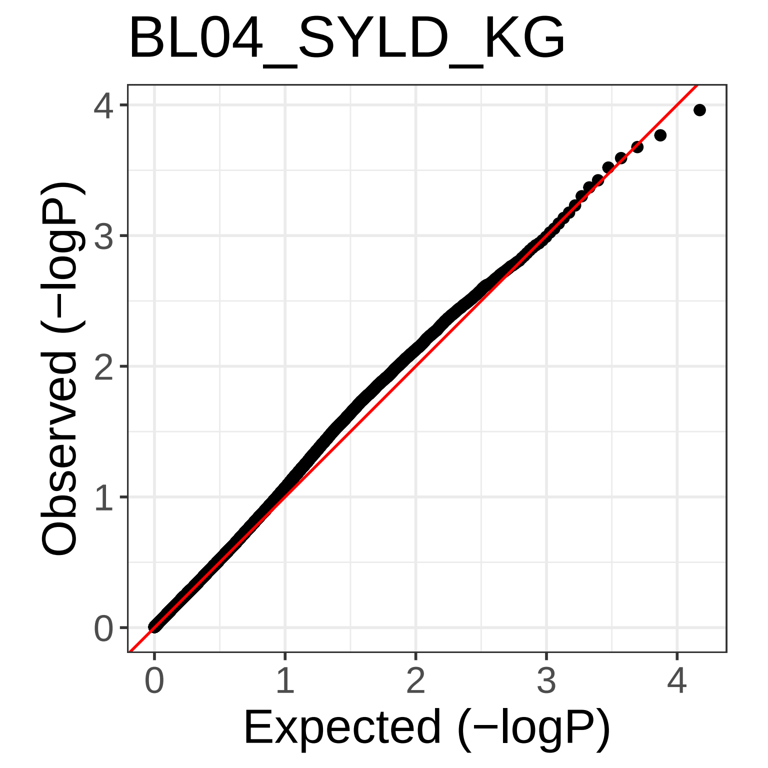
BL04_SYLD_KG
Observed (−logP)
Expected (−logP)
0
1
2
3
4
0
1
2
3
4

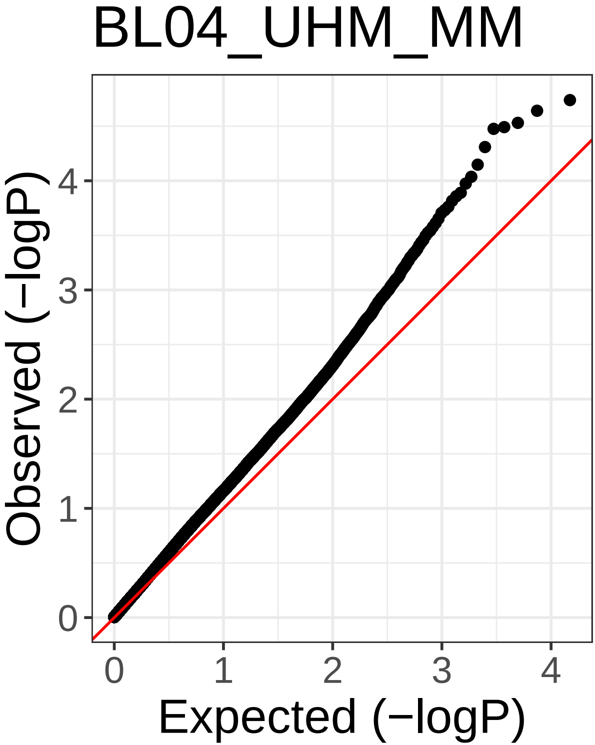

BL04_UHM_MM
Observed (−logP)
Expected (−logP)
0
1
2
3
4
0
1
2
3
4

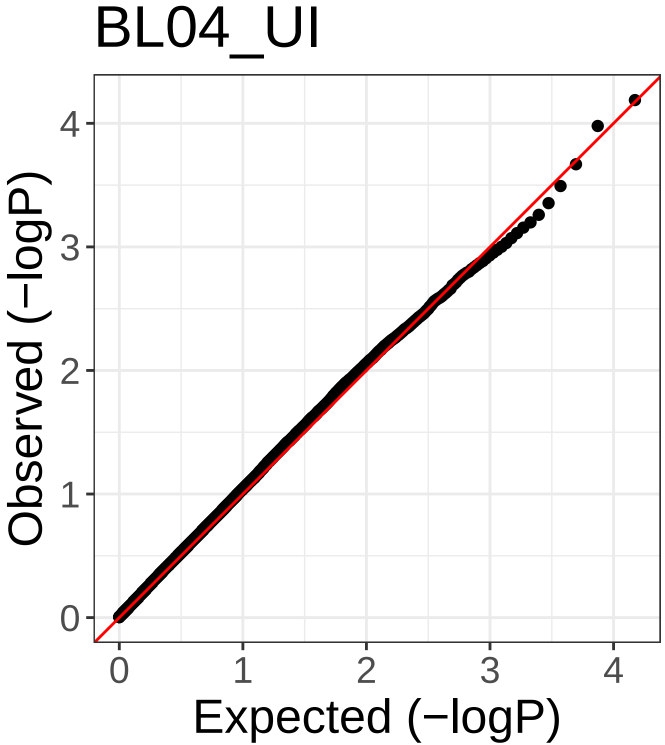
BL04_UI
Observed (−logP)
Expected (−logP)
0
1
2
3
4
0
1
2
3
4

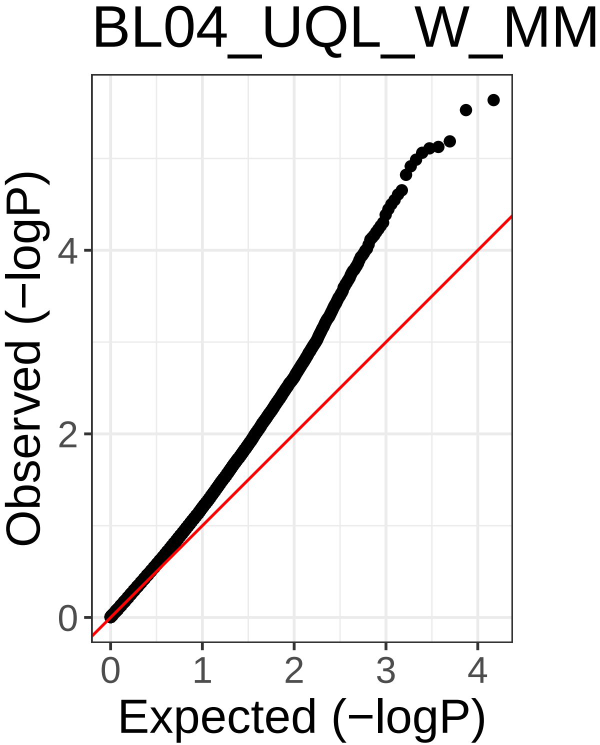
BL04_UQL_W_MM
Observed (−logP)
Expected (−logP)
0
2
4
0
1
2
3
4

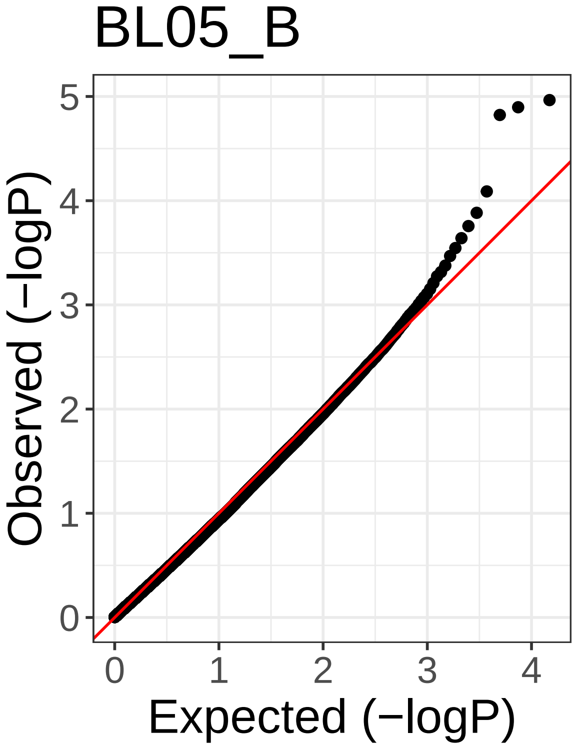
BL05_B
Observed (−logP)
Expected (−logP)
0
1
2
3
4
5
0
1
2
3
4

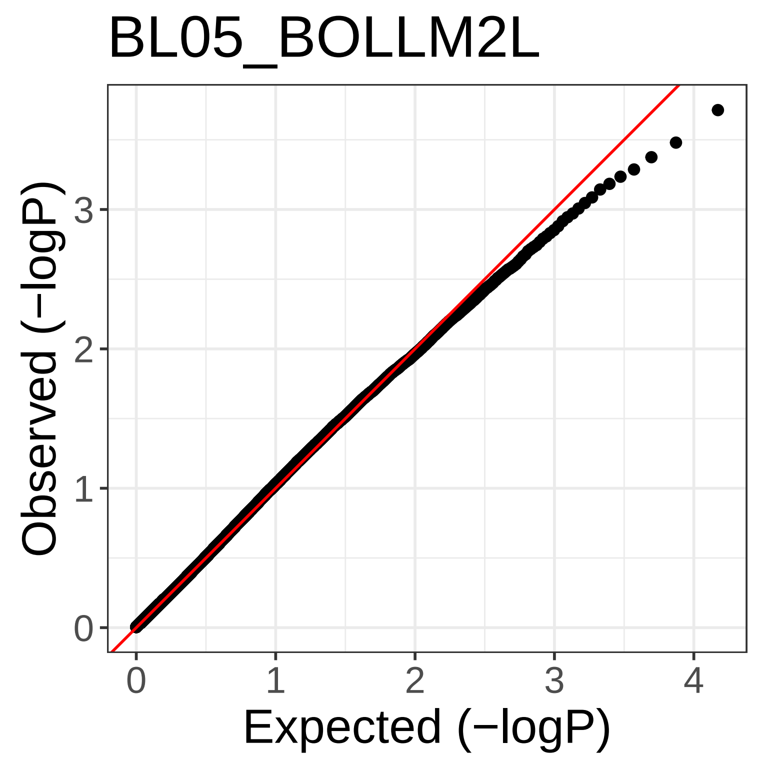

BL05_BOLLM2L
Observed (−logP)
Expected (−logP)
0
1
2
3
0
1
2
3
4

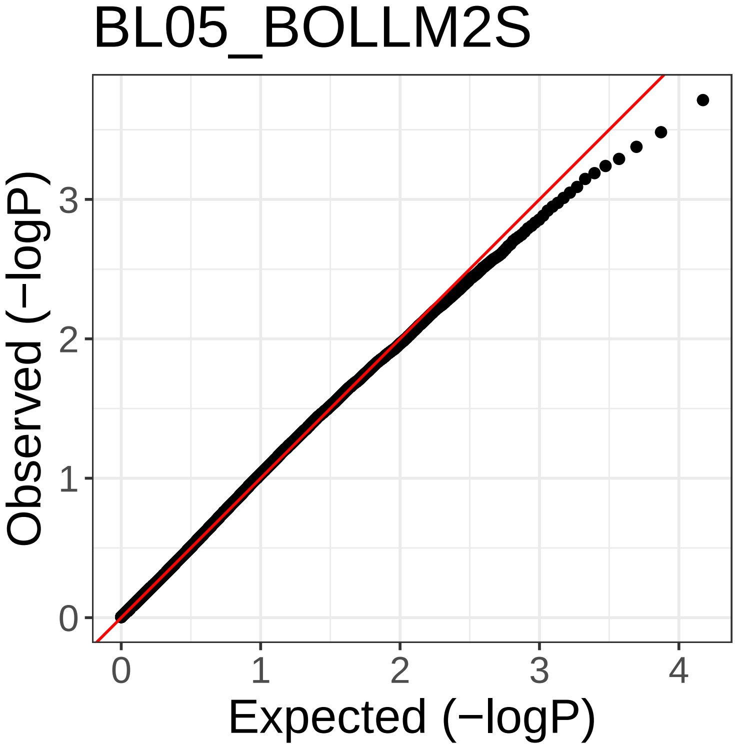
BL05_BOLLM2S
Observed (−logP)
Expected (−logP)
0
1
2
3
0
1
2
3
4

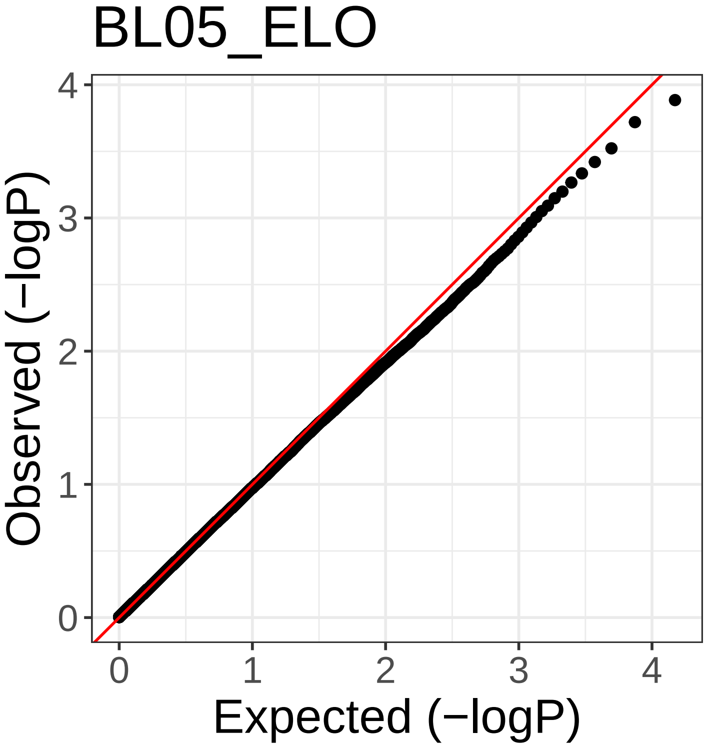
BL05_ELO
Observed (−logP)
Expected (−logP)
0
1
2
3
4
0
1
2
3
4

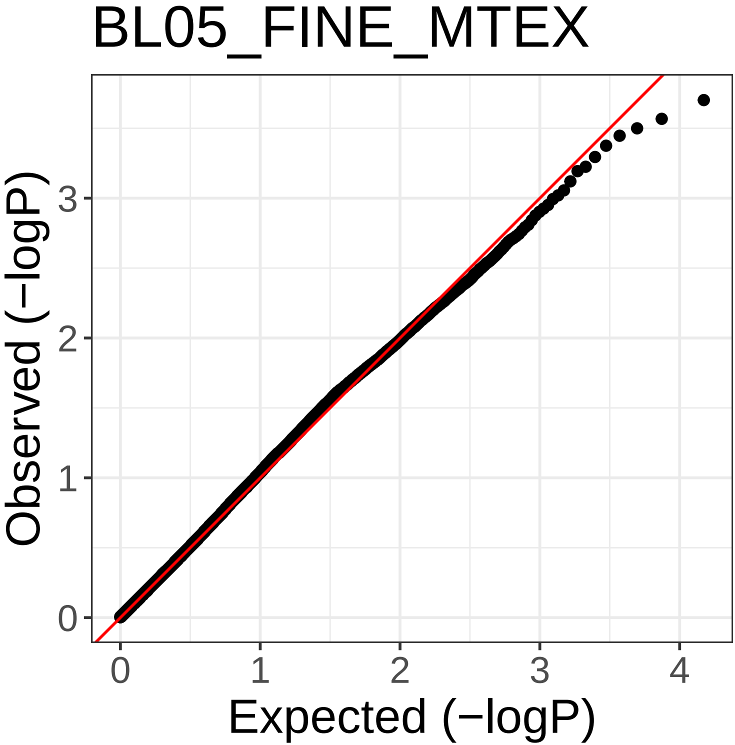

BL05_FINE_MTEX
Observed (−logP)
Expected (−logP)
0
1
2
3
0
1
2
3
4

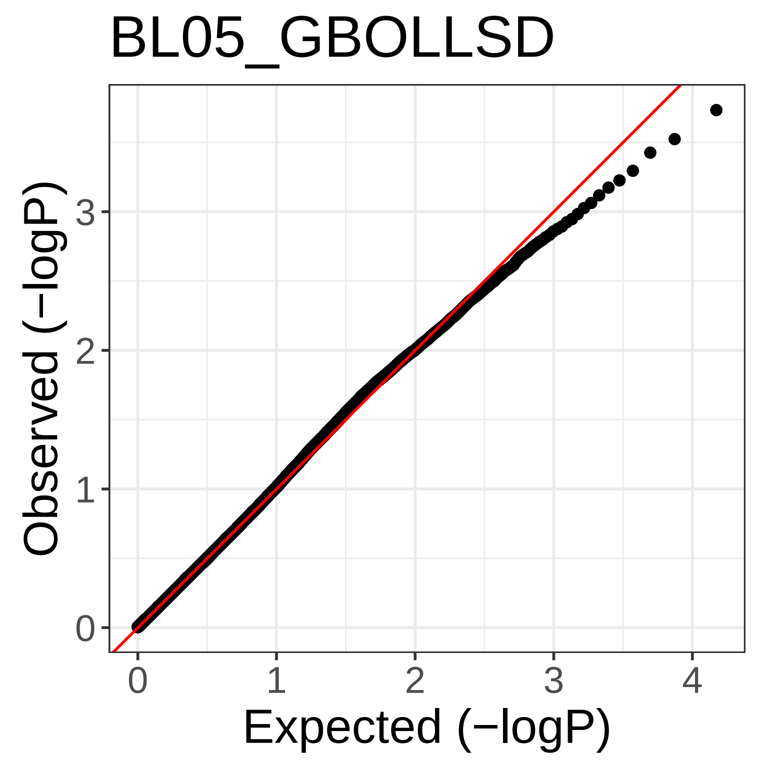
BL05_GBOLLSD
Observed (−logP)
Expected (−logP)
0
1
2
3
4

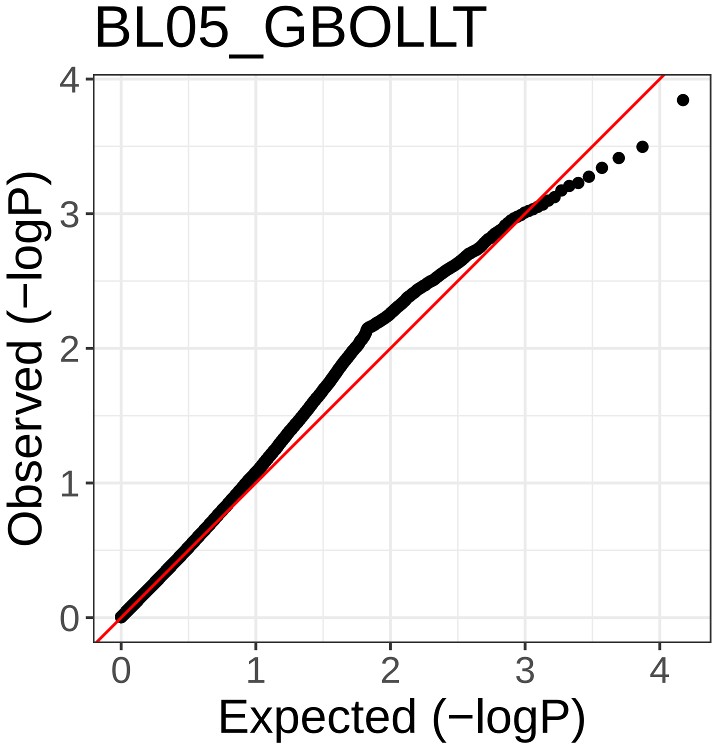
BL05_GBOLLT
Observed (−logP)
Expected (−logP)
0
1
2
3
4
0
1
2
3
4

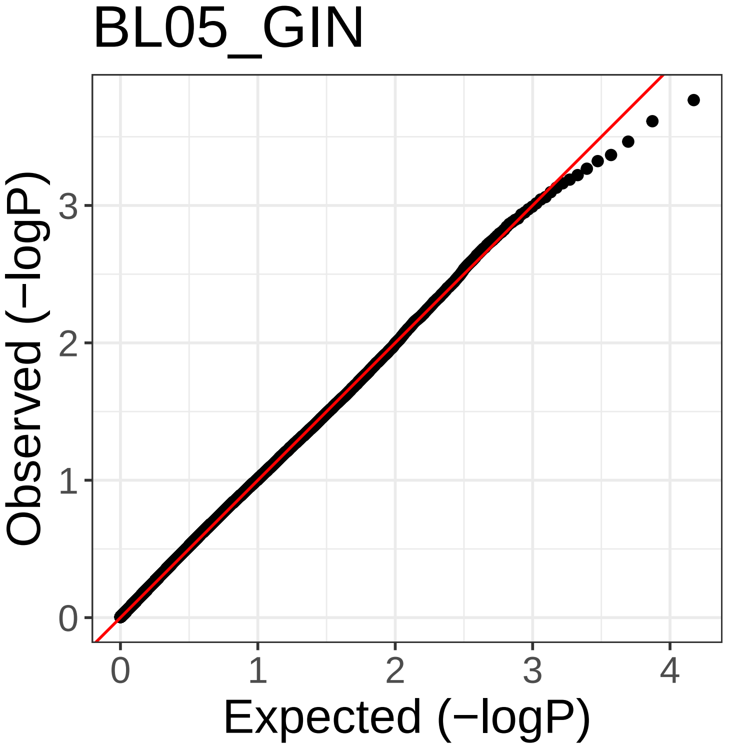
BL05_GIN
Observed (−logP)
Expected (−logP)
0
1
2
3
4

BL05_IFC

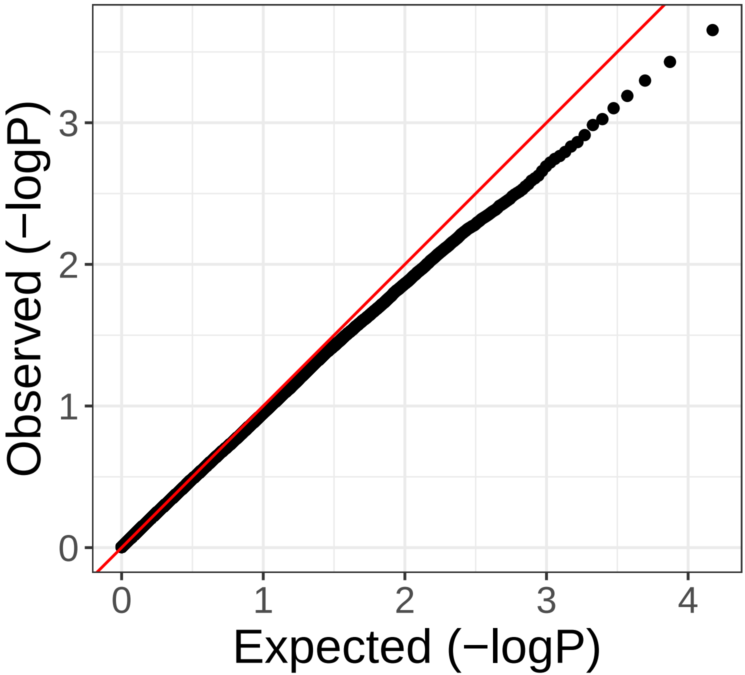

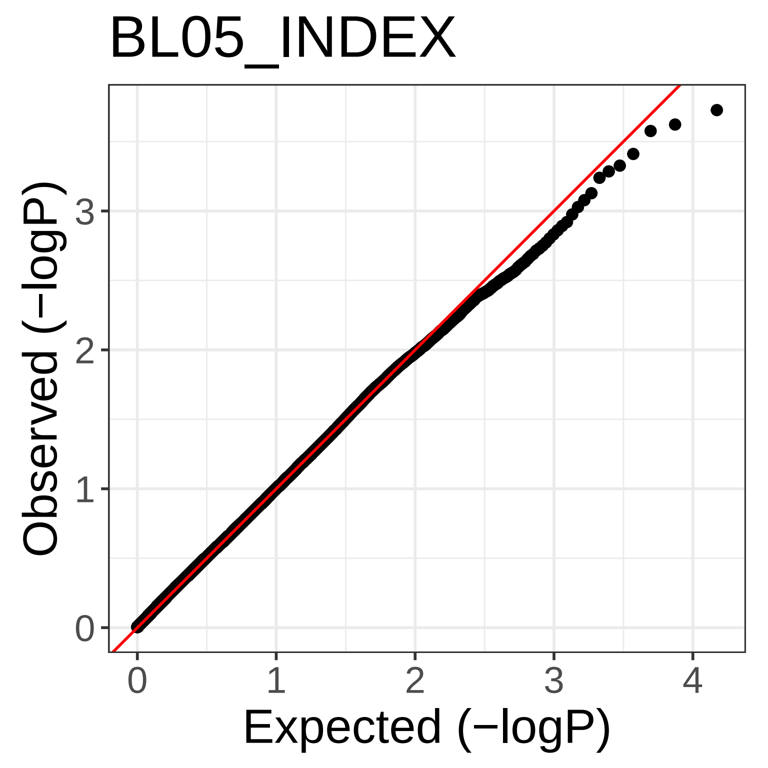
BL05_INDEX
Observed (−logP)
Expected (−logP)
0
1
2
3
4

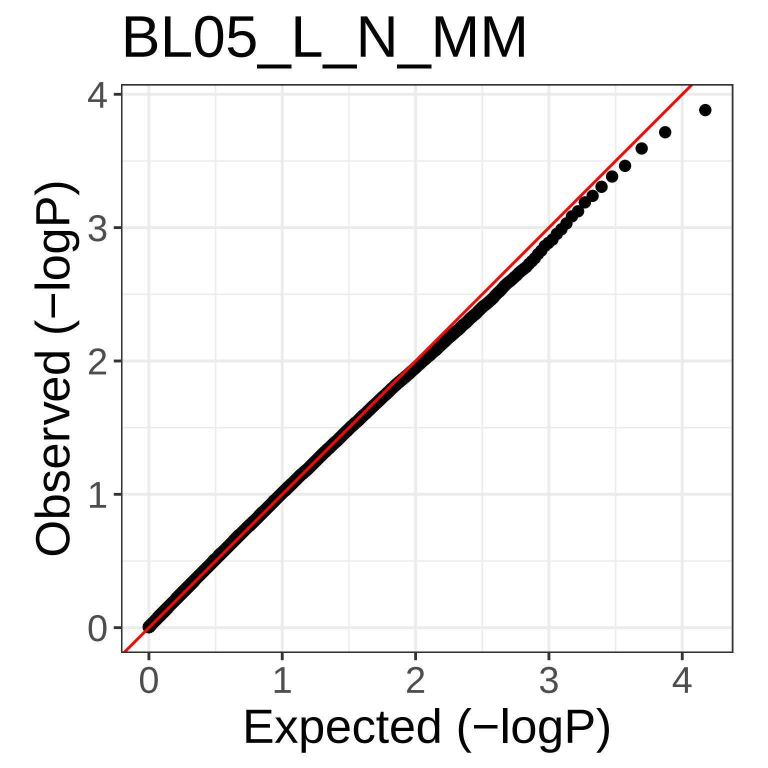
BL05_L_N_MM
Observed (−logP)
Expected (−logP)
0
1
2
3
4
0
1
2
3
4

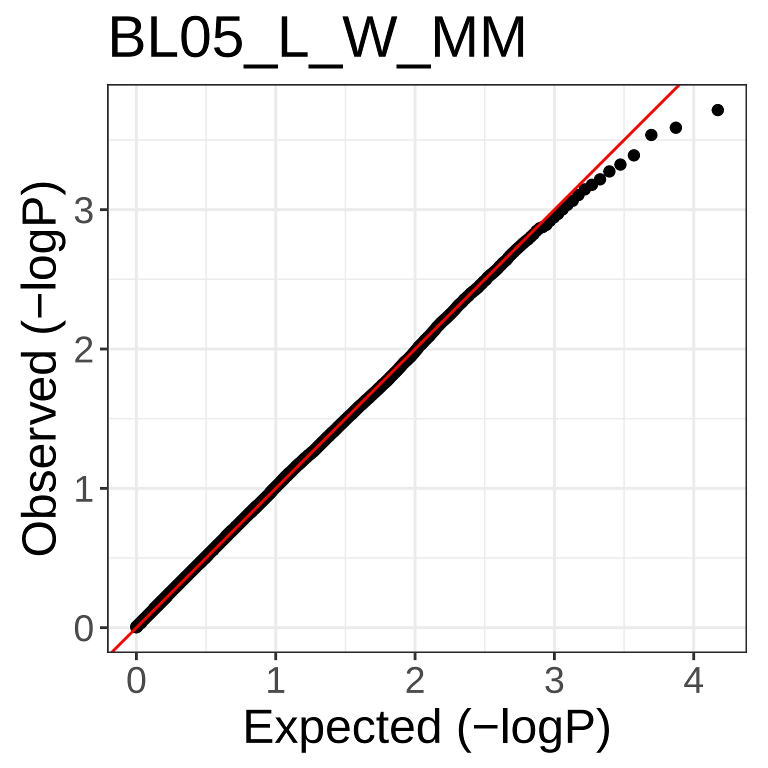

BL05_L_W_MM
Observed (−logP)
Expected (−logP)
0
1
2
3
4

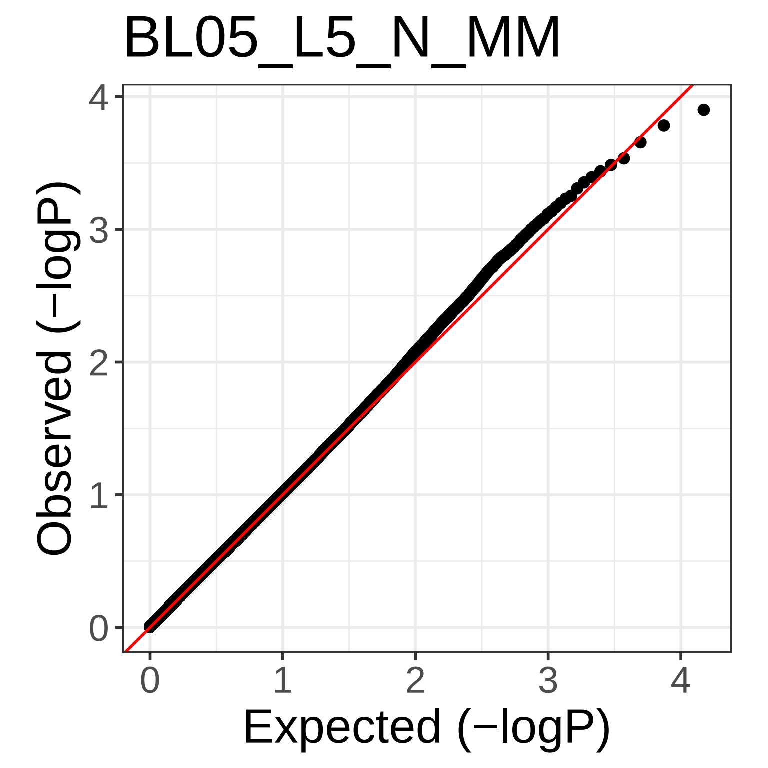
BL05_L5_N_MM
Observed (−logP)
Expected (−logP)
0
1
2
3
4

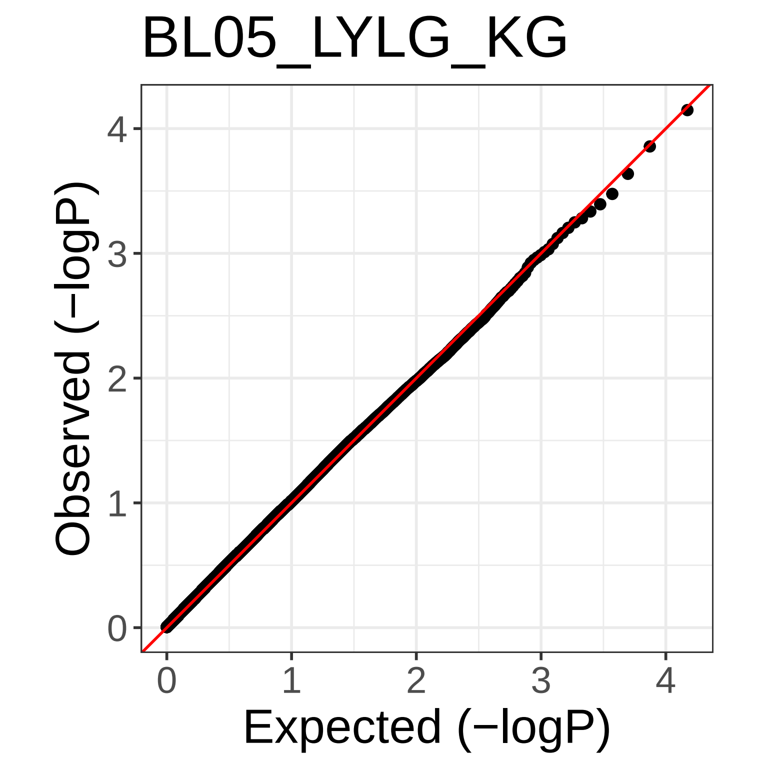
BL05_LYLG_KG
Observed (−logP)
Expected (−logP)
0
1
2
3
4
0
1
2
3
4

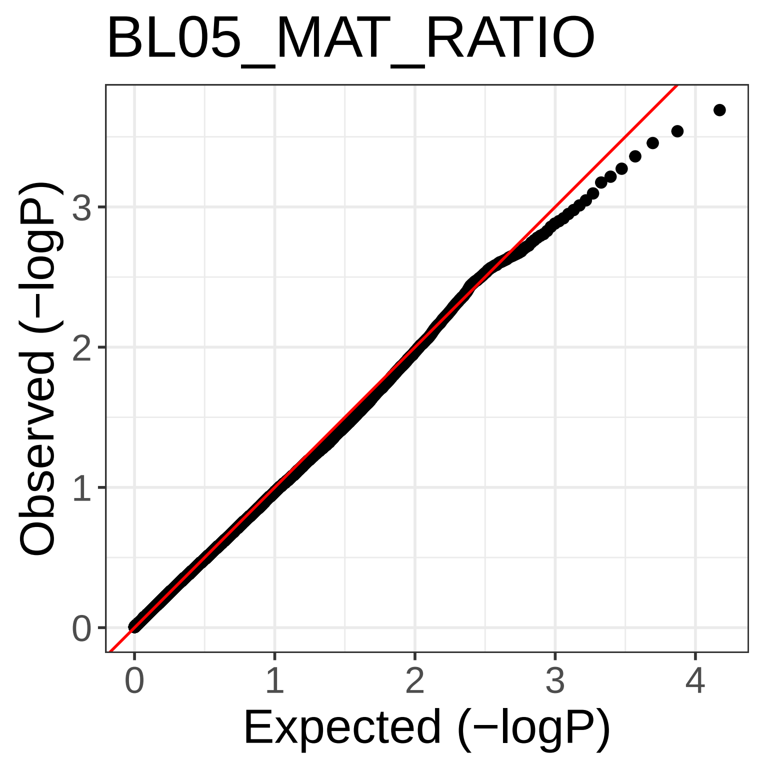
BL05_MAT_RATIO
Observed (−logP)
Expected (−logP)
0
1
2
3
4

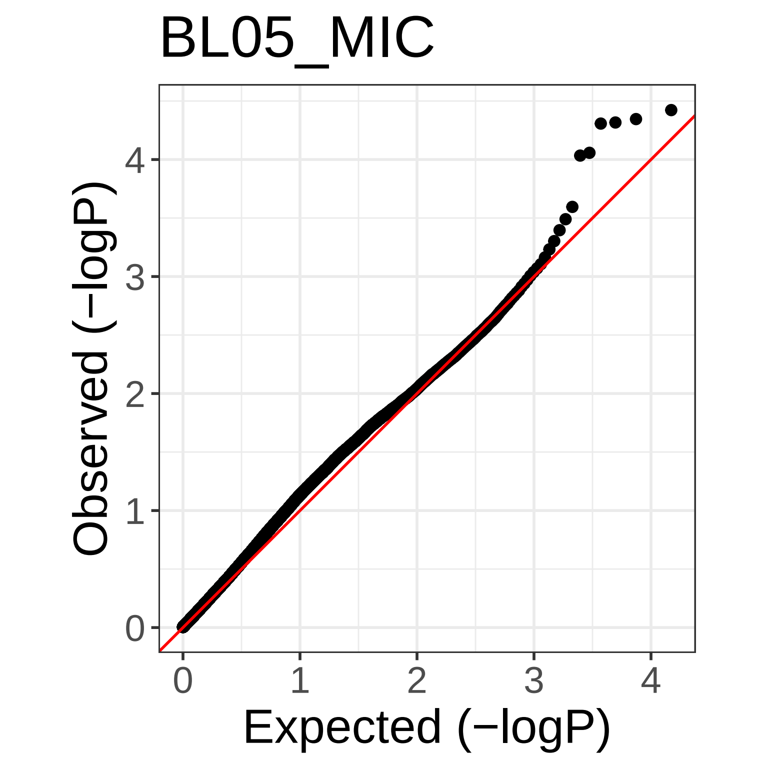
BL05_MIC
Observed (−logP)
Expected (−logP)
0
1
2
3
4

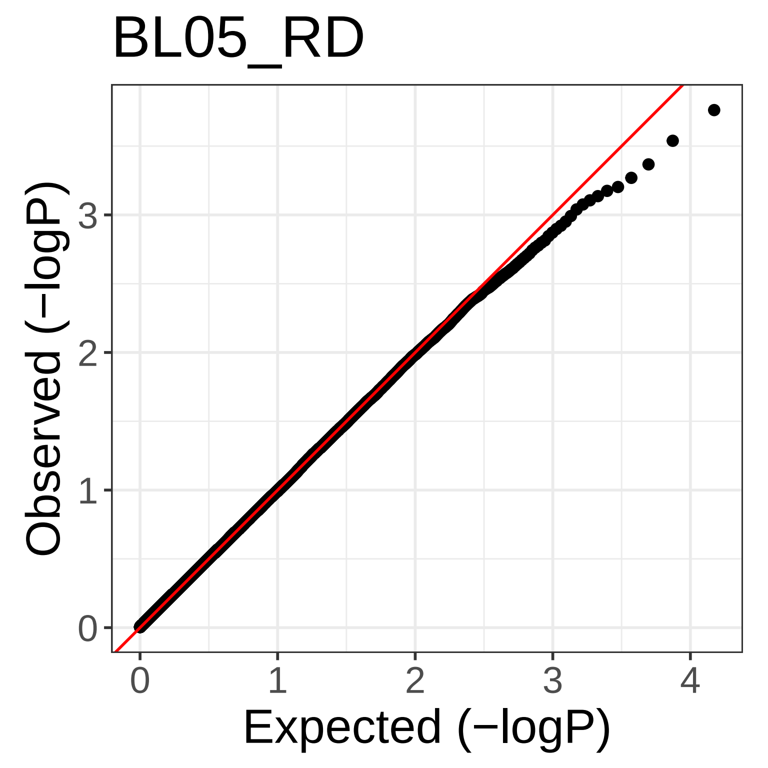
BL05_RD
Observed (−logP)
Expected (−logP)
0
1
2
3
4

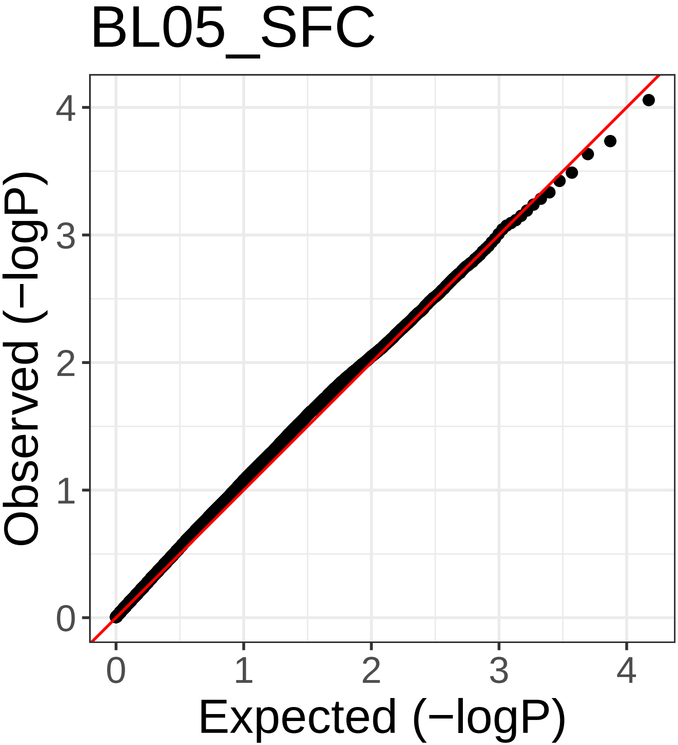
BL05_SFC
Observed (−logP)
Expected (−logP)
0
1
2
3
4
0
1
2
3
4

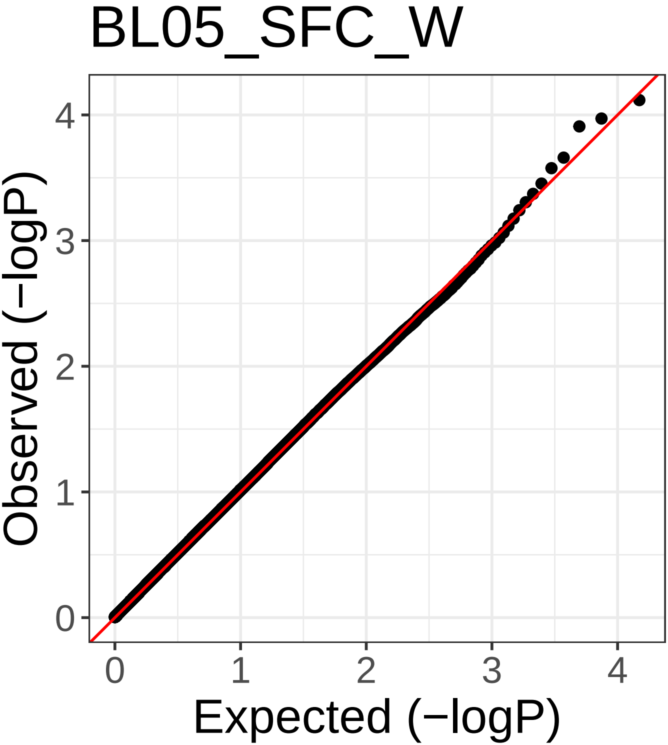
BL05_SFC_W
Observed (−logP)
Expected (−logP)
0
1
2
3
4
0
1
2
3
4

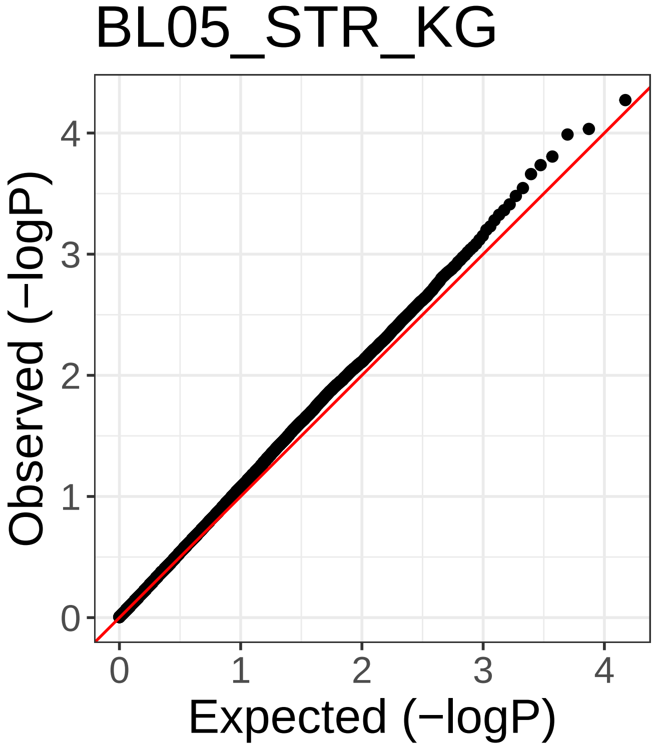
BL05_STR_KG
Observed (−logP)
Expected (−logP)
0
1
2
3
4
0
1
2
3
4

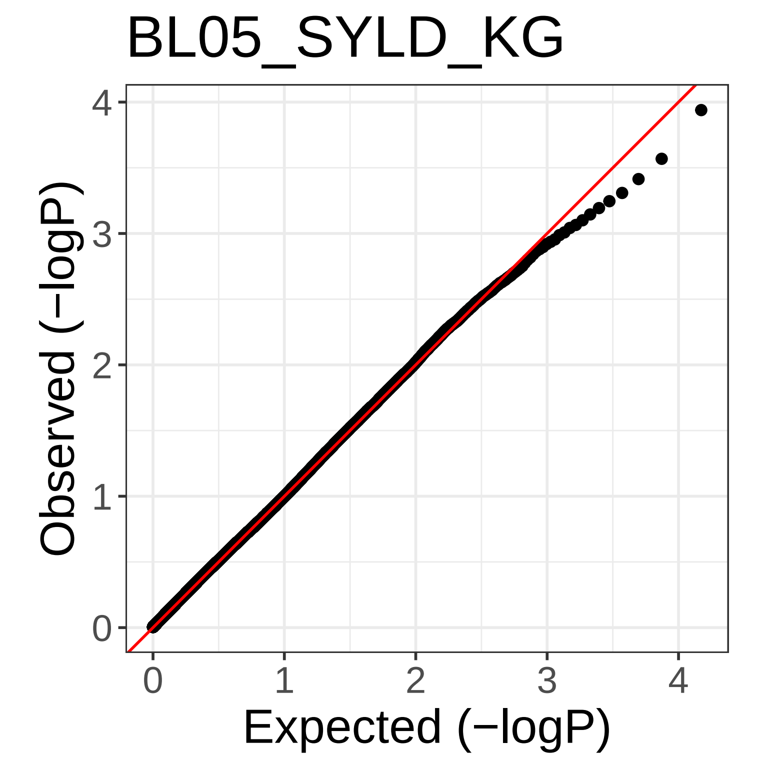
BL05_SYLD_KG
Observed (−logP)
Expected (−logP)
0
1
2
3
4

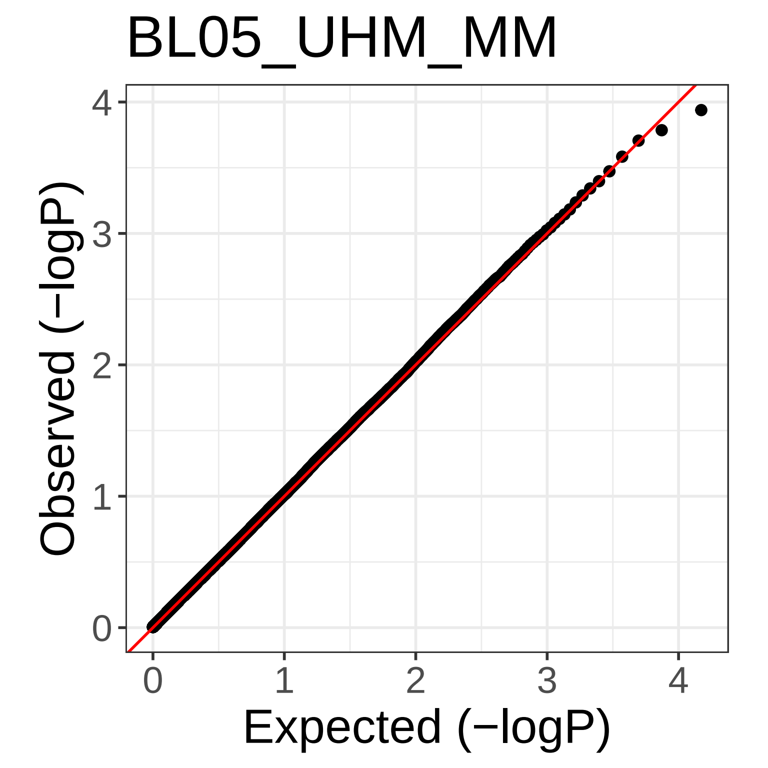

BL05_UHM_MM
Observed (−logP)
Expected (−logP)
0
1
2
3
4
0
1
2
3
4

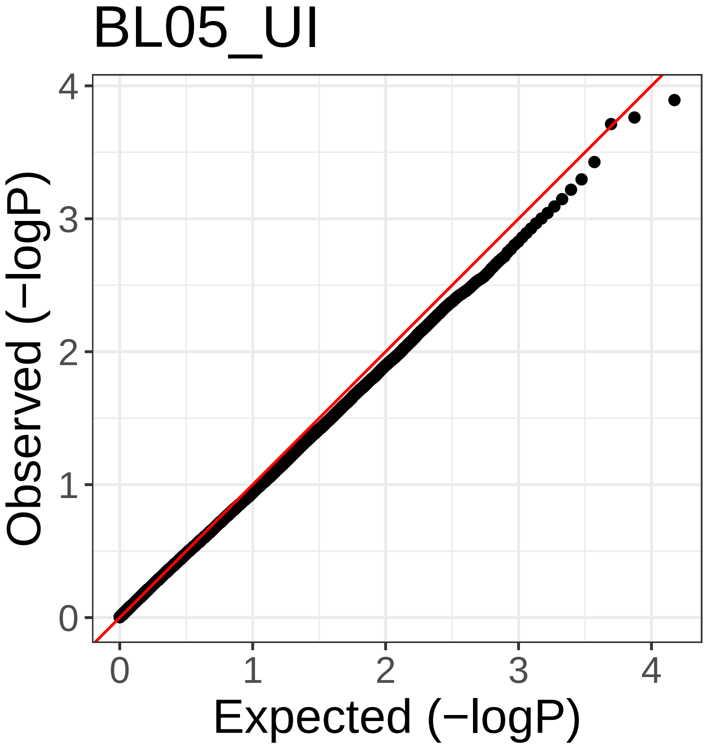

BL05_UI
Observed (−logP)
Expected (−logP)
0
1
2
3
4
0
1
2
3
4

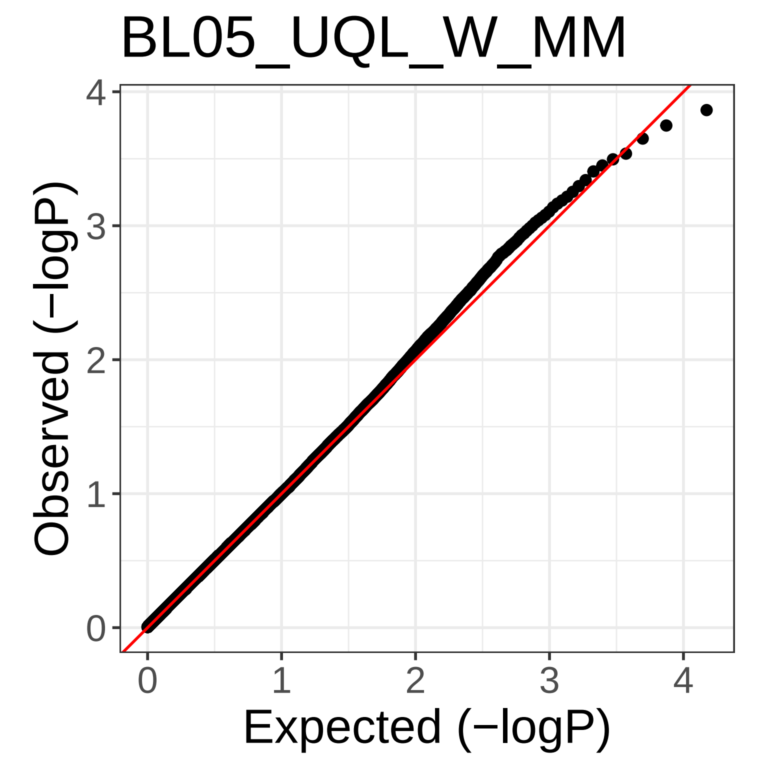
BL05_UQL_W_MM
Observed (−logP)
Expected (−logP)
0
1
2
3
4

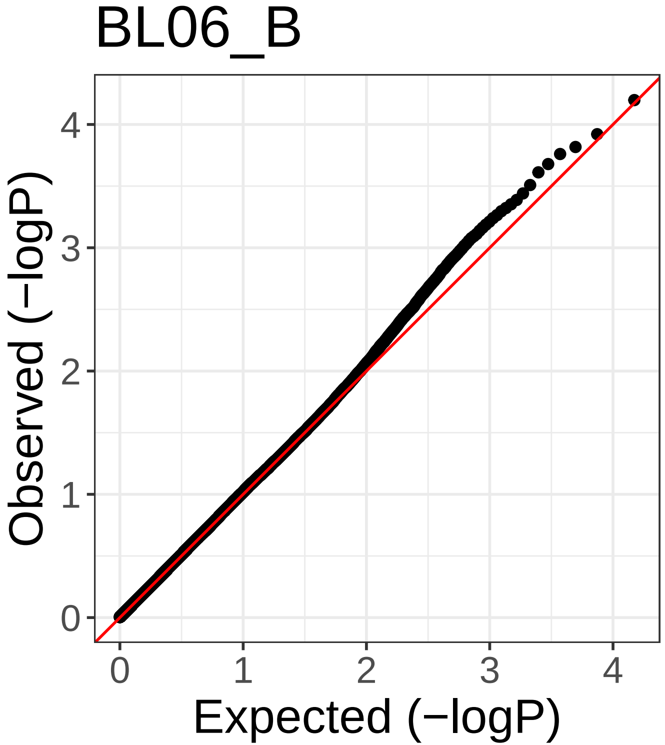

BL06_B
Observed (−logP)
Expected (−logP)
0
1
2
3
4
0
1
2
3
4

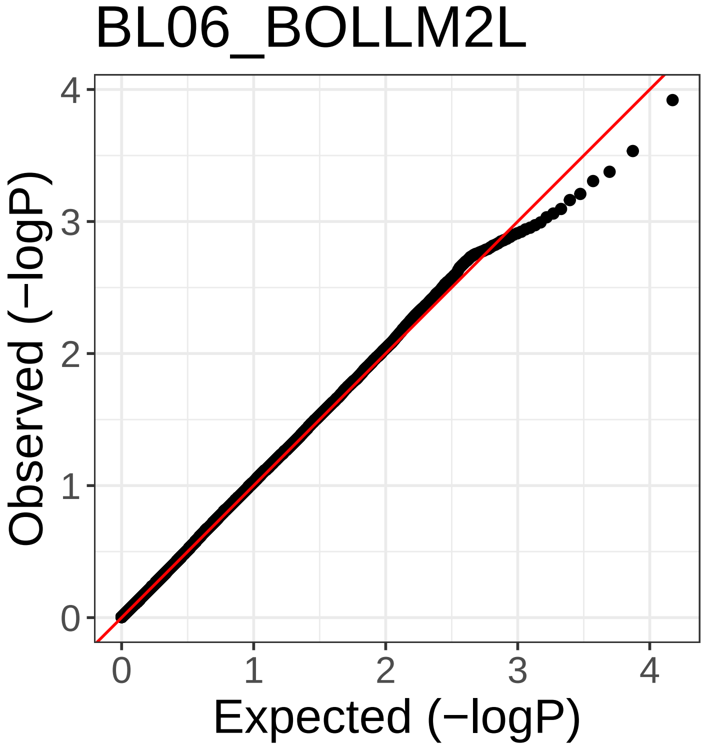

BL06_BOLLM2L
Observed (−logP)
Expected (−logP)
0
1
2
3
4
0
1
2
3
4

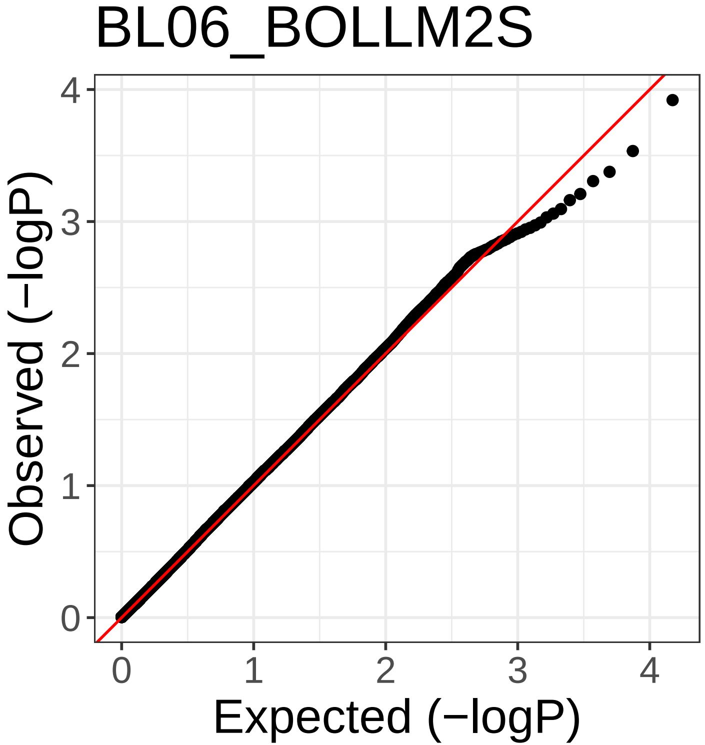

BL06_BOLLM2S
Observed (−logP)
Expected (−logP)
0
1
2
3
4
0
1
2
3
4

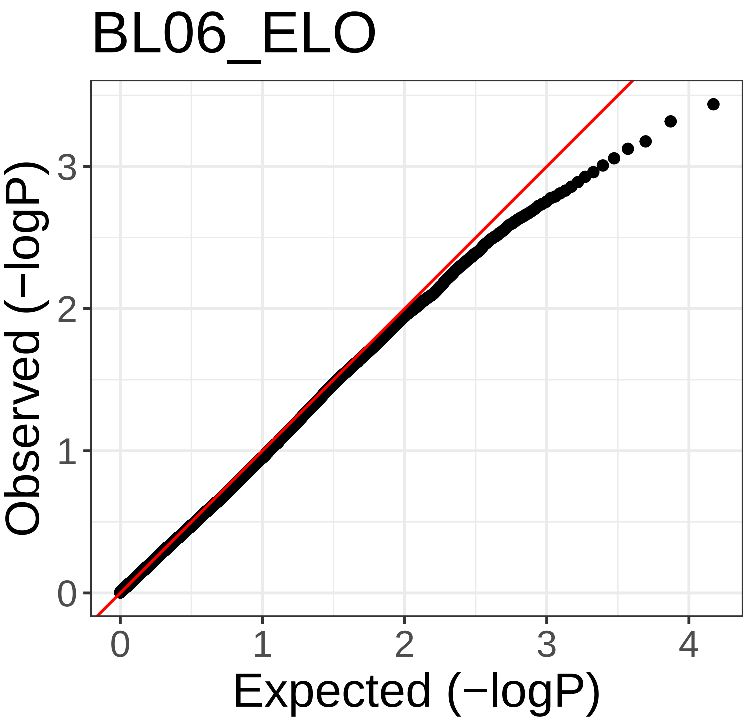

BL06_ELO
Observed (−logP)
Expected (−logP)
0
1
2
3
4

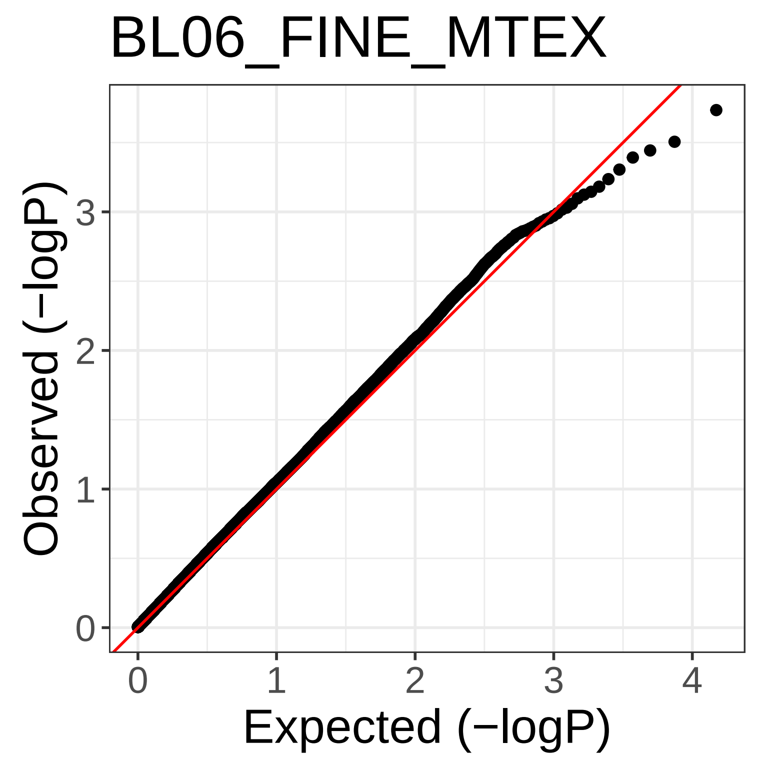

BL06_FINE_MTEX
Observed (−logP)
Expected (−logP)
0
1
2
3
4

BL06_GBOLLSD

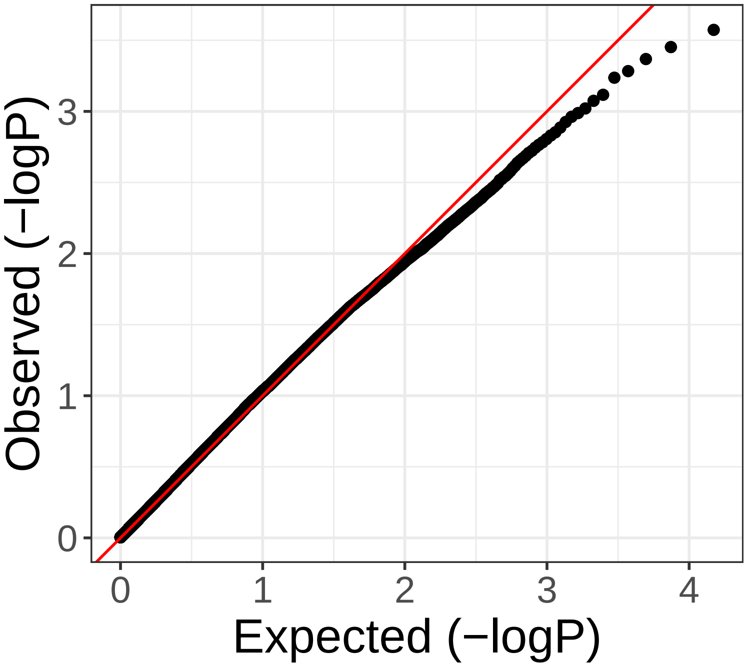

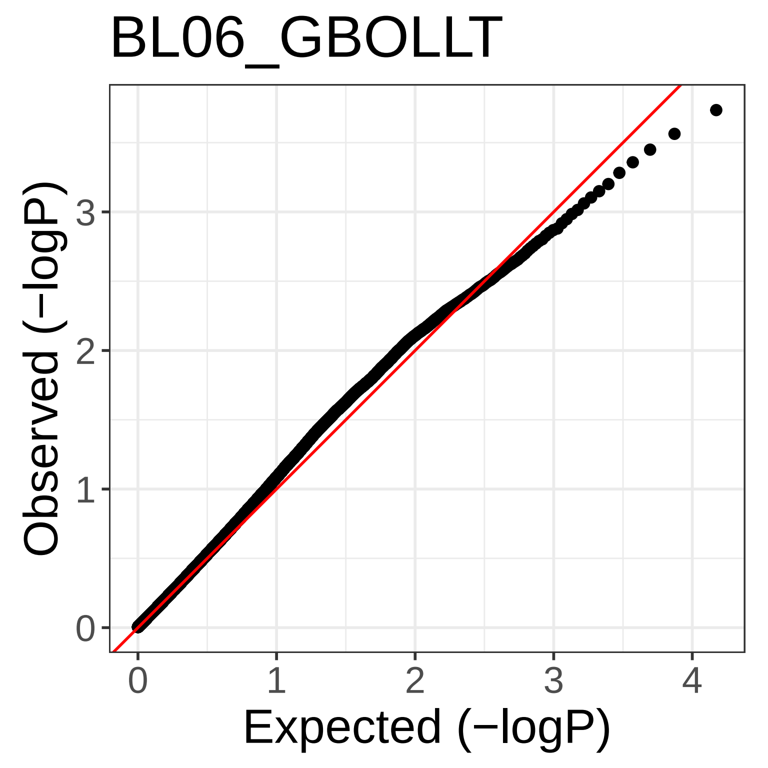

BL06_GBOLLT
Observed (−logP)
Expected (−logP)
0
1
2
3
4

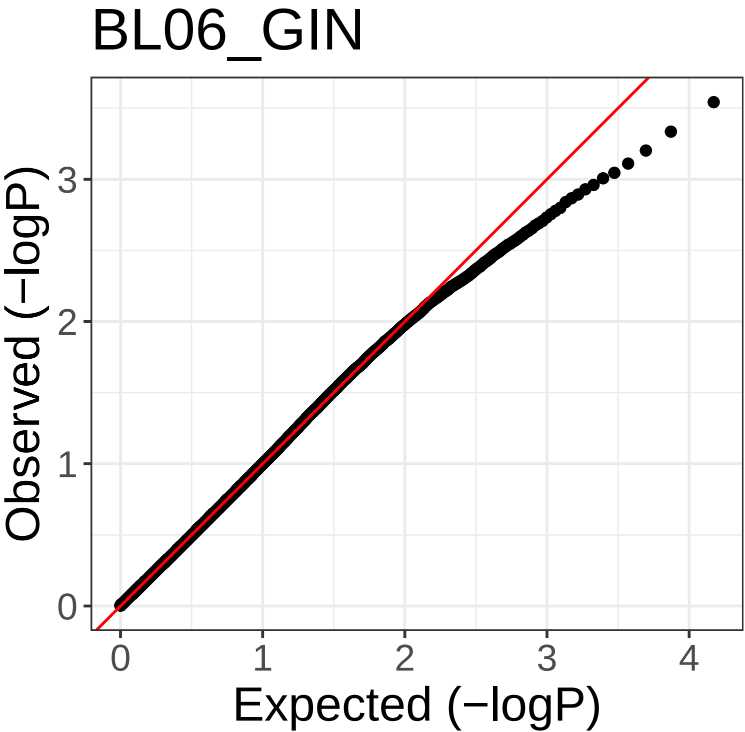
BL06_GIN
Observed (−logP)
Expected (−logP)
0
1
2
3
0
1
2
3
4

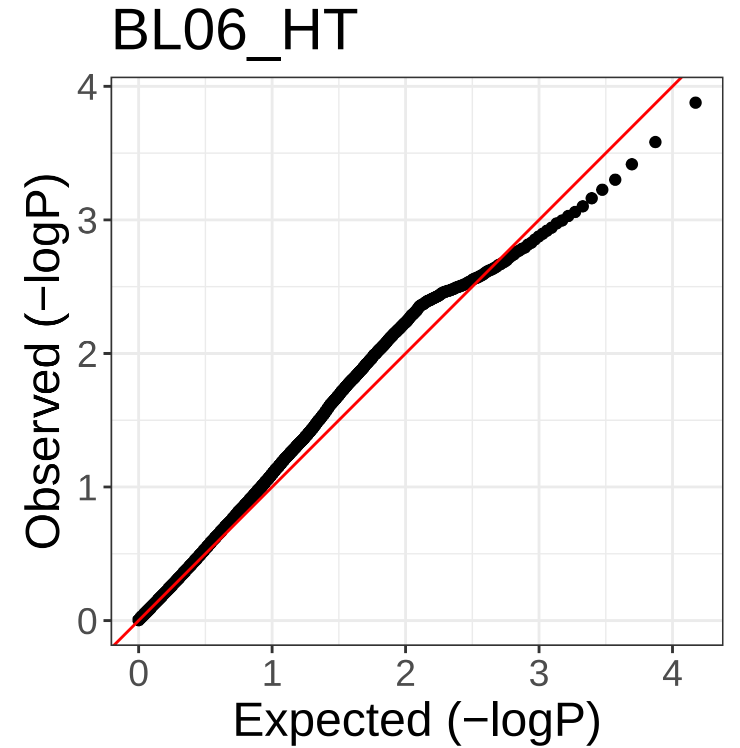
BL06_HT
Observed (−logP)
Expected (−logP)
0
1
2
3
4
0
1
2
3
4

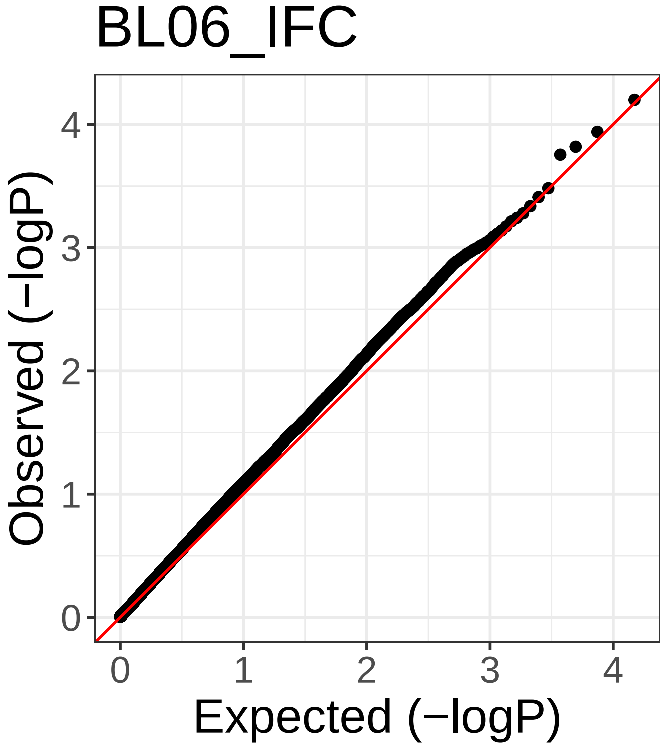
BL06_IFC
Observed (−logP)
Expected (−logP)
0
1
2
3
4
0
1
2
3
4

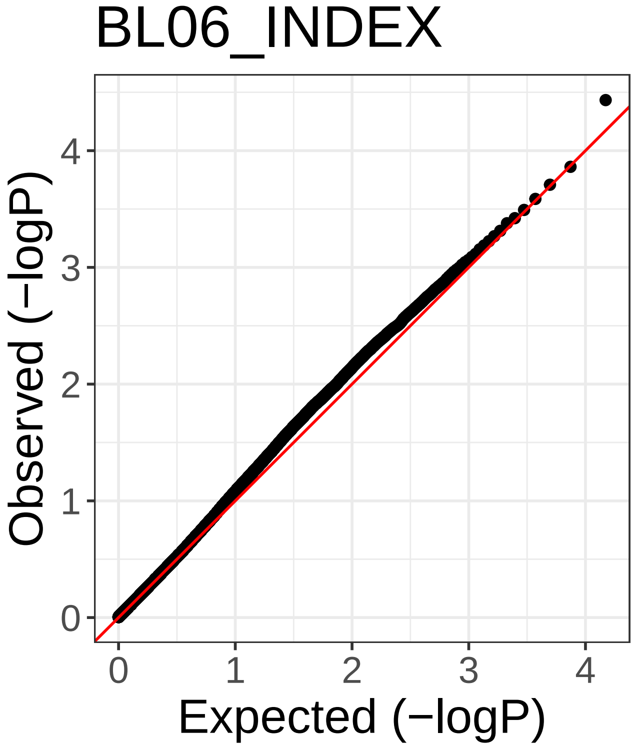

BL06_INDEX
Observed (−logP)
Expected (−logP)
0
1
2
3
4
0
1
2
3
4

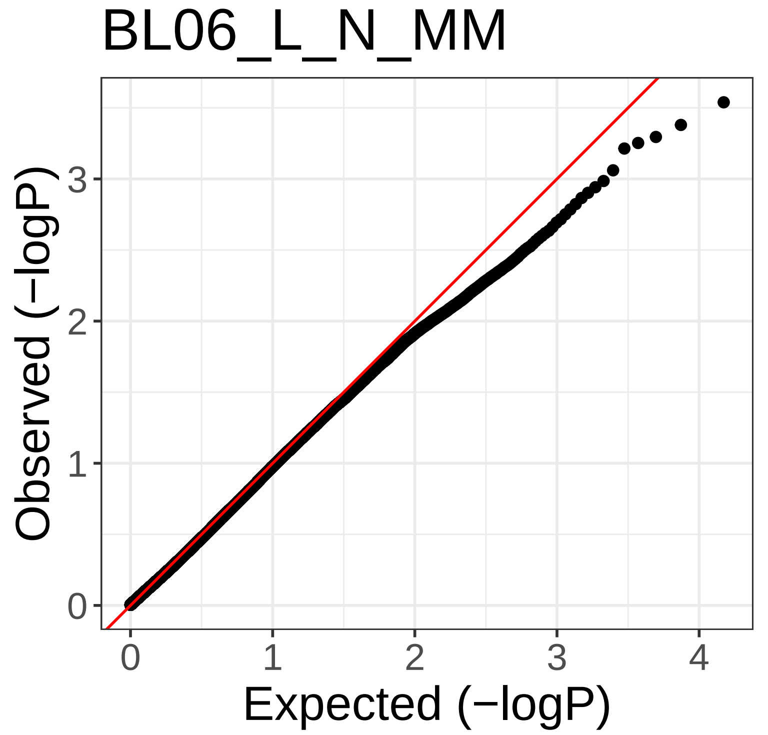
BL06_L_N_MM
Observed (−logP)
Expected (−logP)
0
1
2
3
4

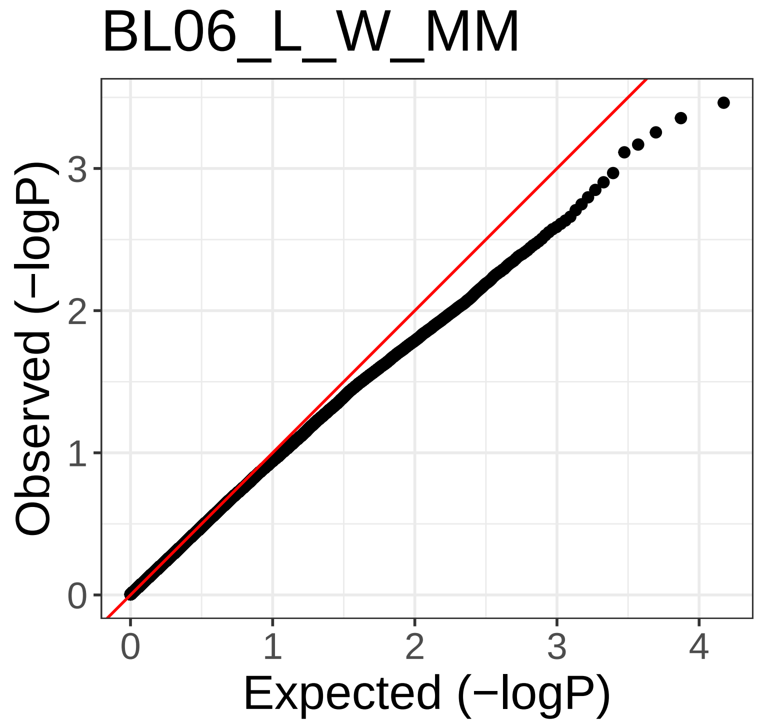
BL06_L_W_MM
Observed (−logP)
Expected (−logP)
0
1
2
3
4

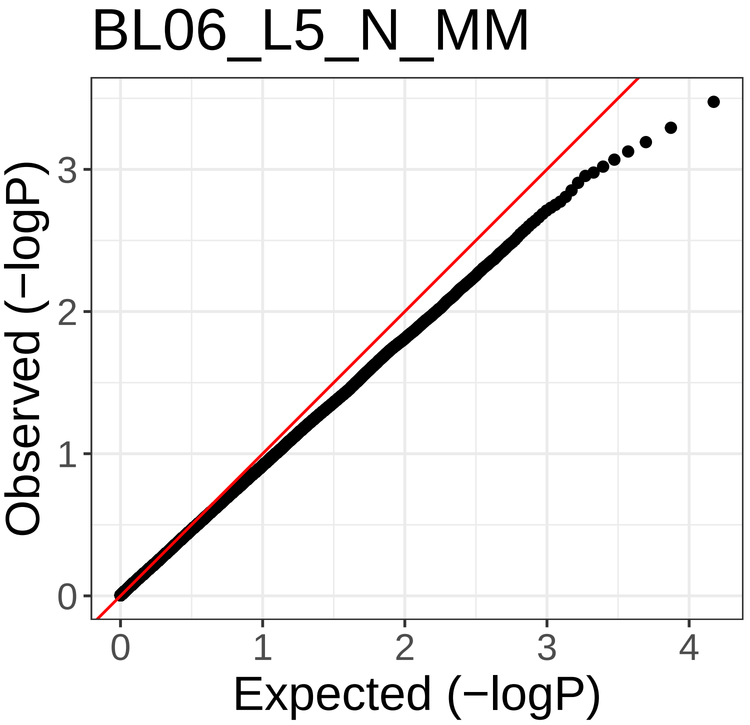
BL06_L5_N_MM
Observed (−logP)
Expected (−logP)
0
1
2
3
4

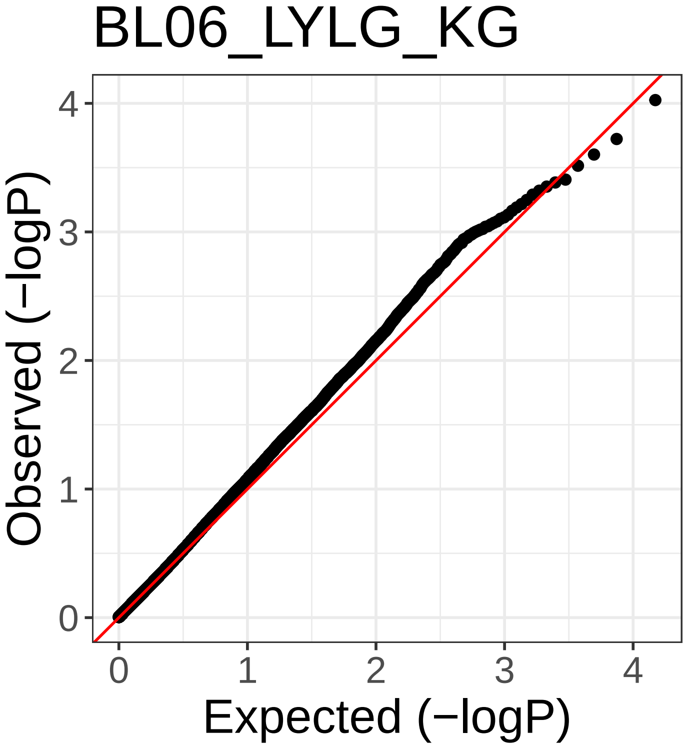
BL06_LYLG_KG
Observed (−logP)
Expected (−logP)
0
1
2
3
4
0
1
2
3
4

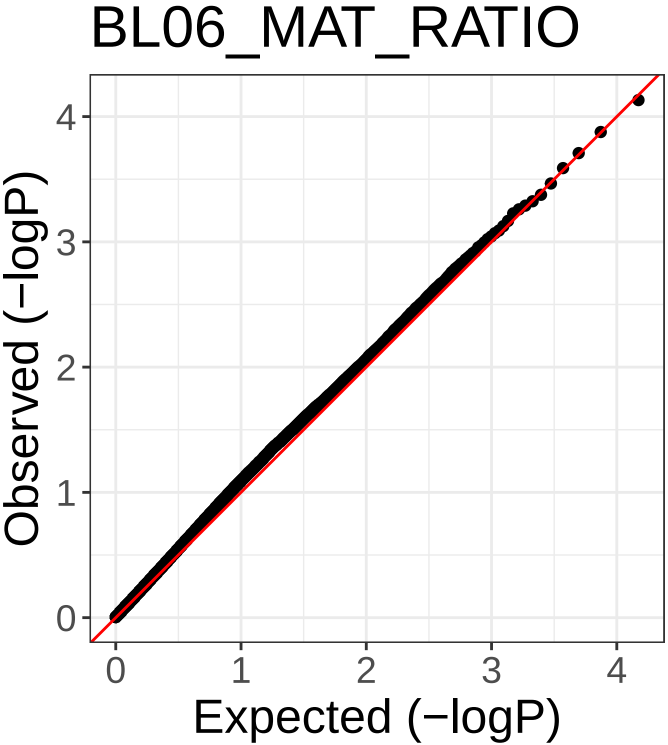

BL06_MAT_RATIO
Observed (−logP)
Expected (−logP)
0
1
2
3
4

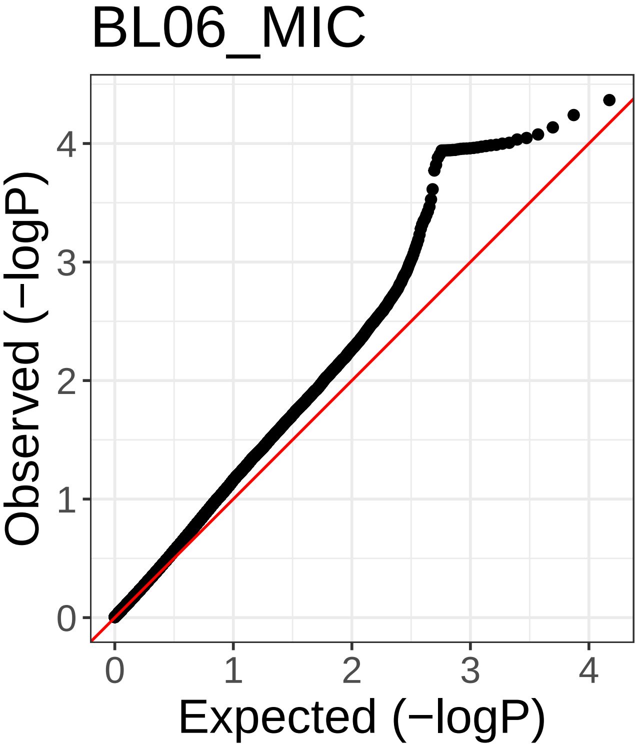
BL06_MIC
Observed (−logP)
Expected (−logP)
0
1
2
3
4
0
1
2
3
4

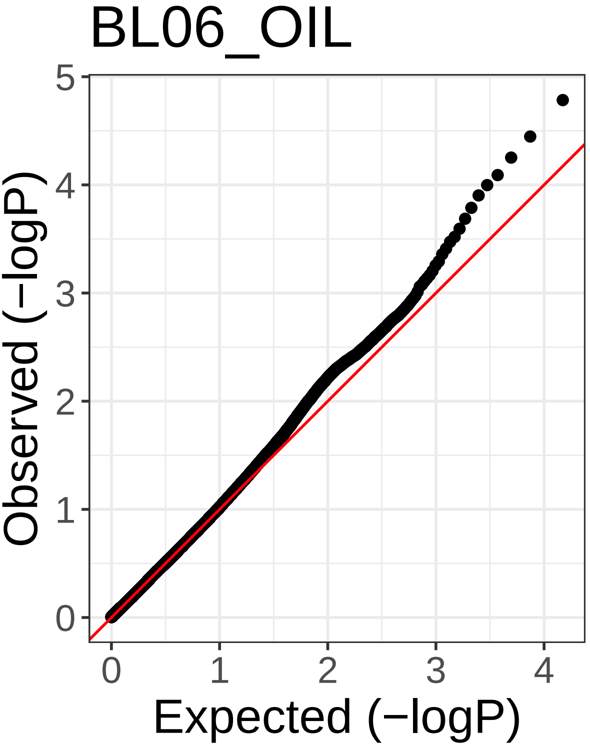
BL06_OIL
Observed (−logP)
Expected (−logP)
0
1
2
3
4
5
0
1
2
3
4

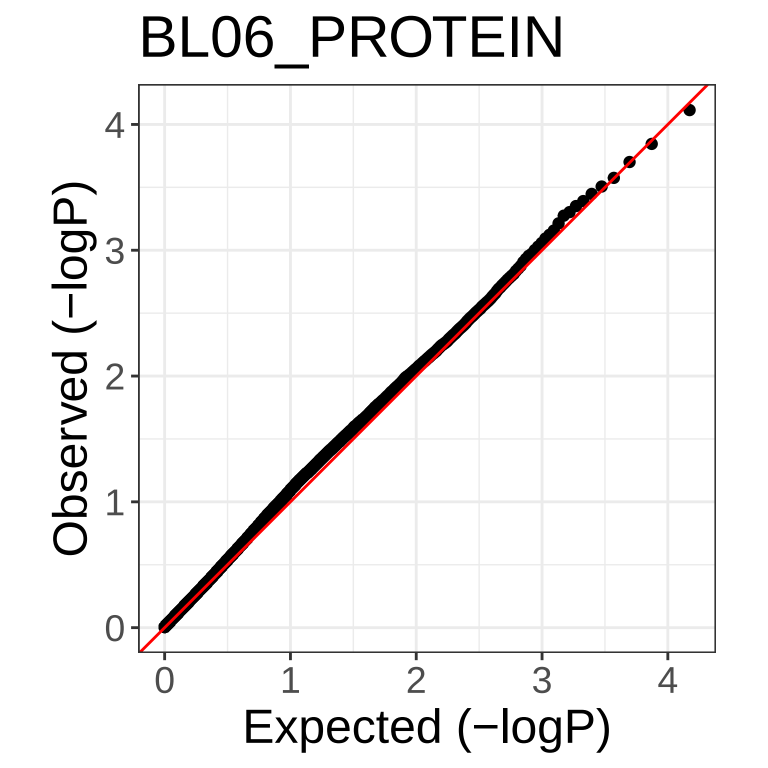
BL06_PROTEIN
Observed (−logP)
Expected (−logP)
0
1
2
3
4
0
1
2
3
4

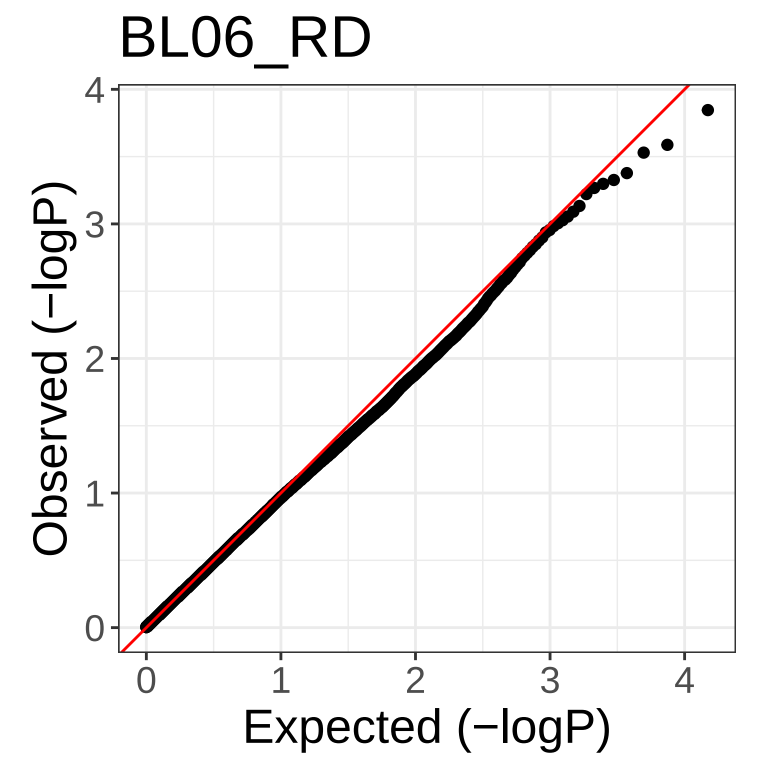
BL06_RD
Observed (−logP)
Expected (−logP)
0
1
2
3
4
0
1
2
3
4

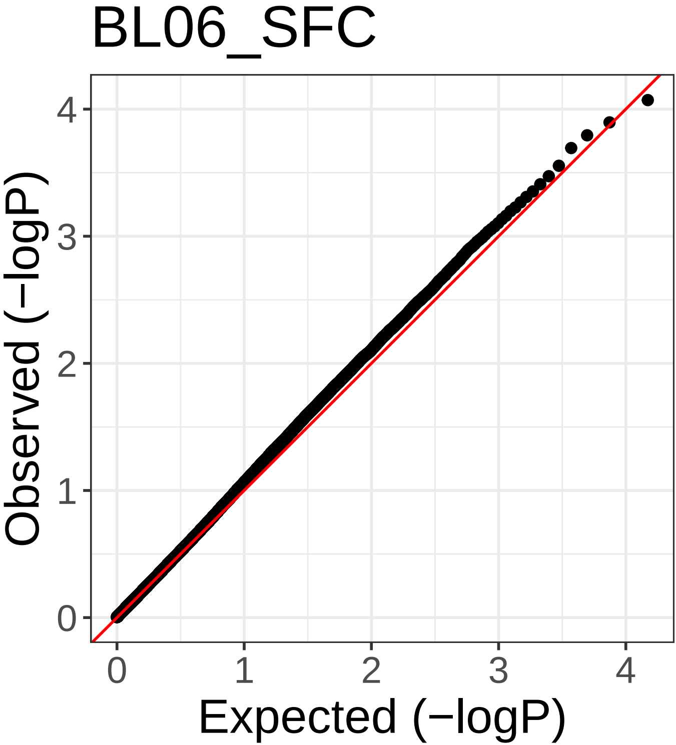
BL06_SFC
Observed (−logP)
Expected (−logP)
0
1
2
3
4
0
1
2
3
4

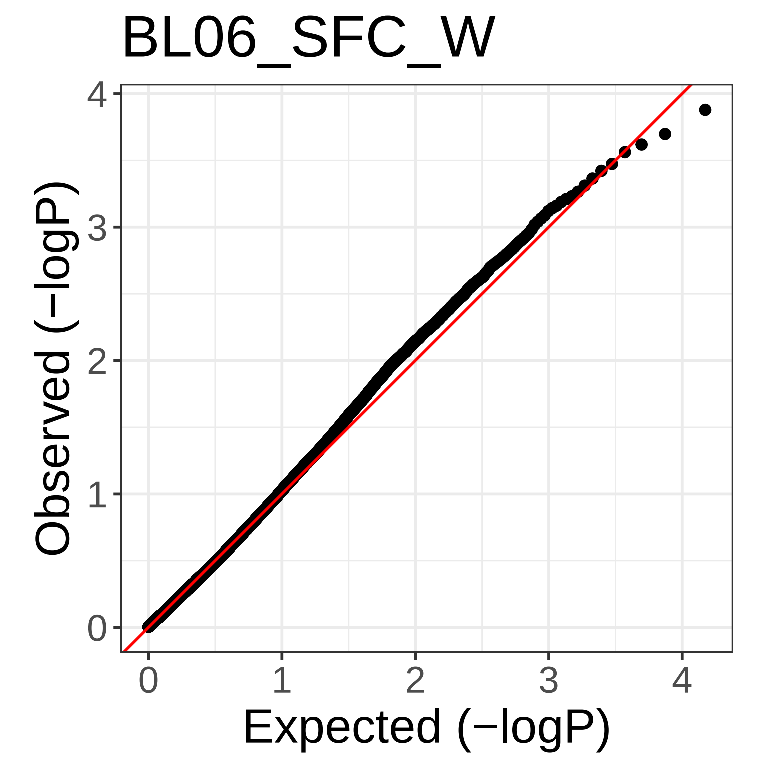
BL06_SFC_W
Observed (−logP)
Expected (−logP)
0
1
2
3
4
0
1
2
3
4

BL06_STR_KG

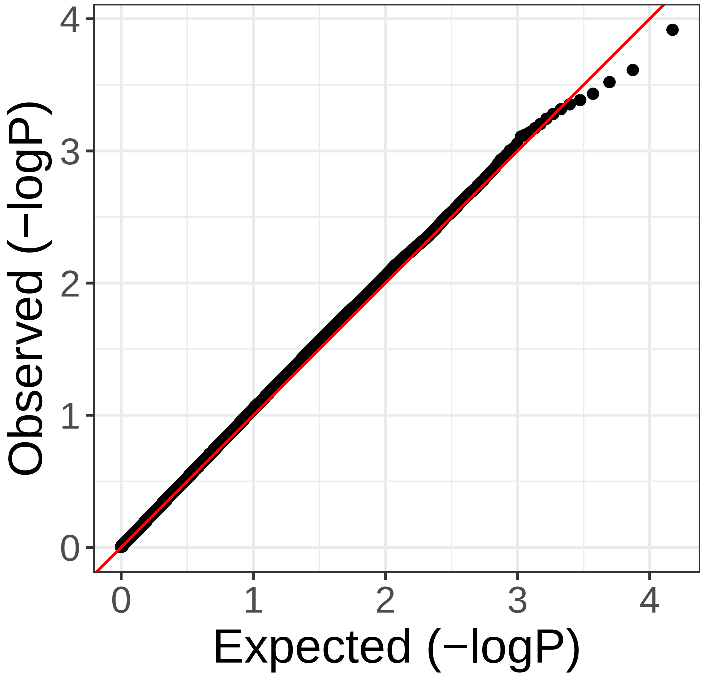

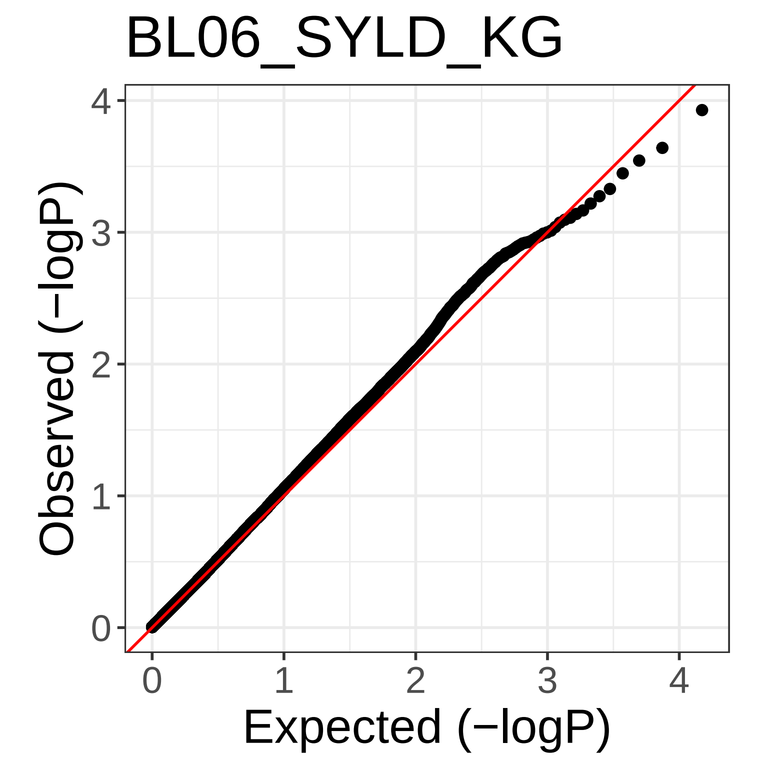
BL06_SYLD_KG
Observed (−logP)
Expected (−logP)
0
1
2
3
4

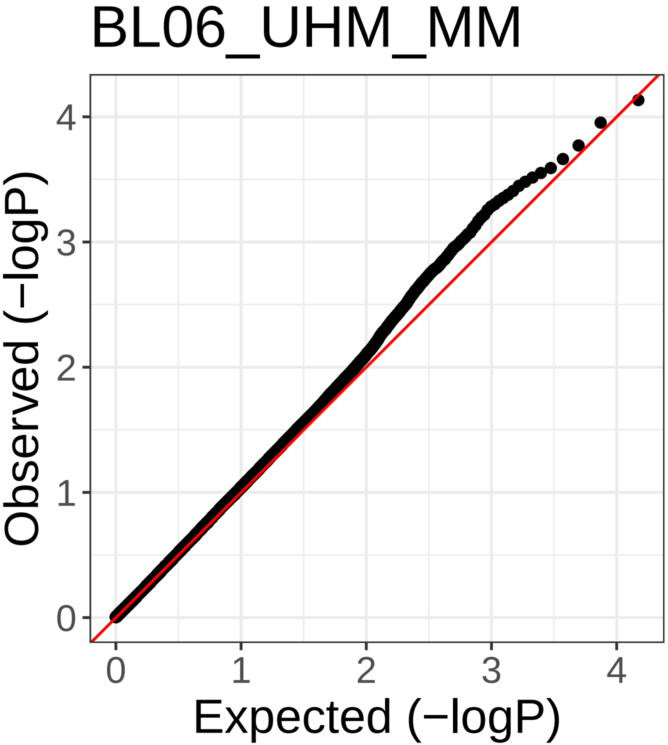
BL06_UHM_MM
Observed (−logP)
Expected (−logP)
0
1
2
3
4
0
1
2
3
4

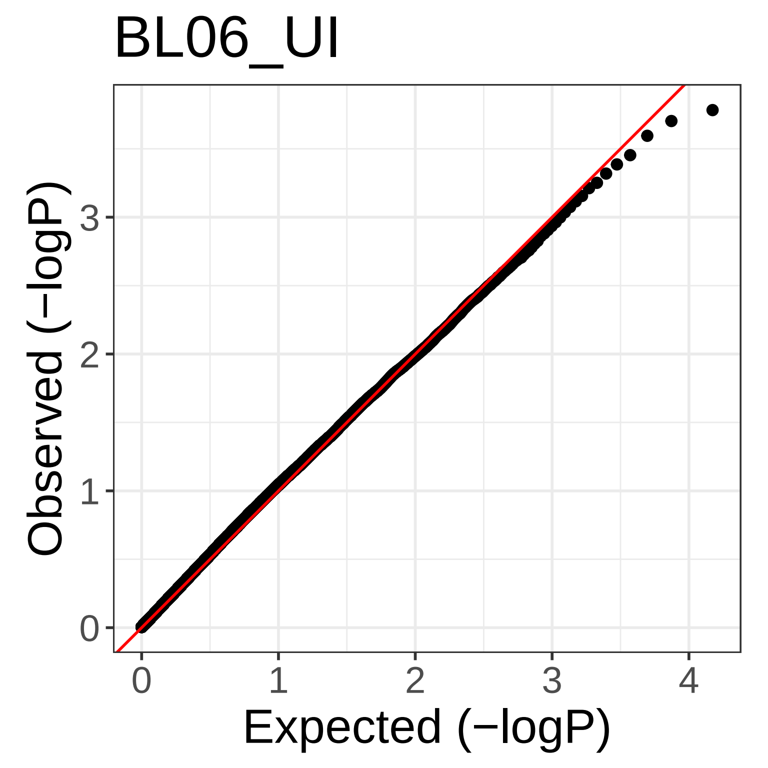

BL06_UI
Observed (−logP)
Expected (−logP)
0
1
2
3
4

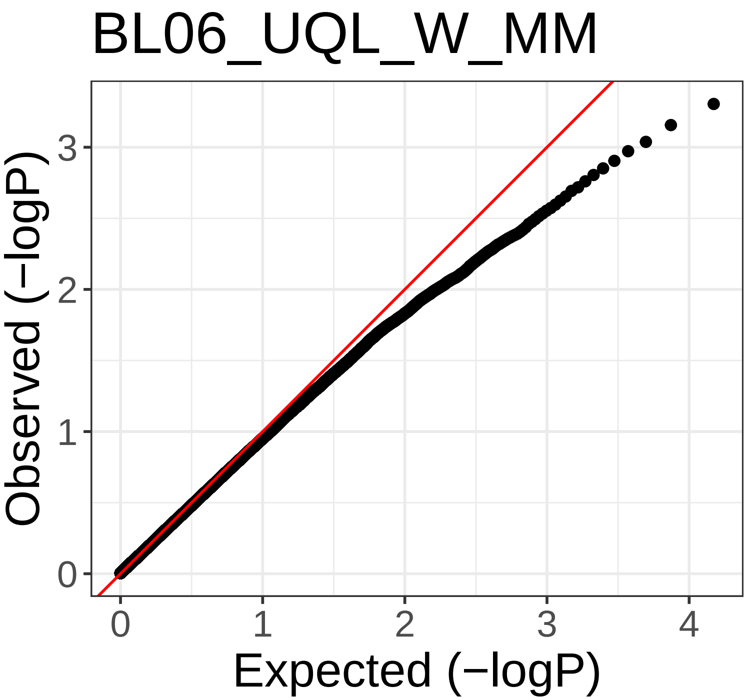

BL06_UQL_W_MM
Observed (−logP)
Expected (−logP)
0
1
2
3
4

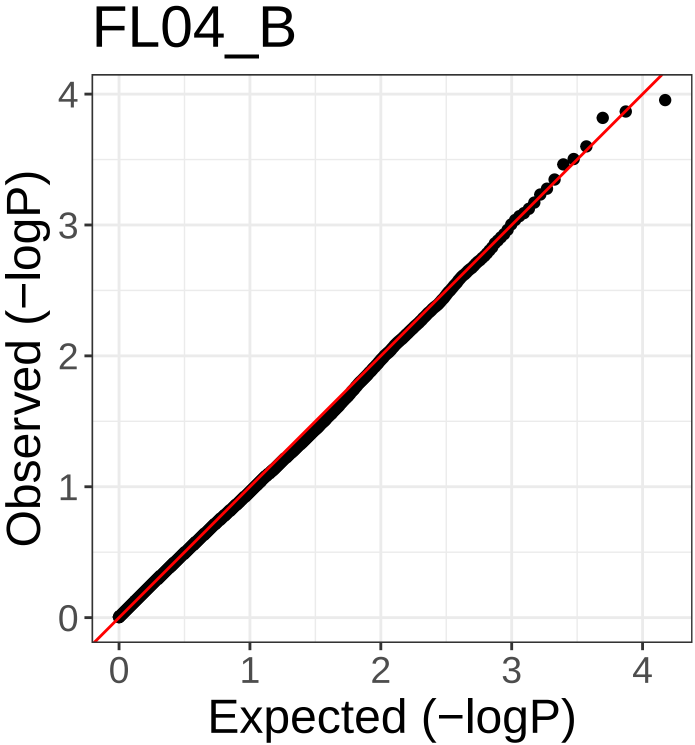

FL04_B
Observed (−logP)
Expected (−logP)
0
1
2
3
4

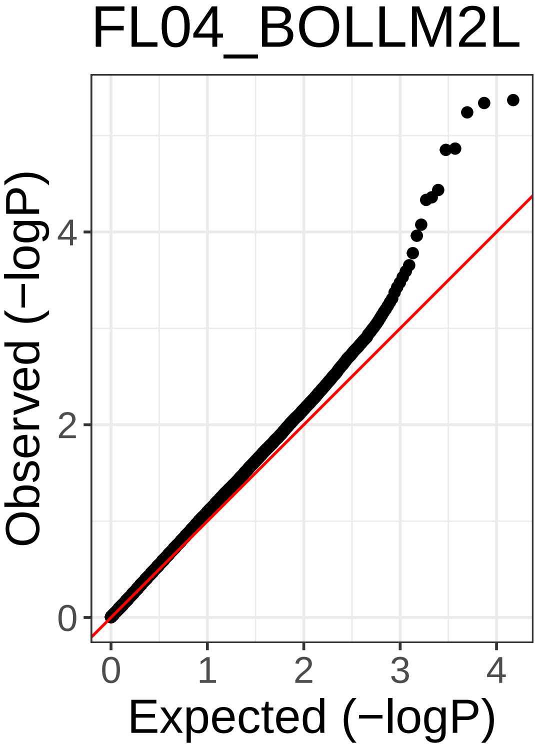
FL04_BOLLM2L
Observed (−logP)
Expected (−logP)
0
2
4
0
1
2
3
4

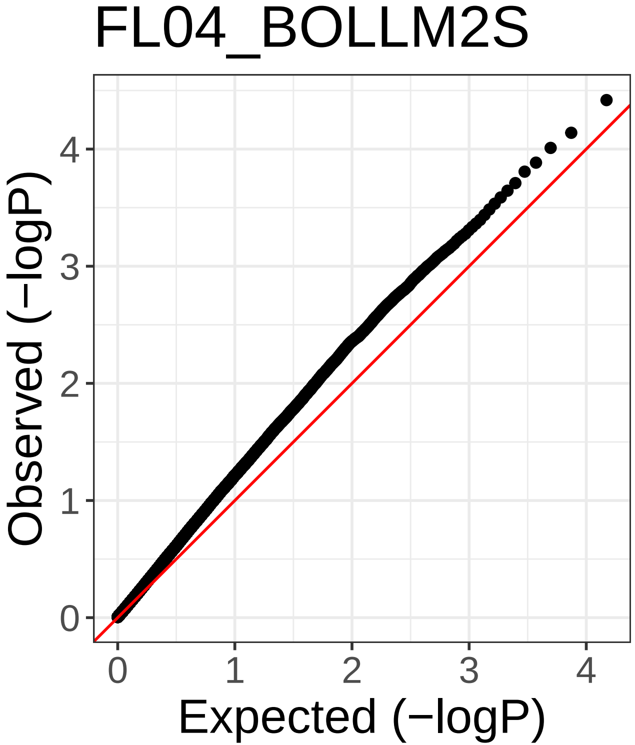
FL04_BOLLM2S
Observed (−logP)
Expected (−logP)
0
1
2
3
4
0
1
2
3
4

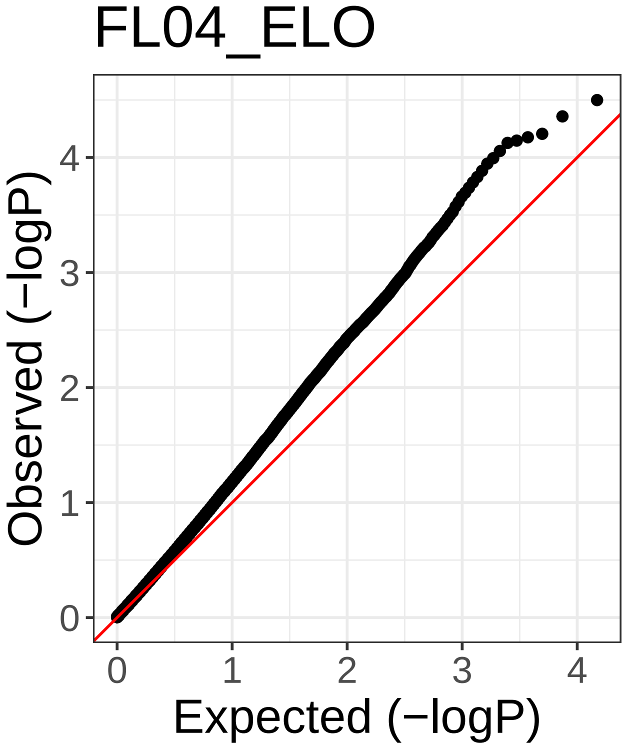
FL04_ELO
Observed (−logP)
0
1
2
3
4
Expected (−logP)
0
1
2
3
4

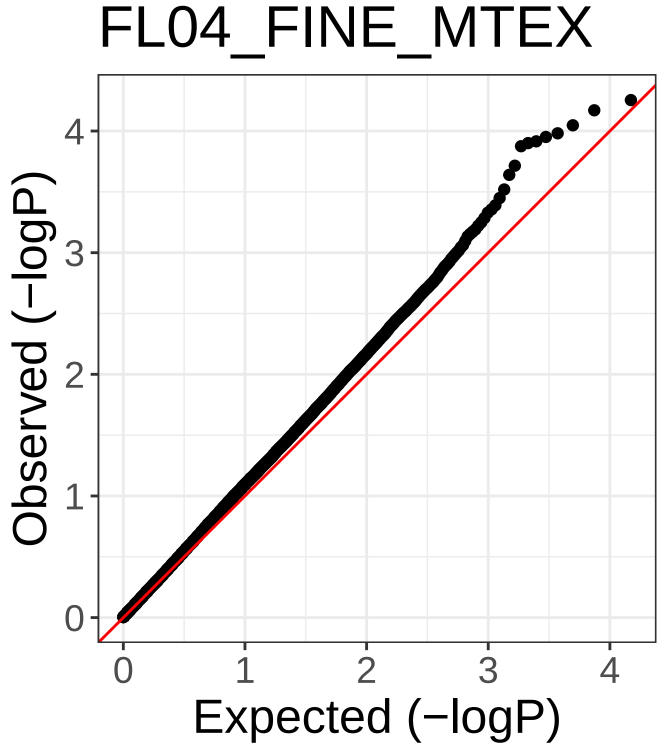
FL04_FINE_MTEX
Observed (−logP)
Expected (−logP)
0
1
2
3
4
0
1
2
3
4

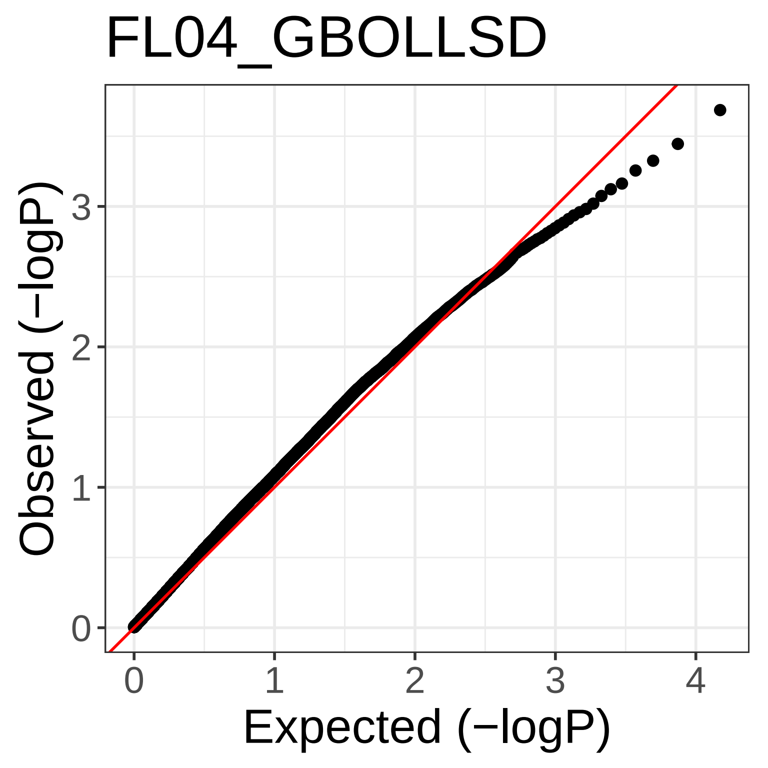
FL04_GBOLLSD
Observed (−logP)
Expected (−logP)
0
1
2
3
0
1
2
3
4

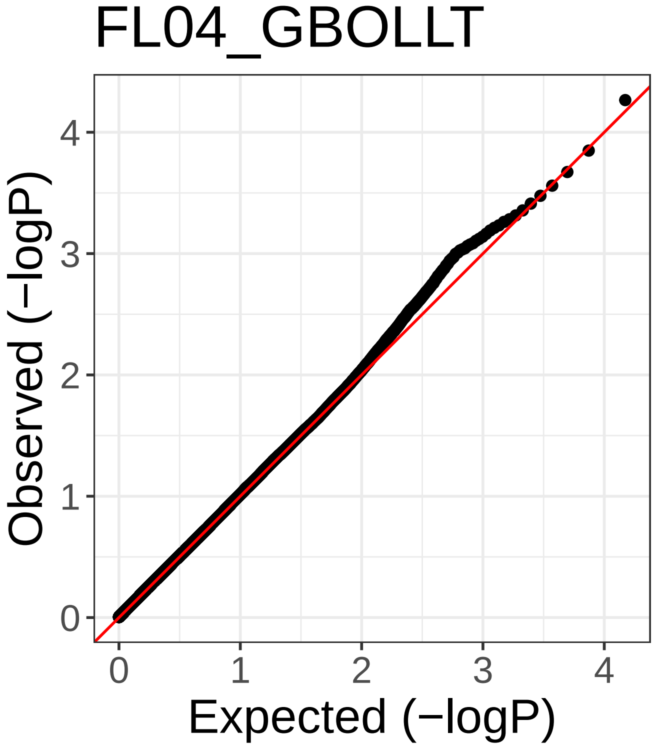
FL04_GBOLLT
Observed (−logP)
Expected (−logP)
0
1
2
3
4
0
1
2
3
4

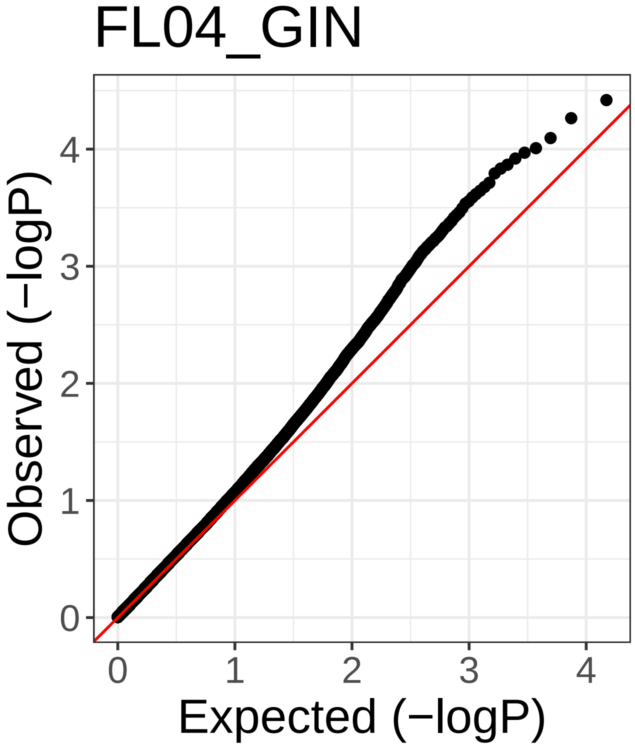
FL04_GIN
Observed (−logP)
Expected (−logP)
0
1
2
3
4
0
1
2
3
4

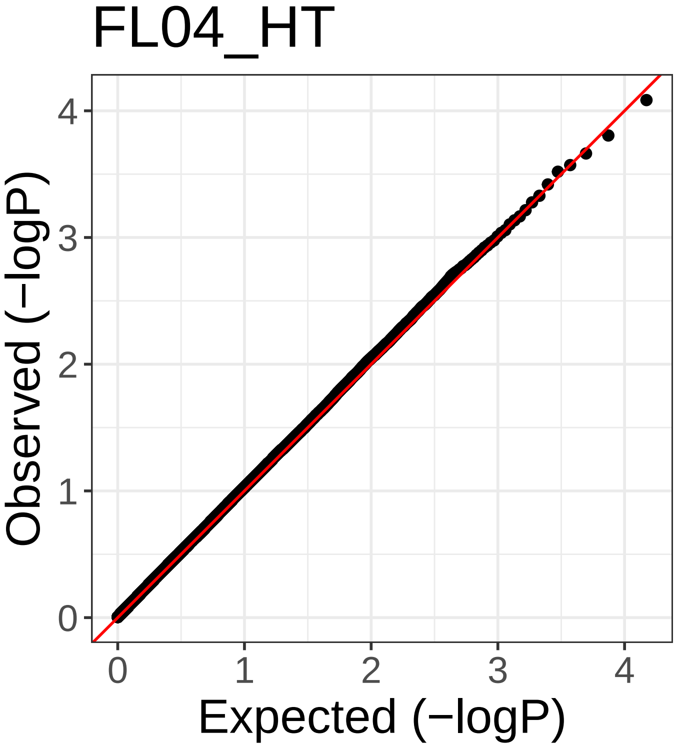
FL04_HT
Observed (−logP)
Expected (−logP)
0
1
2
3
4
0
1
2
3
4

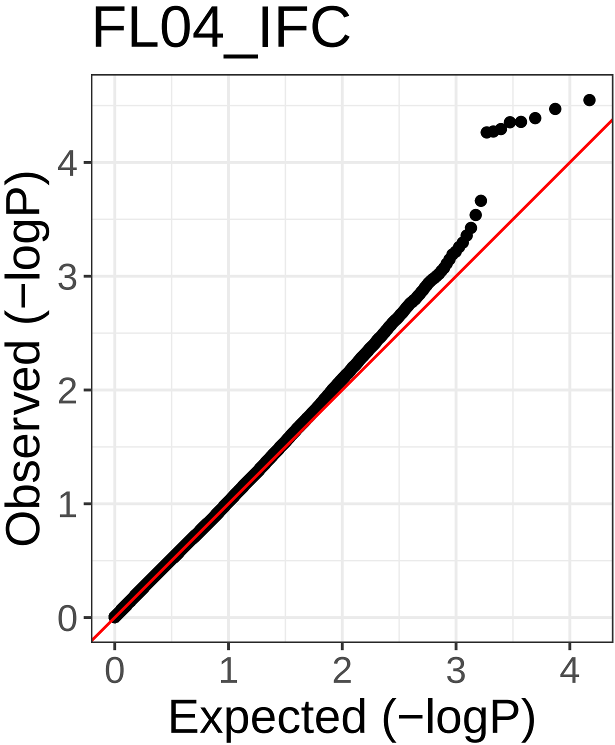

FL04_IFC
Observed (−logP)
Expected (−logP)
0
1
2
3
4
0
1
2
3
4

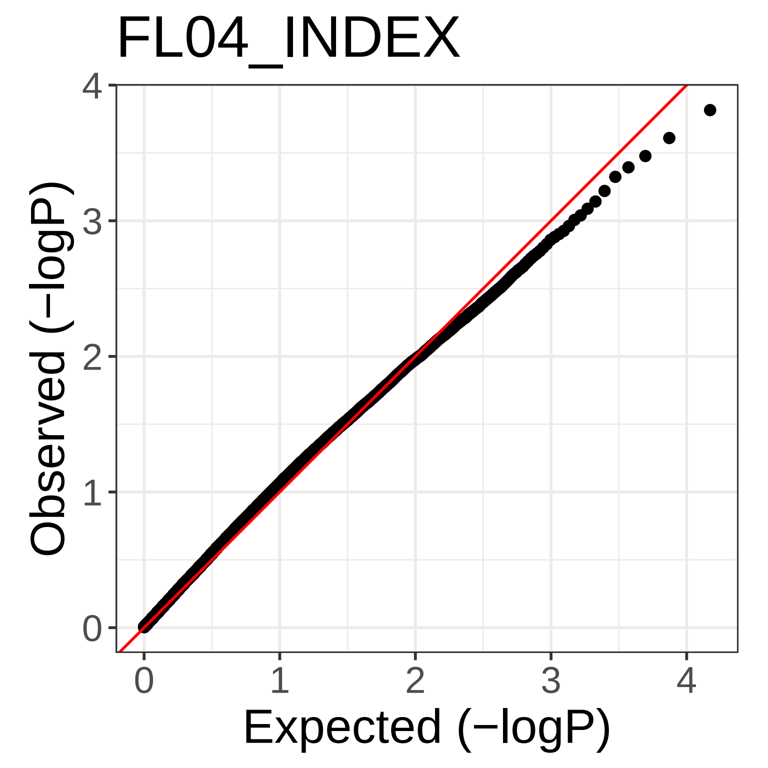
FL04_INDEX
Observed (−logP)
Expected (−logP)
0
1
2
3
4
0
1
2
3
4

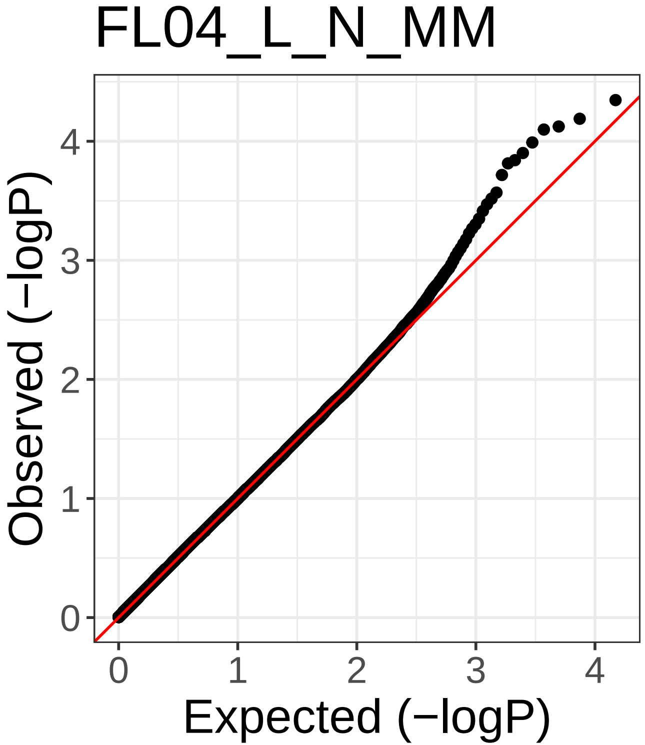
FL04_L_N_MM
Observed (−logP)
Expected (−logP)
0
1
2
3
4
0
1
2
3
4

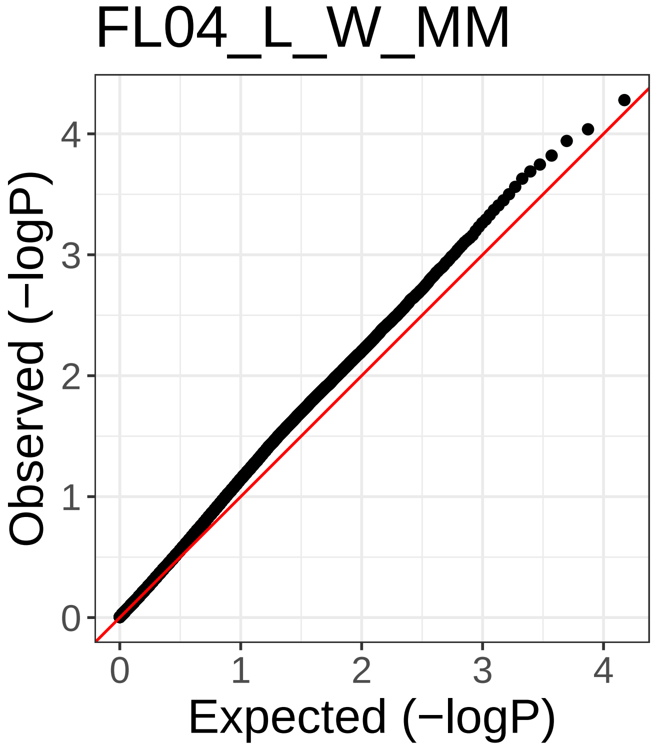
FL04_L_W_MM
Observed (−logP)
Expected (−logP)
0
1
2
3
4
0
1
2
3
4

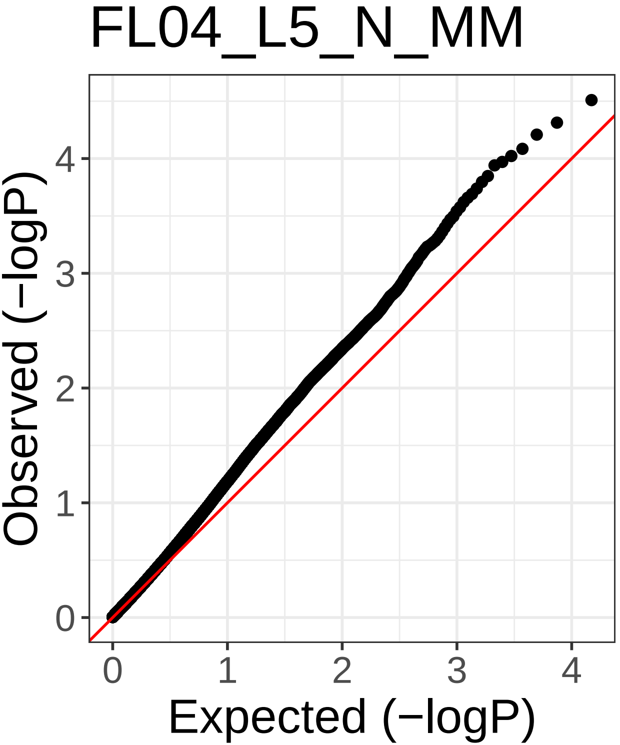
FL04_L5_N_MM
Observed (−logP)
Expected (−logP)
0
1
2
3
4

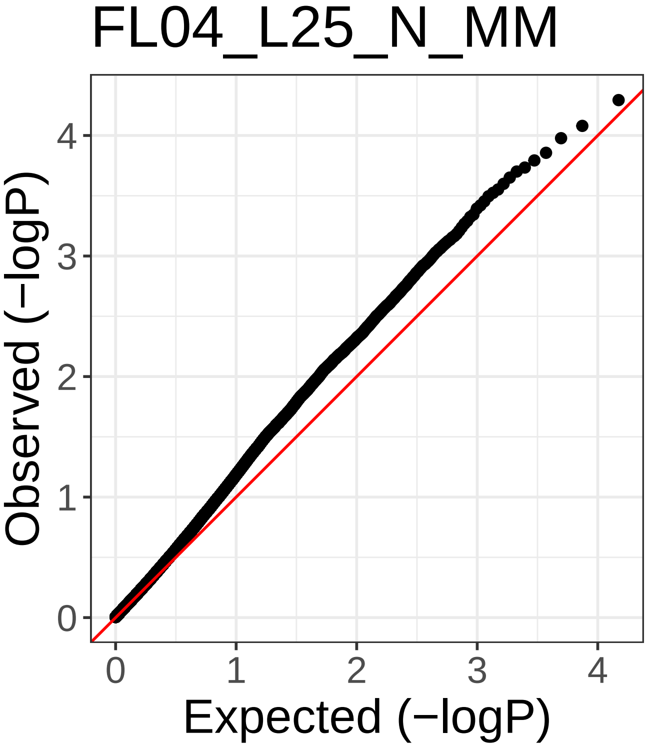
FL04_L25_N_MM
Observed (−logP)
Expected (−logP)
0
1
2
3
4

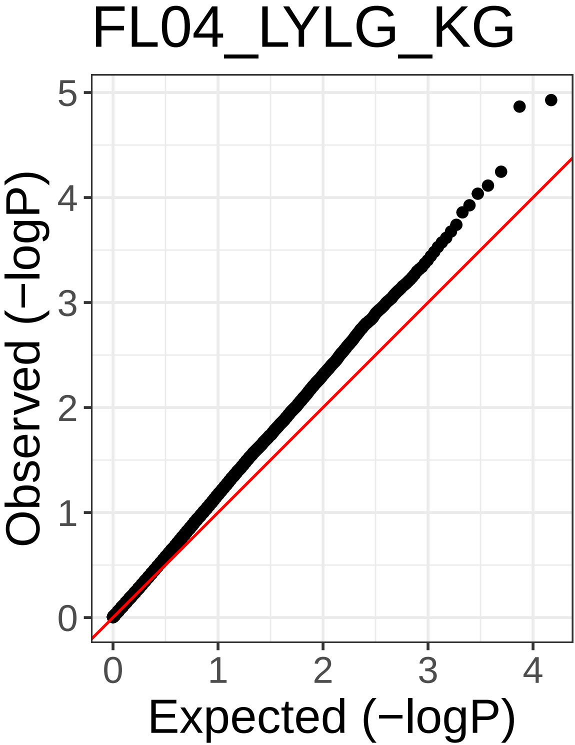
FL04_LYLG_KG
Observed (−logP)
Expected (−logP)
0
1
2
3
4
5

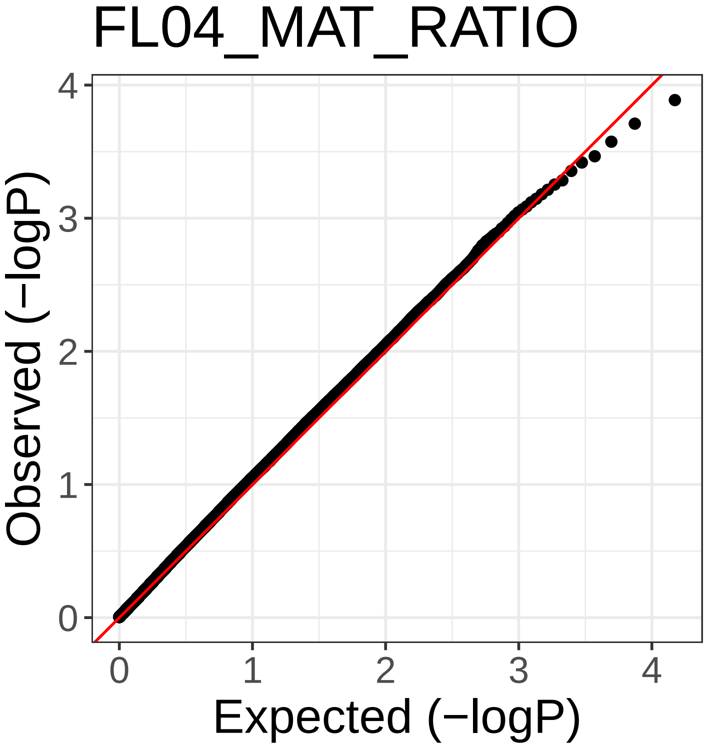

FL04_MAT_RATIO
Observed (−logP)
Expected (−logP)
0
1
2
3
4
0
1
2
3
4

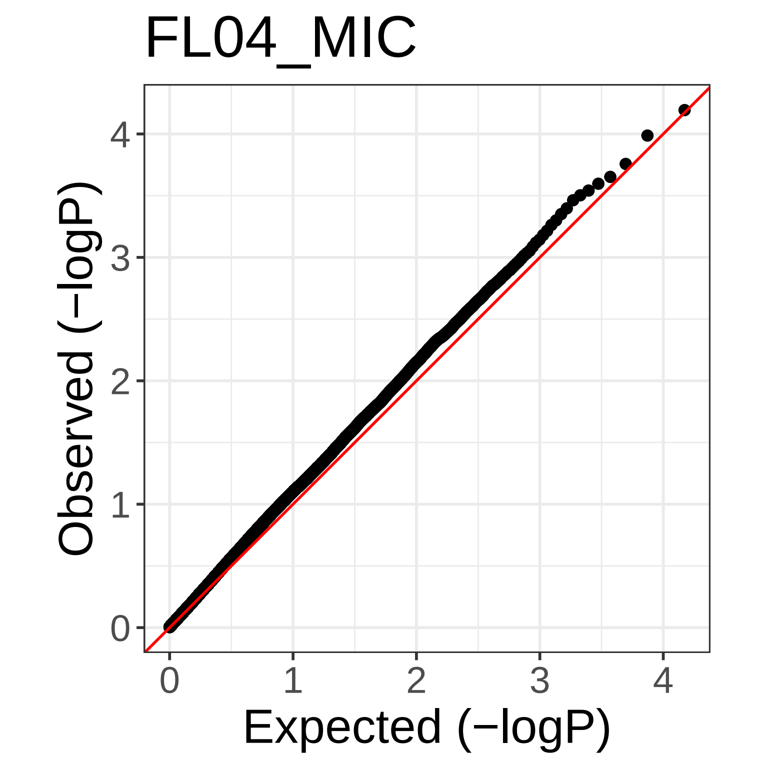
FL04_MIC
Observed (−logP)
Expected (−logP)
0
1
2
3
4

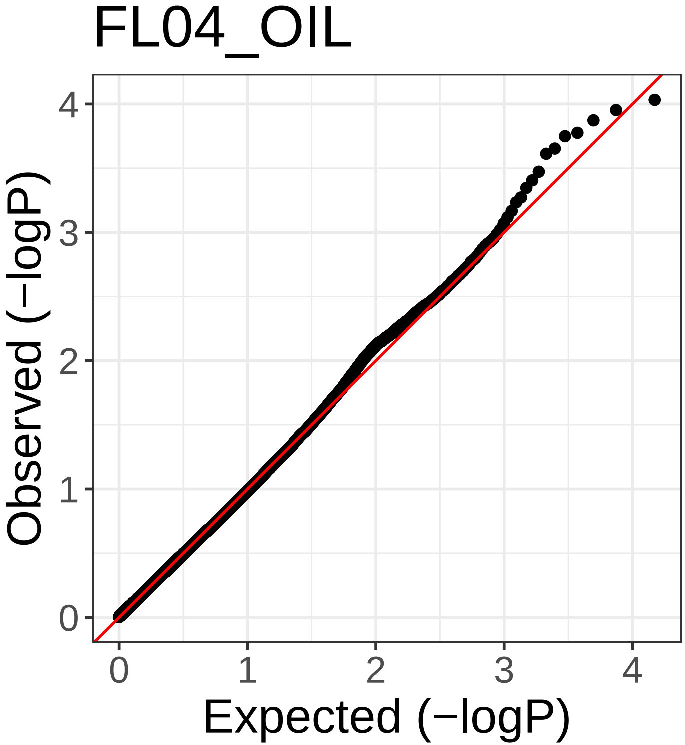
FL04_OIL
Observed (−logP)
Expected (−logP)
0
1
2
3
4
0
1
2
3
4

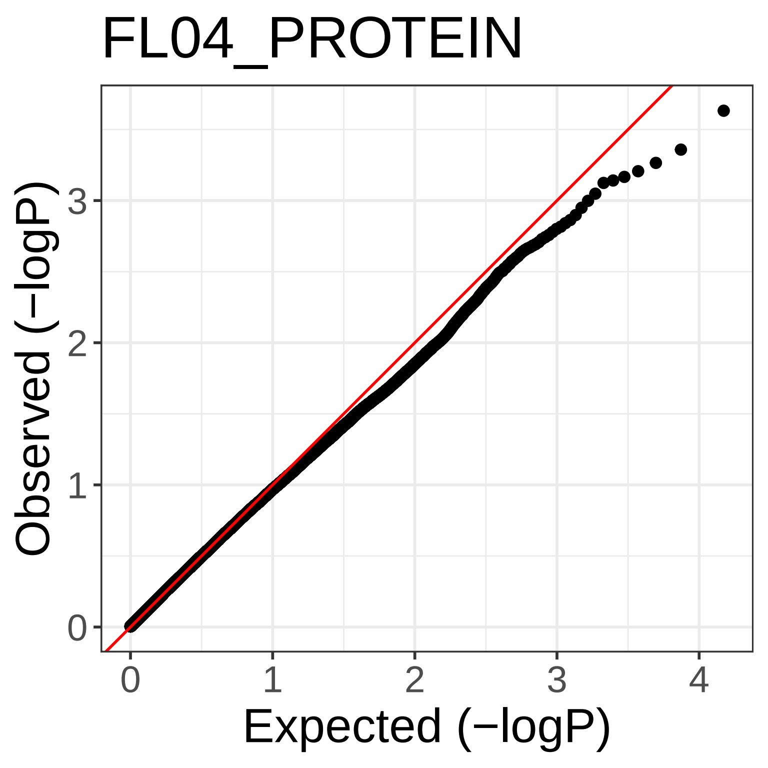
FL04_PROTEIN
Observed (−logP)
Expected (−logP)
0
1
2
3
4

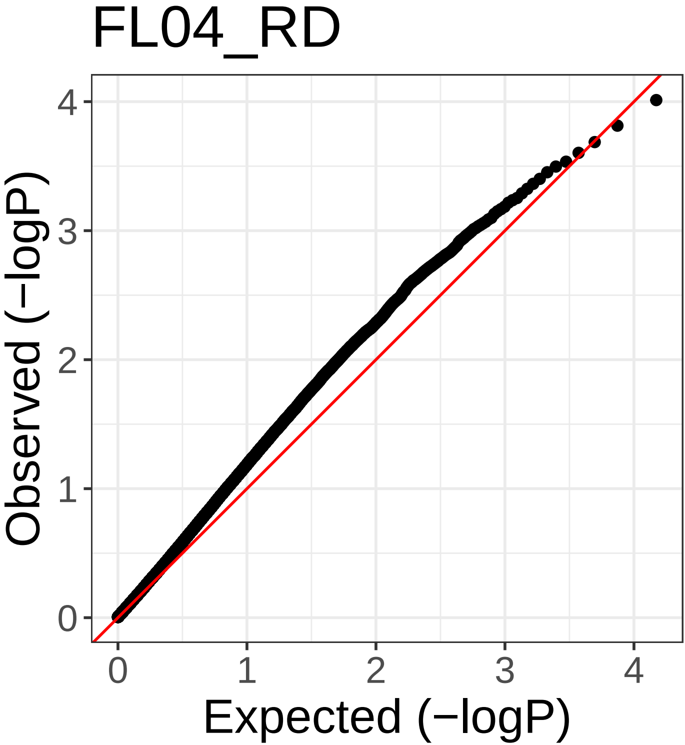

FL04_RD
Observed (−logP)
Expected (−logP)
0
1
2
3
4
0
1
2
3
4

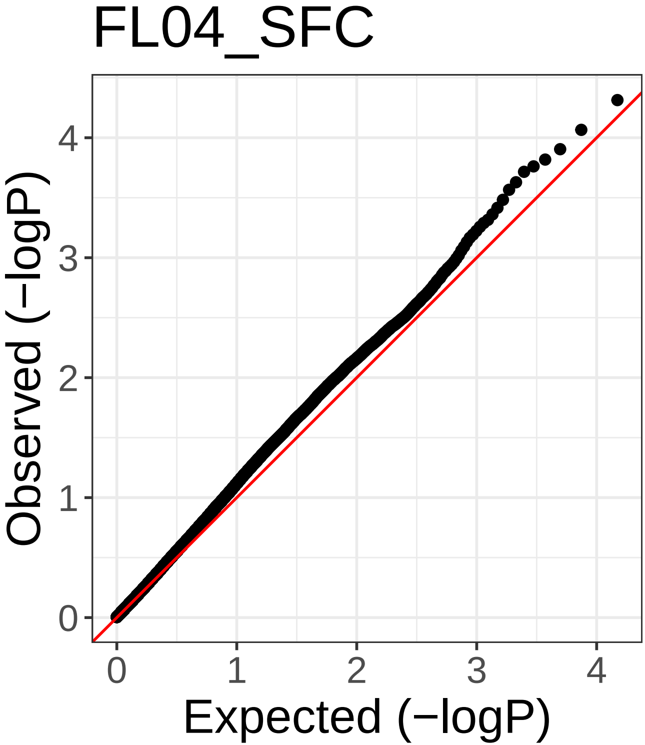

FL04_SFC
Observed (−logP)
Expected (−logP)
0
1
2
3
4
0
1
2
3
4

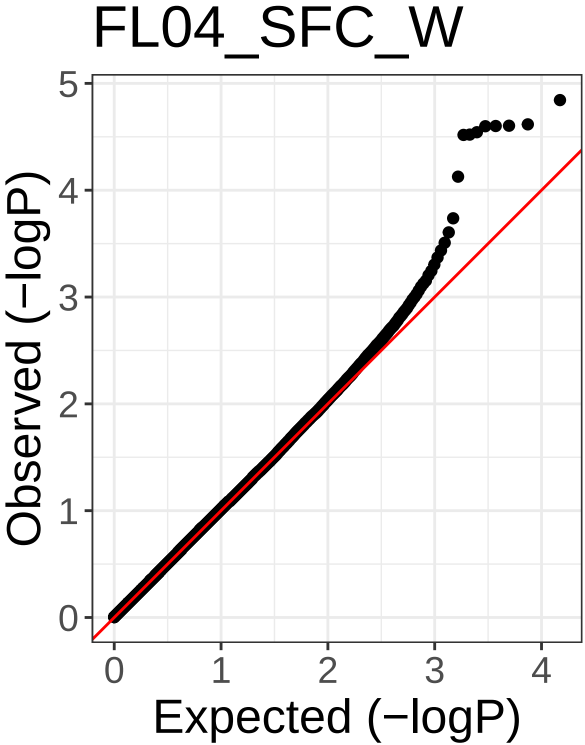
FL04_SFC_W
Observed (−logP)
Expected (−logP)
0
1
2
3
4
5

FL04_STR_KG

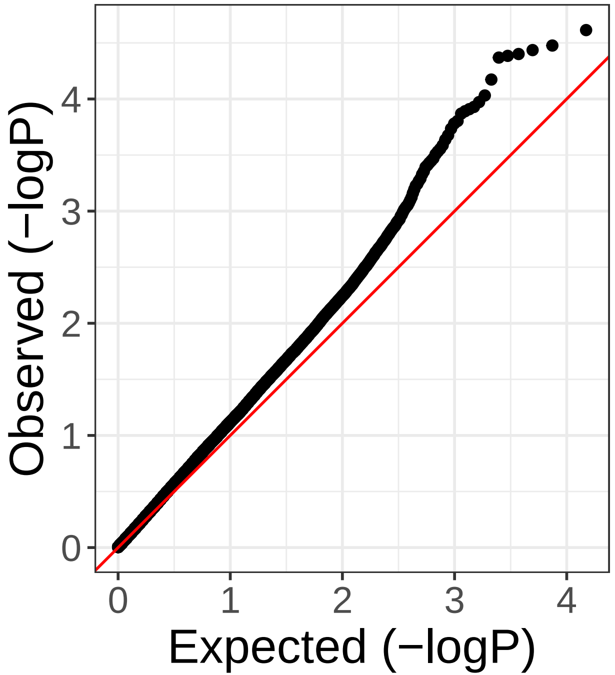

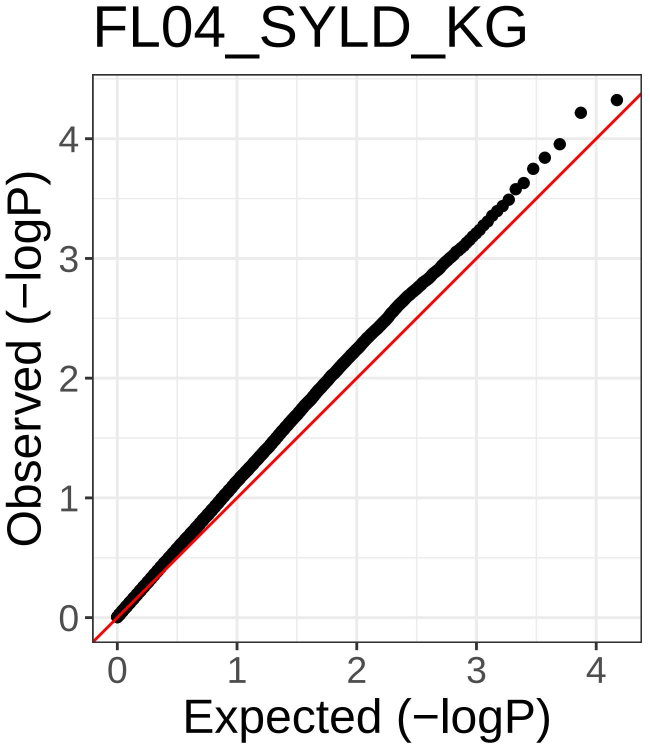
FL04_SYLD_KG
Observed (−logP)
Expected (−logP)
0
1
2
3
4
0
1
2
3
4

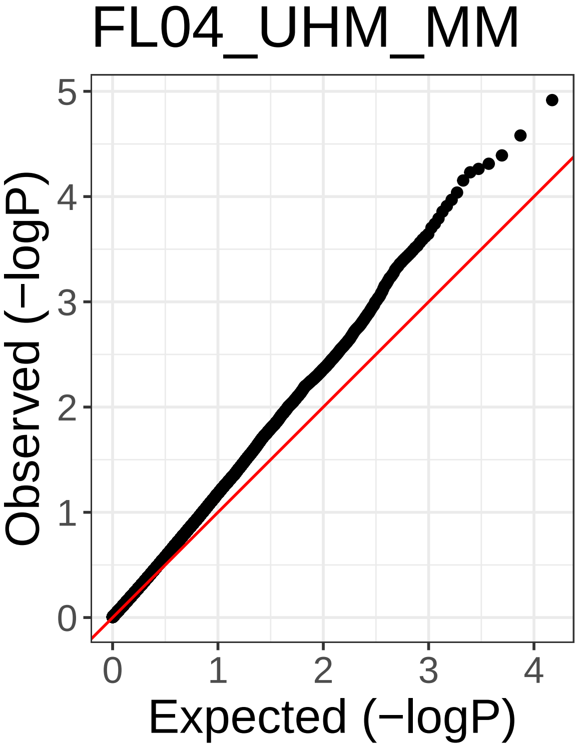
FL04_UHM_MM
Observed (−logP)
Expected (−logP)
0
1
2
3
4
5
0
1
2
3
4

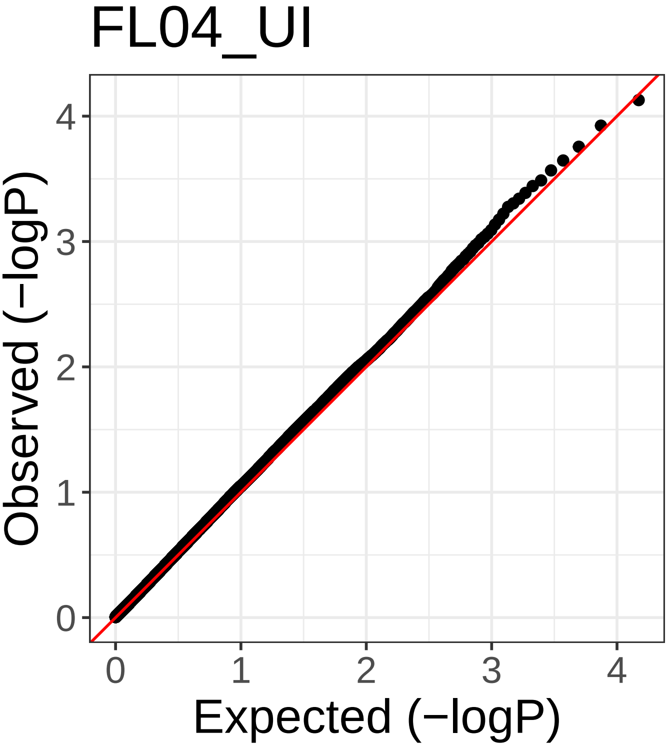
FL04_UI
Observed (−logP)
Expected (−logP)
0
1
2
3
4
0
1
2
3
4

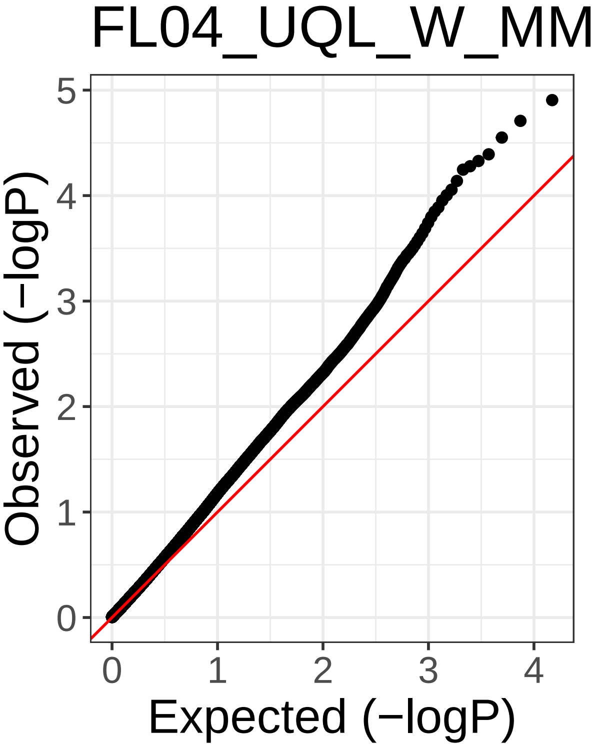
FL04_UQL_W_MM
Observed (−logP)
Expected (−logP)
0
1
2
3
4
5
0
1
2
3
4

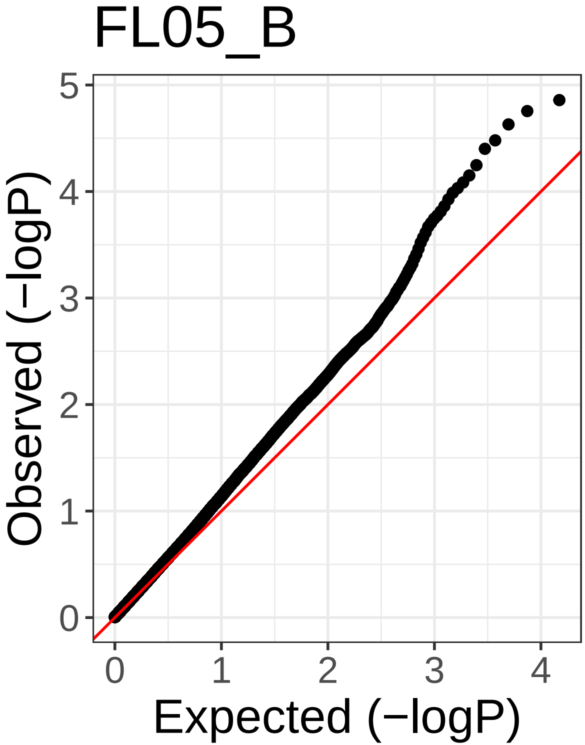

FL05_B
Observed (−logP)
Expected (−logP)
0
1
2
3
4
5
0
1
2
3
4

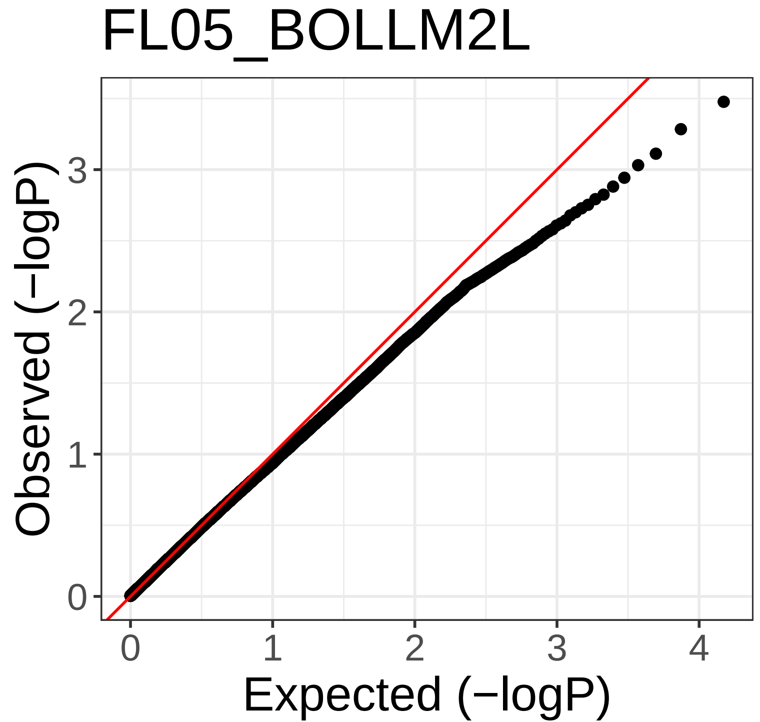
FL05_BOLLM2L
Observed (−logP)
Expected (−logP)
0
1
2
3
4

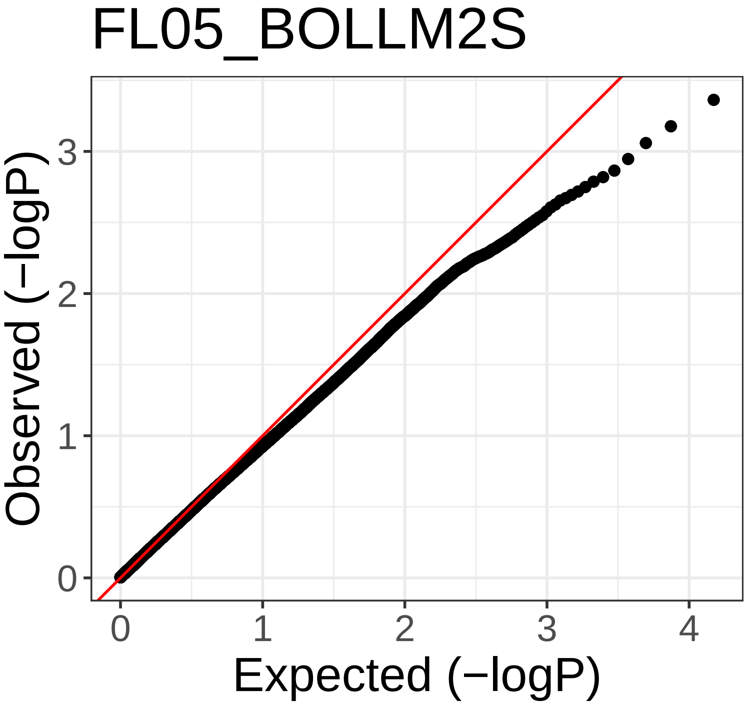
FL05_BOLLM2S
Observed (−logP)
Expected (−logP)
0
1
2
3
4

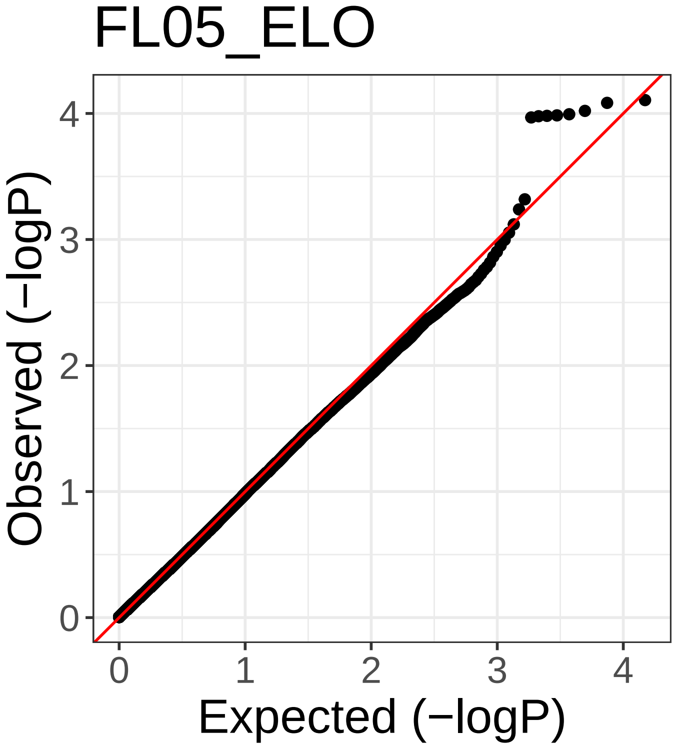
FL05_ELO
Observed (−logP)
Expected (−logP)
0
1
2
3
4
0
1
2
3
4

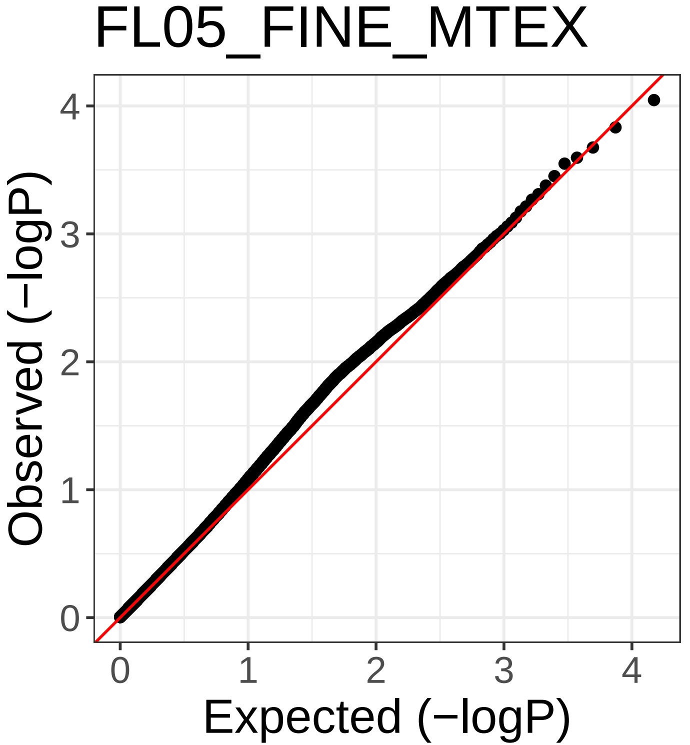
FL05_FINE_MTEX
Observed (−logP)
Expected (−logP)
0
1
2
3
4
0
1
2
3
4

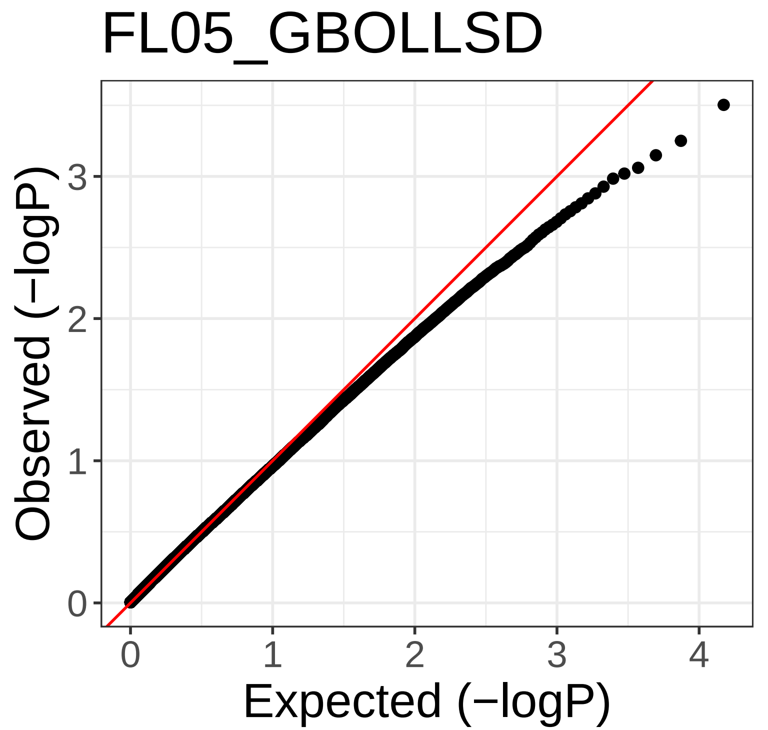
FL05_GBOLLSD
Observed (−logP)
Expected (−logP)
0
1
2
3
4

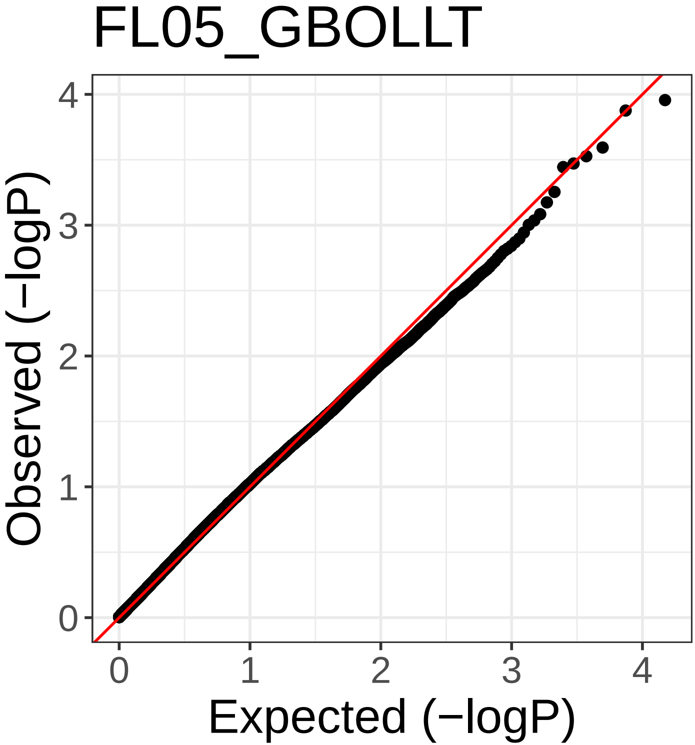
FL05_GBOLLT
Observed (−logP)
Expected (−logP)
0
1
2
3
4
0
1
2
3
4

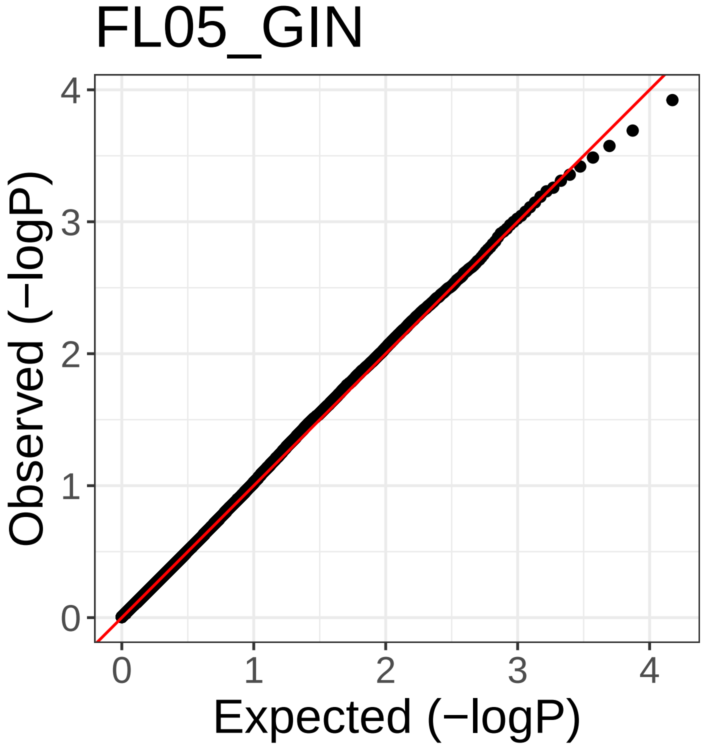

FL05_GIN
Observed (−logP)
Expected (−logP)
0
1
2
3
4
0
1
2
3
4

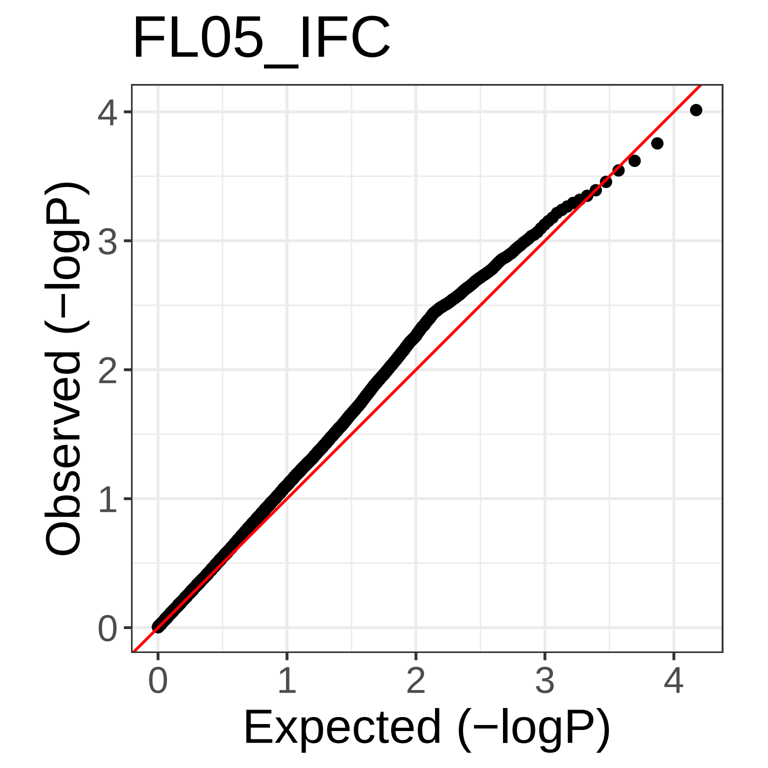
FL05_IFC
Observed (−logP)
Expected (−logP)
0
1
2
3
4
0
1
2
3
4

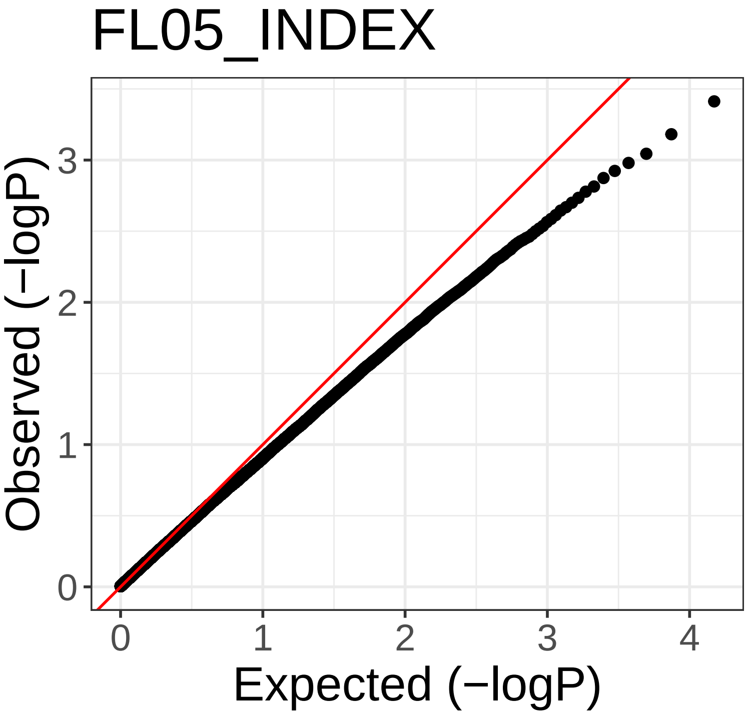
FL05_INDEX
Observed (−logP)
Expected (−logP)
0
1
2
3
4

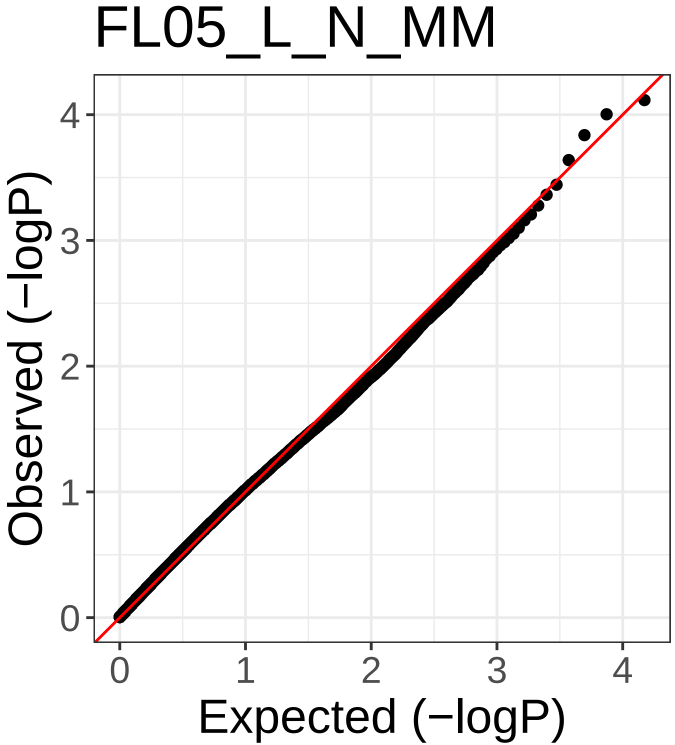
FL05_L_N_MM
Observed (−logP)
Expected (−logP)
0
1
2
3
4
0
1
2
3
4

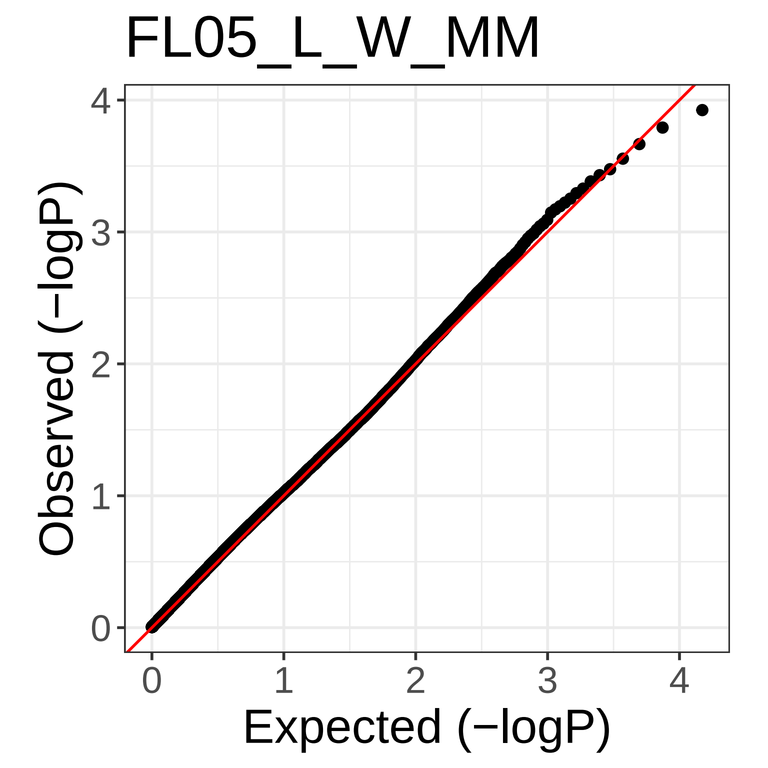

FL05_L_W_MM
Observed (−logP)
Expected (−logP)
0
1
2
3
4
0
1
2
3
4

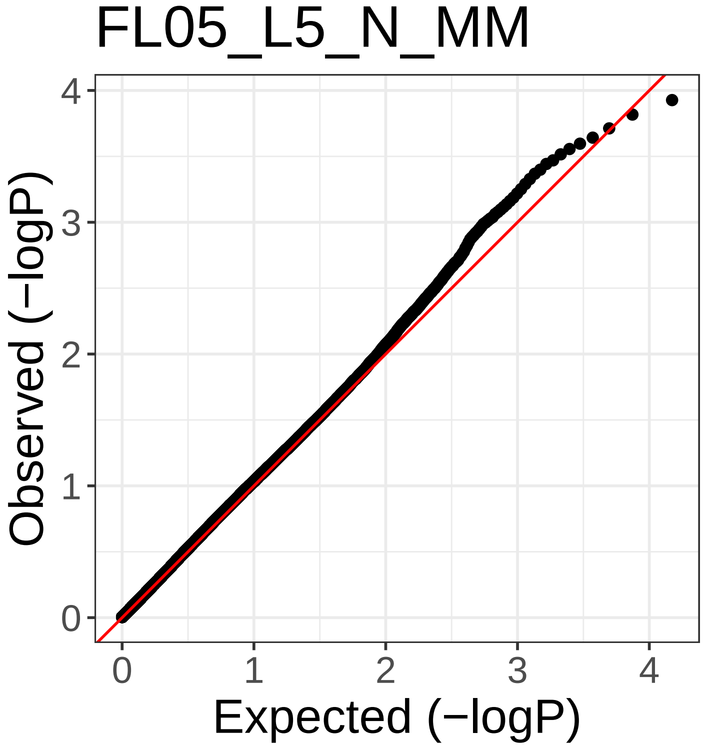
FL05_L5_N_MM
Observed (−logP)
Expected (−logP)
0
1
2
3
4
0
1
2
3
4

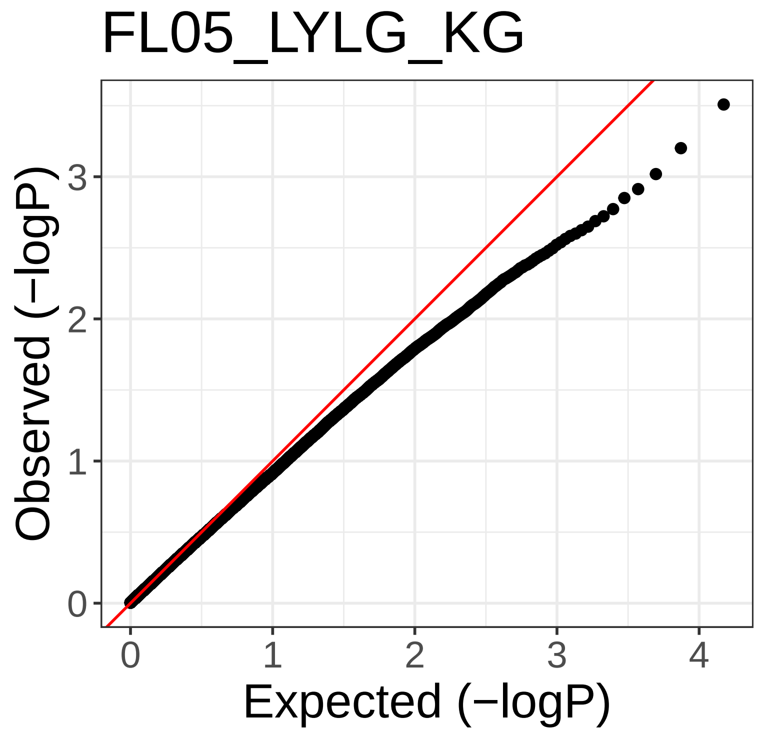
FL05_LYLG_KG
Observed (−logP)
Expected (−logP)
0
1
2
3
4

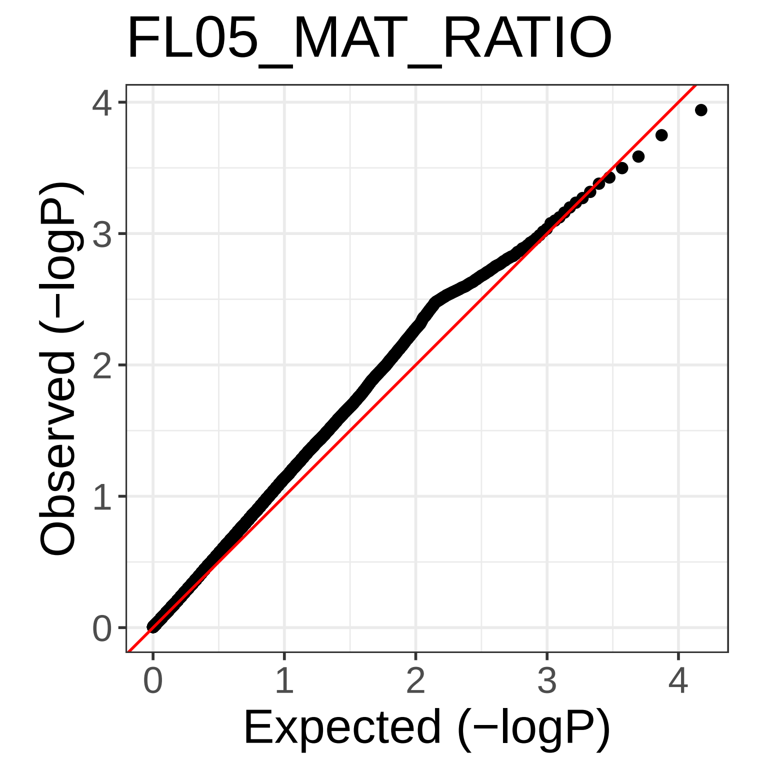
FL05_MAT_RATIO
Observed (−logP)
Expected (−logP)
0
1
2
3
4
0
1
2
3
4

FL05_MIC

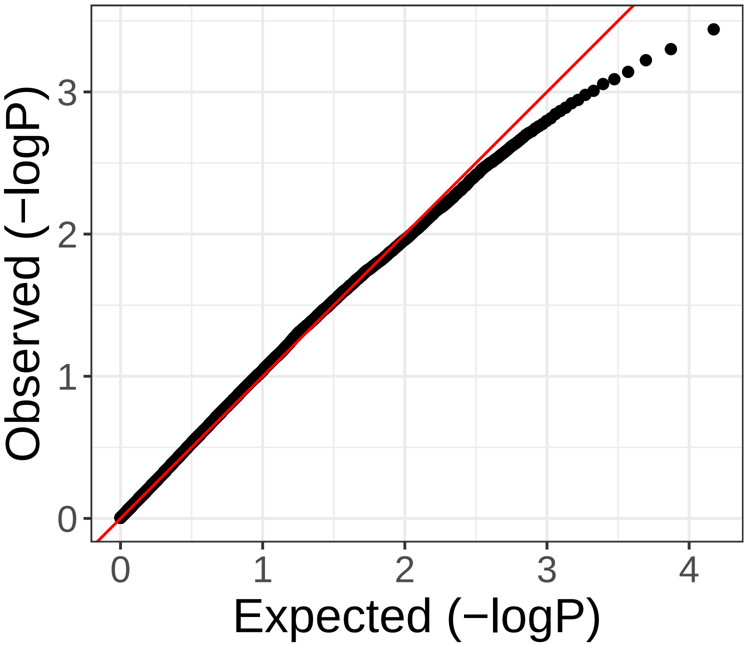

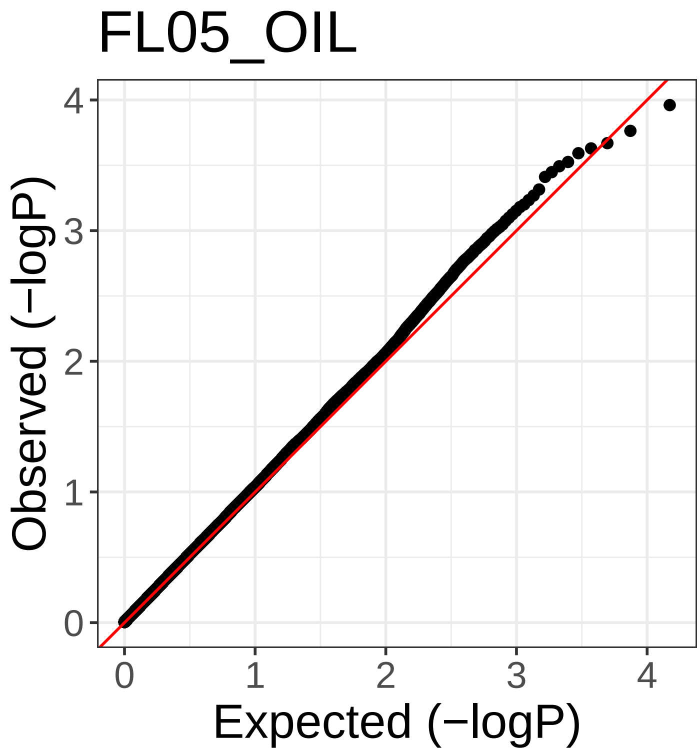
FL05_OIL
Observed (−logP)
Expected (−logP)
0
1
2
3
4

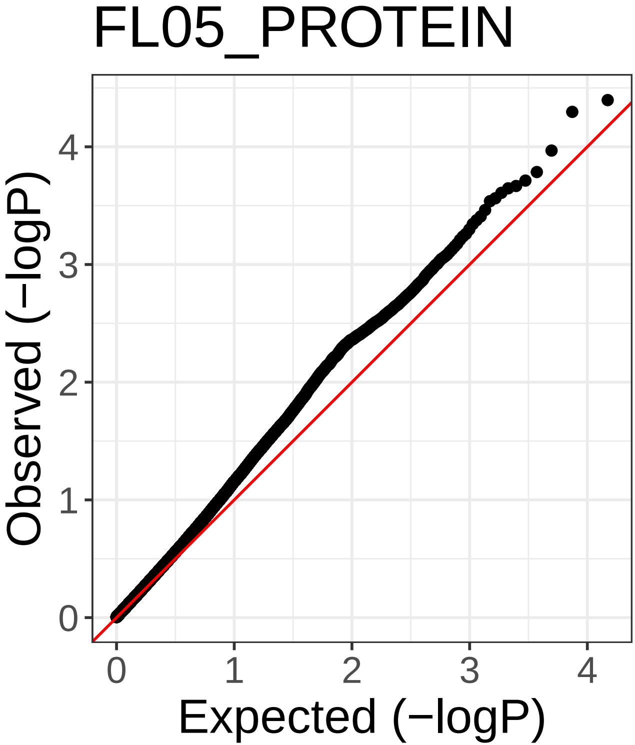
FL05_PROTEIN
Observed (−logP)
Expected (−logP)
0
1
2
3
4
0
1
2
3
4

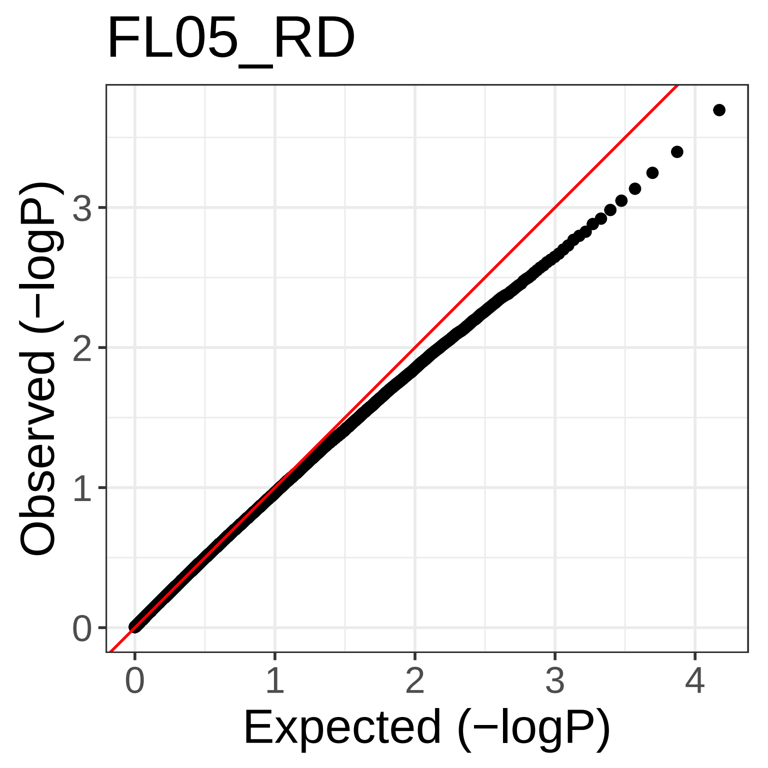
FL05_RD
Observed (−logP)
Expected (−logP)
0
1
2
3
4

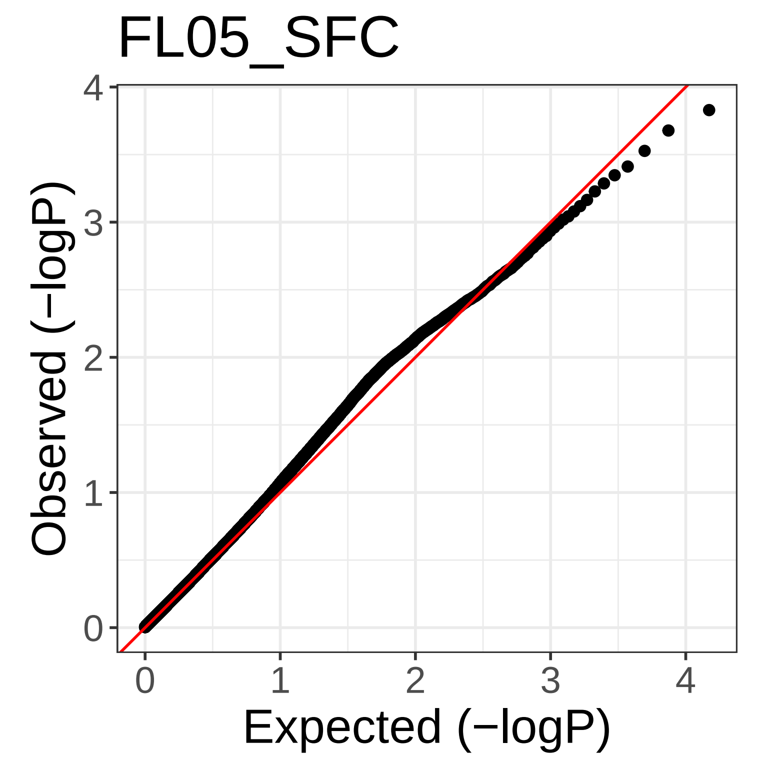
FL05_SFC
Observed (−logP)
Expected (−logP)
0
1
2
3
4
0
1
2
3
4

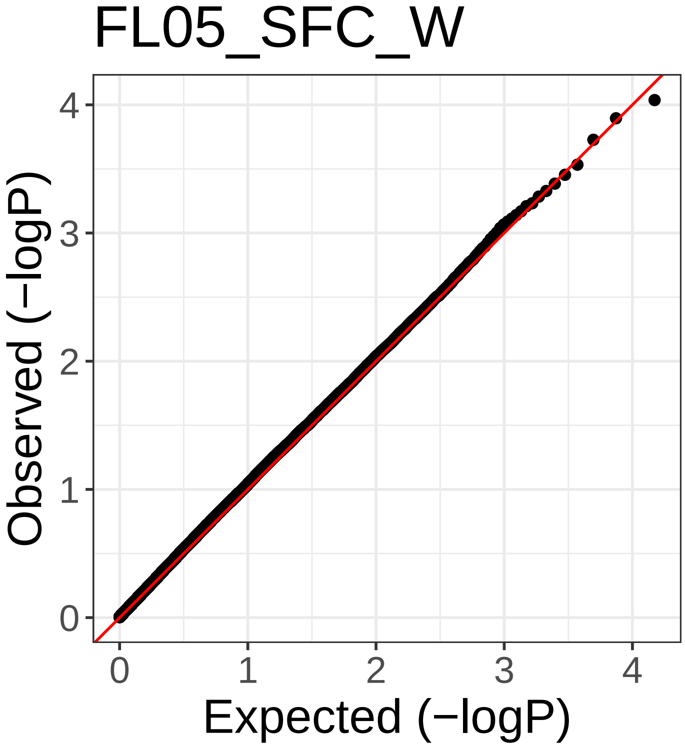
FL05_SFC_W
Observed (−logP)
Expected (−logP)
0
1
2
3
4
0
1
2
3
4

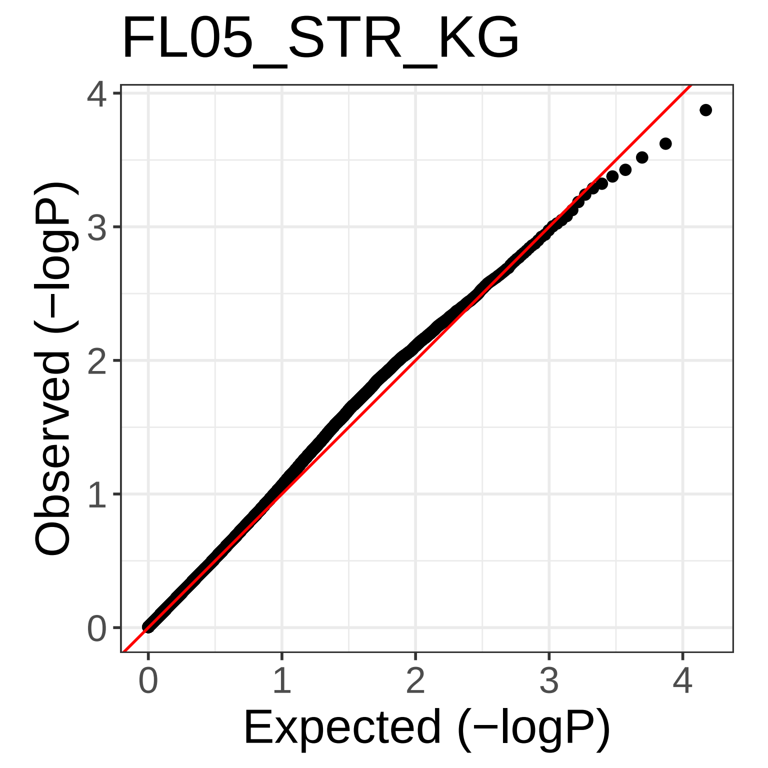
FL05_STR_KG
Observed (−logP)
Expected (−logP)
0
1
2
3
4
0
1
2
3
4

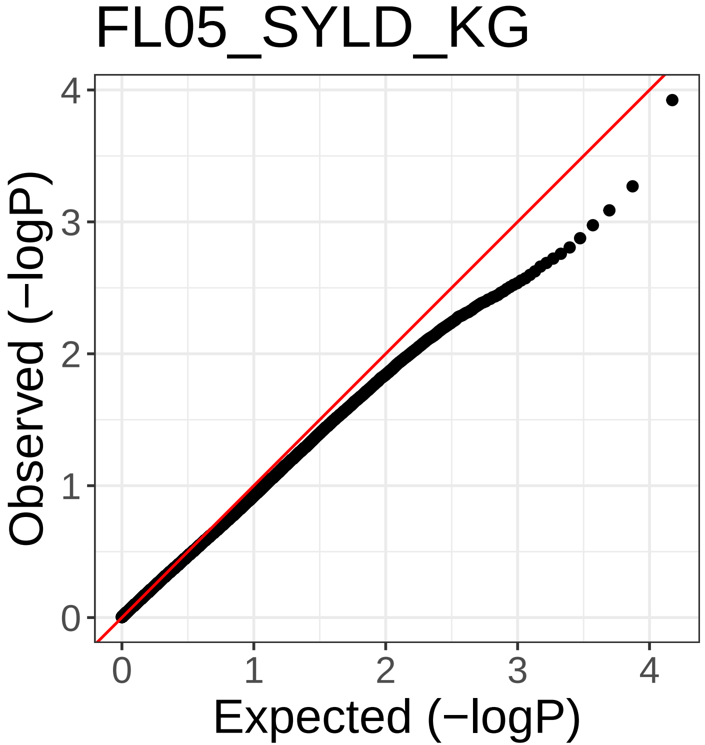
FL05_SYLD_KG
Observed (−logP)
Expected (−logP)
0
1
2
3
4
0
1
2
3
4

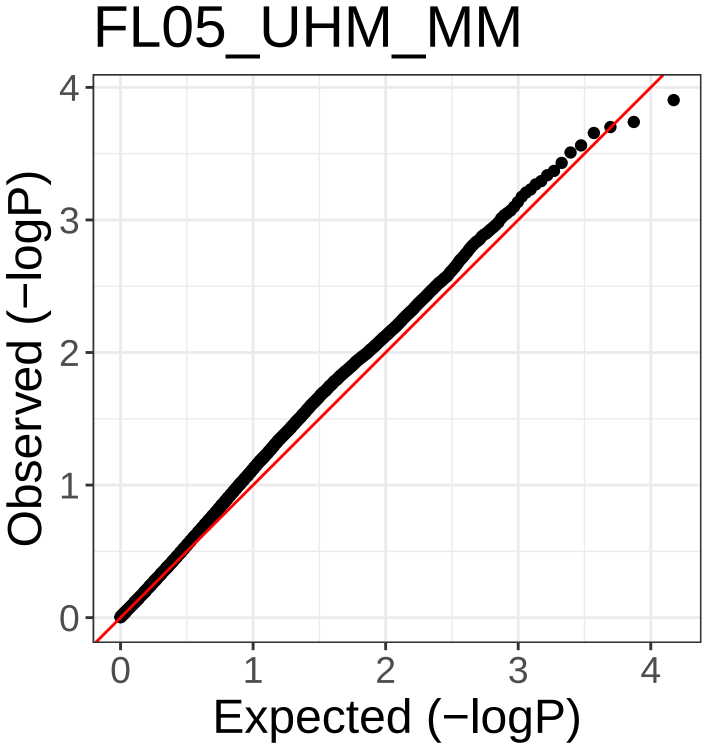
FL05_UHM_MM
Observed (−logP)
Expected (−logP)
0
1
2
3
4
0
1
2
3
4

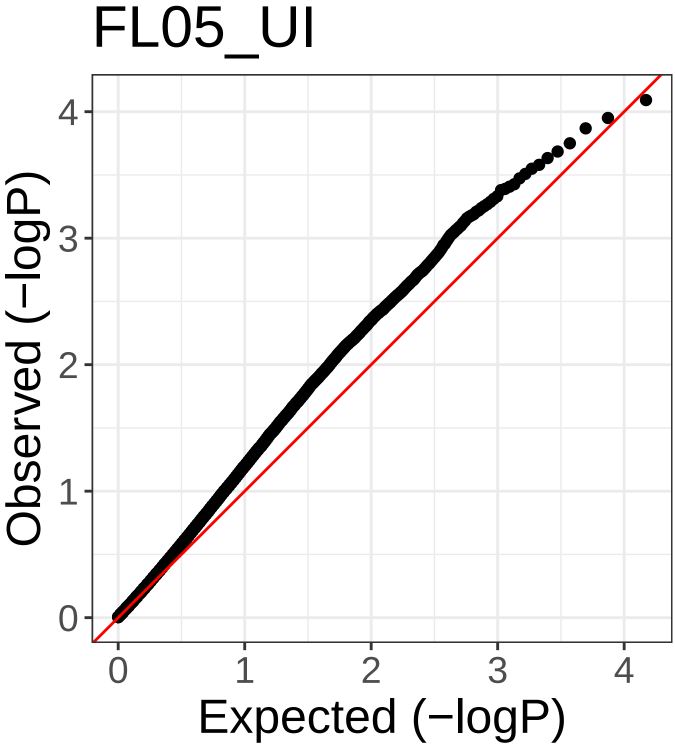
FL05_UI
Observed (−logP)
Expected (−logP)
0
1
2
3
4

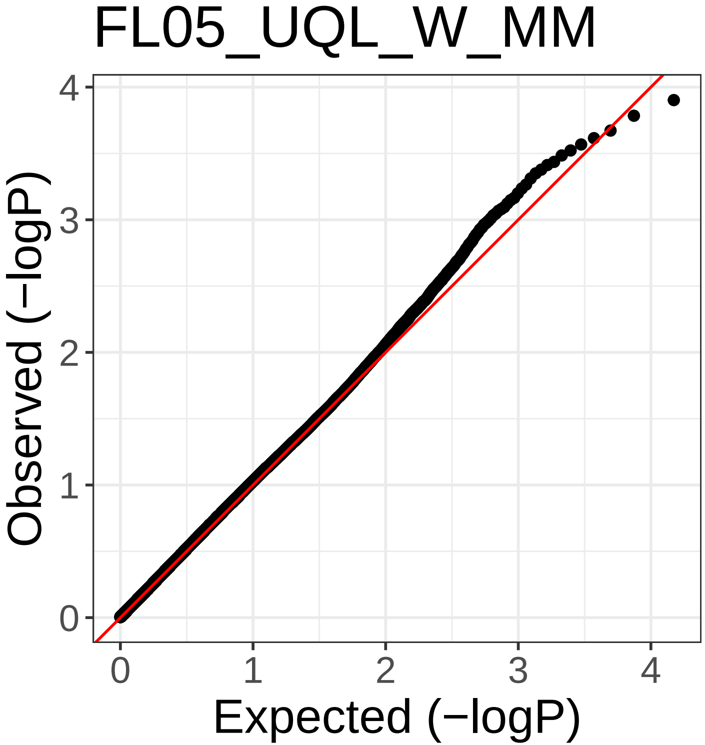

FL05_UQL_W_MM
Observed (−logP)
Expected (−logP)
0
1
2
3
4
0
1
2
3
4

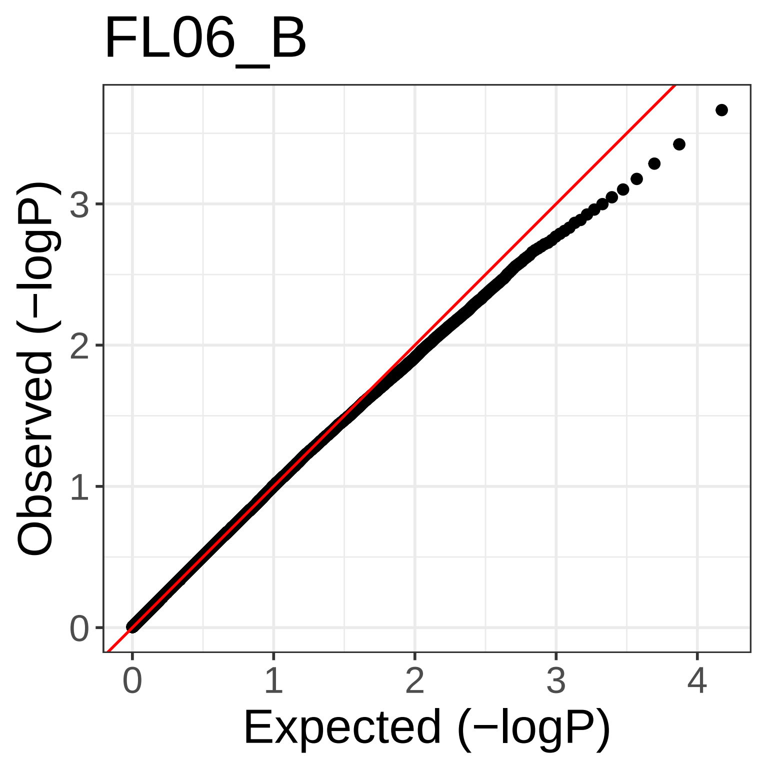
FL06_B
Observed (−logP)
Expected (−logP)
0
1
2
3
4

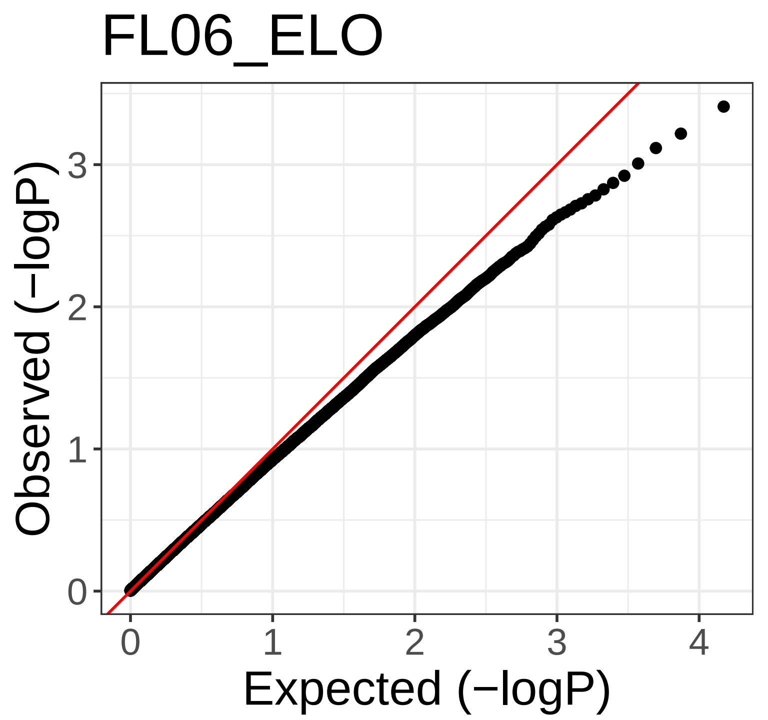
FL06_ELO
Observed (−logP)
Expected (−logP)
0
1
2
3
4

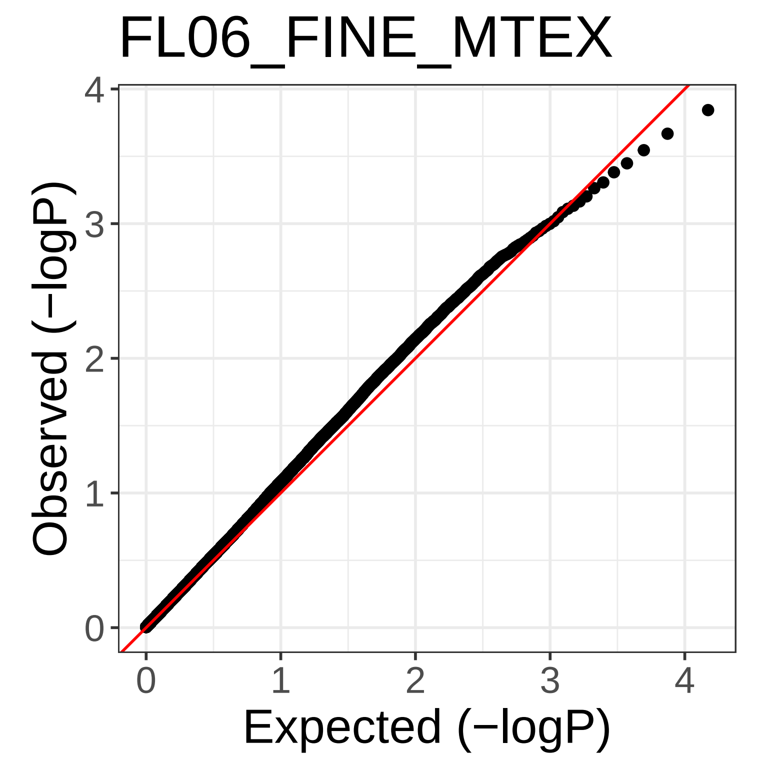

FL06_FINE_MTEX
Observed (−logP)
Expected (−logP)
0
1
2
3
4
0
1
2
3
4

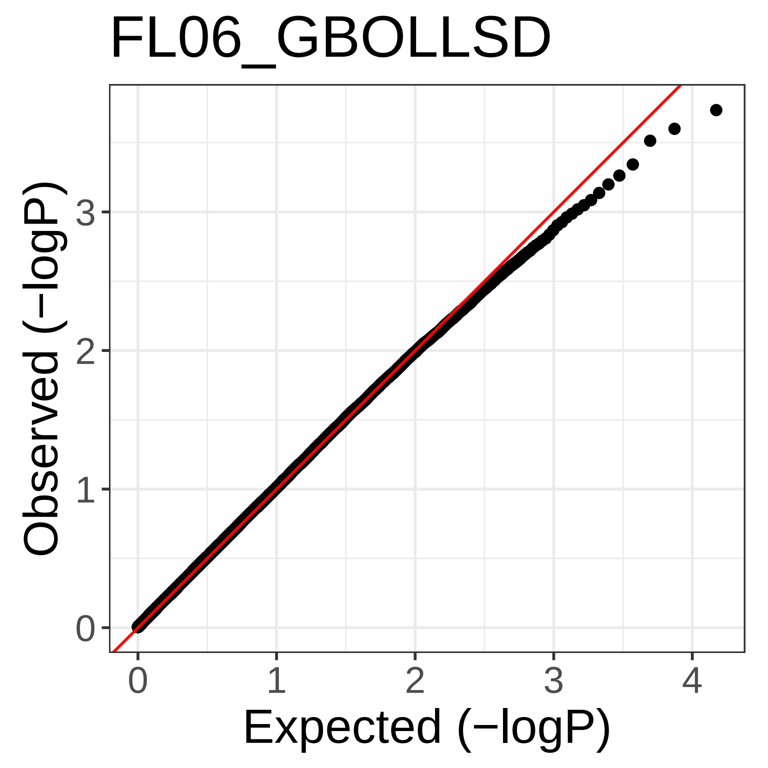
FL06_GBOLLSD
Observed (−logP)
Expected (−logP)
0
1
2
3
0
1
2
3
4

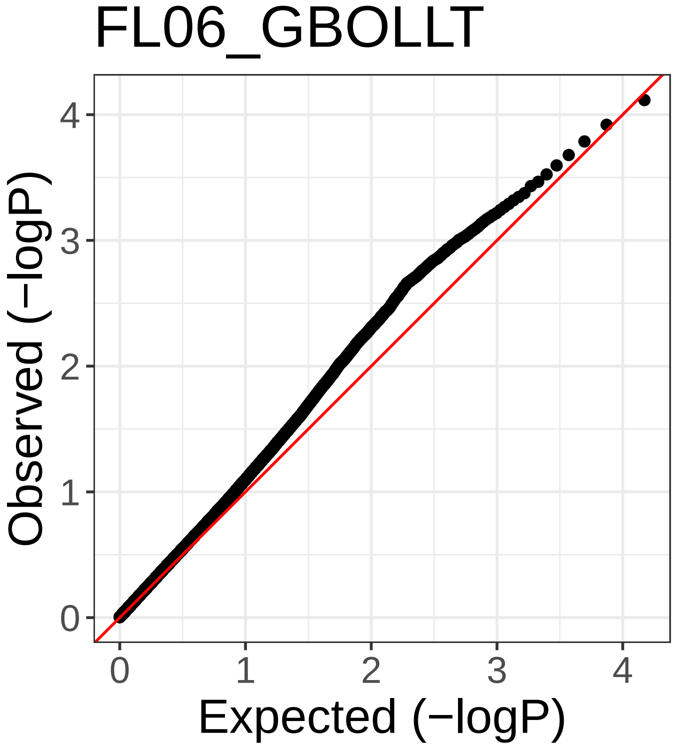
FL06_GBOLLT
Observed (−logP)
Expected (−logP)
0
1
2
3
4
0
1
2
3
4

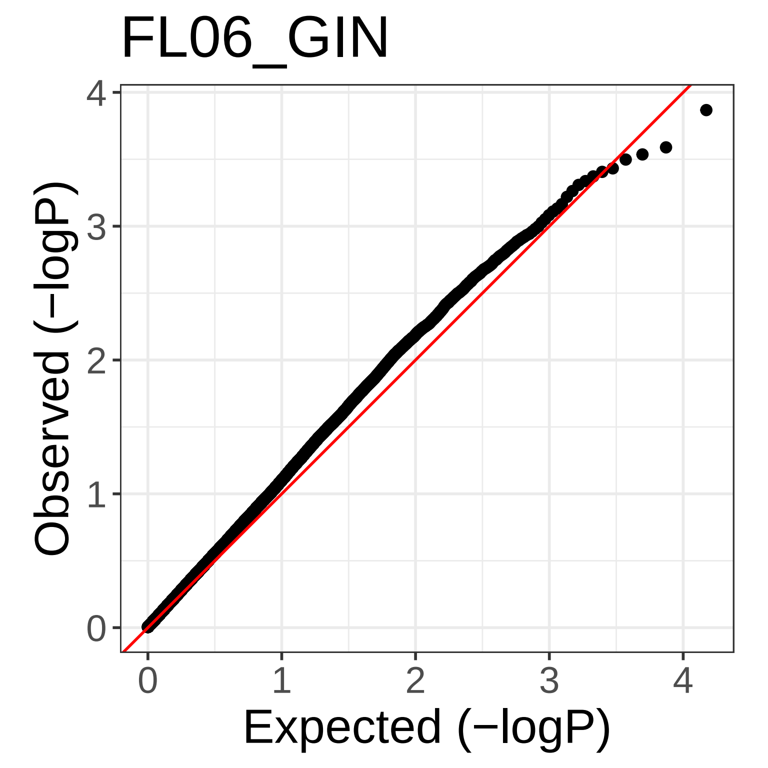
FL06_GIN
Observed (−logP)
Expected (−logP)
0
1
2
3
4

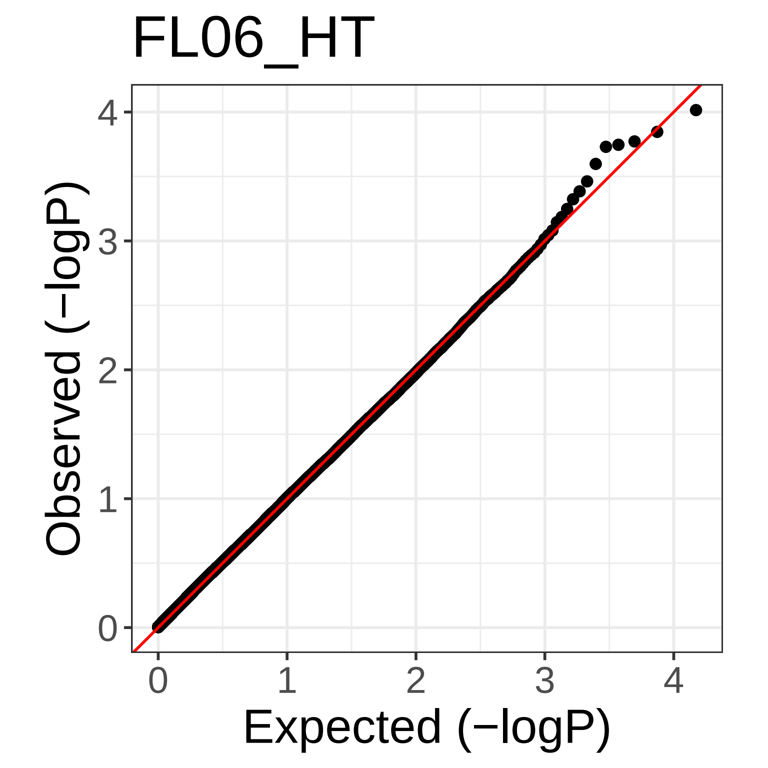
FL06_HT
Observed (−logP)
Expected (−logP)
0
1
2
3
4
0
1
2
3
4

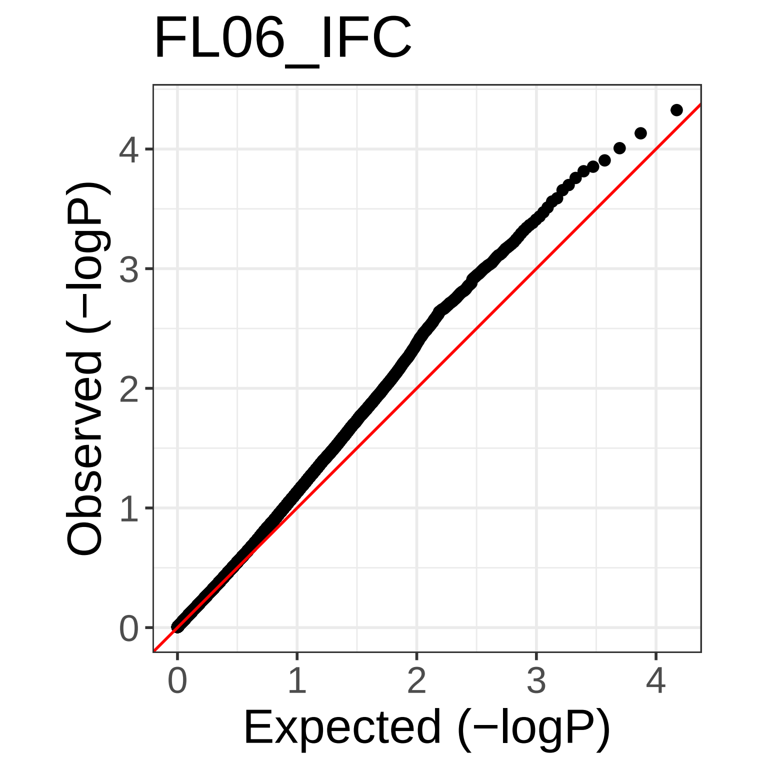

FL06_IFC
Observed (−logP)
Expected (−logP)
0
1
2
3
4
0
1
2
3
4

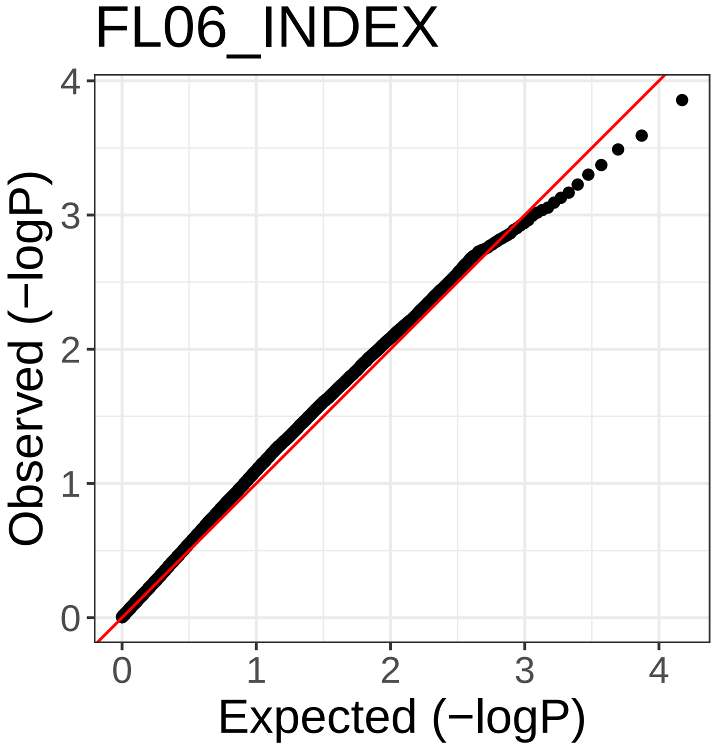
FL06_INDEX
Observed (−logP)
Expected (−logP)
0
1
2
3
4
0
1
2
3
4

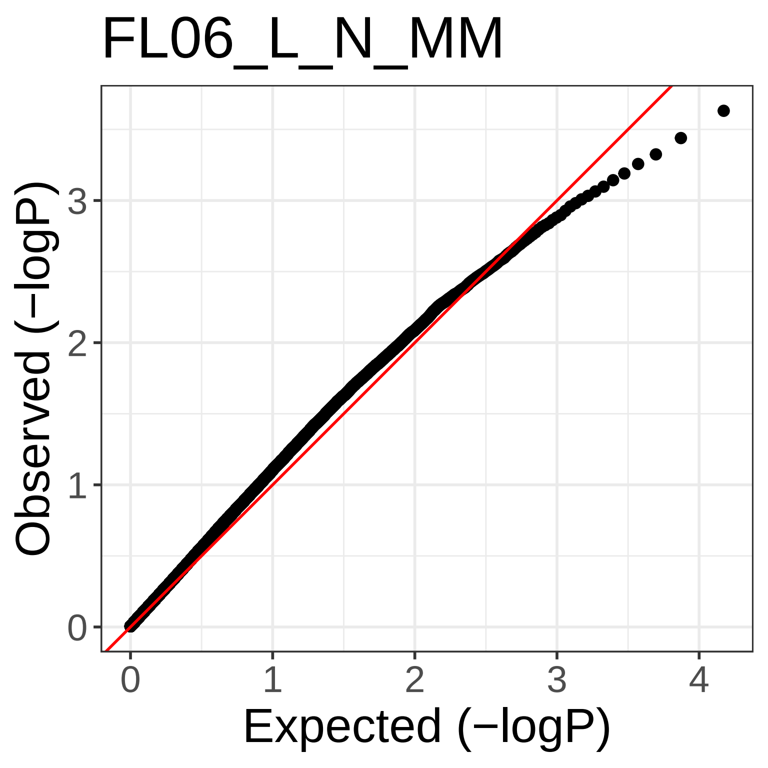

FL06_L_N_MM
Observed (−logP)
Expected (−logP)
0
1
2
3
4

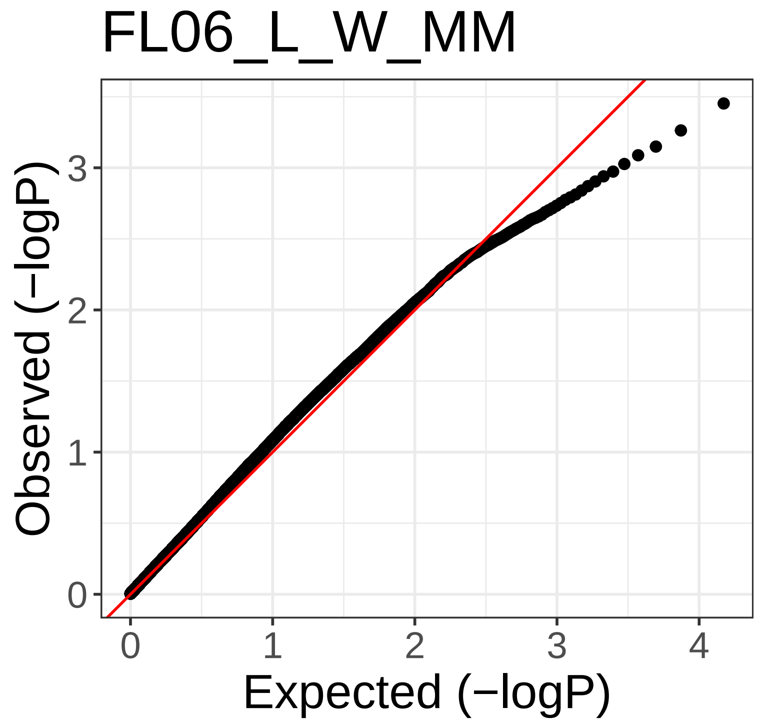

FL06_L_W_MM
Observed (−logP)
Expected (−logP)
0
1
2
3
0
1
2
3
4

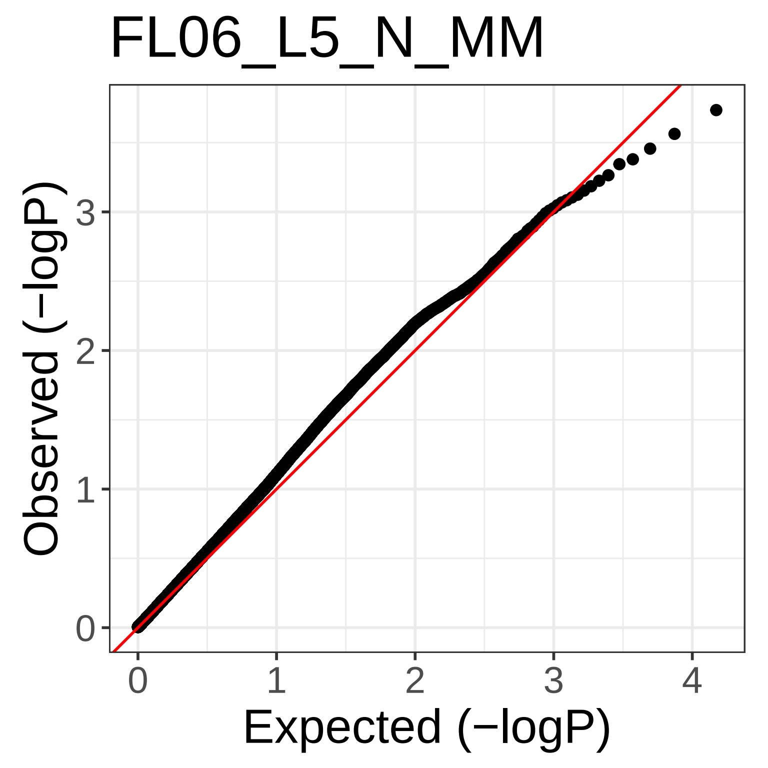
FL06_L5_N_MM
Observed (−logP)
Expected (−logP)
0
1
2
3
0
1
2
3
4

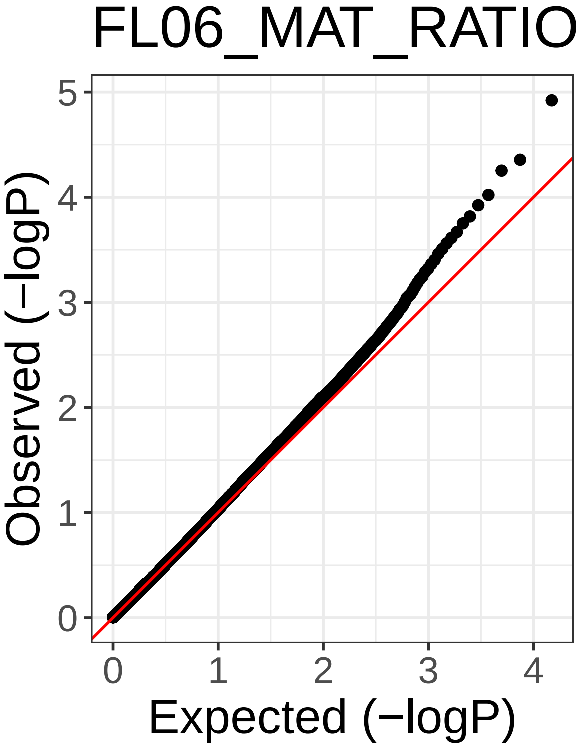

FL06_MAT_RATIO
Observed (−logP)
Expected (−logP)
0
1
2
3
4
5

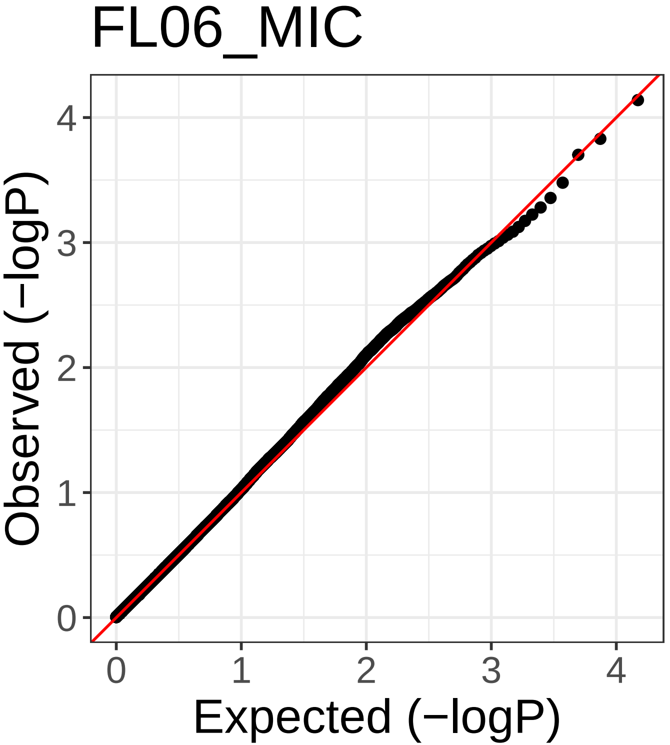
FL06_MIC
Observed (−logP)
Expected (−logP)
0
1
2
3
4
0
1
2
3
4

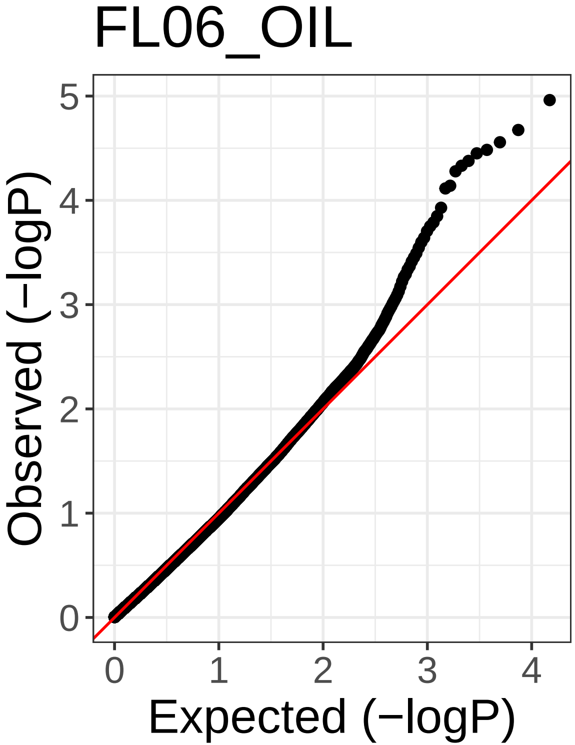
FL06_OIL
Observed (−logP)
Expected (−logP)
0
1
2
3
4
5
0
1
2
3
4

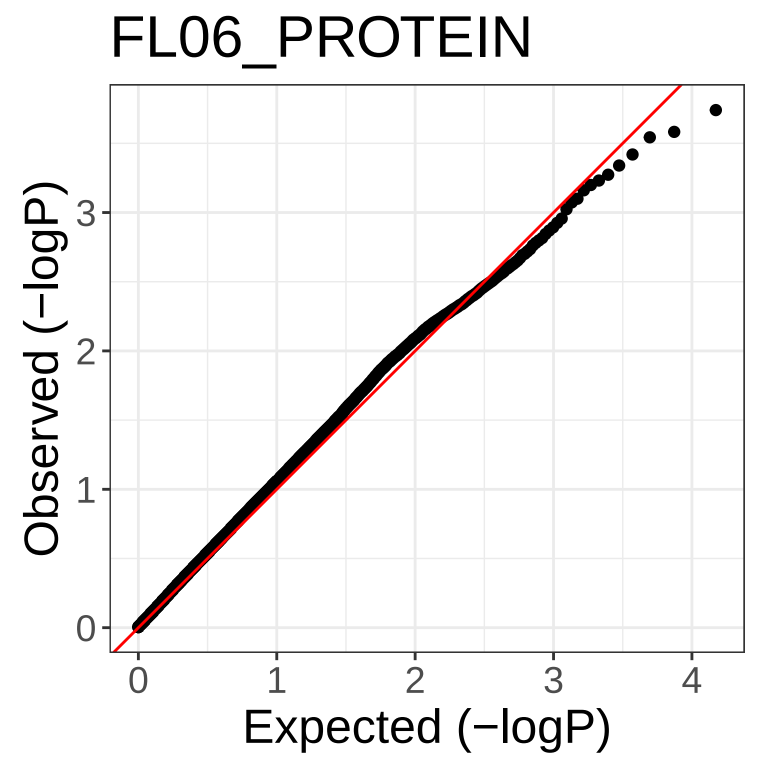
FL06_PROTEIN
Observed (−logP)
Expected (−logP)
0
1
2
3
4

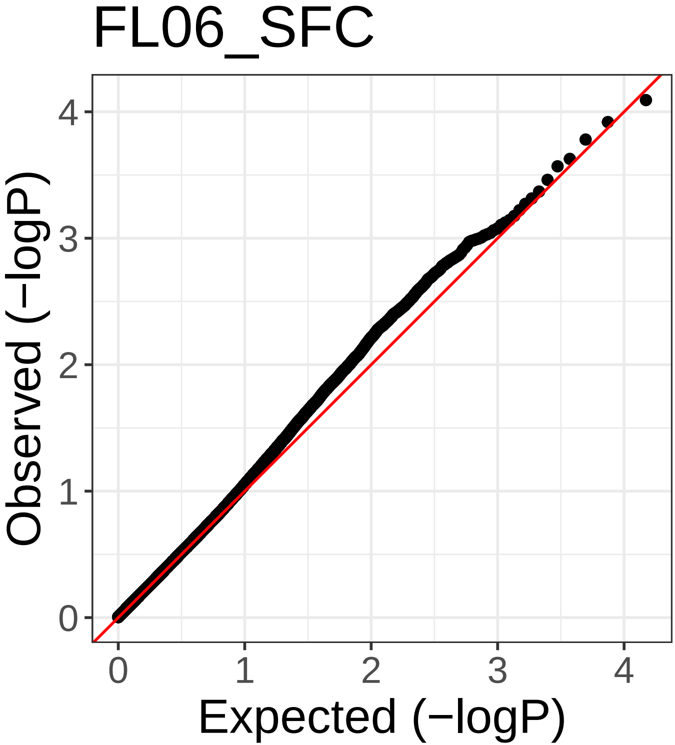
FL06_SFC
Observed (−logP)
Expected (−logP)
0
1
2
3
4
0
1
2
3
4

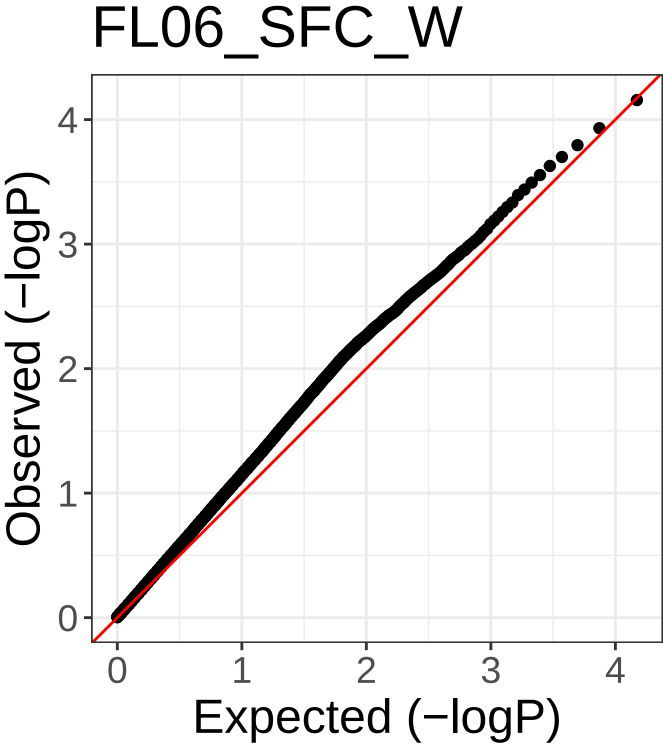
FL06_SFC_W
Observed (−logP)
Expected (−logP)
0
1
2
3
4
0
1
2
3
4

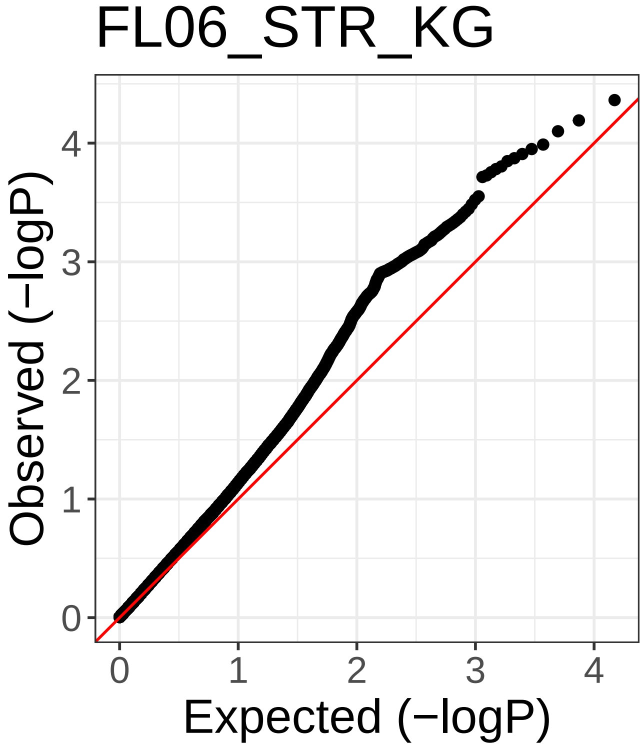

FL06_STR_KG
Observed (−logP)
Expected (−logP)
0
1
2
3
4
0
1
2
3
4

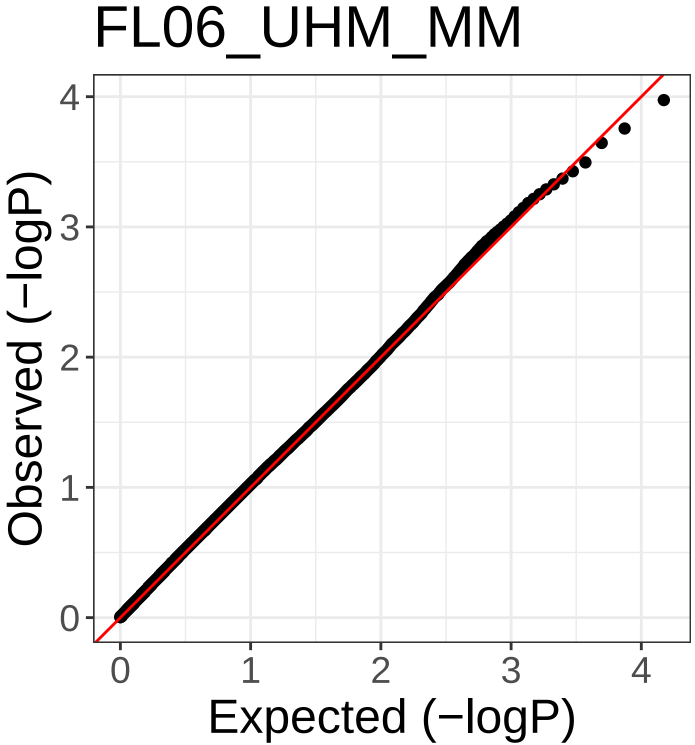
FL06_UHM_MM
Observed (−logP)
Expected (−logP)
0
1
2
3
4

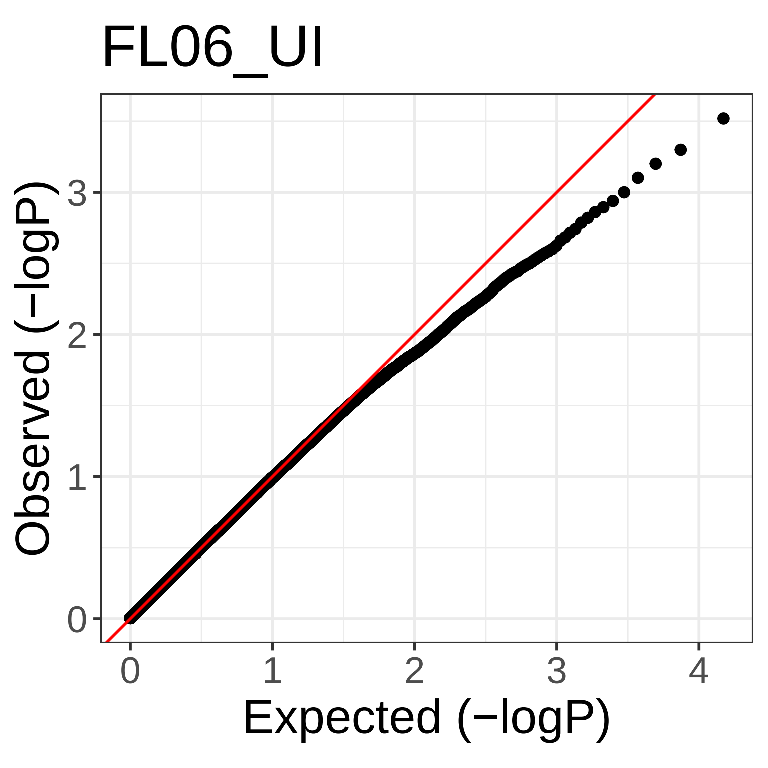
FL06_UI
Observed (−logP)
Expected (−logP)
0
1
2
3
0
1
2
3
4

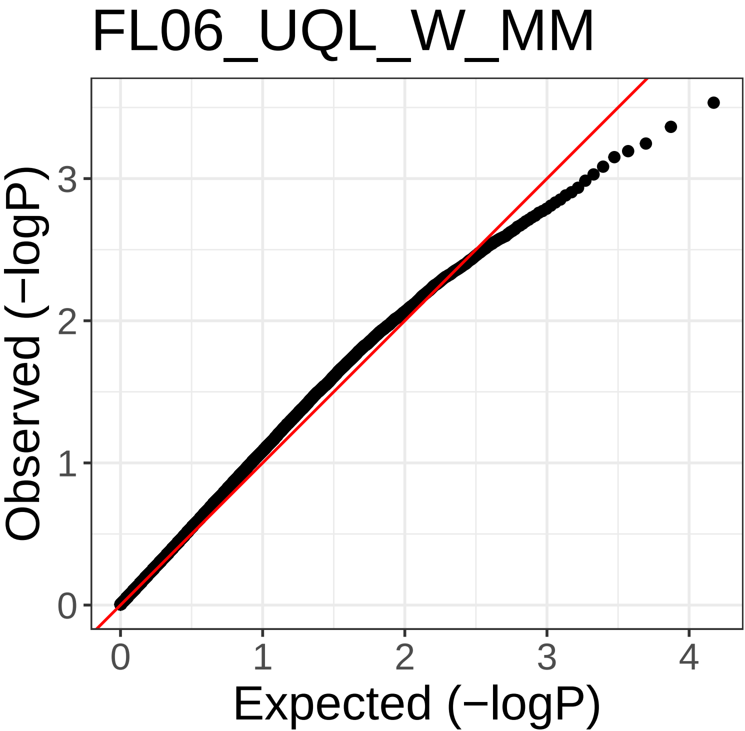
FL06_UQL_W_MM
Observed (−logP)
Expected (−logP)
0
1
2
3
0
1
2
3
4

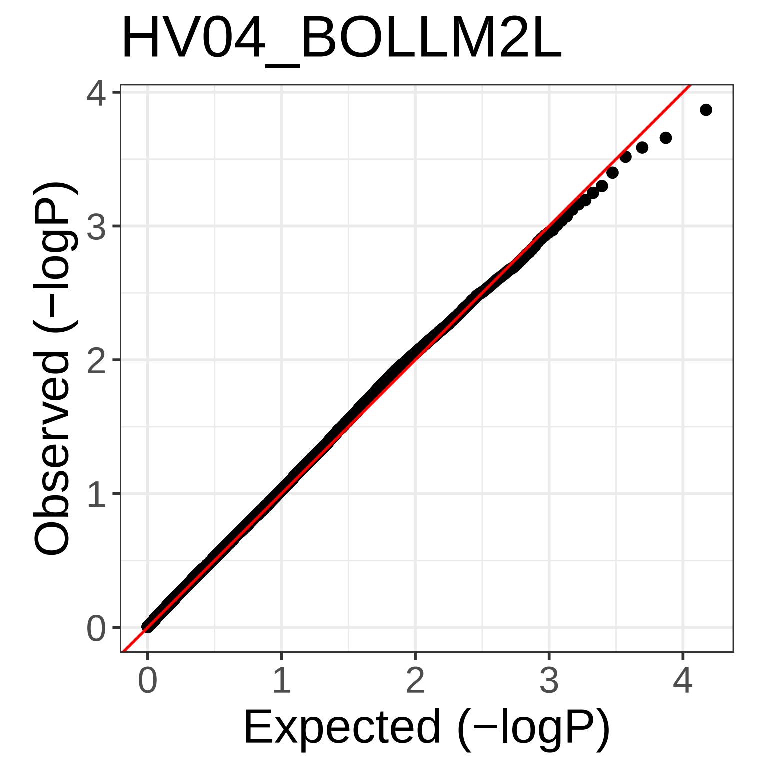
HV04_BOLLM2L
Observed (−logP)
Expected (−logP)
0
1
2
3
4

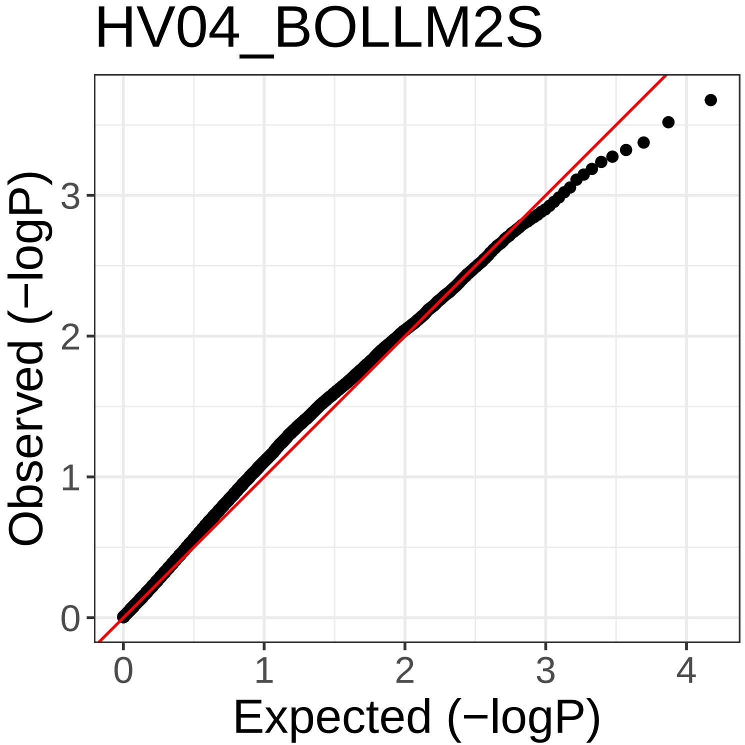
HV04_BOLLM2S
Observed (−logP)
Expected (−logP)
0
1
2
3
0
1
2
3
4

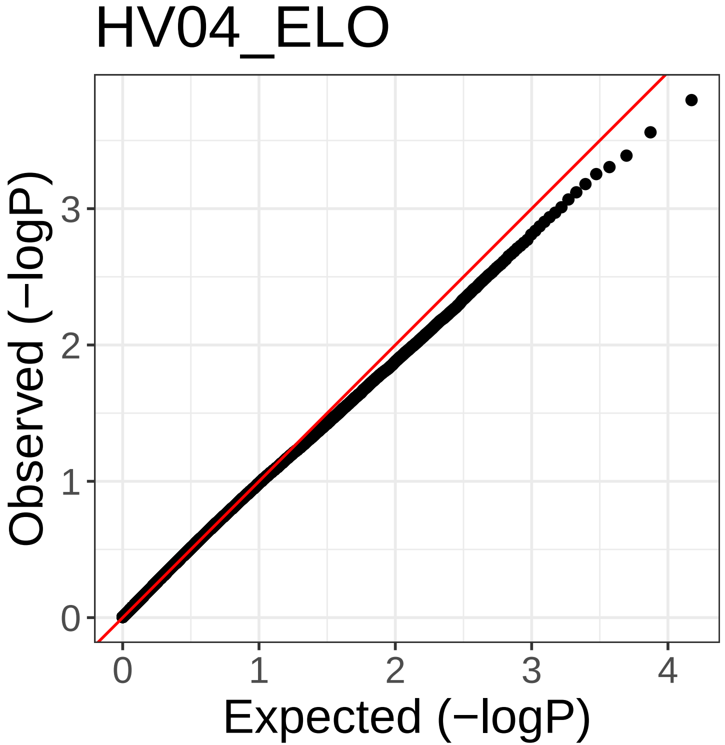
HV04_ELO
Observed (−logP)
Expected (−logP)
0
1
2
3
4

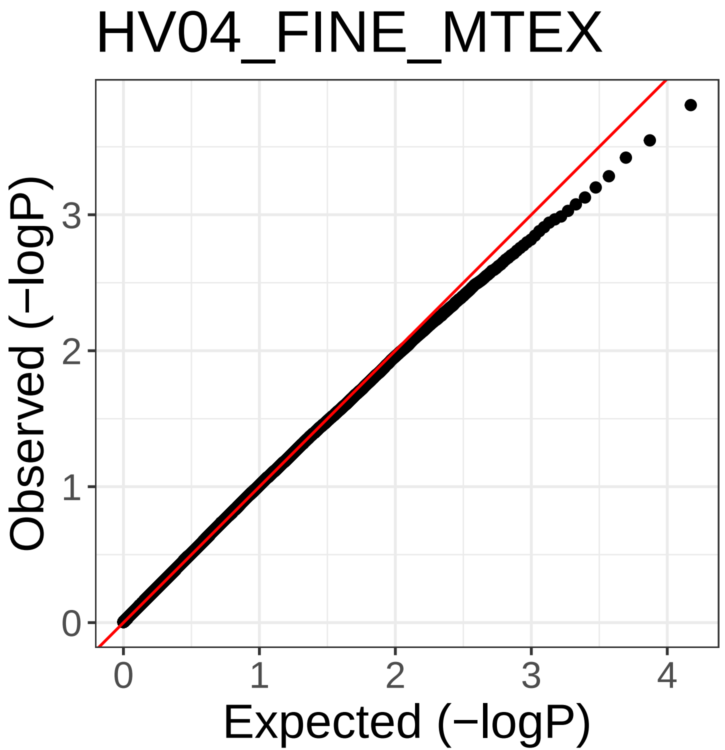
HV04_FINE_MTEX
Observed (−logP)
Expected (−logP)
0
1
2
3
0
1
2
3
4

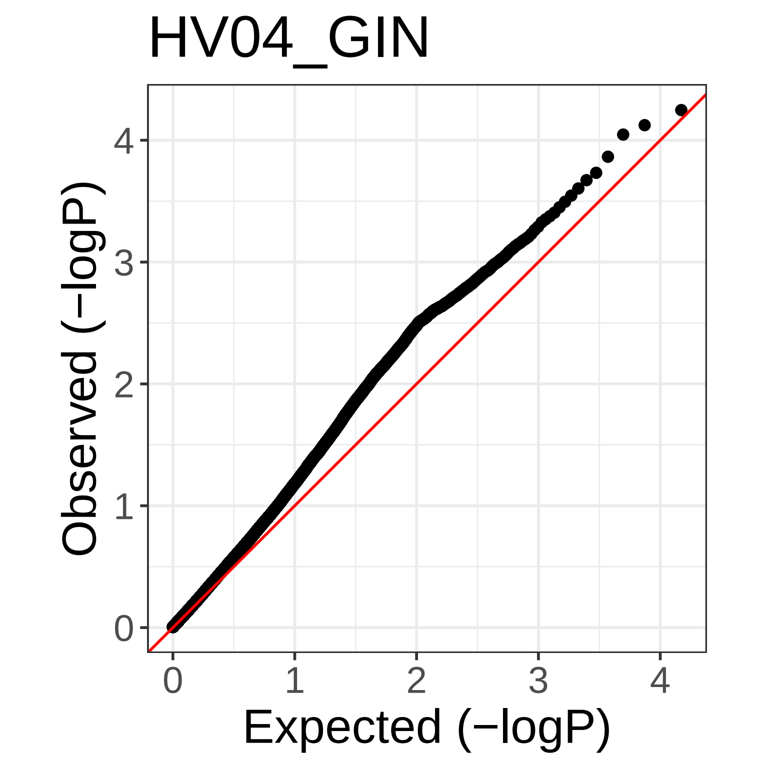

HV04_GIN
Observed (−logP)
Expected (−logP)
0
1
2
3
4
0
1
2
3
4

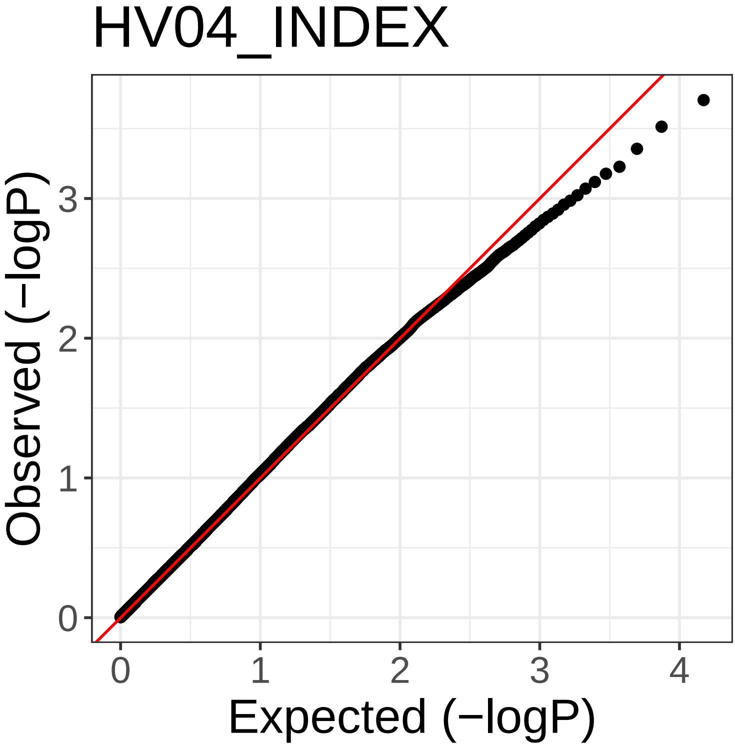

HV04_INDEX
Observed (−logP)
Expected (−logP)
0
1
2
3
4

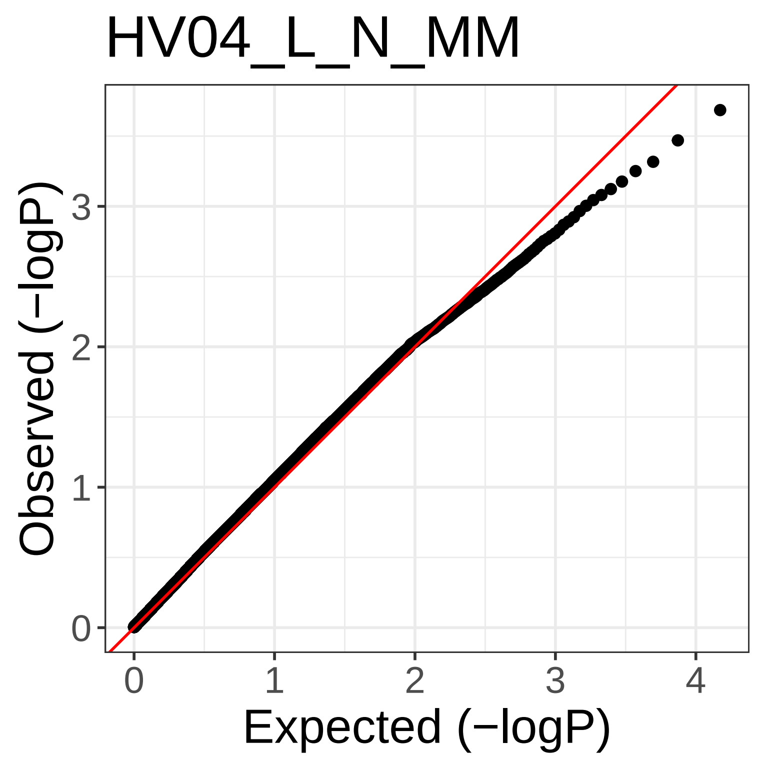
HV04_L_N_MM
Observed (−logP)
Expected (−logP)
0
1
2
3
4

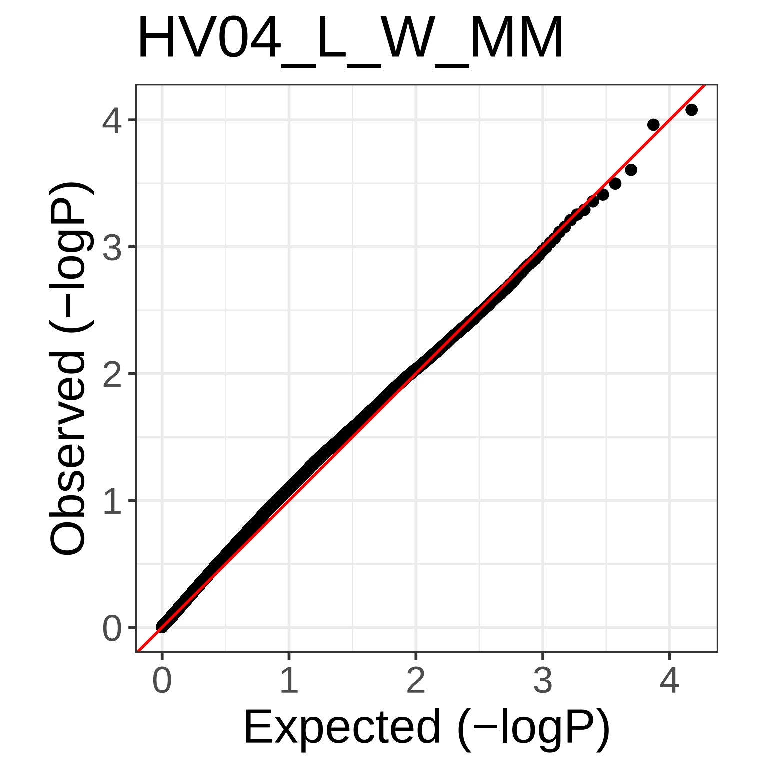
HV04_L_W_MM
Observed (−logP)
Expected (−logP)
0
1
2
3
4
0
1
2
3
4

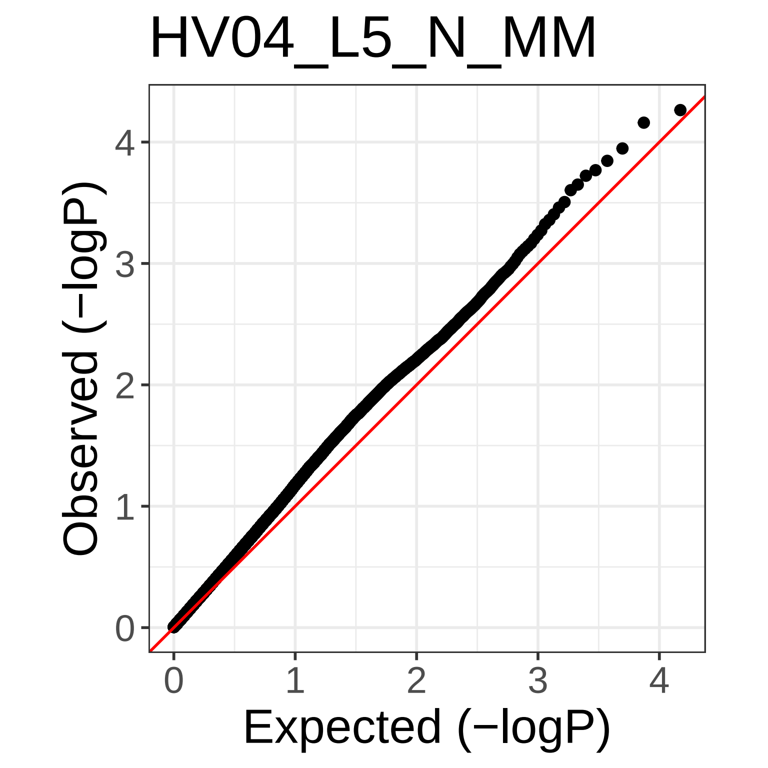
HV04_L5_N_MM
Observed (−logP)
Expected (−logP)
0
1
2
3
4
0
1
2
3
4

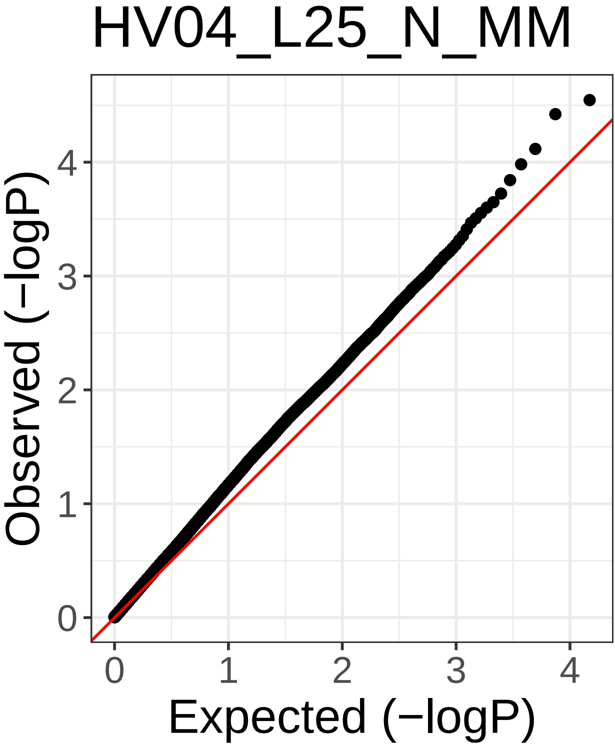

HV04_L25_N_MM
Observed (−logP)
Expected (−logP)
0
1
2
3
4
0
1
2
3
4

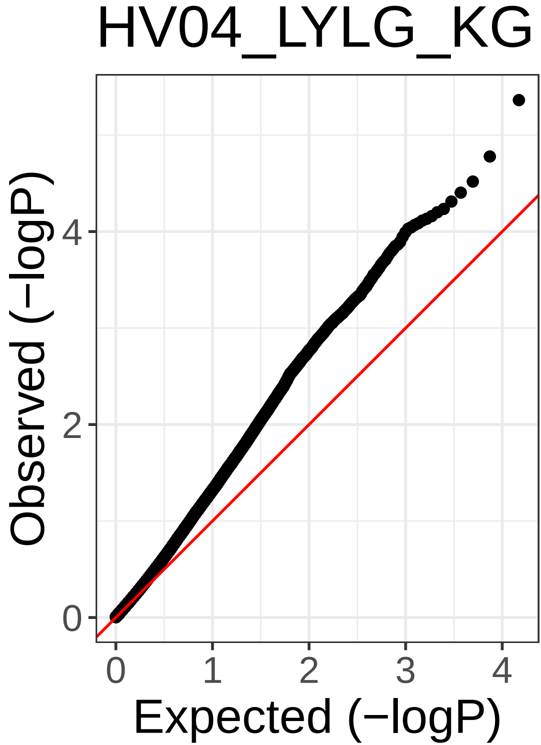
HV04_LYLG_KG
Observed (−logP)
Expected (−logP)
0
2
4
0
1
2
3
4

HV04_MAT_RATIO

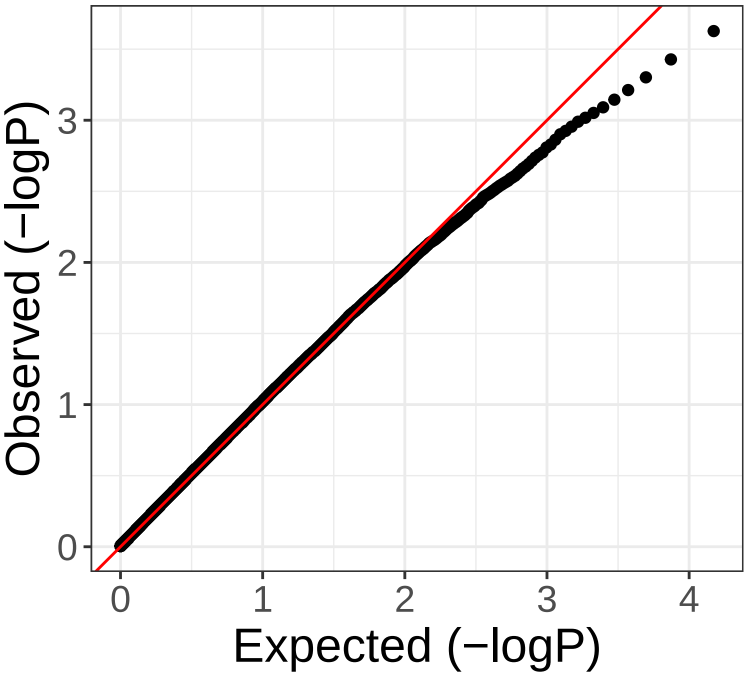

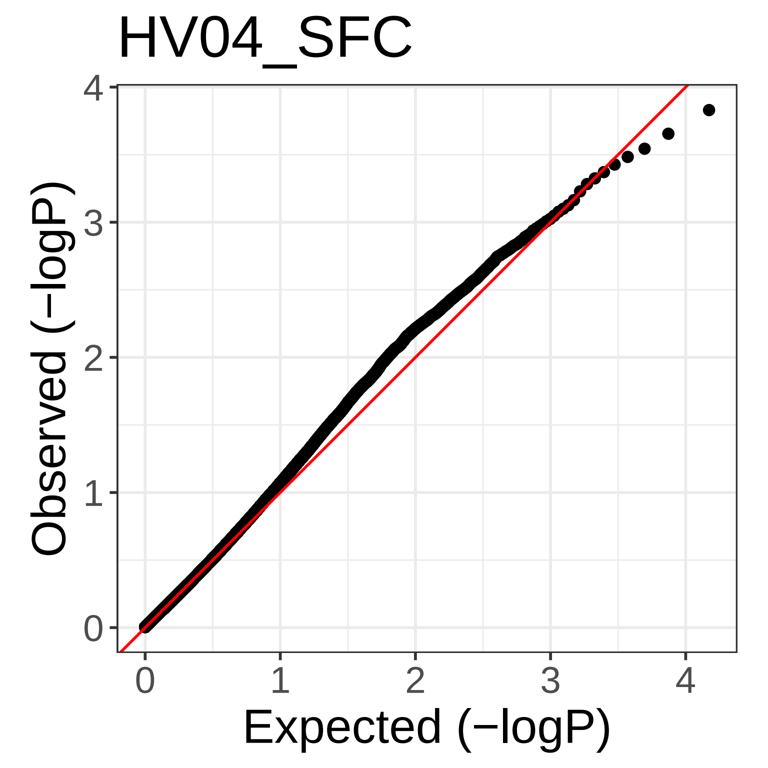
HV04_SFC
Observed (−logP)
Expected (−logP)
0
1
2
3
4
0
1
2
3
4

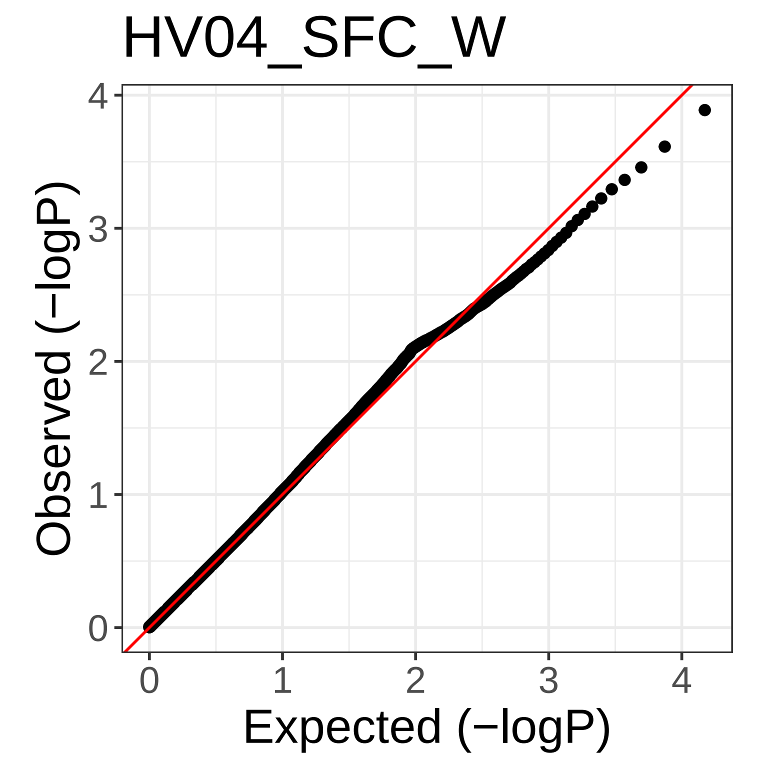

HV04_SFC_W
Observed (−logP)
Expected (−logP)
0
1
2
3
4
0
1
2
3
4

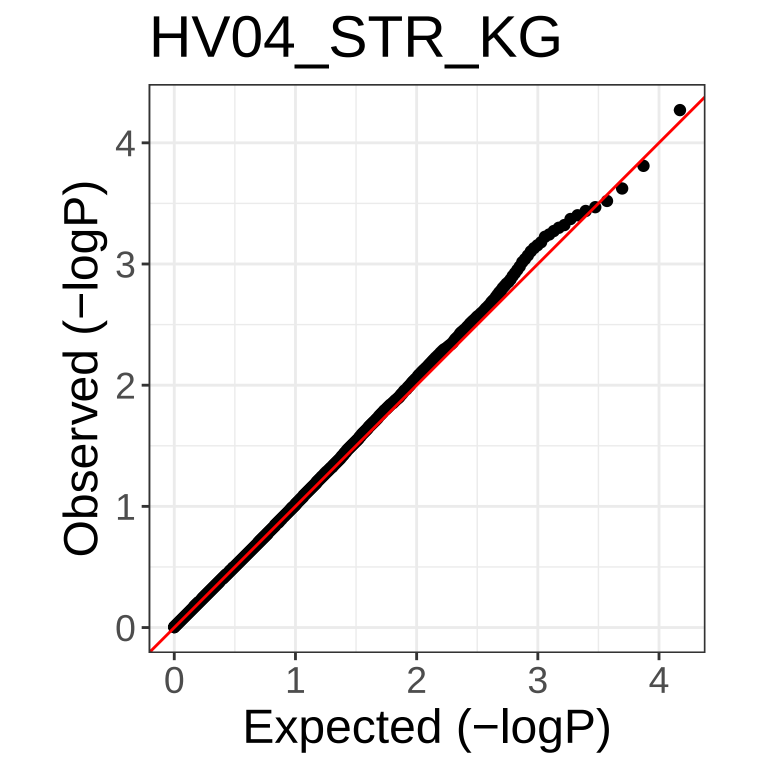
HV04_STR_KG
Observed (−logP)
Expected (−logP)
0
1
2
3
4
0
1
2
3
4

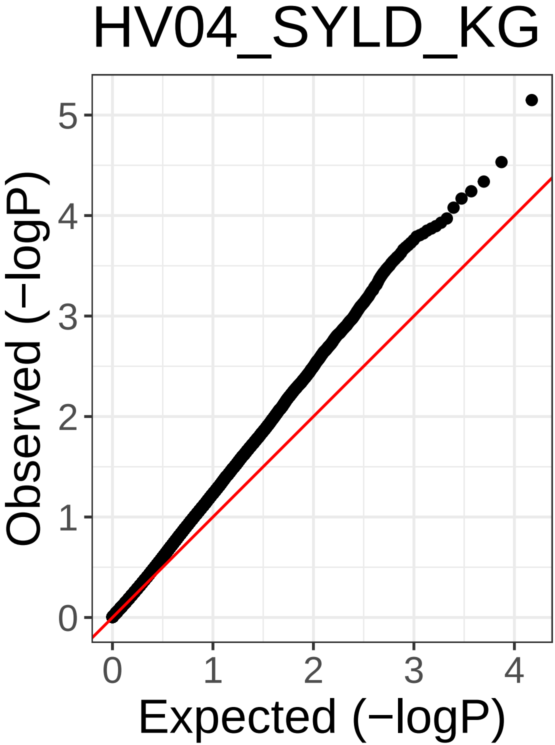
HV04_SYLD_KG
Observed (−logP)
Expected (−logP)
0
1
2
3
4
5
0
1
2
3
4

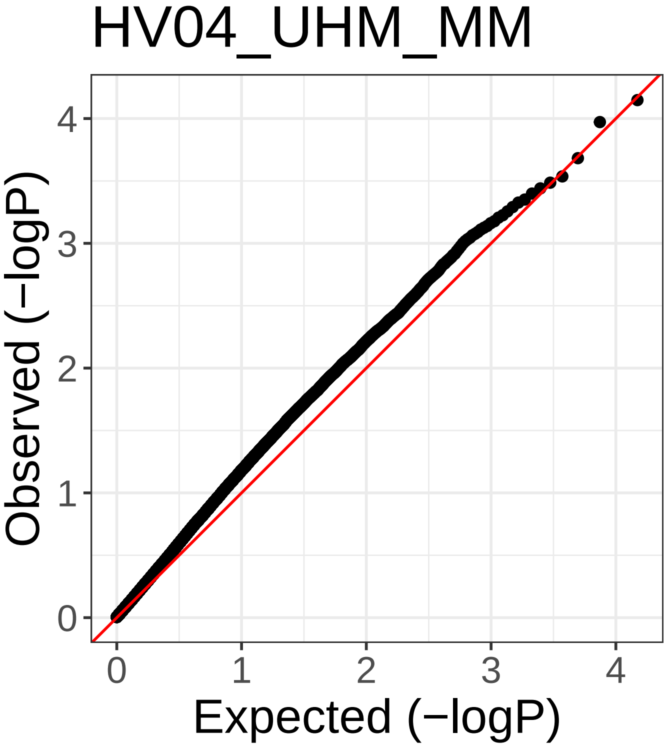
HV04_UHM_MM
Observed (−logP)
Expected (−logP)
0
1
2
3
4
0
1
2
3
4

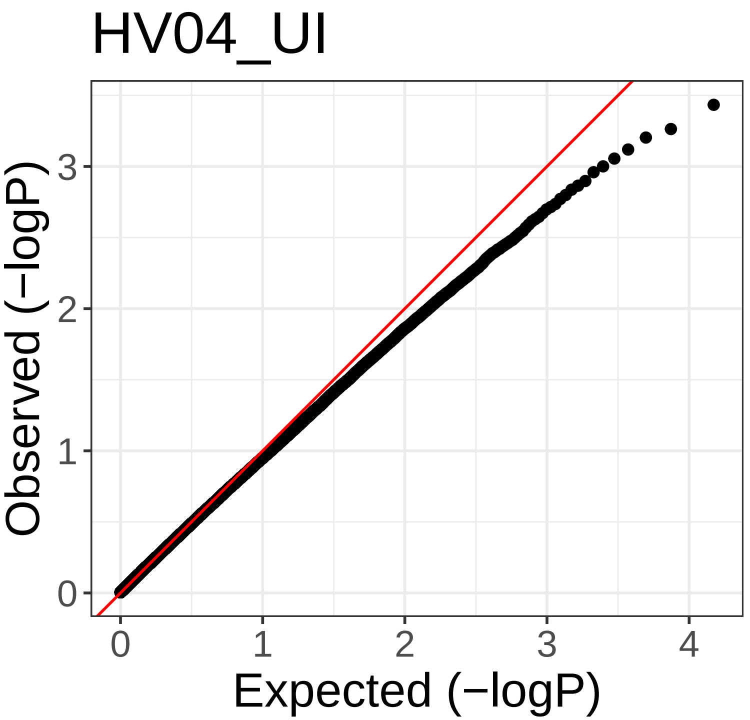
HV04_UI
Observed (−logP)
Expected (−logP)
0
1
2
3
4

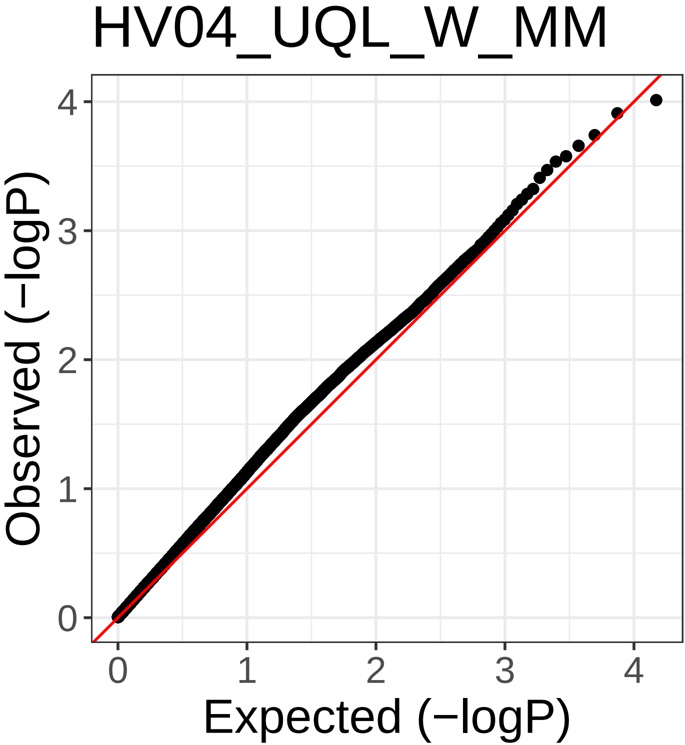

HV04_UQL_W_MM
Observed (−logP)
Expected (−logP)
0
1
2
3
4
0
1
2
3
4

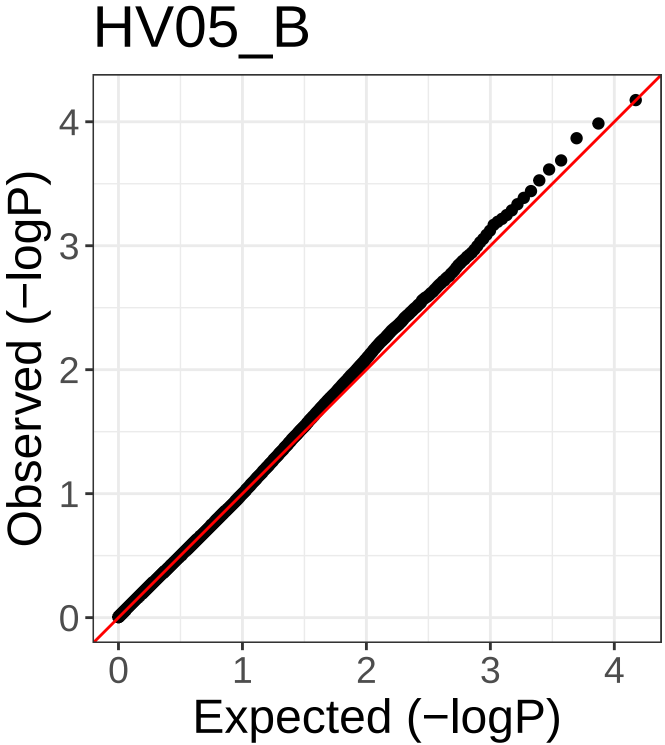

HV05_B
Observed (−logP)
Expected (−logP)
0
1
2
3
4
0
1
2
3
4

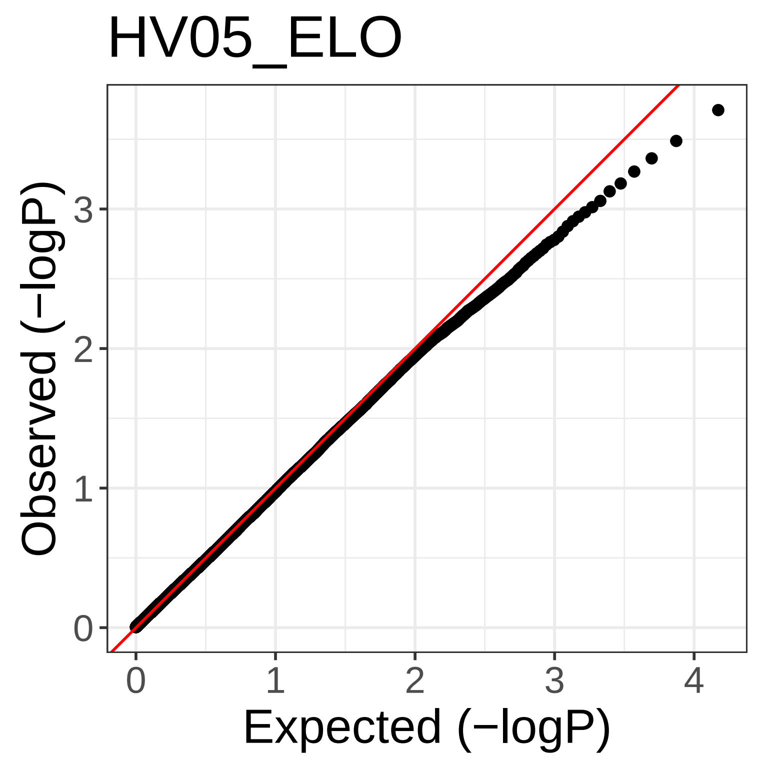
HV05_ELO
Observed (−logP)
Expected (−logP)
0
1
2
3
4

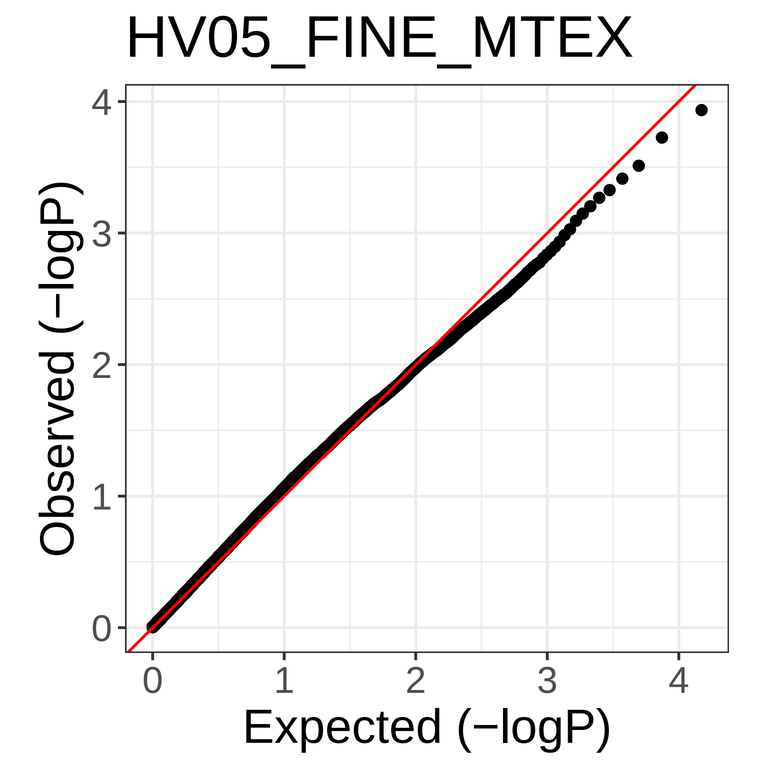
HV05_FINE_MTEX
Observed (−logP)
Expected (−logP)
0
1
2
3
4
0
1
2
3
4

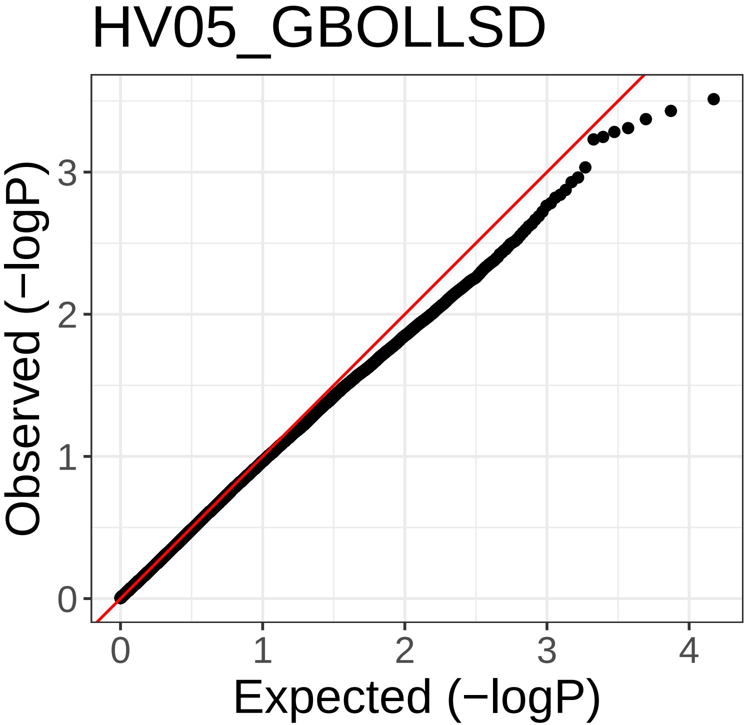
HV05_GBOLLSD
Observed (−logP)
Expected (−logP)
0
1
2
3
4
0
1
2
3

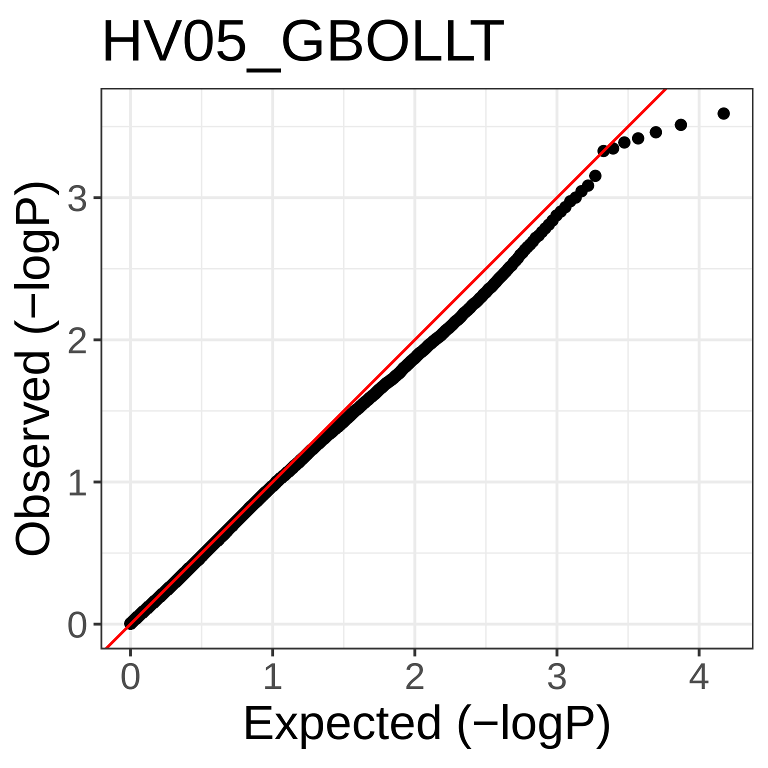

HV05_GBOLLT
Observed (−logP)
Expected (−logP)
0
1
2
3
4

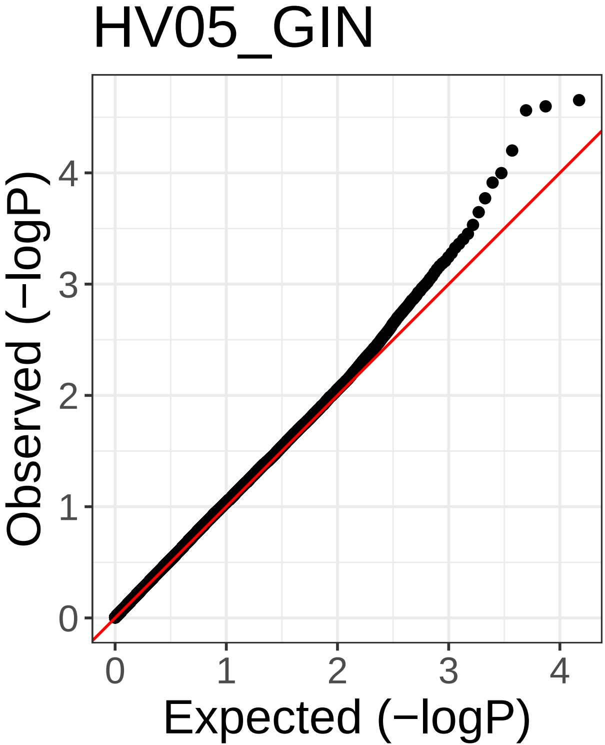
HV05_GIN
Observed (−logP)
Expected (−logP)
0
1
2
3
4

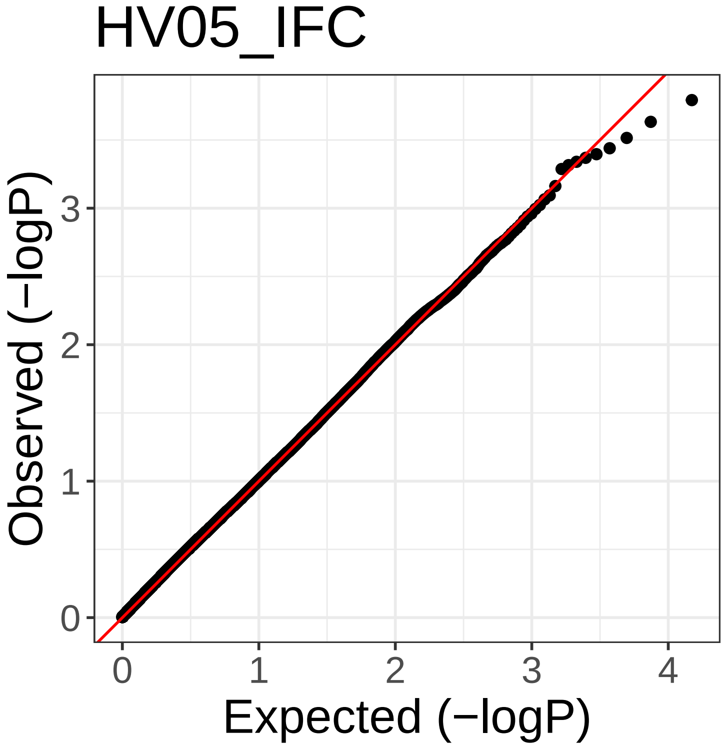
HV05_IFC
Observed (−logP)
Expected (−logP)
0
1
2
3
0
1
2
3
4

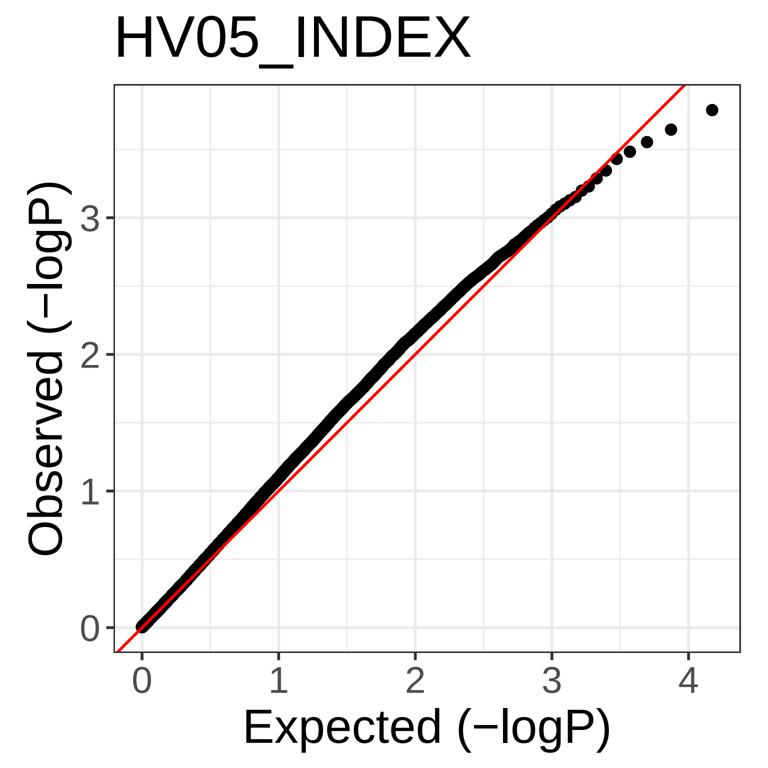
HV05_INDEX
Observed (−logP)
Expected (−logP)
0
1
2
3
4

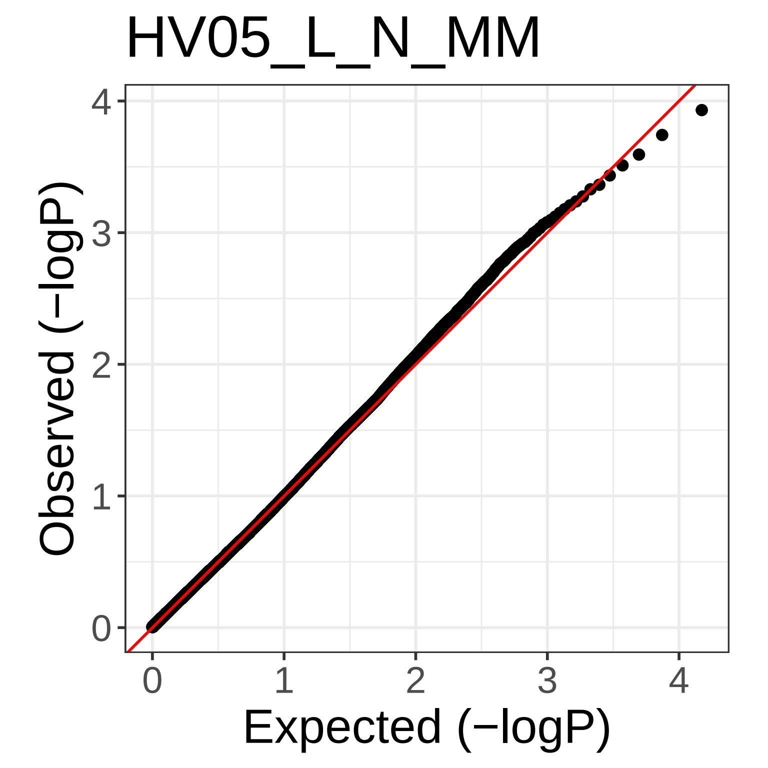

HV05_L_N_MM
Observed (−logP)
Expected (−logP)
0
1
2
3
4
0
1
2
3
4

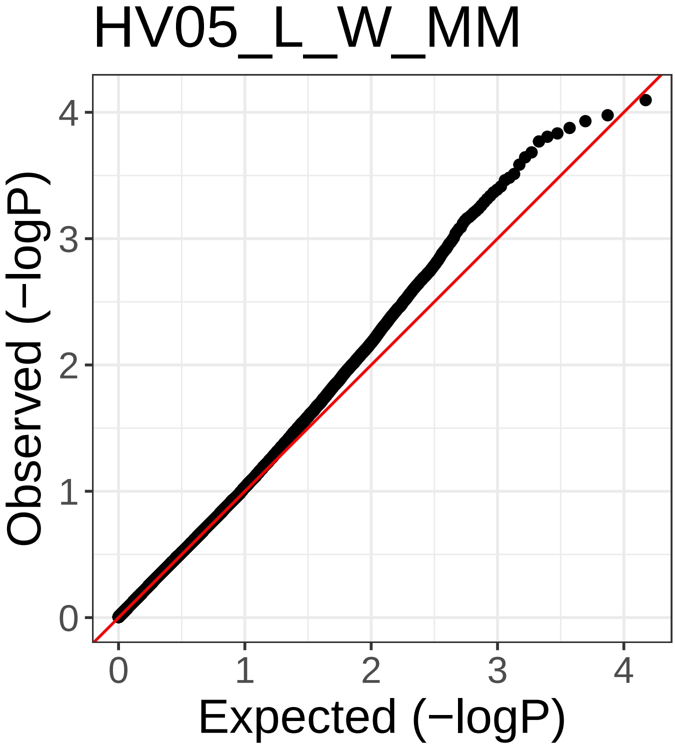

HV05_L_W_MM
Observed (−logP)
Expected (−logP)
0
1
2
3
4
0
1
2
3
4

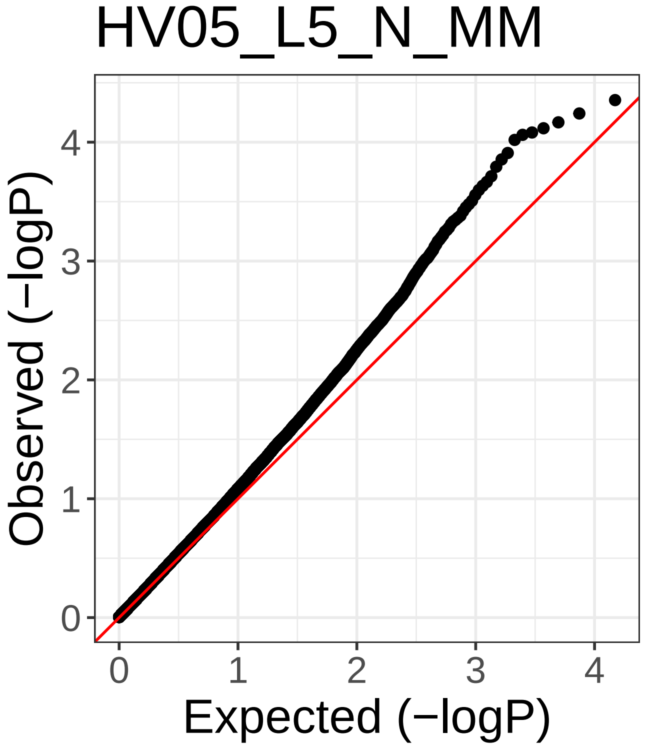
HV05_L5_N_MM
Observed (−logP)
Expected (−logP)
0
1
2
3
4

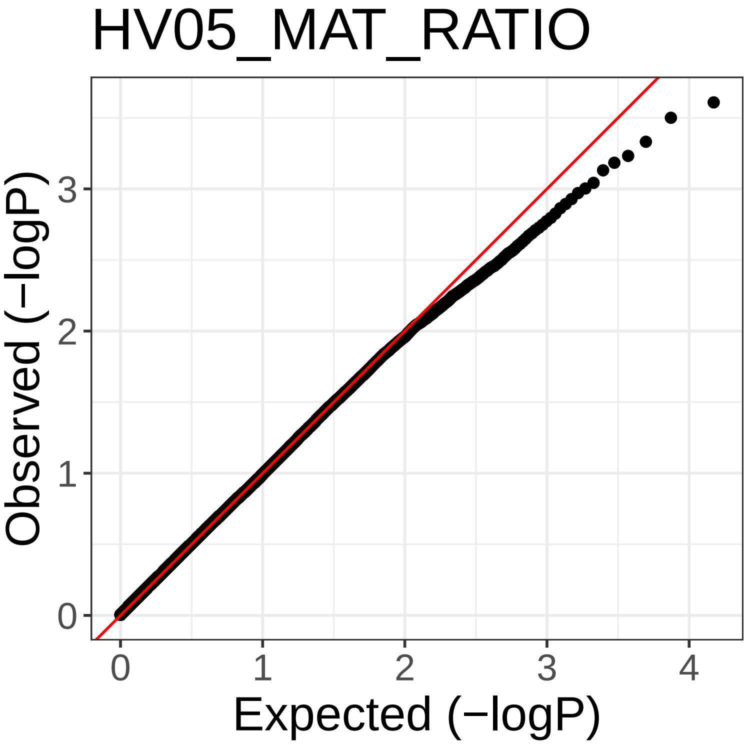
HV05_MAT_RATIO
Observed (−logP)
Expected (−logP)
0
1
2
3
4

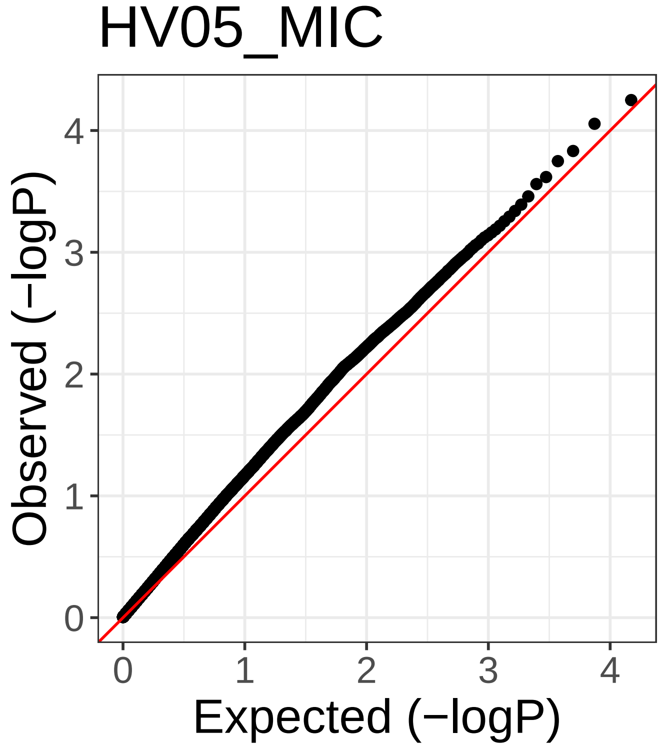
HV05_MIC
Observed (−logP)
Expected (−logP)
0
1
2
3
4

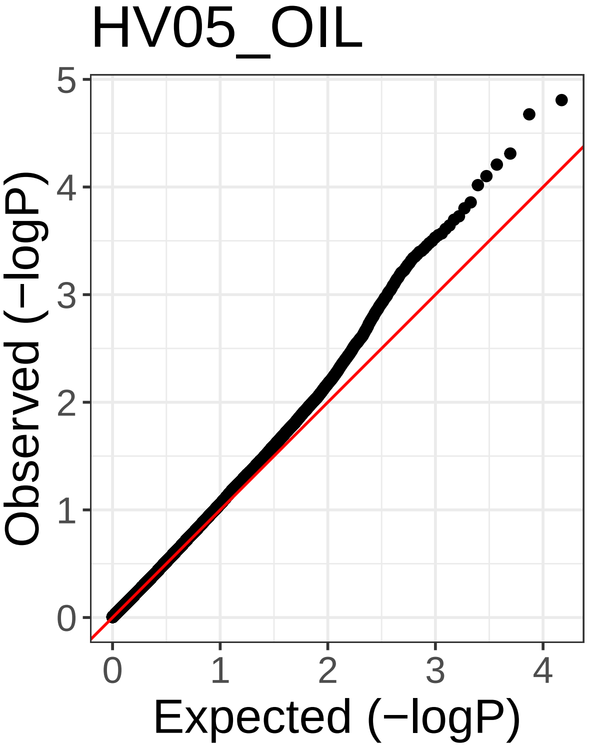
HV05_OIL
Observed (−logP)
Expected (−logP)
0
1
2
3
4
5
0
1
2
3
4

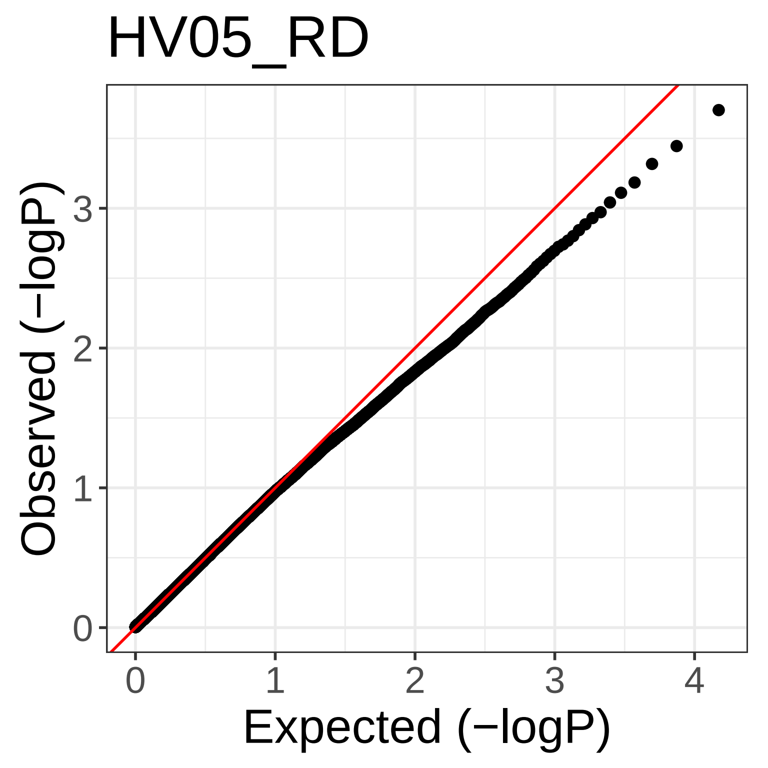

HV05_RD
Observed (−logP)
Expected (−logP)
0
1
2
3
4

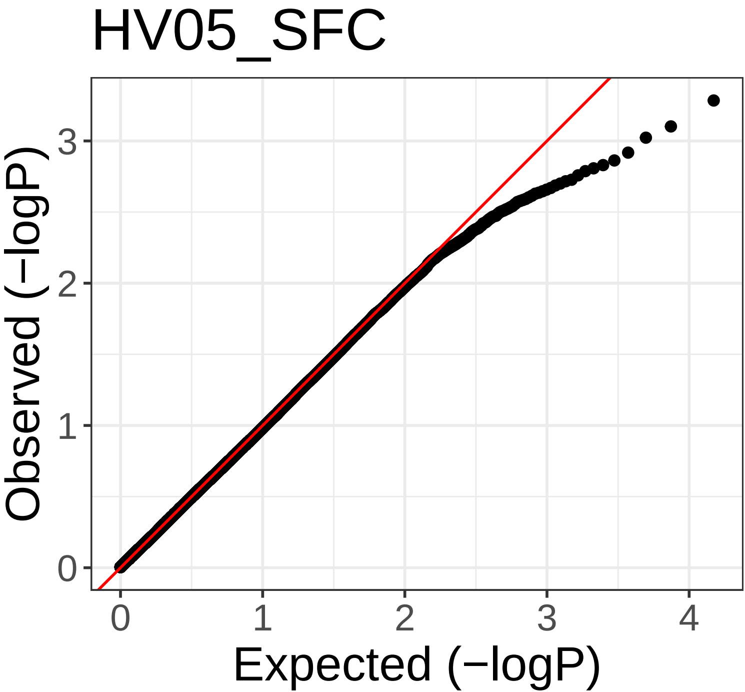

HV05_SFC
Observed (−logP)
Expected (−logP)
0
1
2
3
0
1
2
3
4

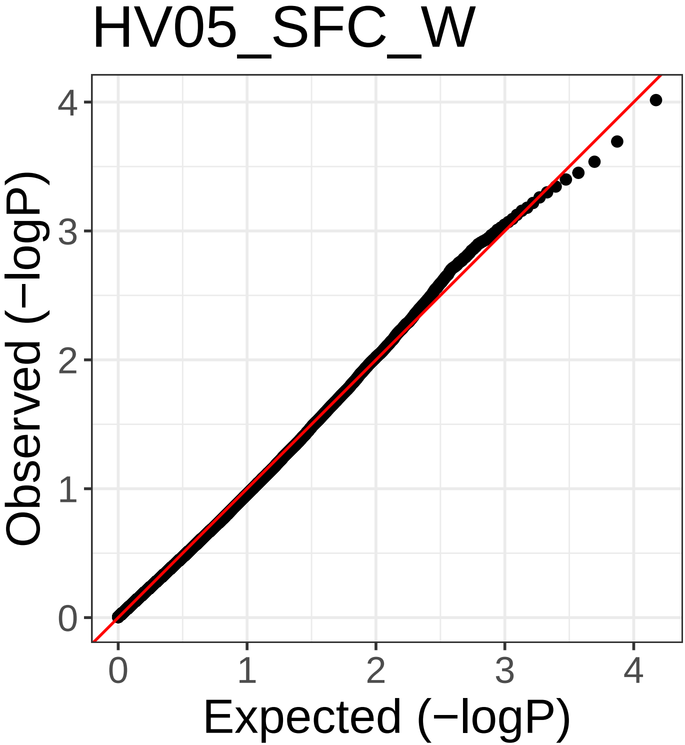

HV05_SFC_W
Observed (−logP)
Expected (−logP)
0
1
2
3
4

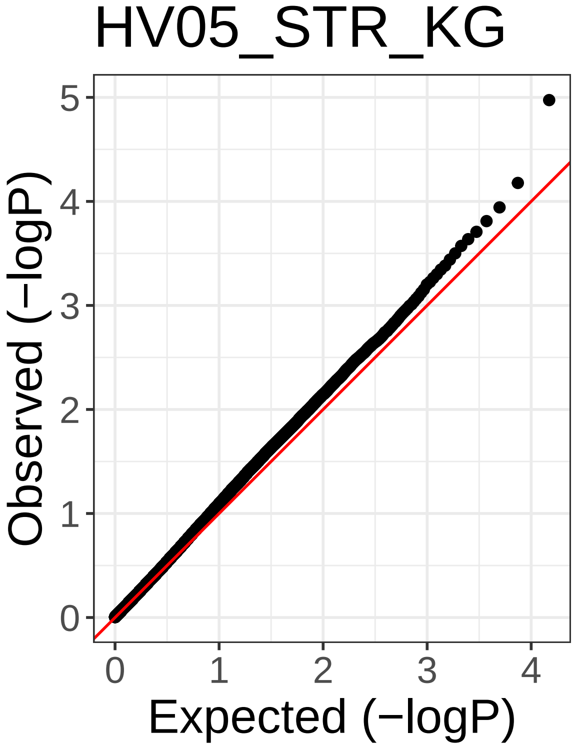

HV05_STR_KG
Observed (−logP)
Expected (−logP)
0
1
2
3
4
5
0
1
2
3
4

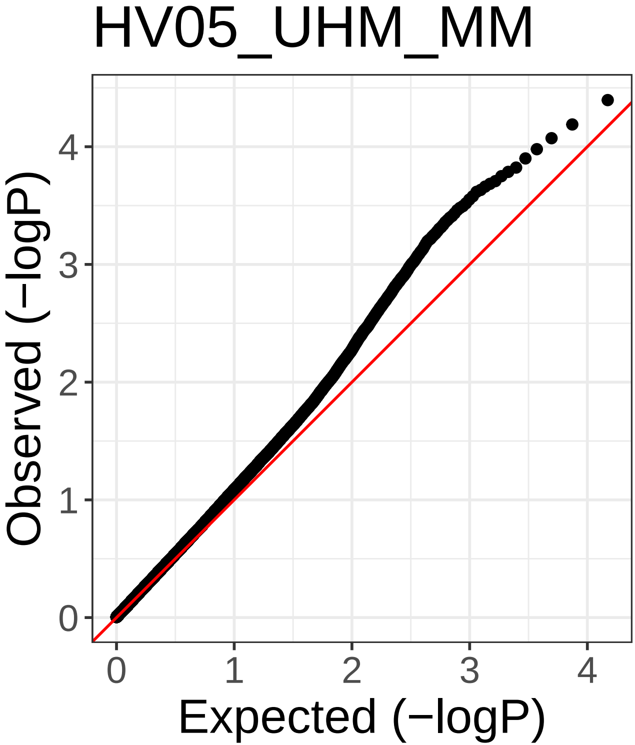
HV05_UHM_MM
Observed (−logP)
Expected (−logP)
0
1
2
3
4
0
1
2
3
4

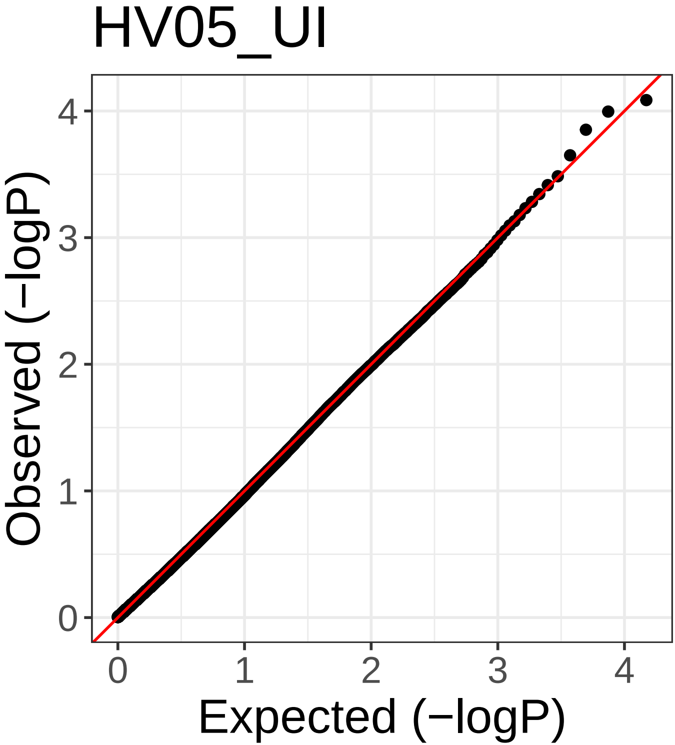

HV05_UI
Observed (−logP)
Expected (−logP)
0
1
2
3
4
0
1
2
3
4

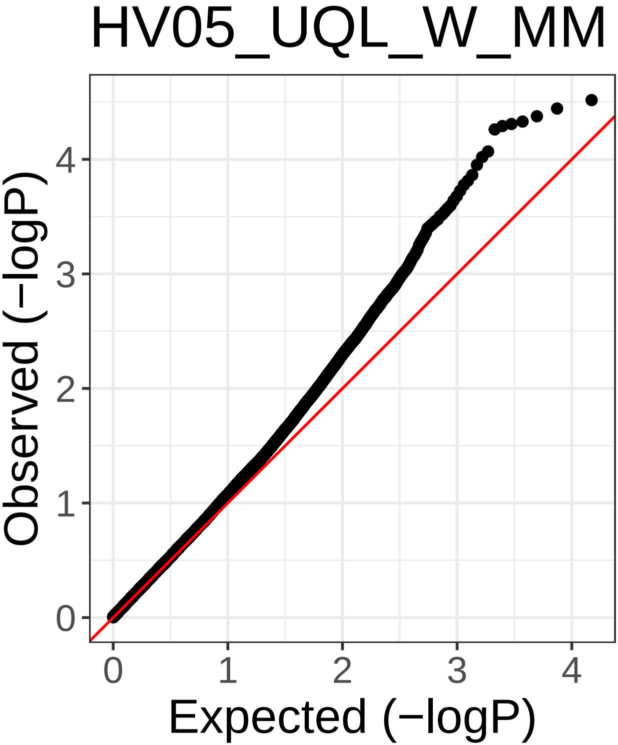
HV05_UQL_W_MM
Observed (−logP)
Expected (−logP)
0
1
2
3
4
0
1
2
3
4

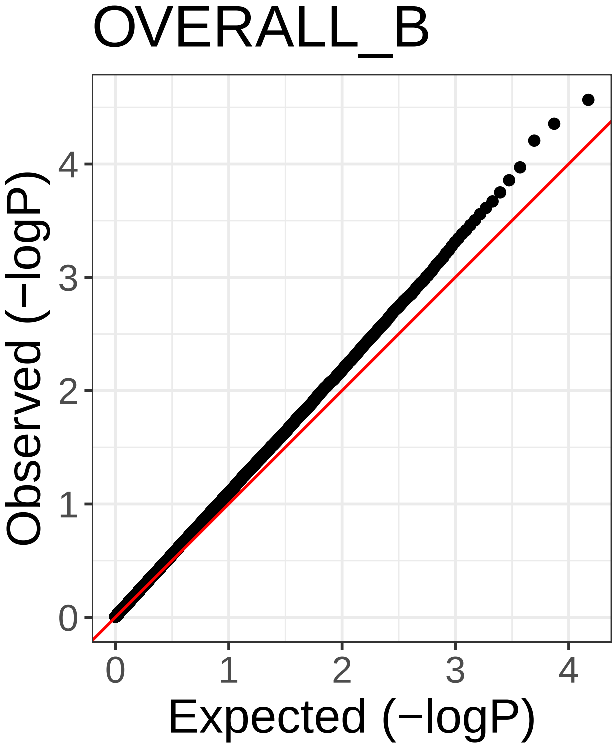
OVERALL_B
Observed (−logP)
Expected (−logP)
0
1
2
3
4
0
1
2
3
4

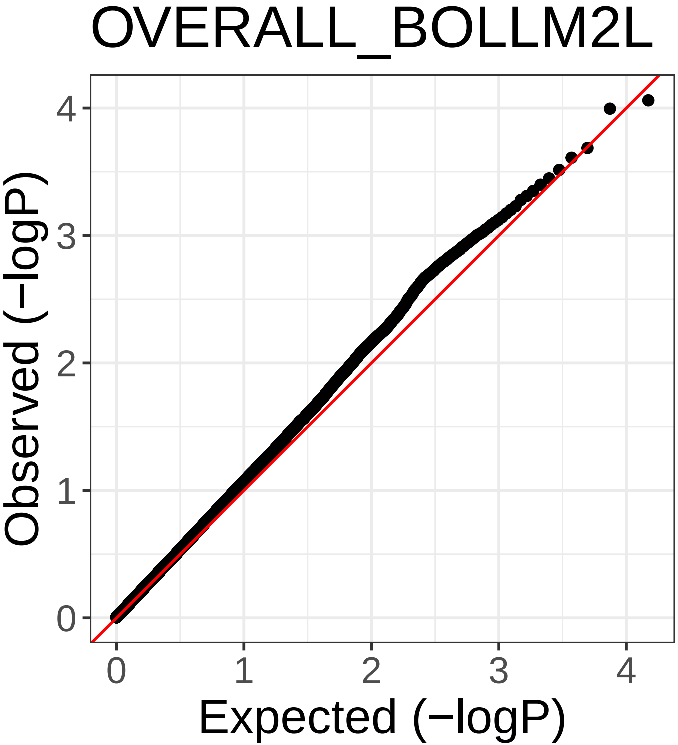

OVERALL_BOLLM2L
Observed (−logP)
Expected (−logP)
0
1
2
3
4
0
1
2
3
4

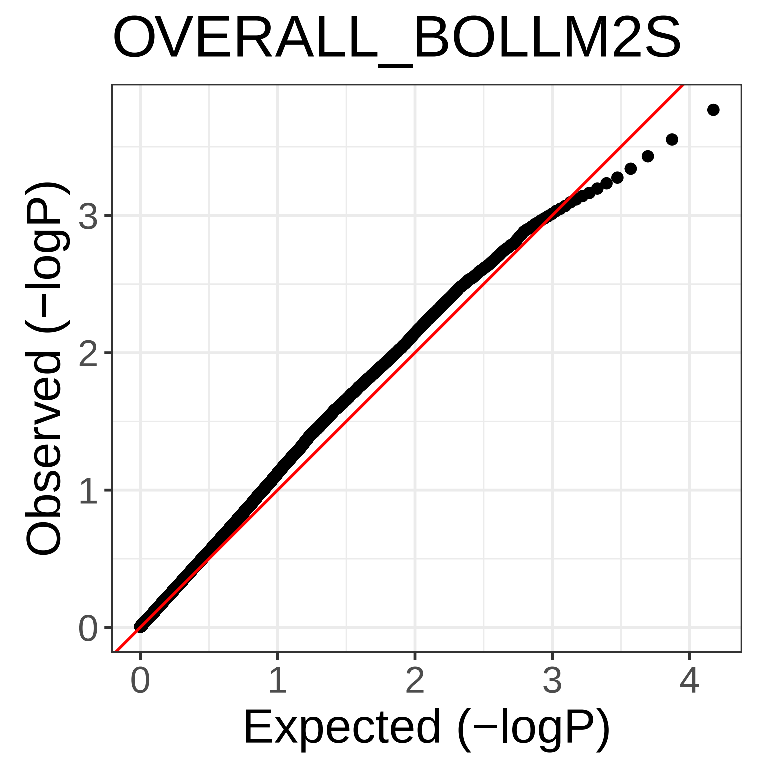
OVERALL_BOLLM2S
Observed (−logP)
Expected (−logP)
0
1
2
3
4

OVERALL_ELO

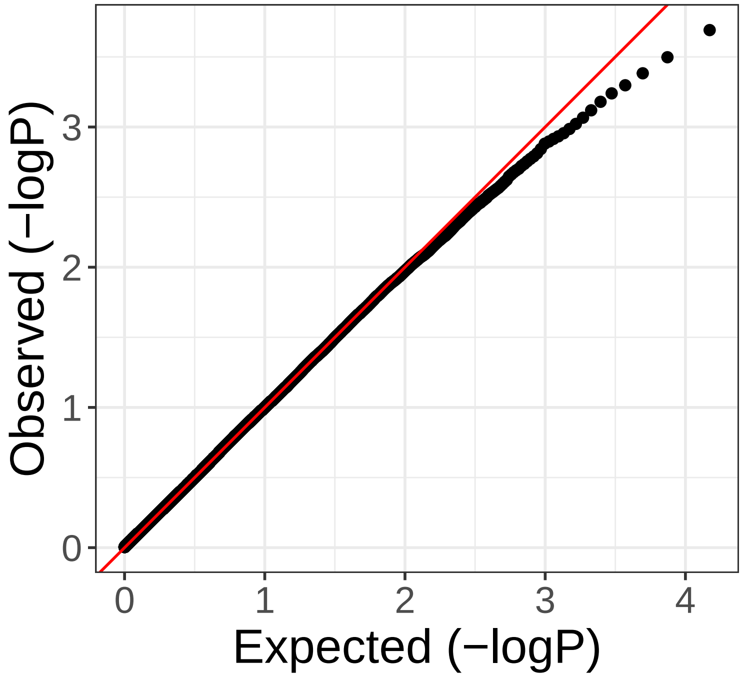

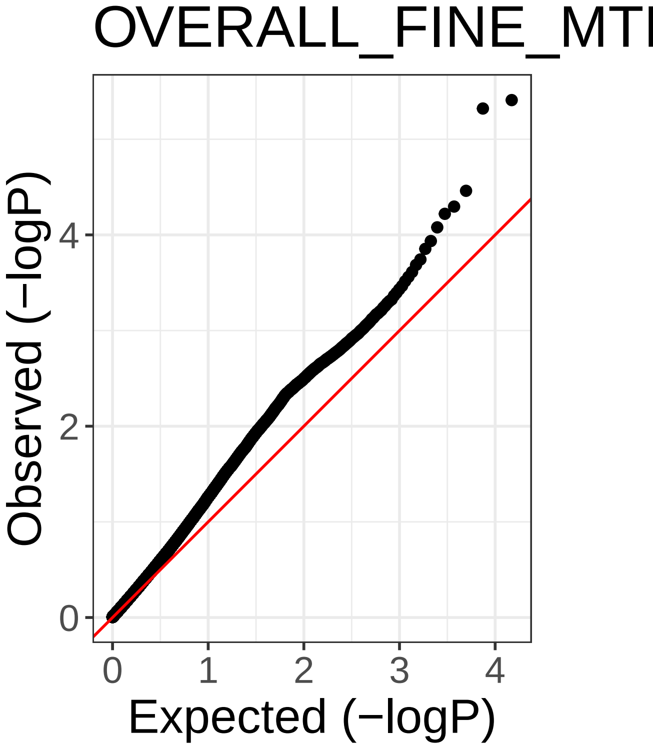

OVERALL_FINE_MTI
Observed (−logP)
Expected (−logP)
0
2
4
0
1
2
3
4

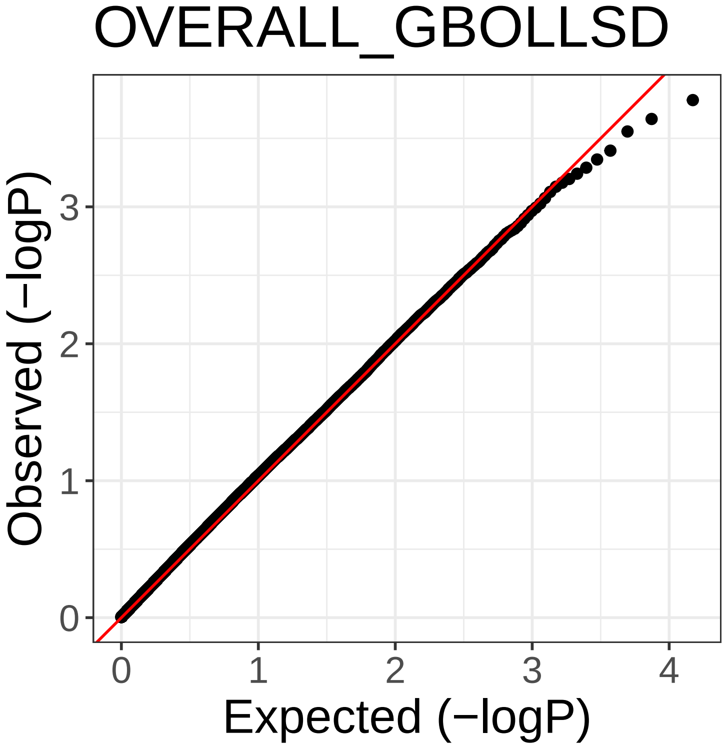

OVERALL_GBOLLSD
Observed (−logP)
Expected (−logP)
0
1
2
3
4

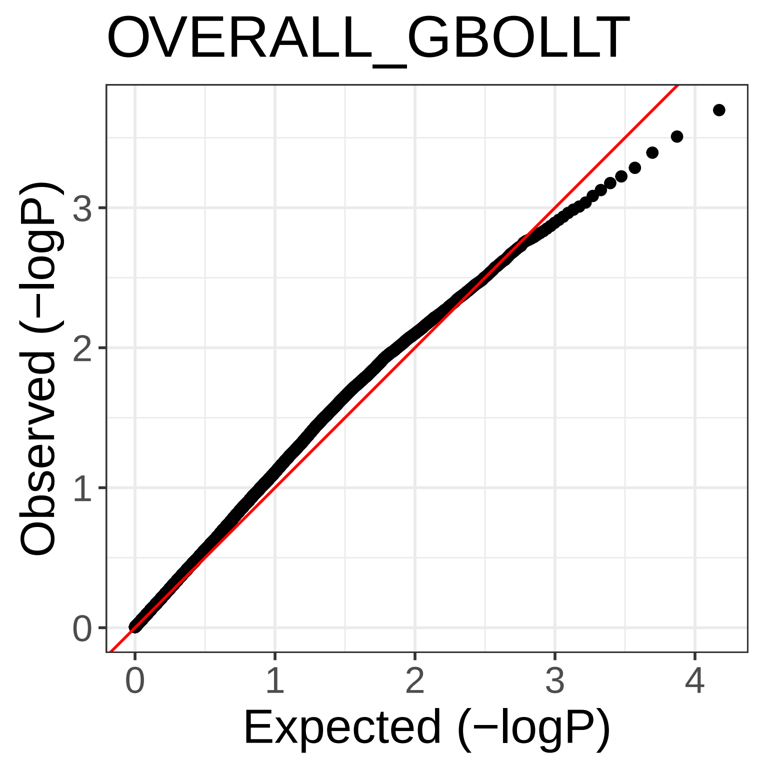
OVERALL_GBOLLT
Observed (−logP)
Expected (−logP)
0
1
2
3
4

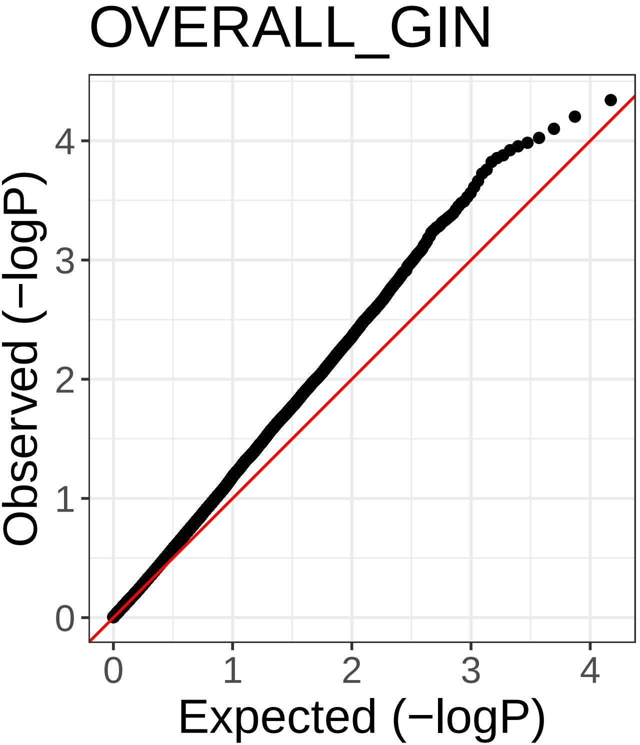
OVERALL_GIN
Observed (−logP)
Expected (−logP)
0
1
2
3
4

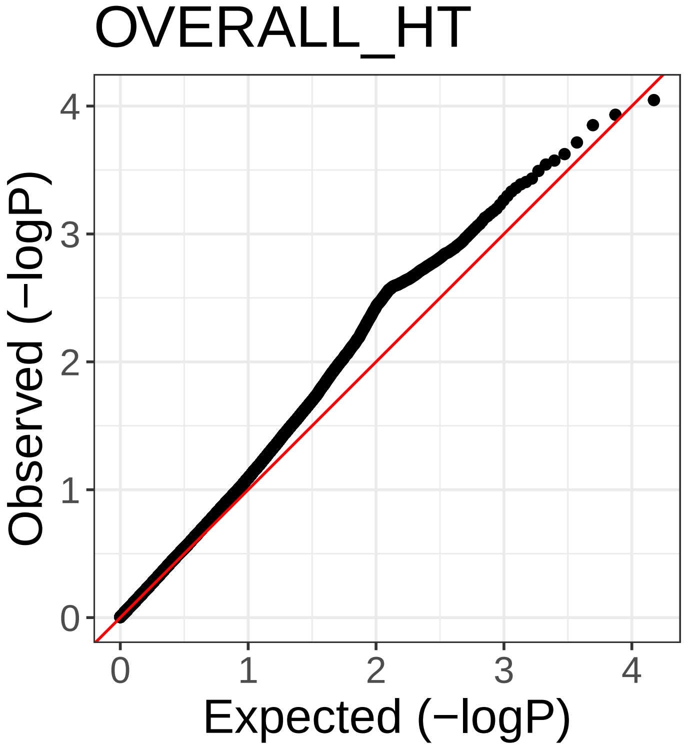
OVERALL_HT
Observed (−logP)
Expected (−logP)
0
1
2
3
4
0
1
2
3
4

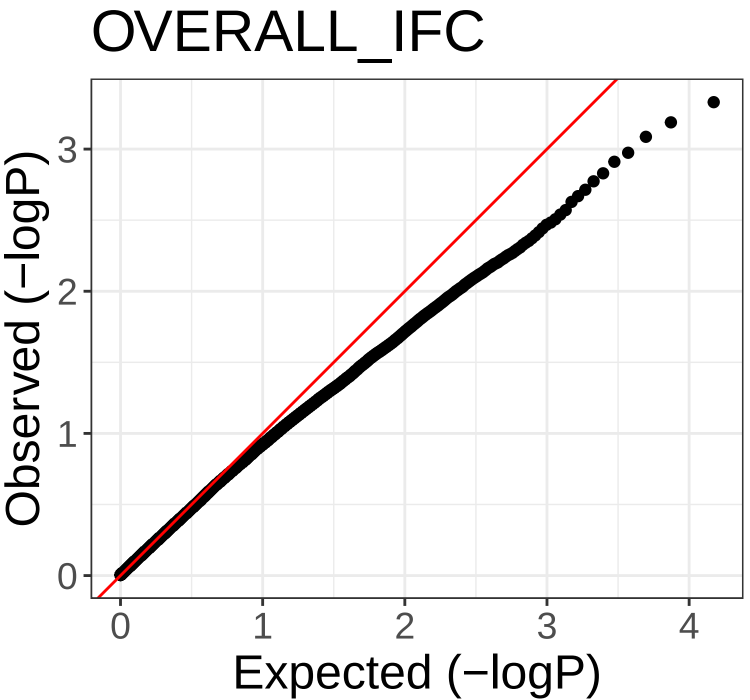
OVERALL_IFC
Observed (−logP)
Expected (−logP)
0
1
2
3
4

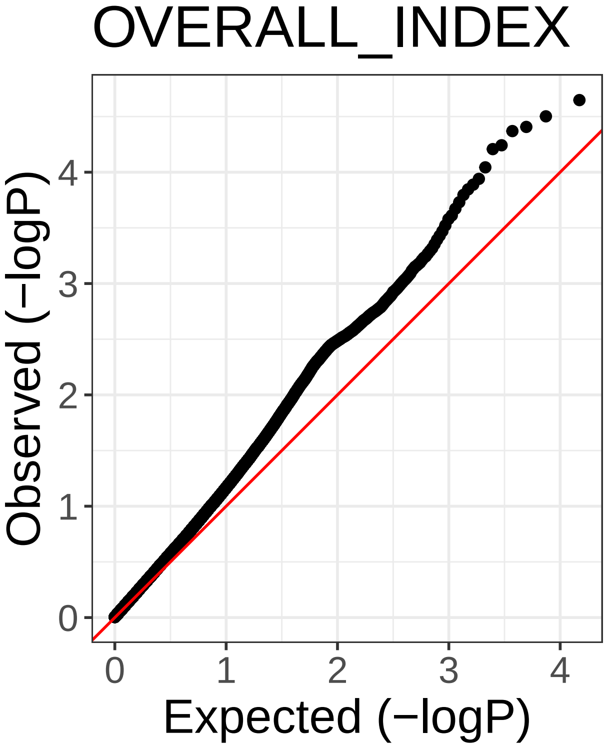

OVERALL_INDEX
Observed (−logP)
Expected (−logP)
0
1
2
3
4
0
1
2
3
4

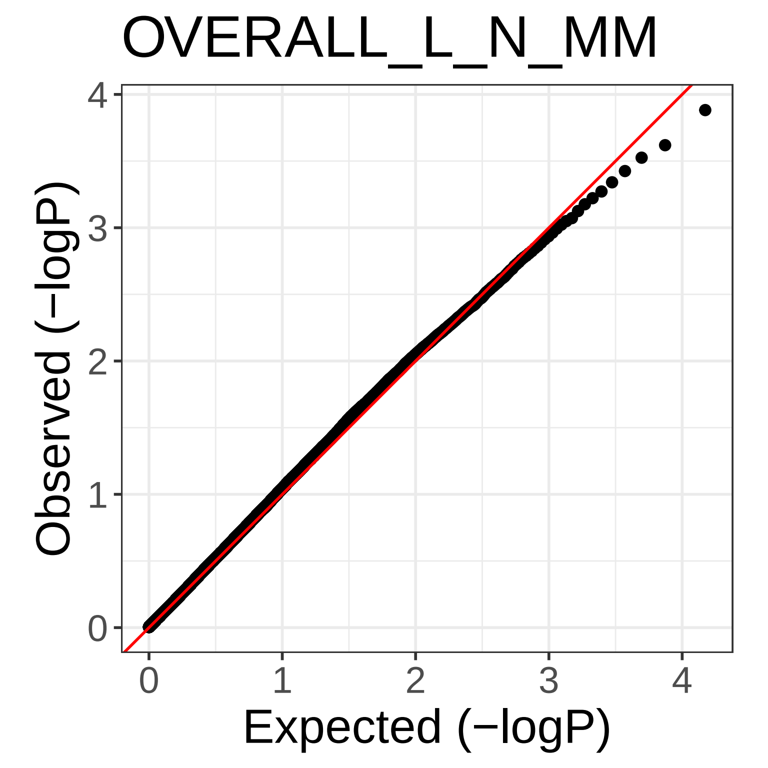
OVERALL_L_N_MM
Observed (−logP)
Expected (−logP)
0
1
2
3
4
0
1
2
3
4

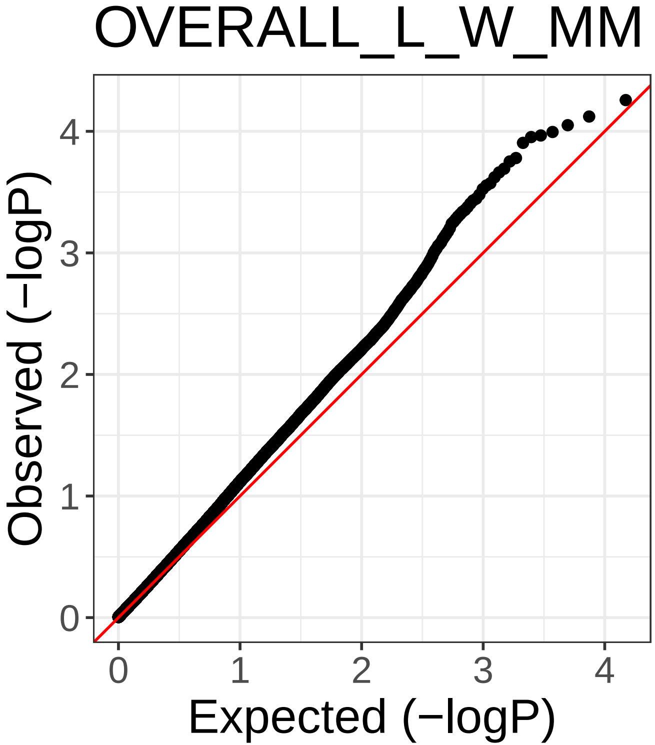
OVERALL_L_W_MM
Observed (−logP)
Expected (−logP)
0
1
2
3
4
0
1
2
3
4

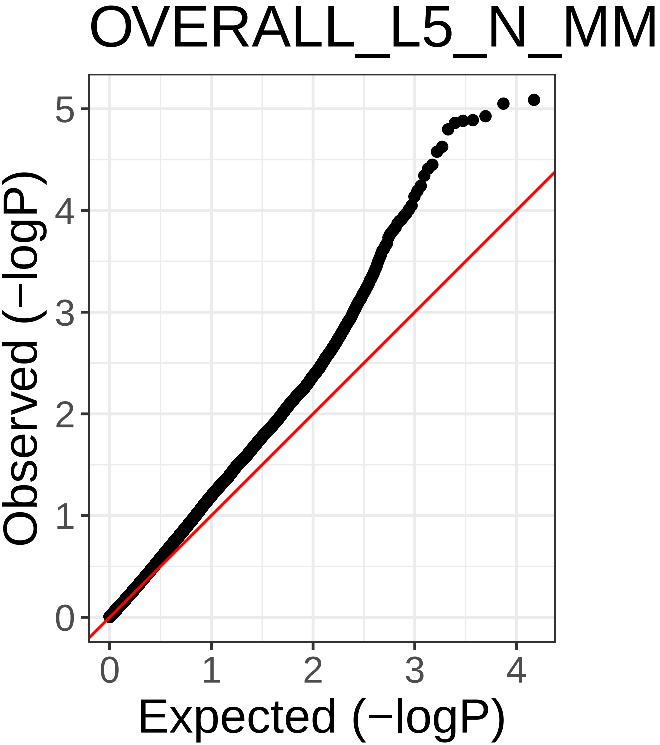

OVERALL_L5_N_MM
Observed (−logP)
Expected (−logP)
0
1
2
3
4
5
0
1
2
3
4

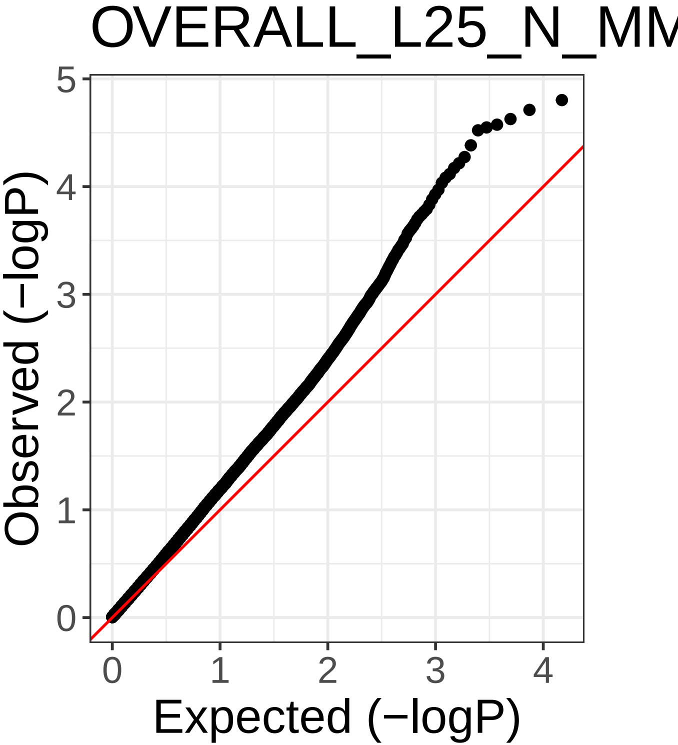

OVERALL_L25_N_MM
Observed (−logP)
Expected (−logP)
0
1
2
3
4
5

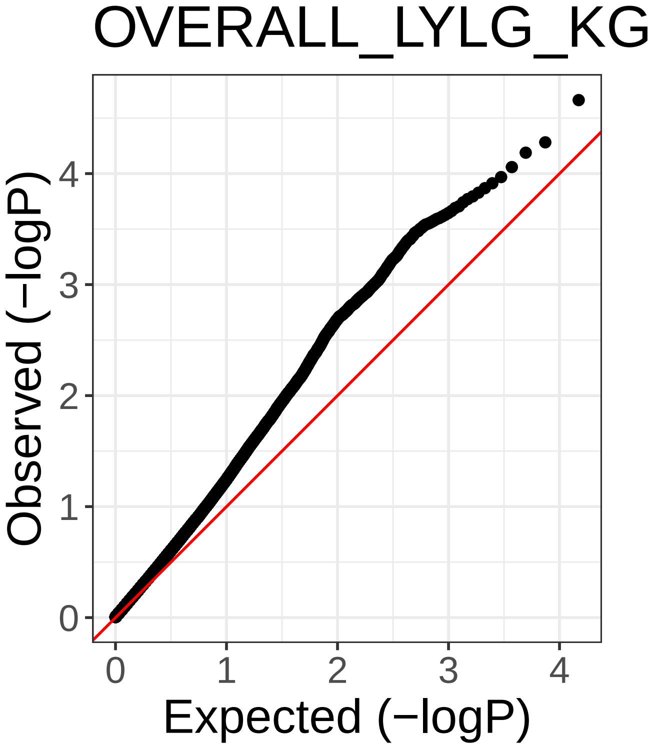
OVERALL_LYLG_KG
Observed (−logP)
Expected (−logP)
0
1
2
3
4
0
1
2
3
4

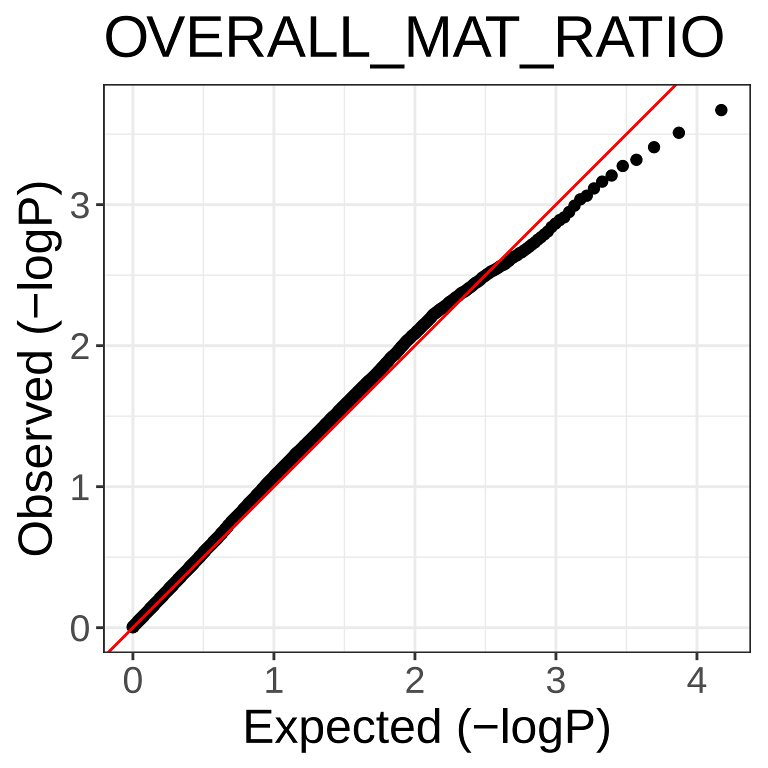
OVERALL_MAT_RATIO
Observed (−logP)
Expected (−logP)
0
1
2
3
4

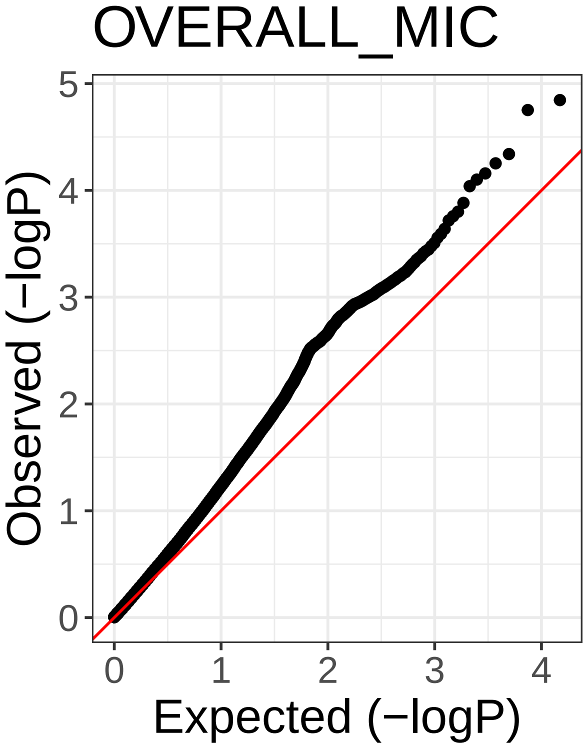
OVERALL_MIC
Observed (−logP)
Expected (−logP)
0
1
2
3
4
5

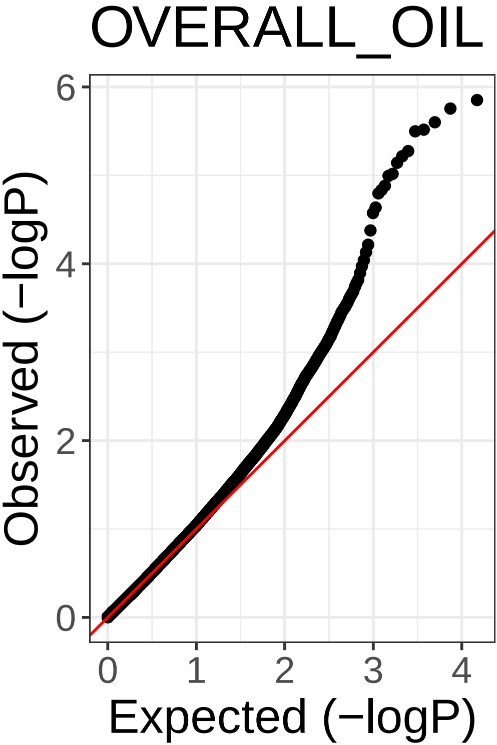

OVERALL_OIL
Observed (−logP)
Expected (−logP)
0
2
4
6
0
1
2
3
4

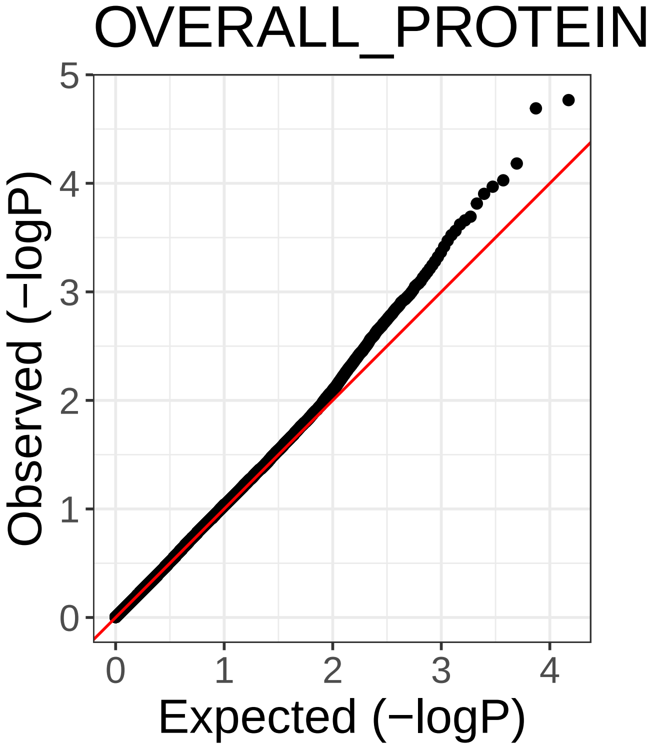
OVERALL_PROTEIN
Observed (−logP)
Expected (−logP)
0
1
2
3
4
5
0
1
2
3
4

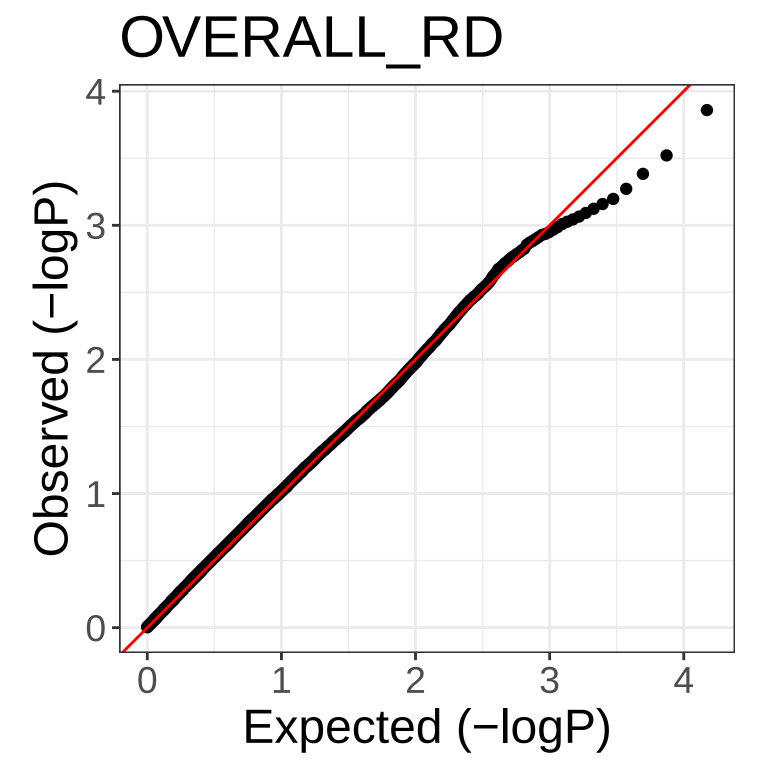
OVERALL_RD
Observed (−logP)
Expected (−logP)
0
1
2
3
4
0
1
2
3
4

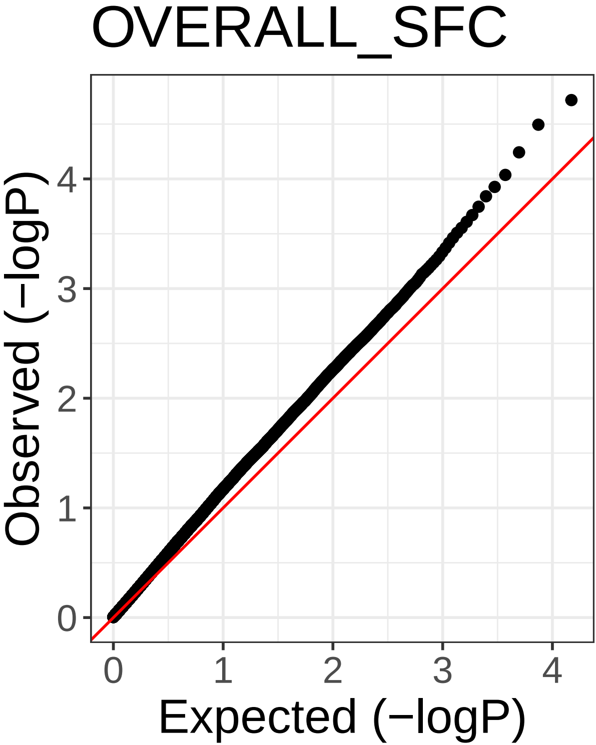

OVERALL_SFC
Observed (−logP)
Expected (−logP)
0
1
2
3
4
0
1
2
3
4

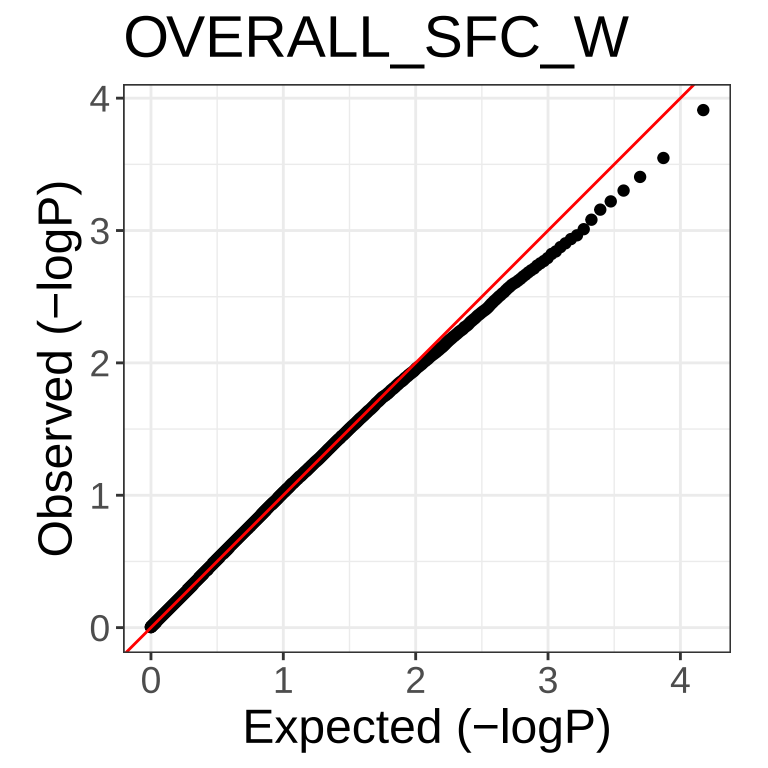
OVERALL_SFC_W
Observed (−logP)
Expected (−logP)
0
1
2
3
4
0
1
2
3
4

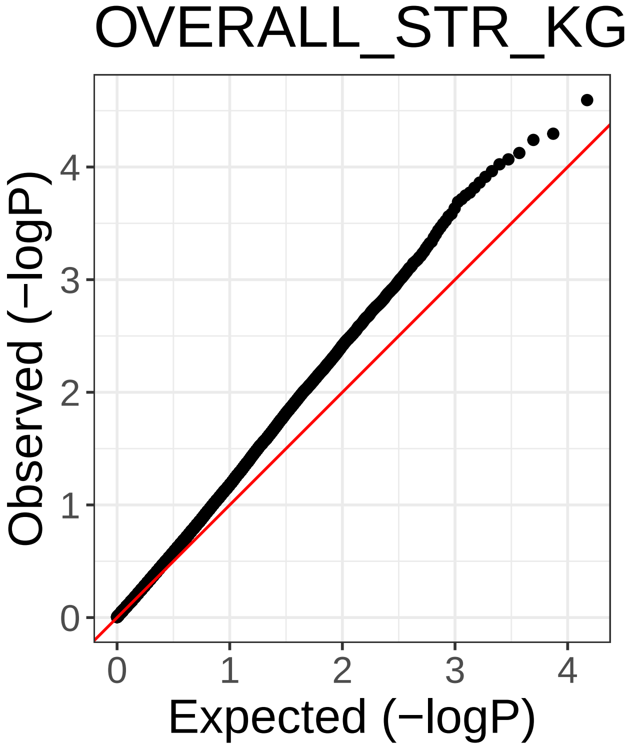

OVERALL_STR_KG
Observed (−logP)
Expected (−logP)
0
1
2
3
4
0
1
2
3
4

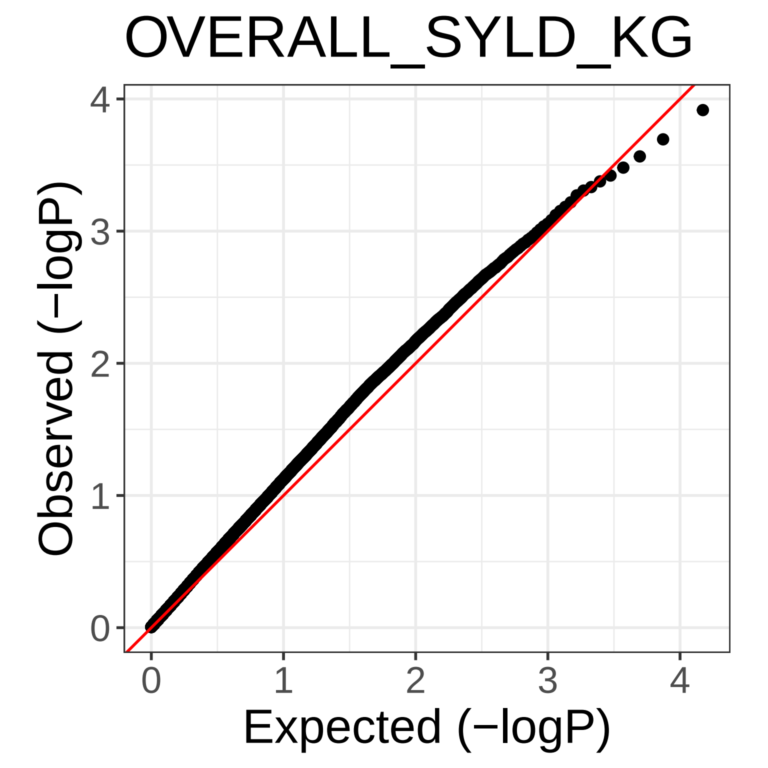
OVERALL_SYLD_KG
Observed (−logP)
Expected (−logP)
0
1
2
3
4
0
1
2
3
4

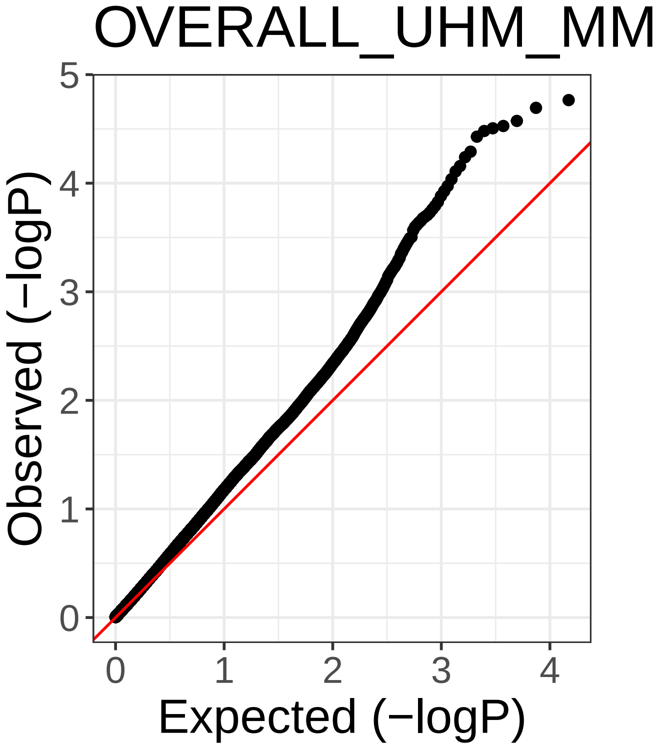
OVERALL_UHM_MM
Observed (−logP)
Expected (−logP)
0
1
2
3
4
5

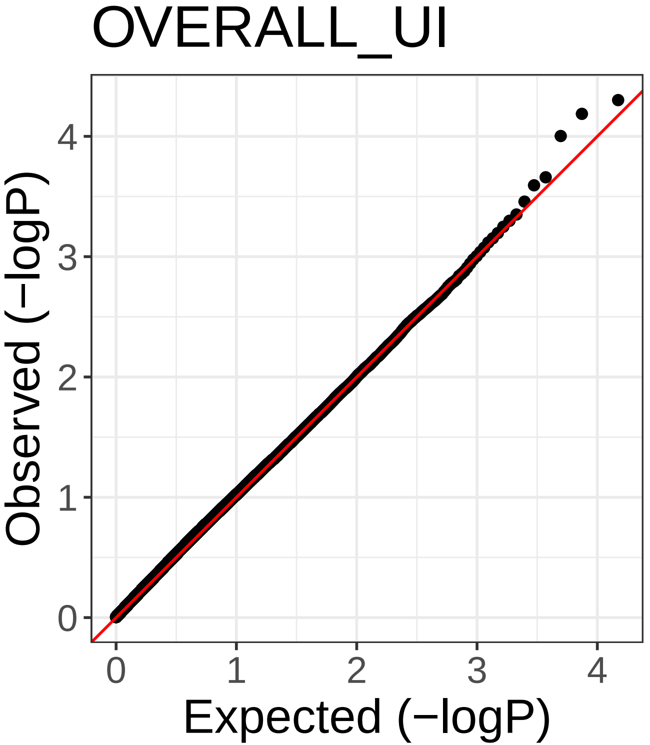

OVERALL_UI
Observed (−logP)
Expected (−logP)
0
1
2
3
4

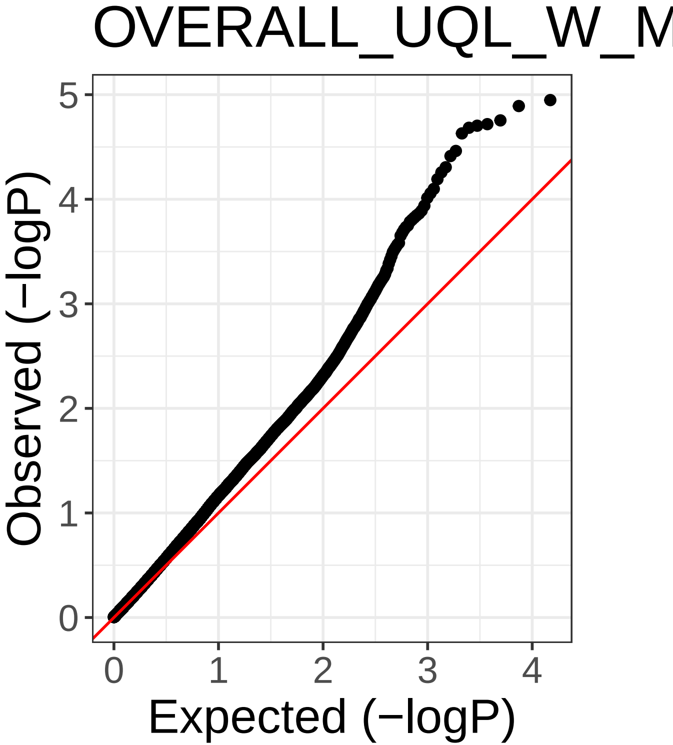
OVERALL_UQL_W_M
Observed (−logP)
5
4
3
2
1
0
0
1
2
3
4
Expected (−logP)

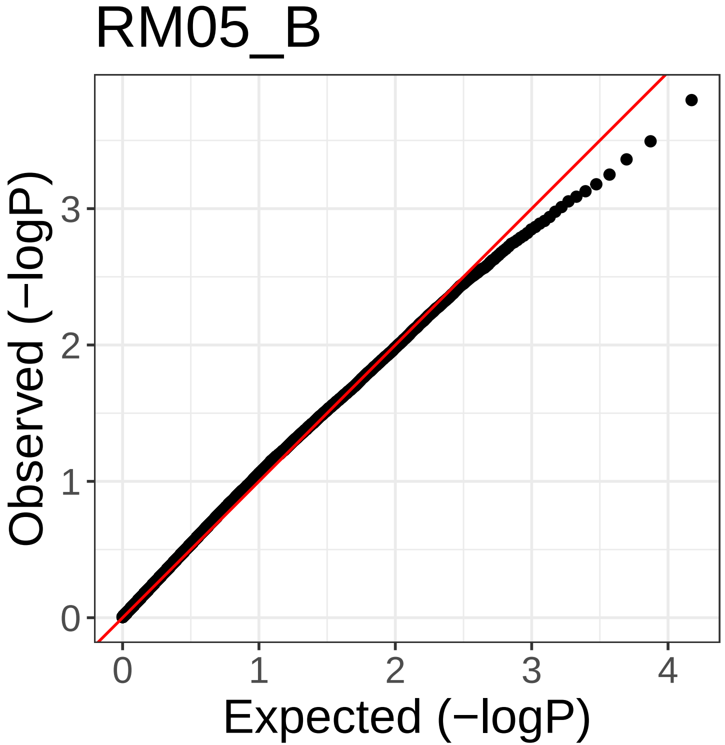
RM05_B
Observed (−logP)
Expected (−logP)
0
1
2
3
0
1
2
3
4

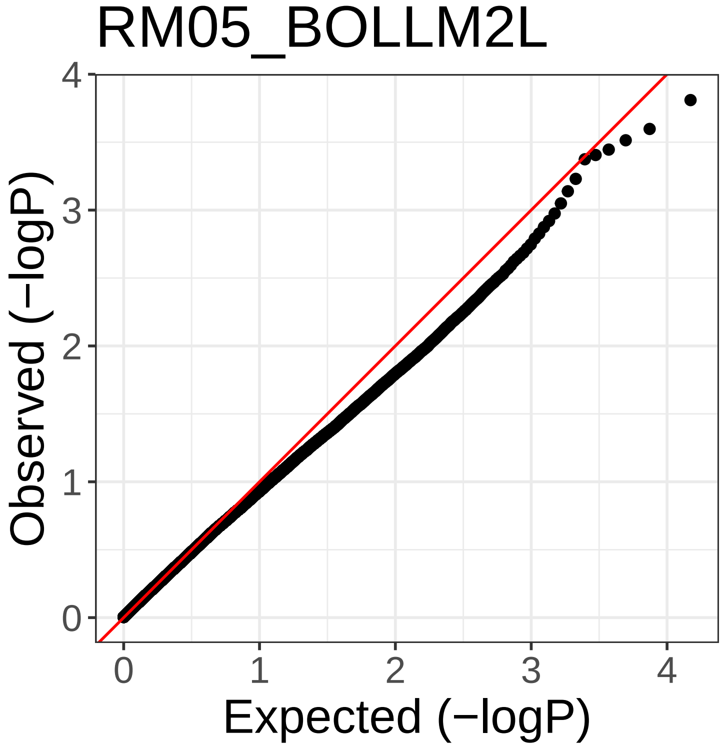
RM05_BOLLM2L
Observed (−logP)
Expected (−logP)
0
1
2
3
4
0
1
2
3
4

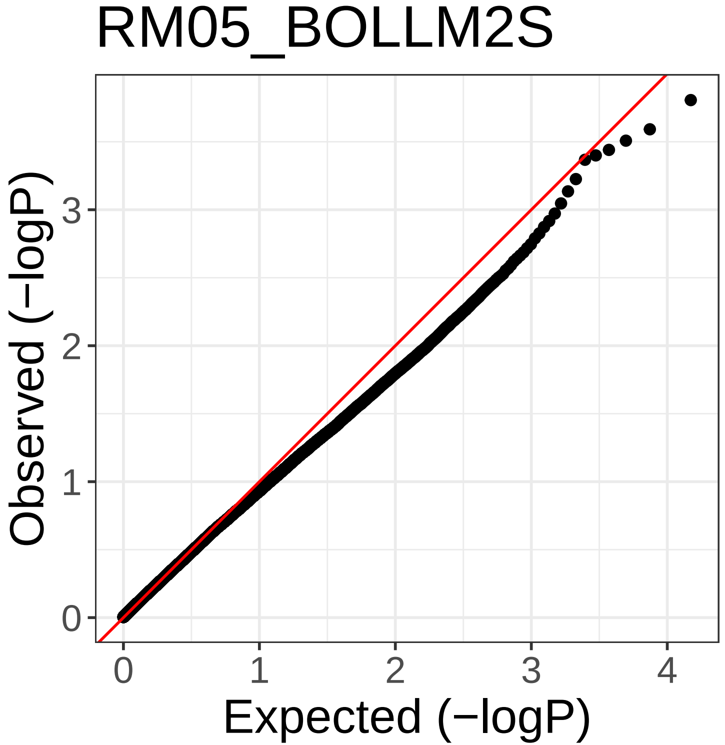
RM05_BOLLM2S
Observed (−logP)
Expected (−logP)
0
1
2
3
4

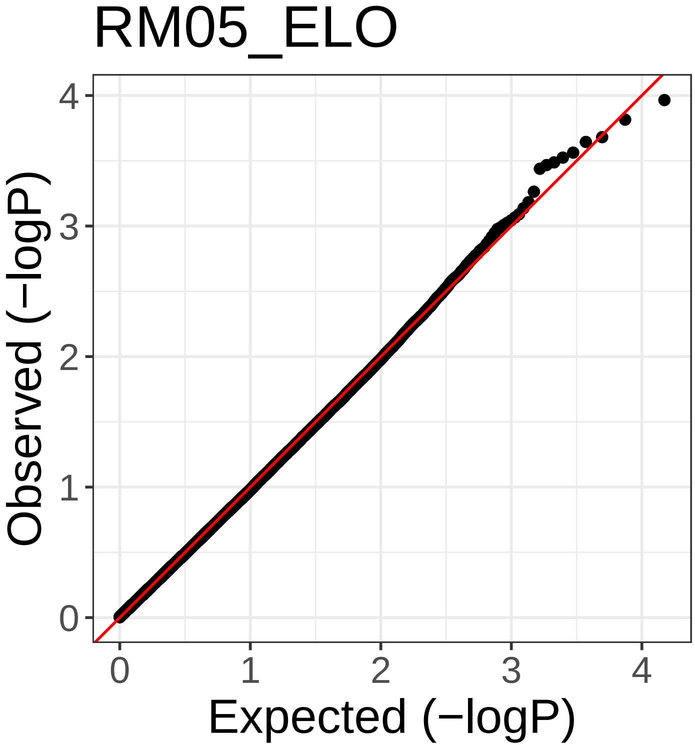
RM05_ELO
Observed (−logP)
Expected (−logP)
0
1
2
3
4
0
1
2
3
4

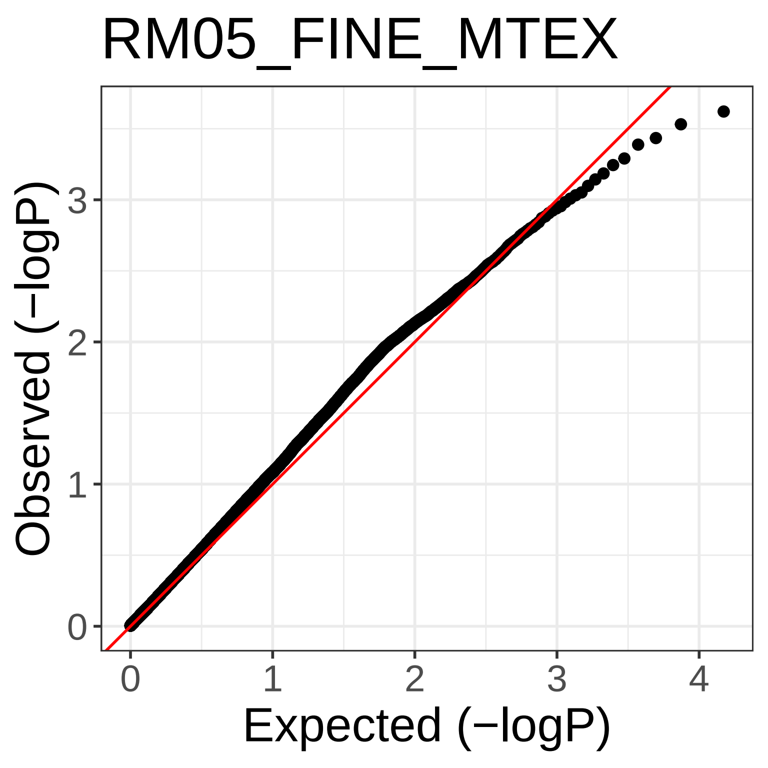

RM05_FINE_MTEX
Observed (−logP)
Expected (−logP)
0
1
2
3
4

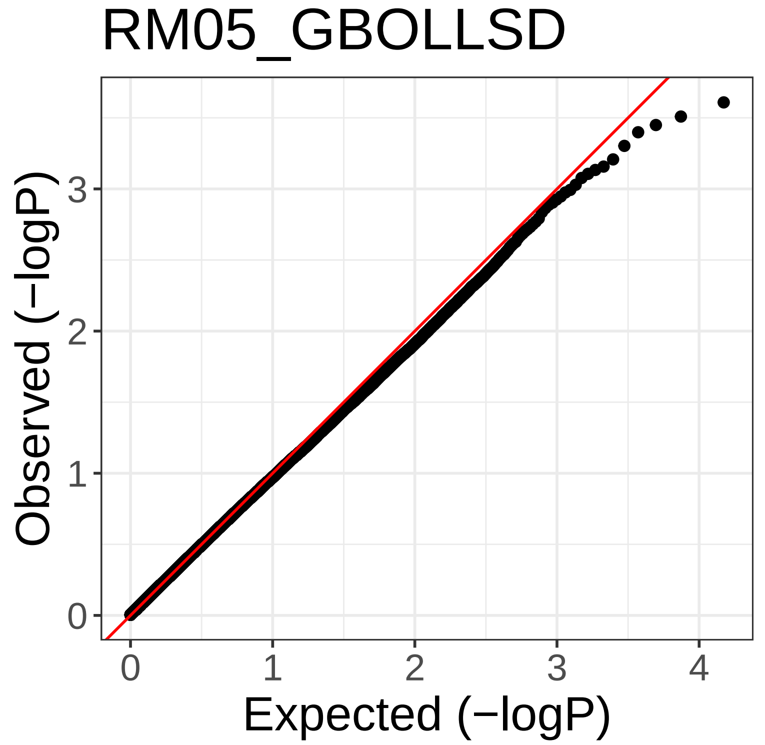
RM05_GBOLLSD
Observed (−logP)
Expected (−logP)
0
1
2
3
0
1
2
3
4

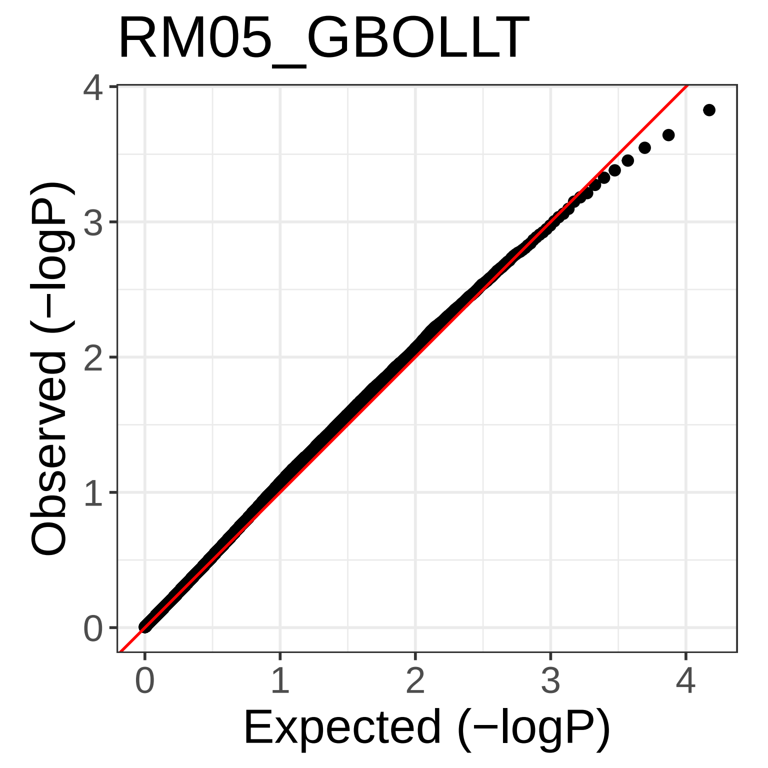
RM05_GBOLLT
Observed (−logP)
Expected (−logP)
0
1
2
3
4
0
1
2
3
4

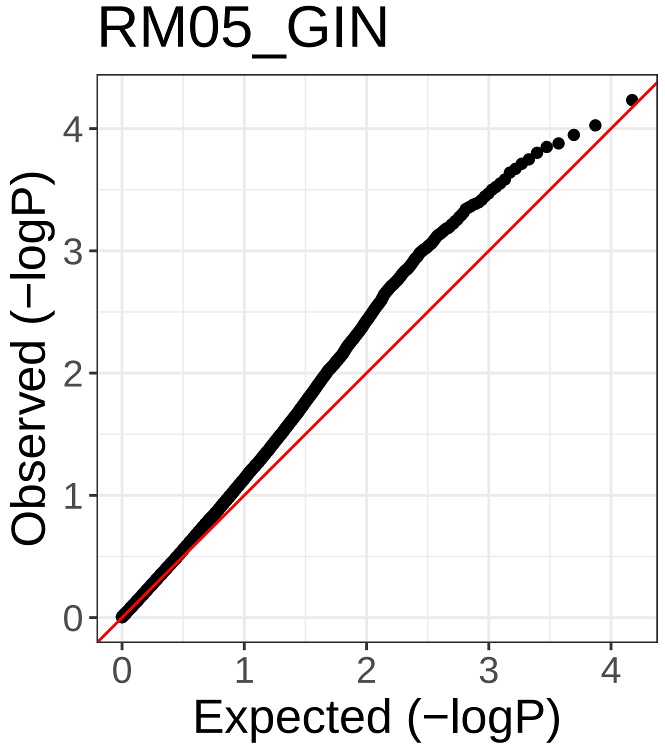

RM05_GIN
Observed (−logP)
Expected (−logP)
0
1
2
3
4
0
1
2
3
4

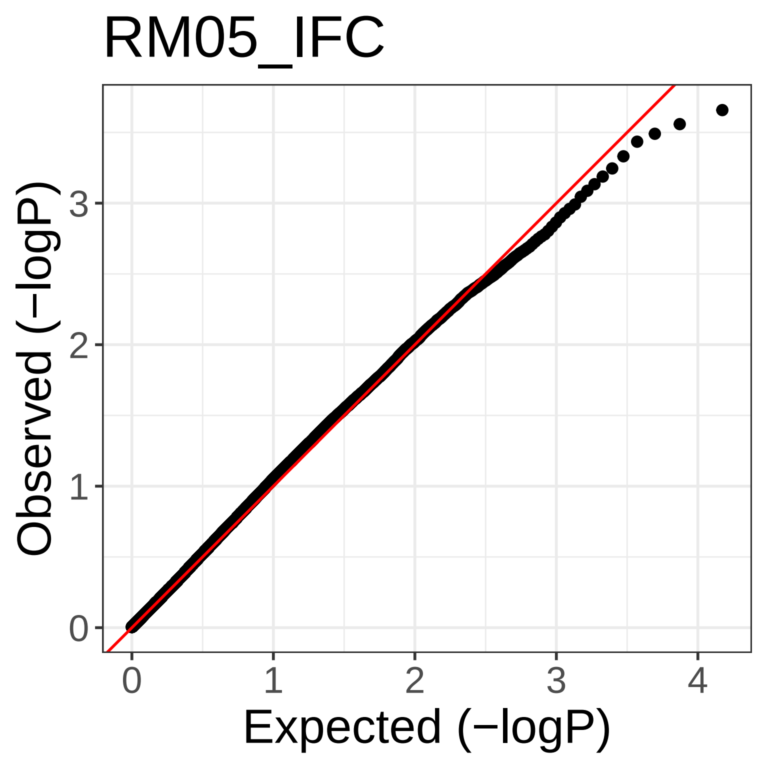
RM05_IFC
Observed (−logP)
Expected (−logP)
0
1
2
3
4

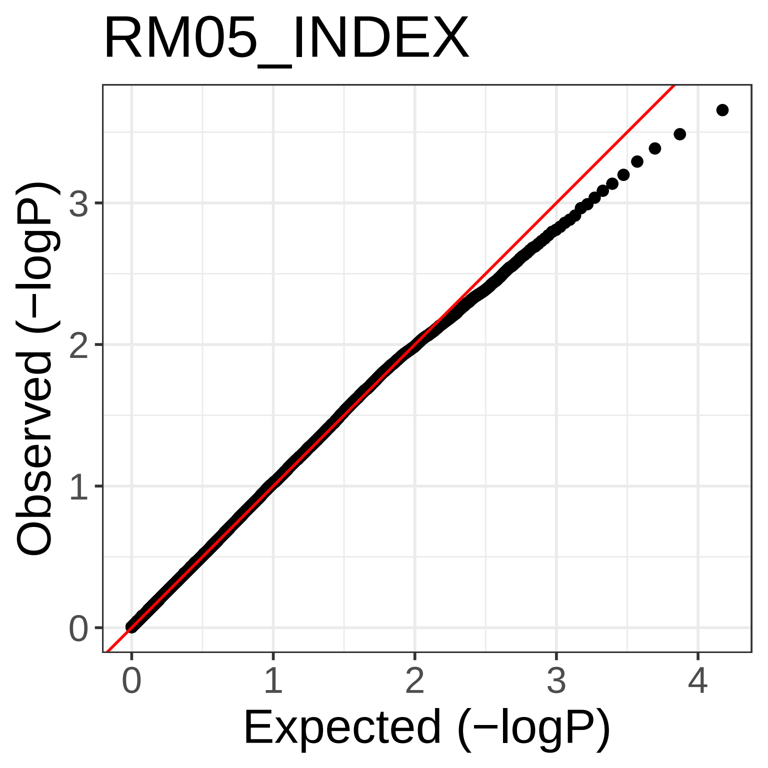
RM05_INDEX
Observed (−logP)
Expected (−logP)
0
1
2
3
4

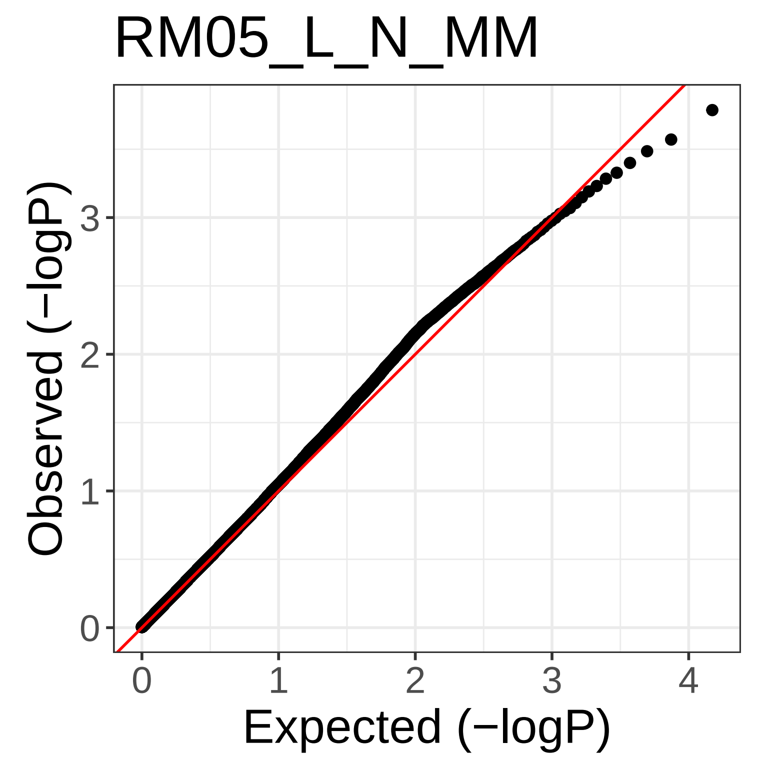
RM05_L_N_MM
Observed (−logP)
Expected (−logP)
0
1
2
3
4

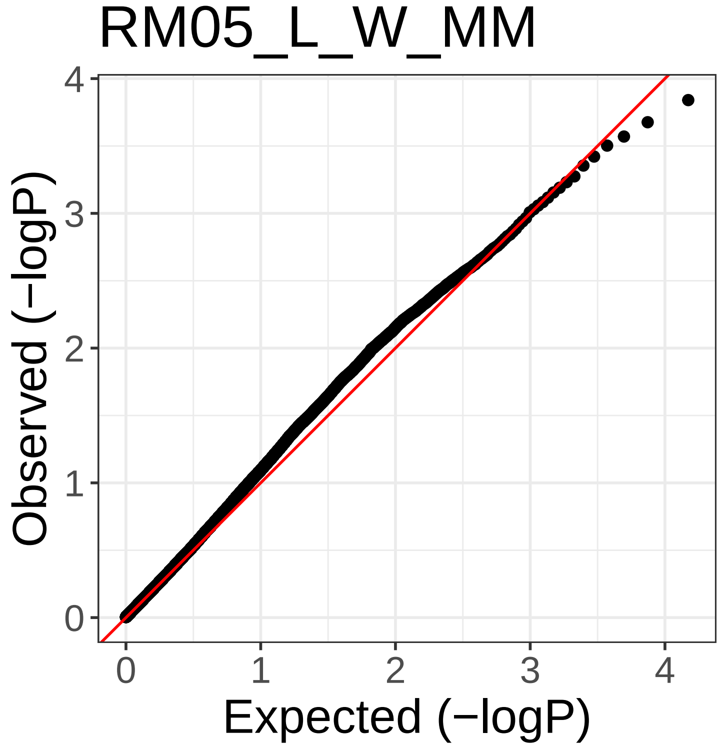
RM05_L_W_MM
Observed (−logP)
Expected (−logP)
0
1
2
3
4
0
1
2
3
4

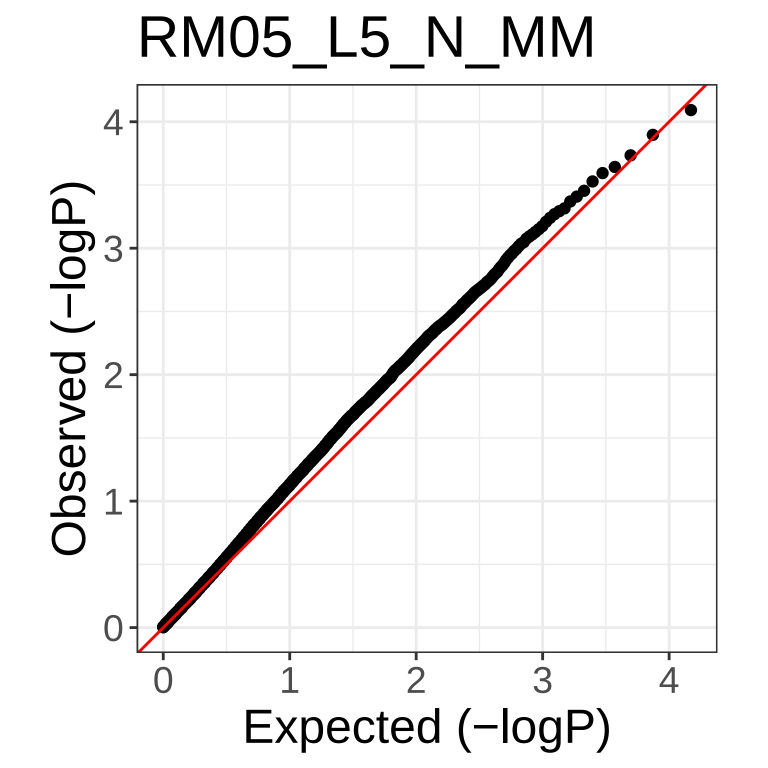

RM05_L5_N_MM
Observed (−logP)
Expected (−logP)
0
1
2
3
4
0
1
2
3
4

RM05_LYLG_KG

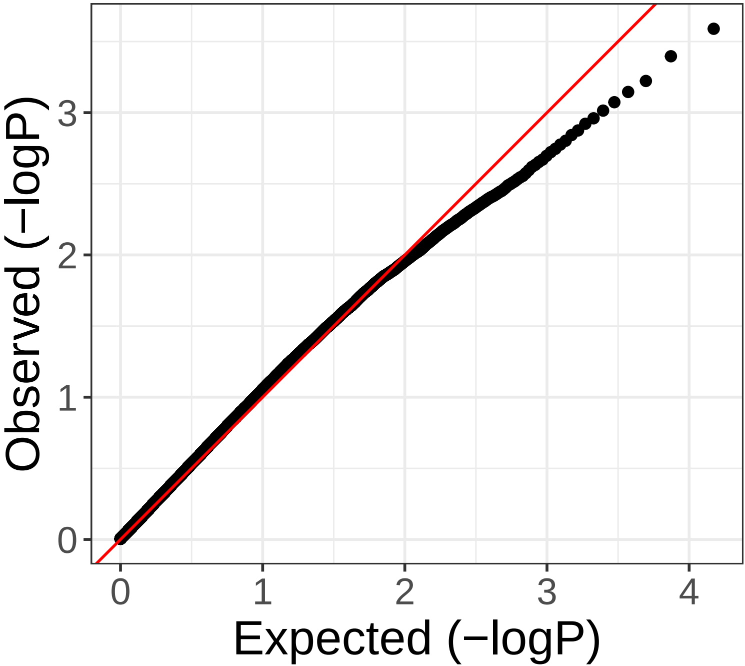

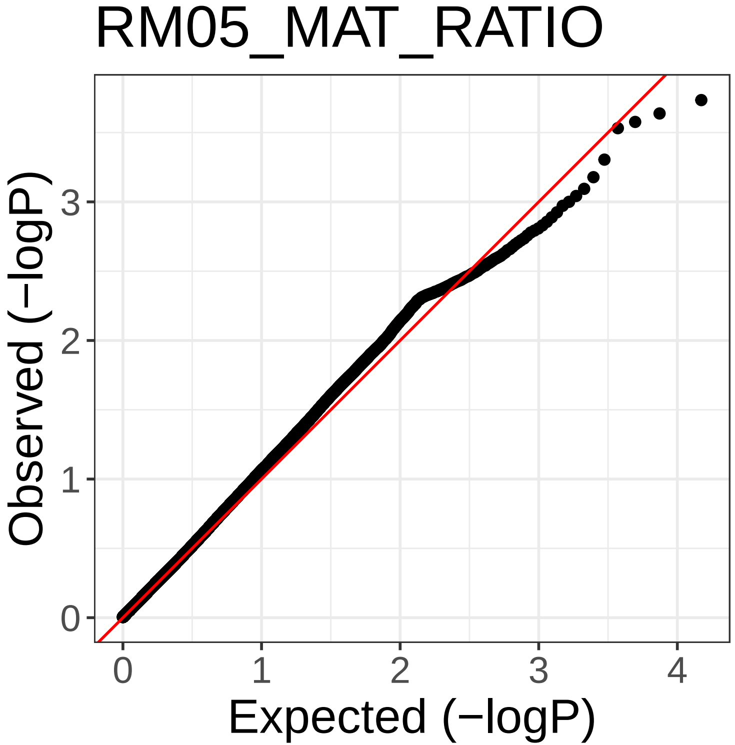
RM05_MAT_RATIO
Observed (−logP)
Expected (−logP)
0
1
2
3
0
1
2
3
4

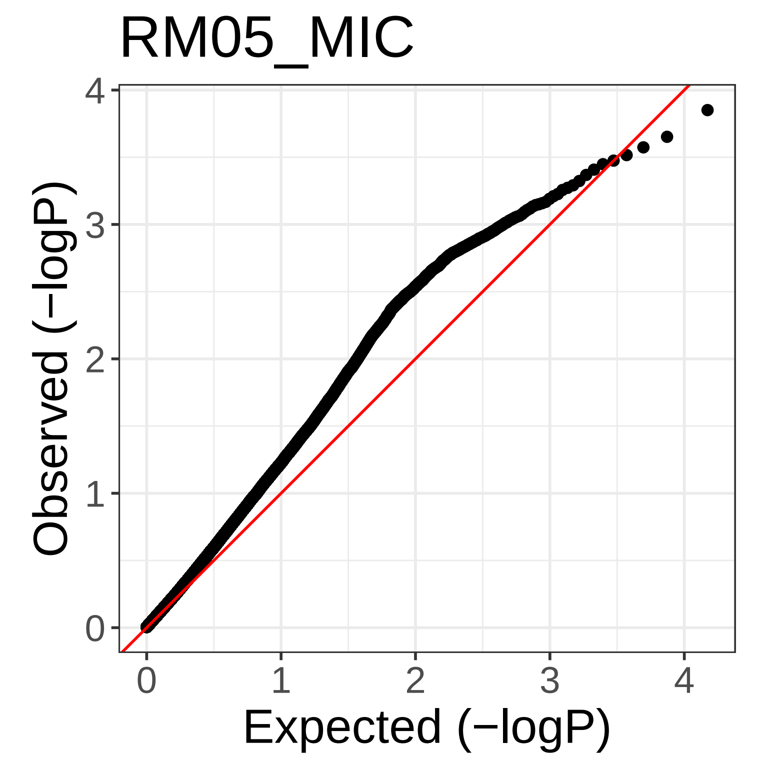

RM05_MIC
Observed (−logP)
Expected (−logP)
0
1
2
3
4
0
1
2
3
4

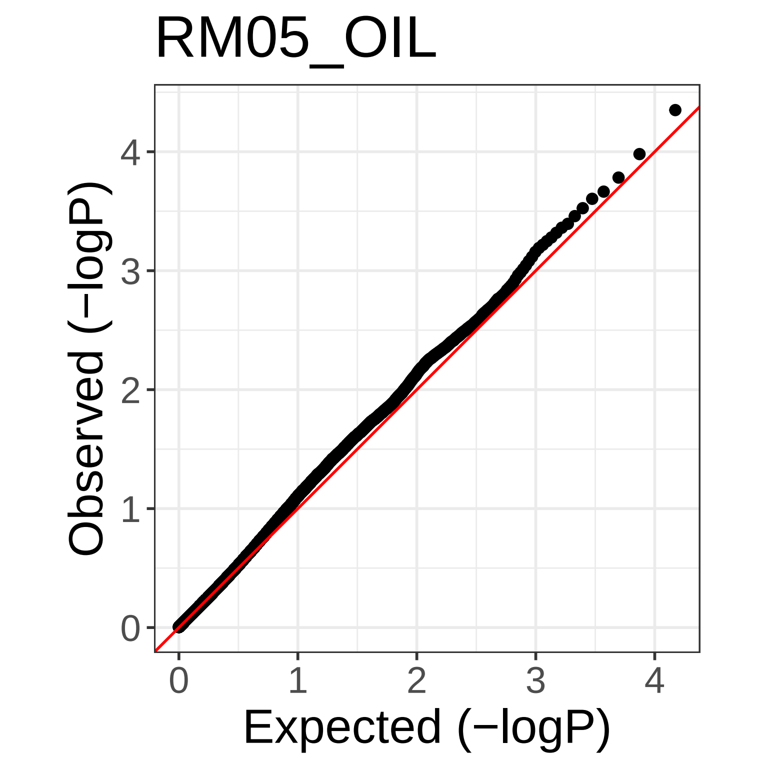

RM05_OIL
Observed (−logP)
Expected (−logP)
0
1
2
3
4
0
1
2
3
4

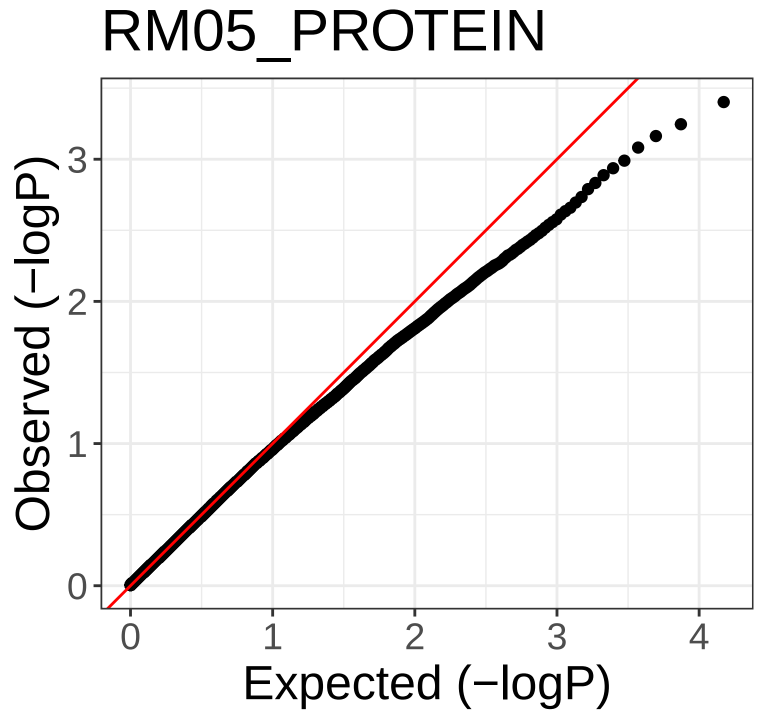
RM05_PROTEIN
Observed (−logP)
Expected (−logP)
0
1
2
3
0
1
2
3
4

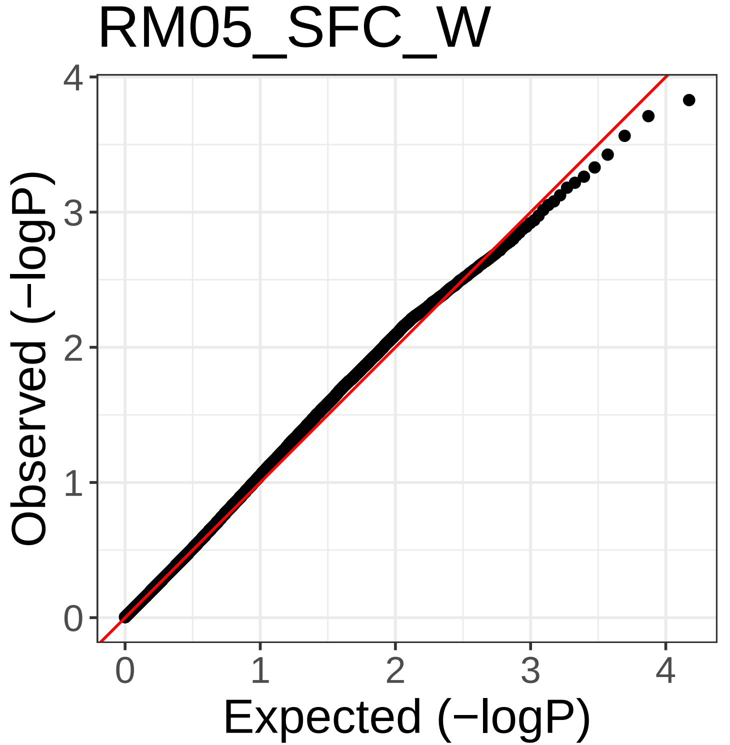
RM05_SFC_W
Observed (−logP)
Expected (−logP)
0
1
2
3
4
0
1
2
3
4

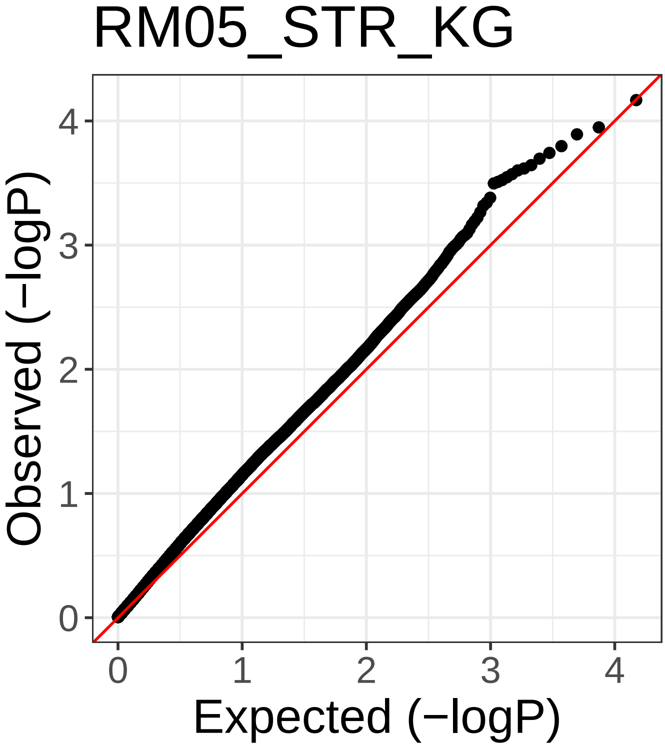

RM05_STR_KG
Observed (−logP)
Expected (−logP)
0
1
2
3
4
0
1
2
3
4

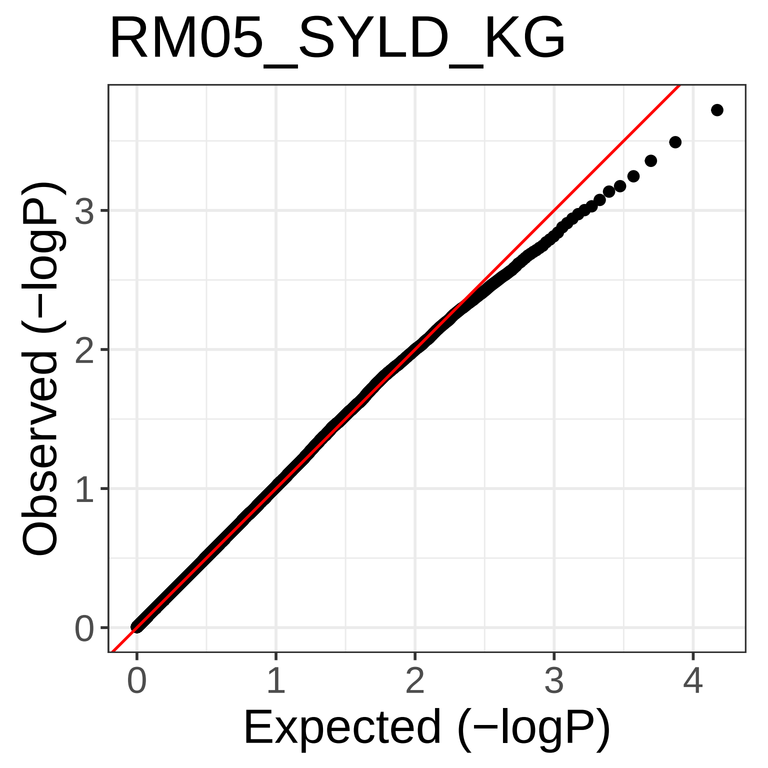
RM05_SYLD_KG
Observed (−logP)
Expected (−logP)
0
1
2
3
0
1
2
3
4

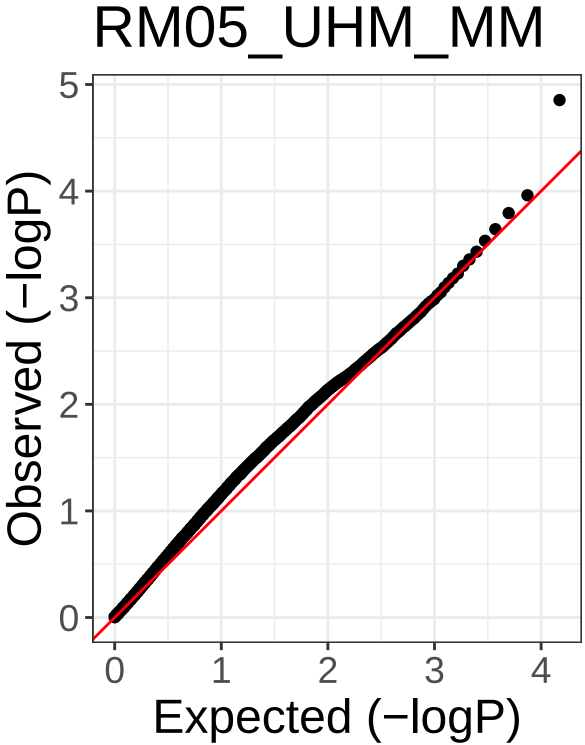
RM05_UHM_MM
Observed (−logP)
Expected (−logP)
0
1
2
3
4
5
0
1
2
3
4

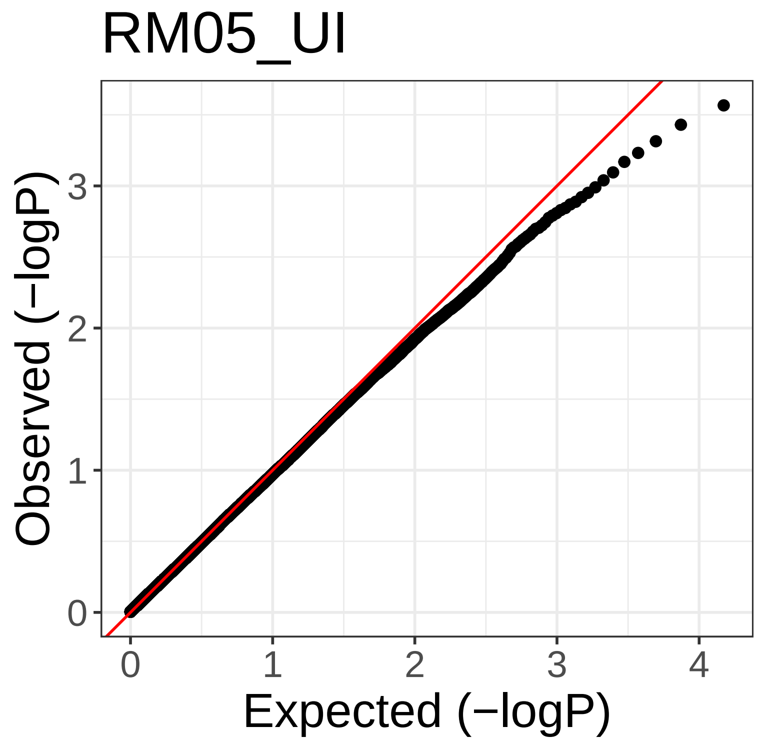
RM05_UI
Observed (−logP)
Expected (−logP)
0
1
2
3
4

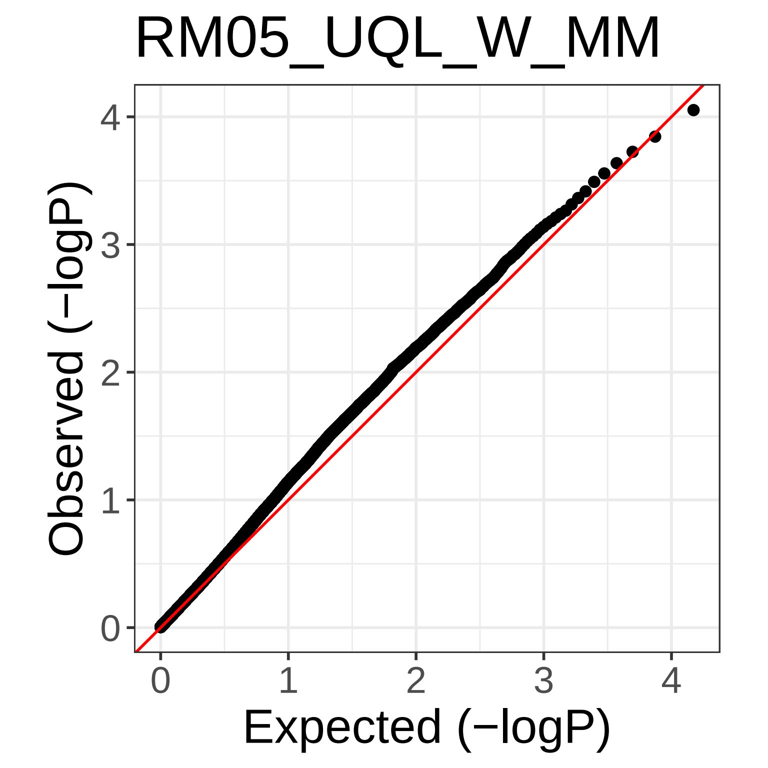
RM05_UQL_W_MM
Observed (−logP)
Expected (−logP)
0
1
2
3
4
0
1
2
3
4

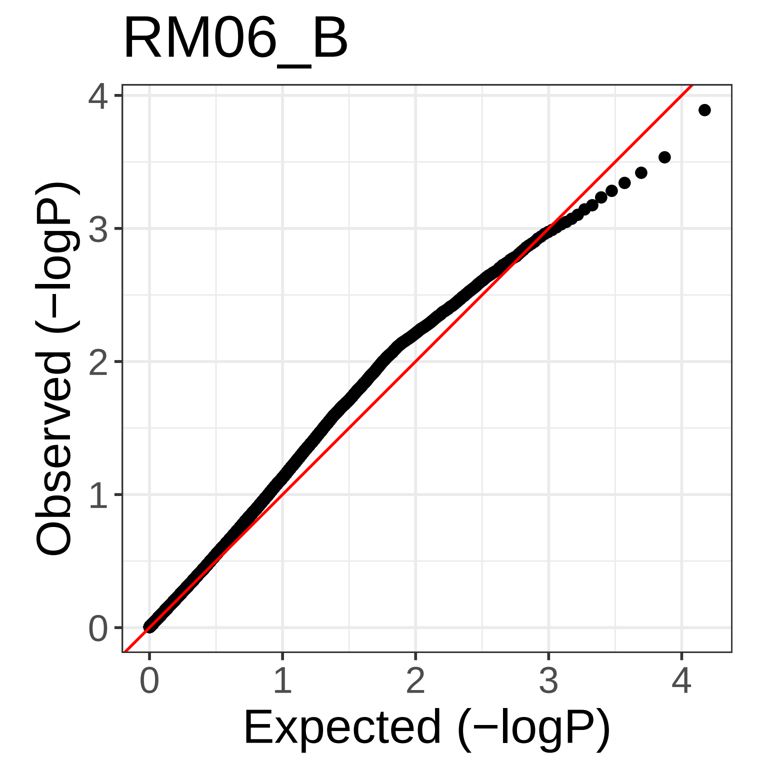

RM06_B
Observed (−logP)
Expected (−logP)
0
1
2
3
4
0
1
2
3
4

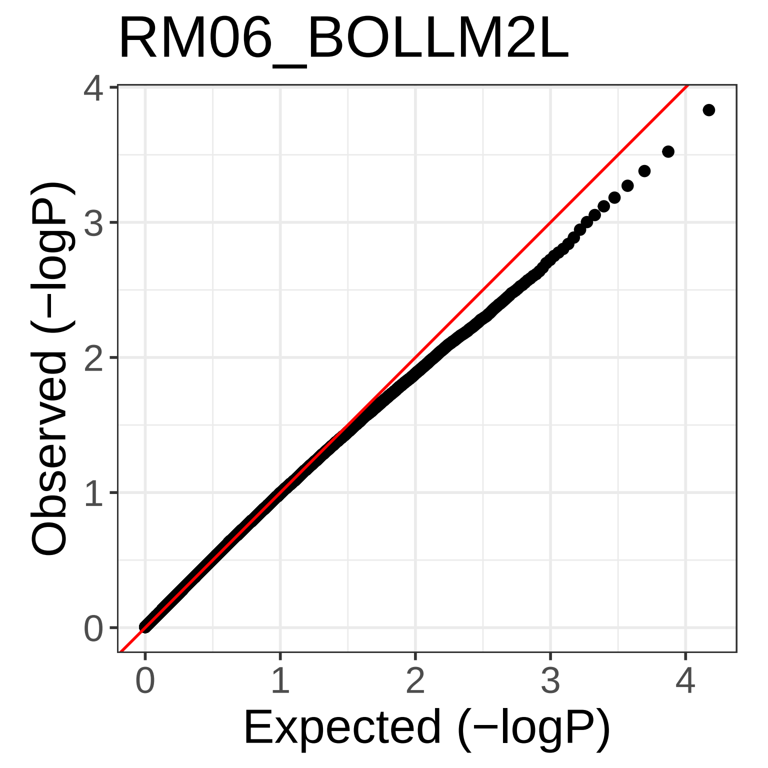

RM06_BOLLM2L
Observed (−logP)
Expected (−logP)
0
1
2
3
4
0
1
2
3
4

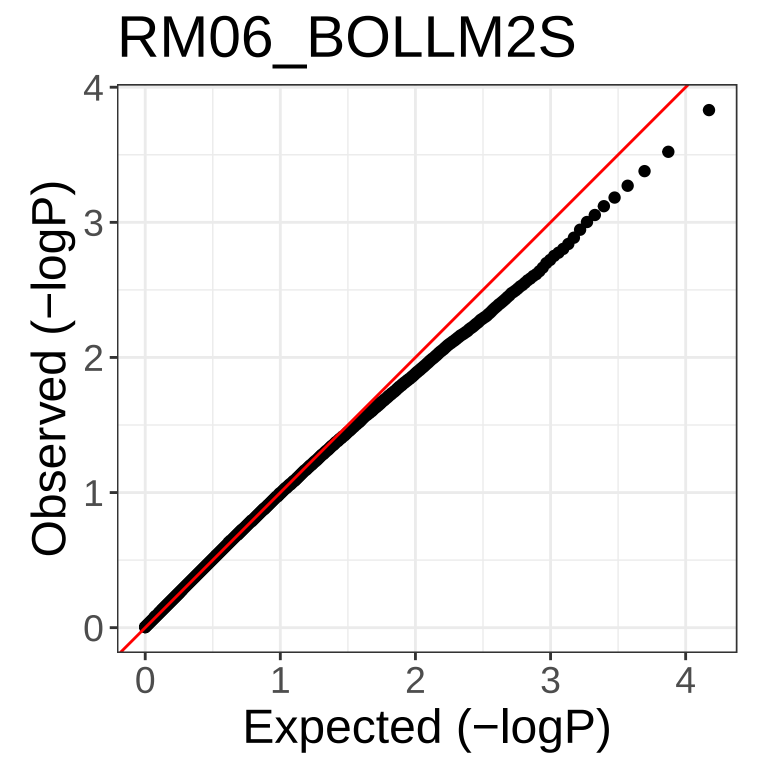
RM06_BOLLM2S
Observed (−logP)
Expected (−logP)
0
1
2
3
4
0
1
2
3
4

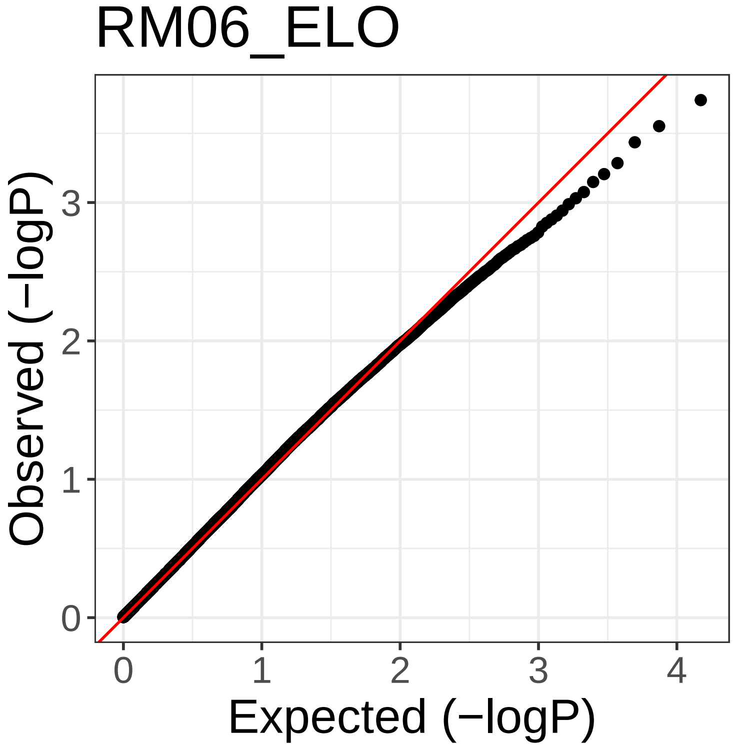
RM06_ELO
Observed (−logP)
Expected (−logP)
0
1
2
3
0
1
2
3
4

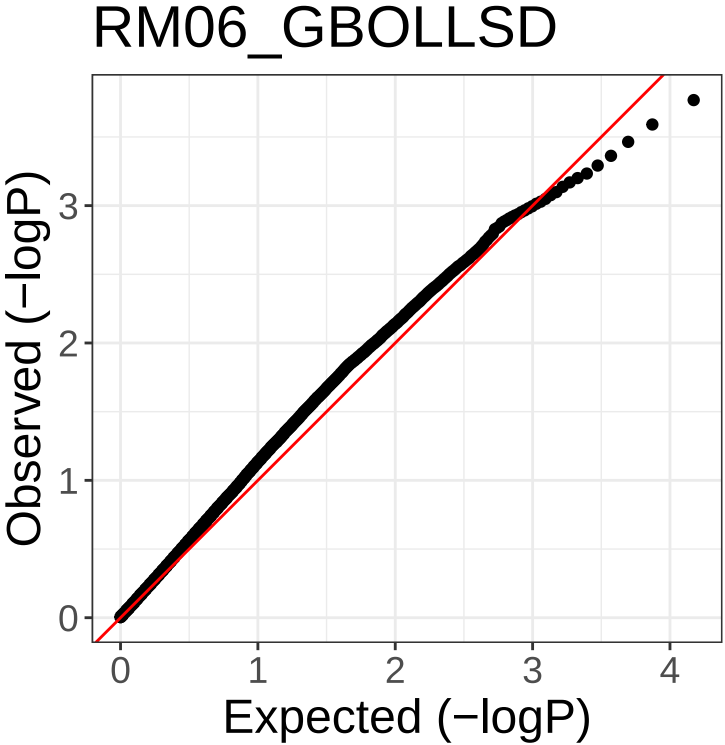

RM06_GBOLLSD
Observed (−logP)
Expected (−logP)
0
1
2
3
4

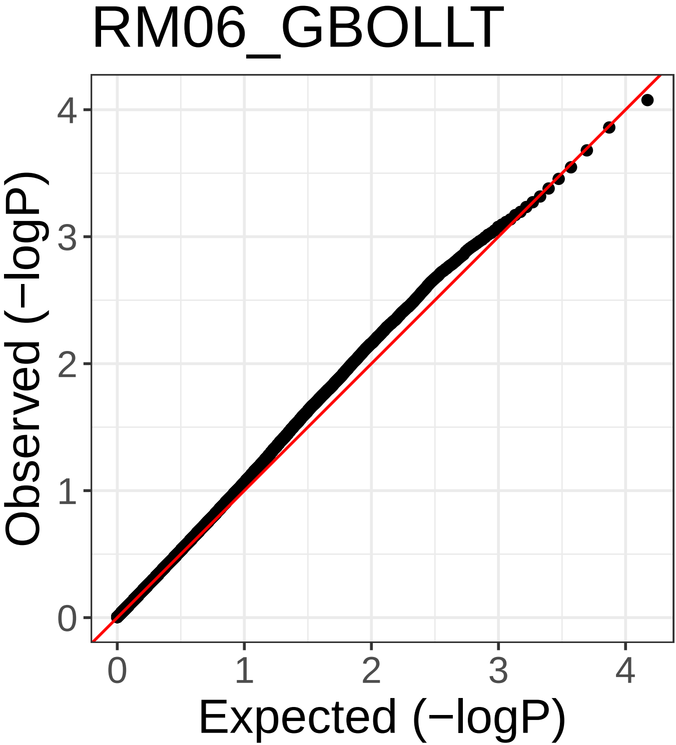

RM06_GBOLLT
Observed (−logP)
Expected (−logP)
0
1
2
3
4
0
1
2
3
4

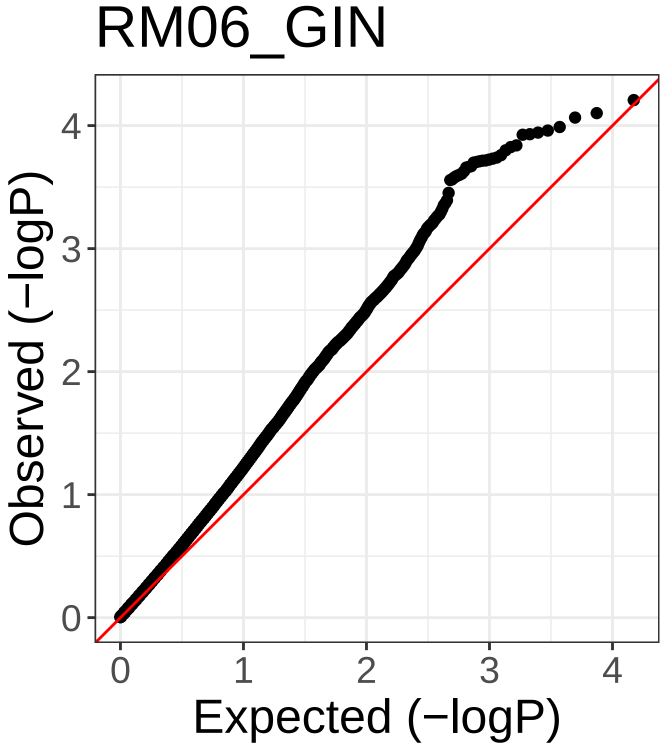
RM06_GIN
Observed (−logP)
Expected (−logP)
0
1
2
3
4
0
1
2
3
4

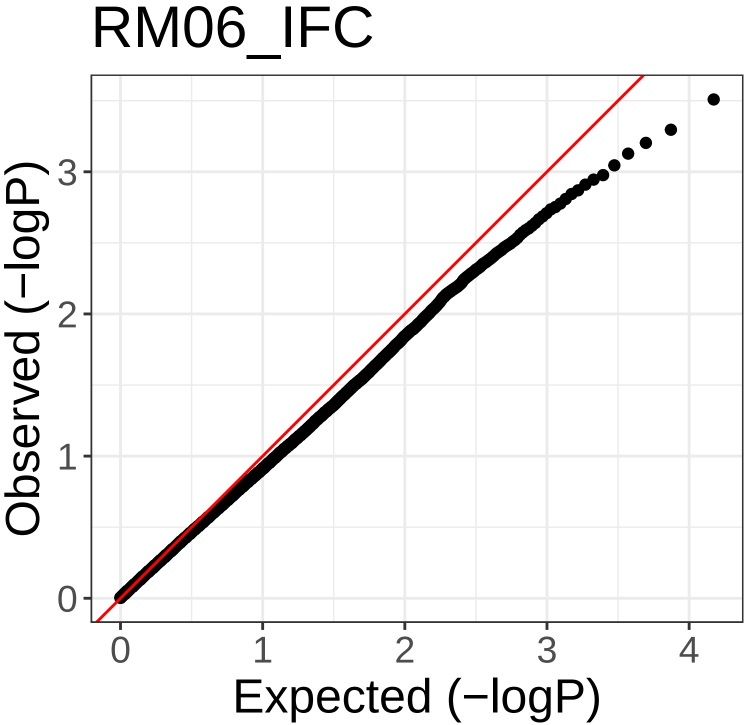
RM06_IFC
Observed (−logP)
Expected (−logP)
0
1
2
3
4

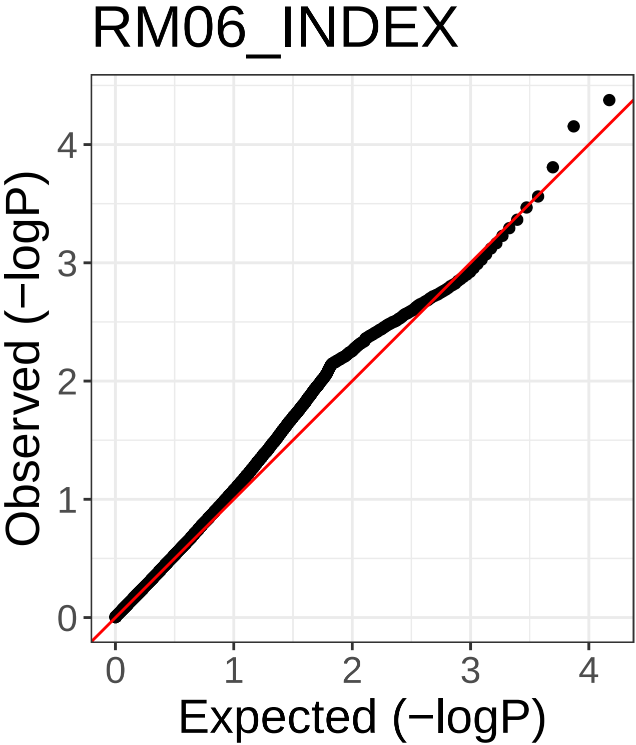
RM06_INDEX
Observed (−logP)
Expected (−logP)
0
1
2
3
4
0
1
2
3
4

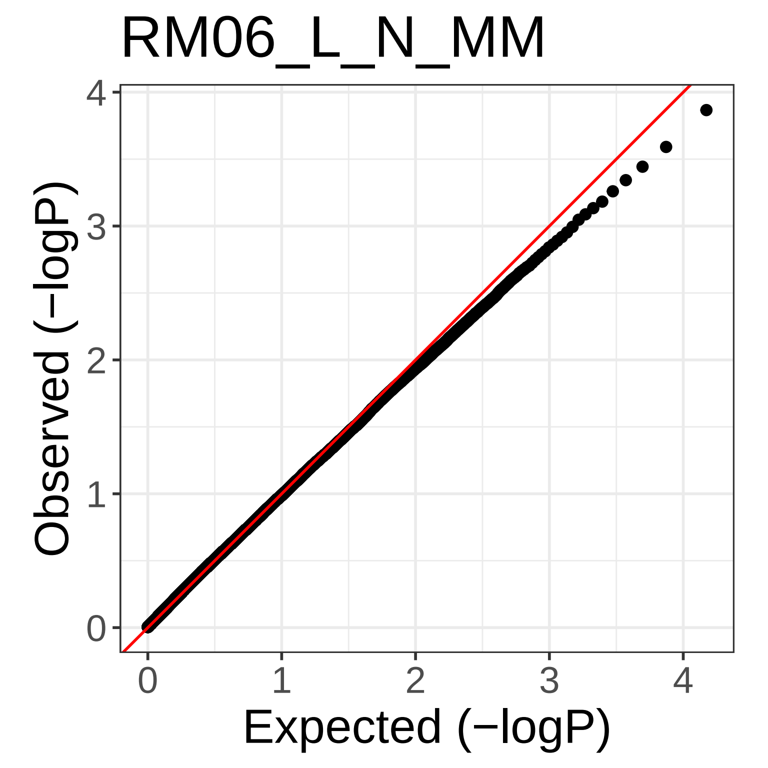
RM06_L_N_MM
Observed (−logP)
Expected (−logP)
0
1
2
3
4

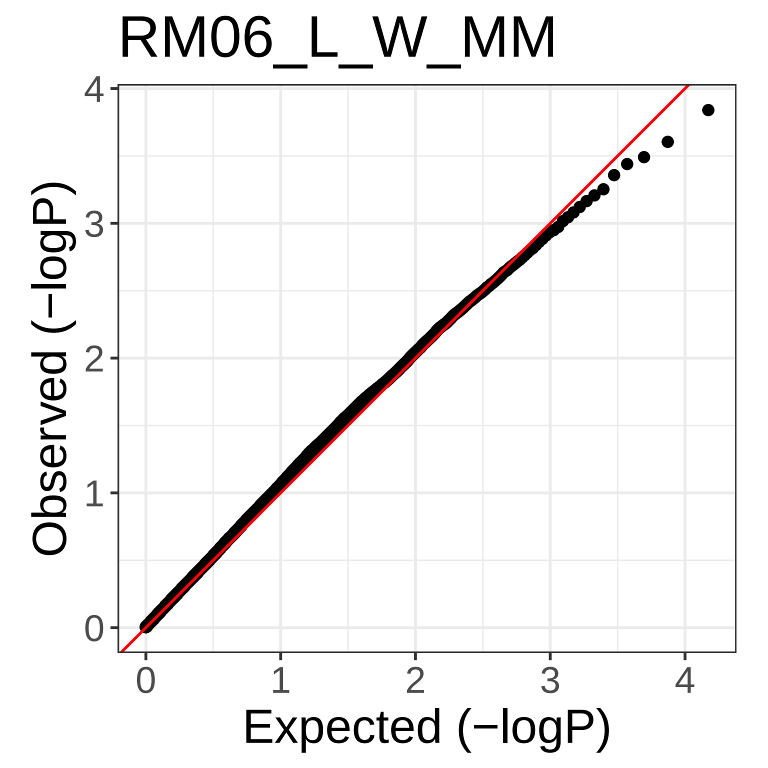
RM06_L_W_MM
Observed (−logP)
Expected (−logP)
0
1
2
3
4
0
1
2
3
4

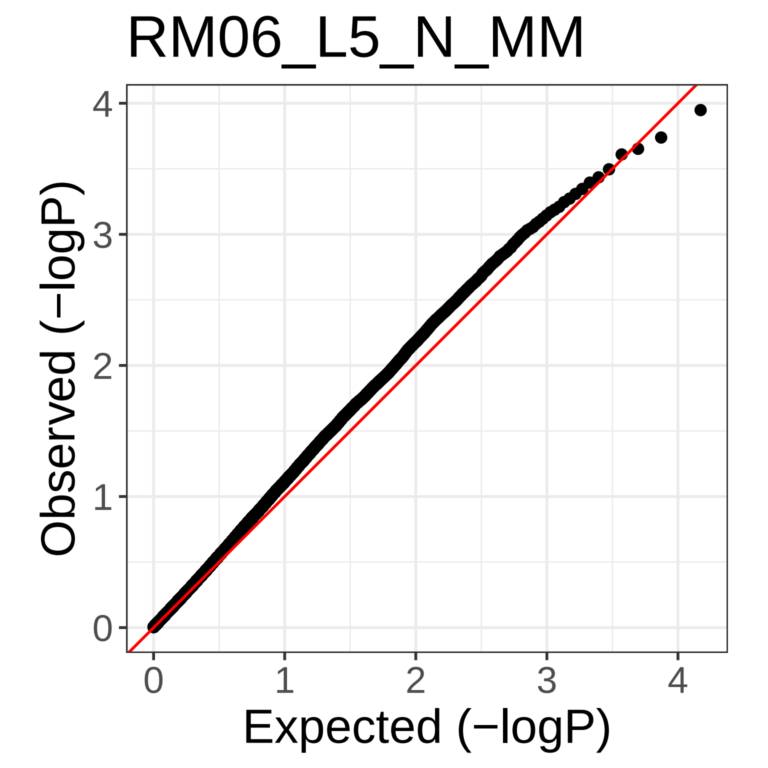
RM06_L5_N_MM
Observed (−logP)
Expected (−logP)
0
1
2
3
4
0
1
2
3
4

RM06_LYLG_KG

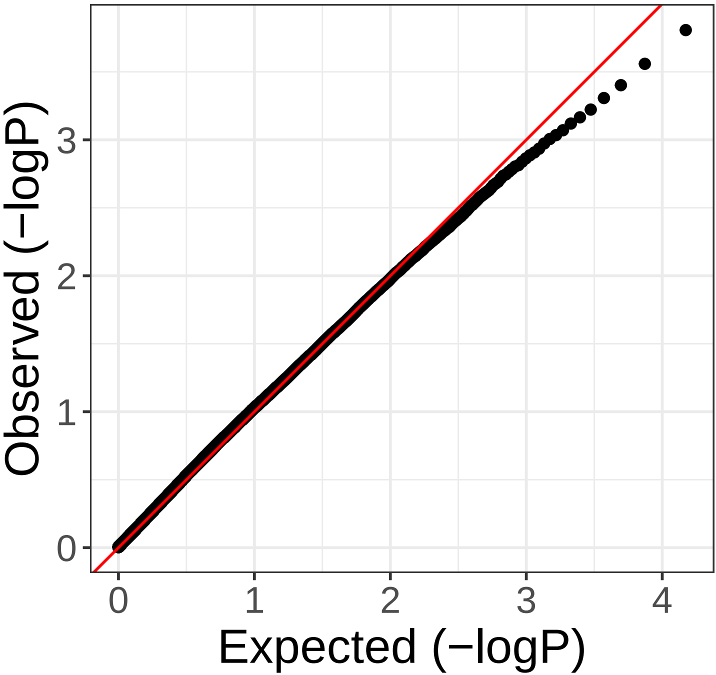

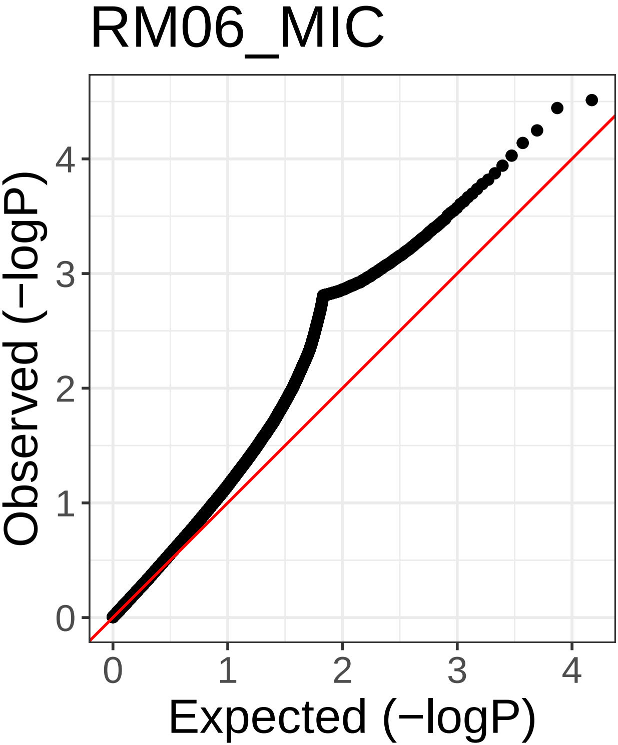
RM06_MIC
Observed (−logP)
Expected (−logP)
0
1
2
3
4
0
1
2
3
4

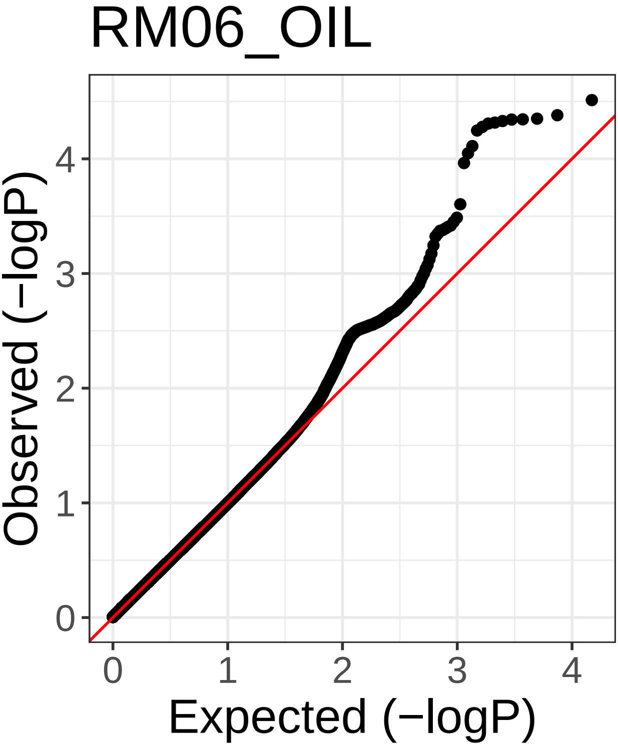

RM06_OIL
Observed (−logP)
Expected (−logP)
0
1
2
3
4
0
1
2
3
4

RM06_PROTEIN

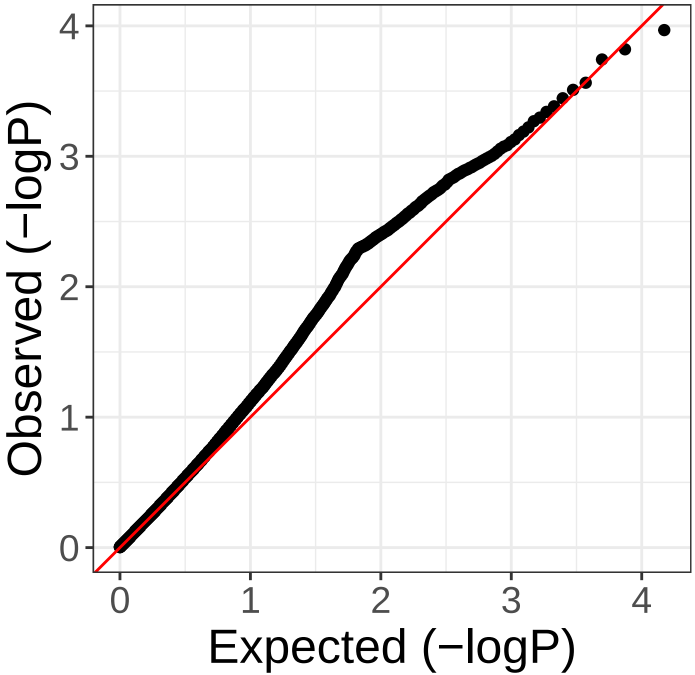

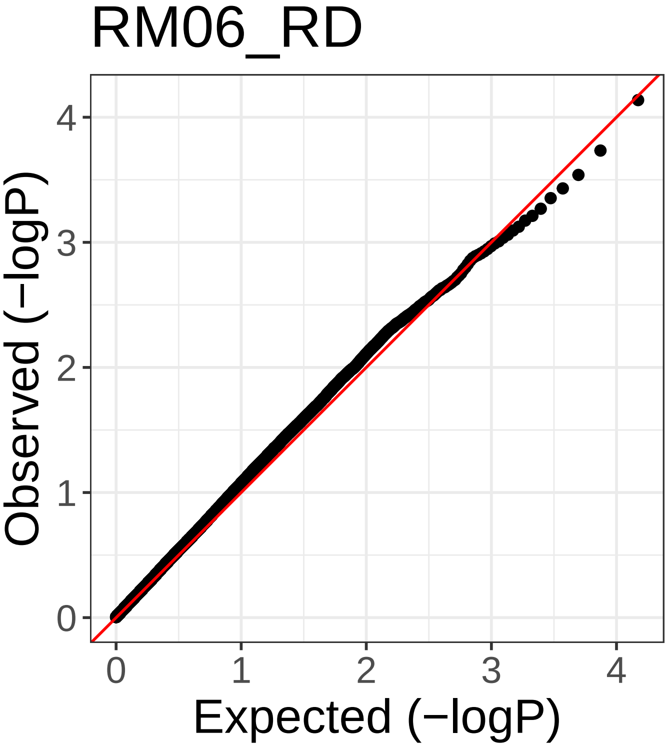
RM06_RD
Observed (−logP)
Expected (−logP)
0
1
2
3
4
0
1
2
3
4

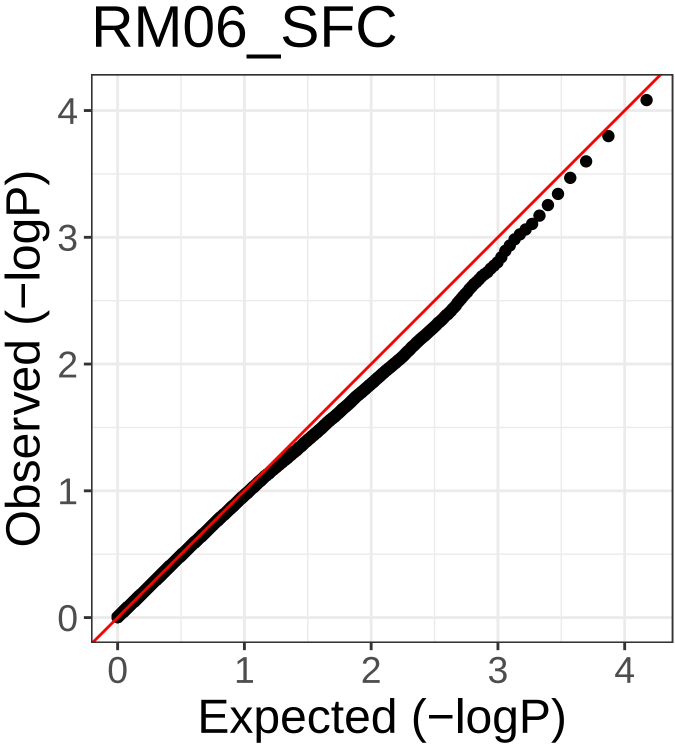
RM06_SFC
Observed (−logP)
Expected (−logP)
0
1
2
3
4
0
1
2
3
4

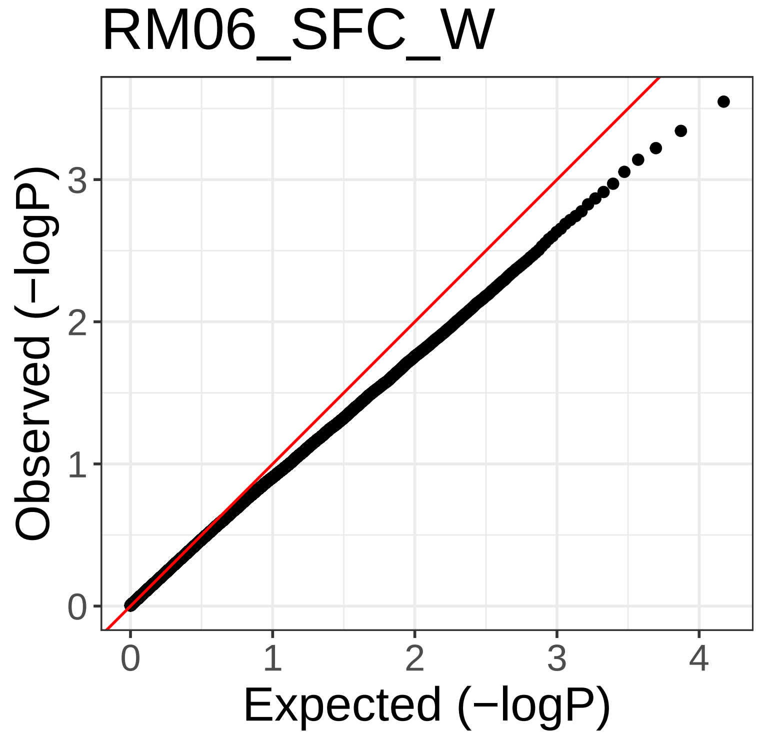
RM06_SFC_W
Observed (−logP)
Expected (−logP)
0
1
2
3
0
1
2
3
4

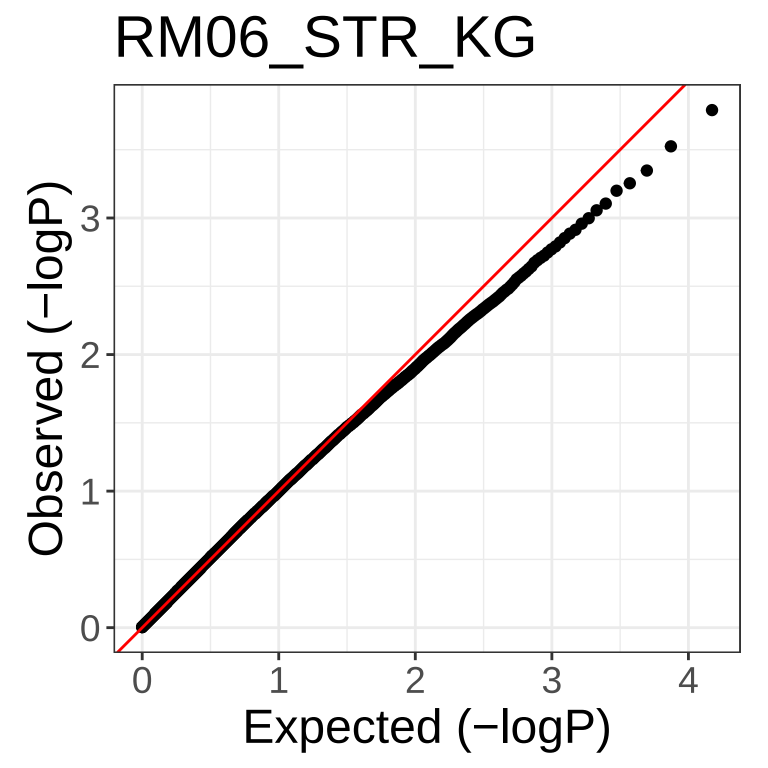
RM06_STR_KG
Observed (−logP)
Expected (−logP)
0
1
2
3
4

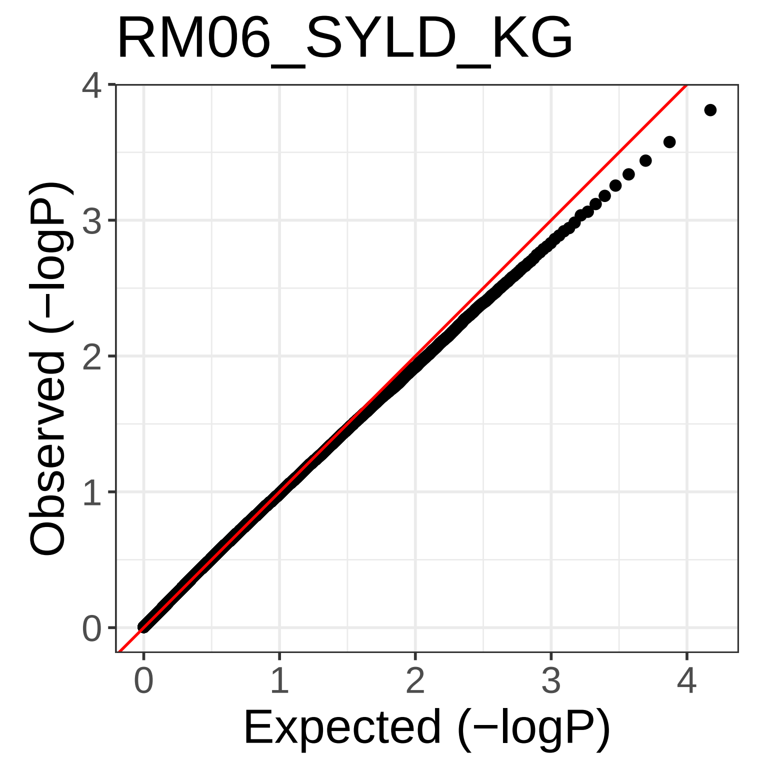
RM06_SYLD_KG
Observed (−logP)
Expected (−logP)
0
1
2
3
4

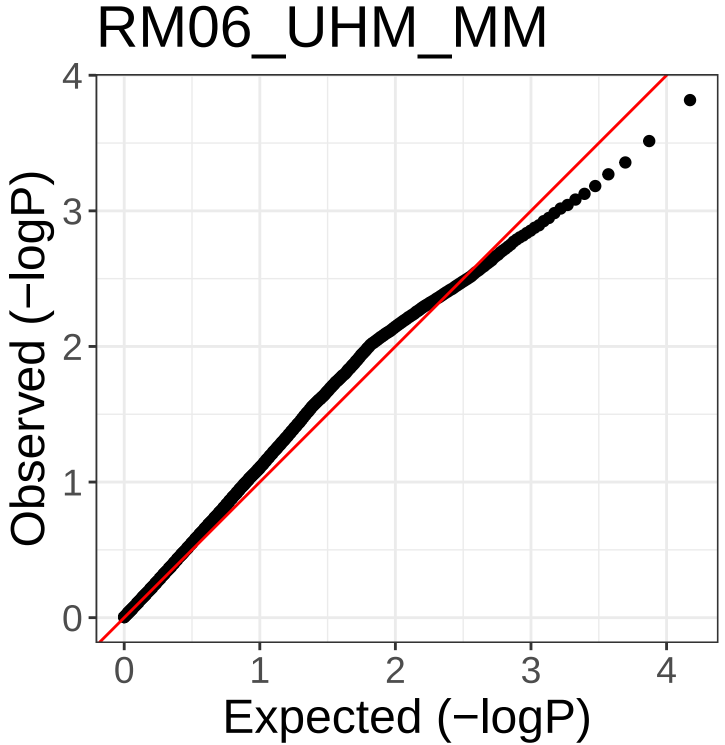
RM06_UHM_MM
Observed (−logP)
Expected (−logP)
0
1
2
3
4
0
1
2
3
4

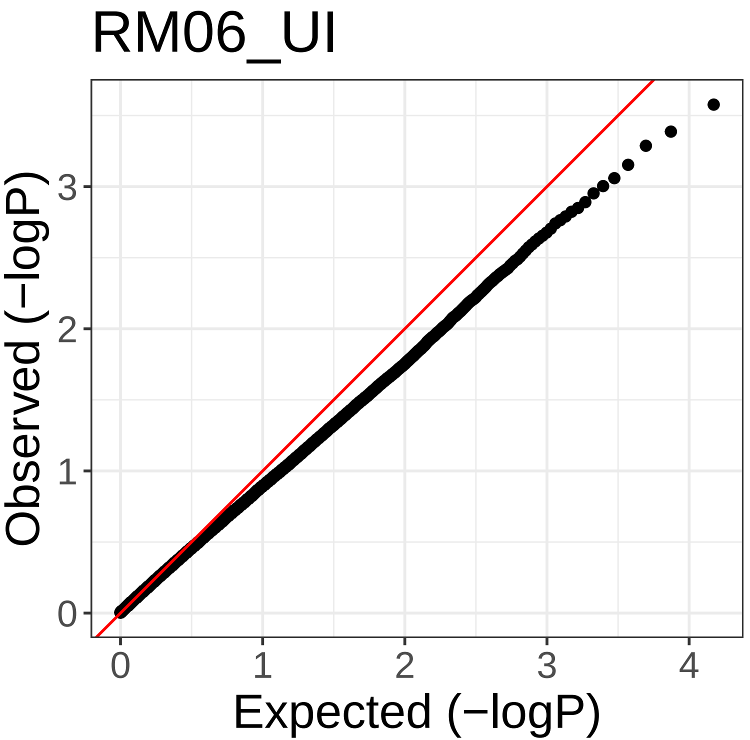
RM06_UI
Observed (−logP)
Expected (−logP)
0
1
2
3
4

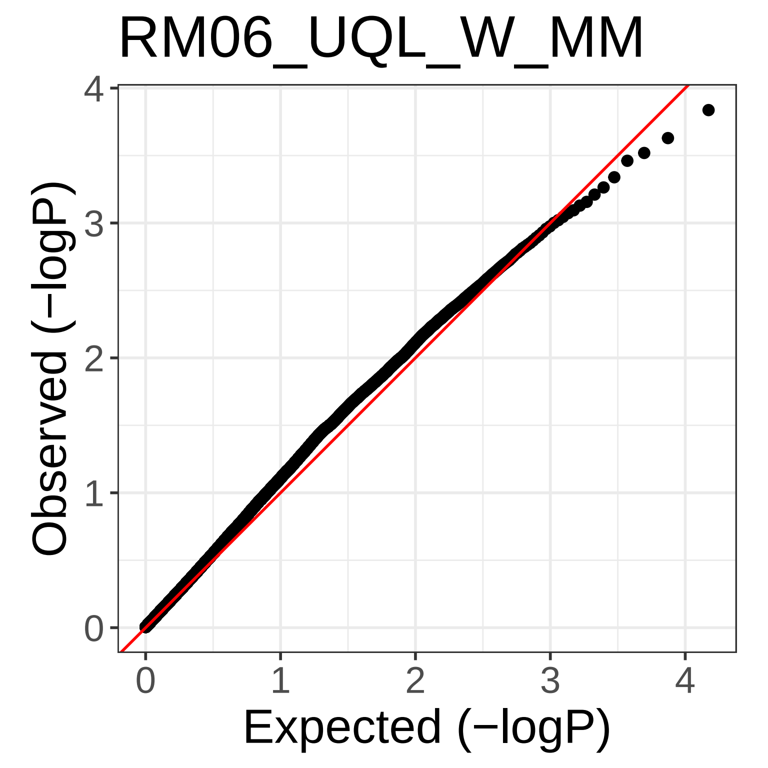
RM06_UQL_W_MM
Observed (−logP)
Expected (−logP)
0
1
2
3
4
0
1
2
3
4

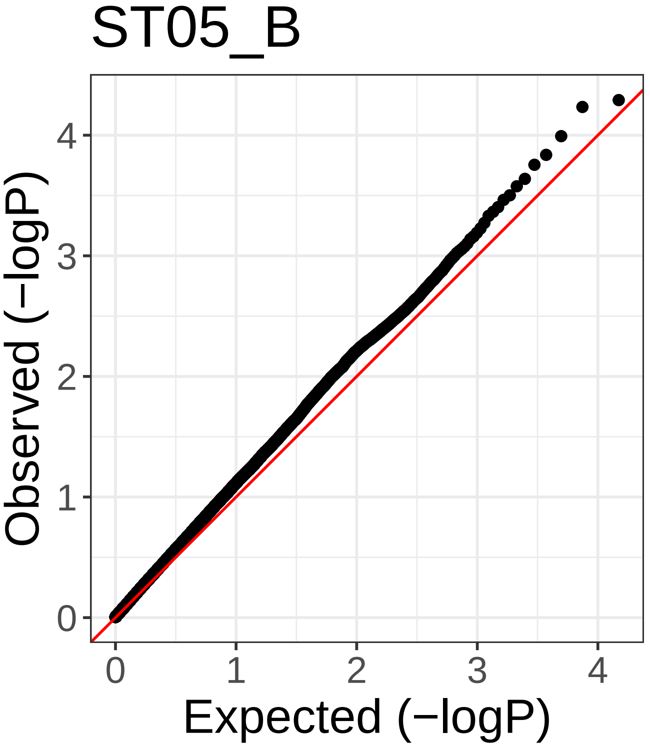

ST05_B
Observed (−logP)
Expected (−logP)
0
1
2
3
4

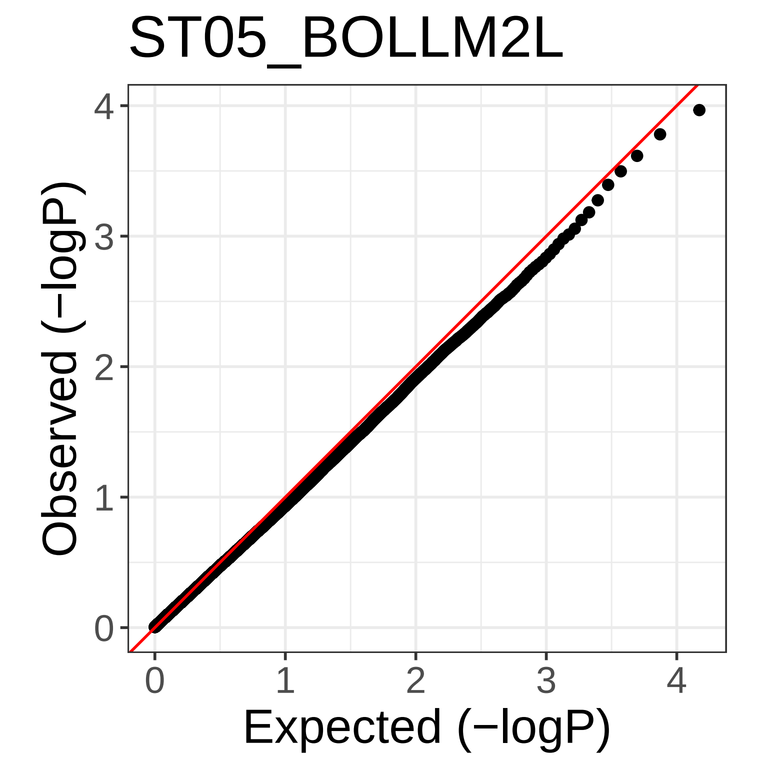

ST05_BOLLM2L
Observed (−logP)
Expected (−logP)
0
1
2
3
4

ST05_BOLLM2S

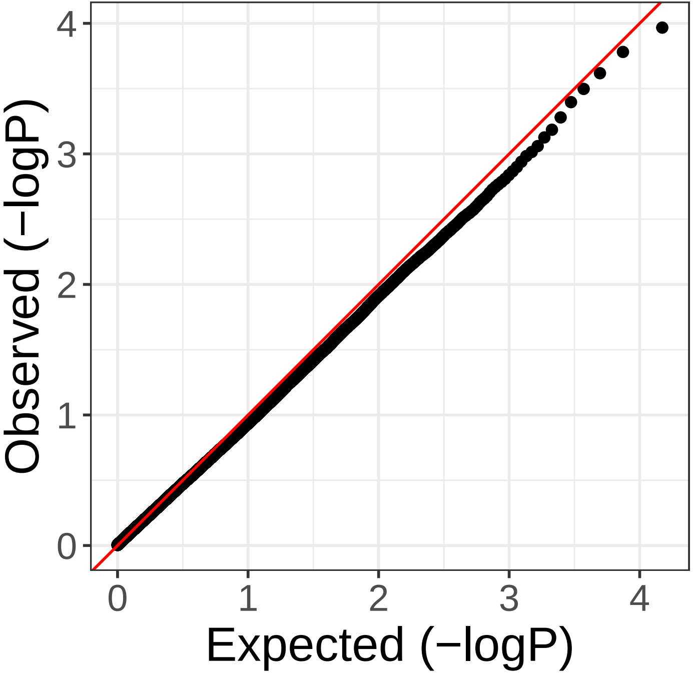

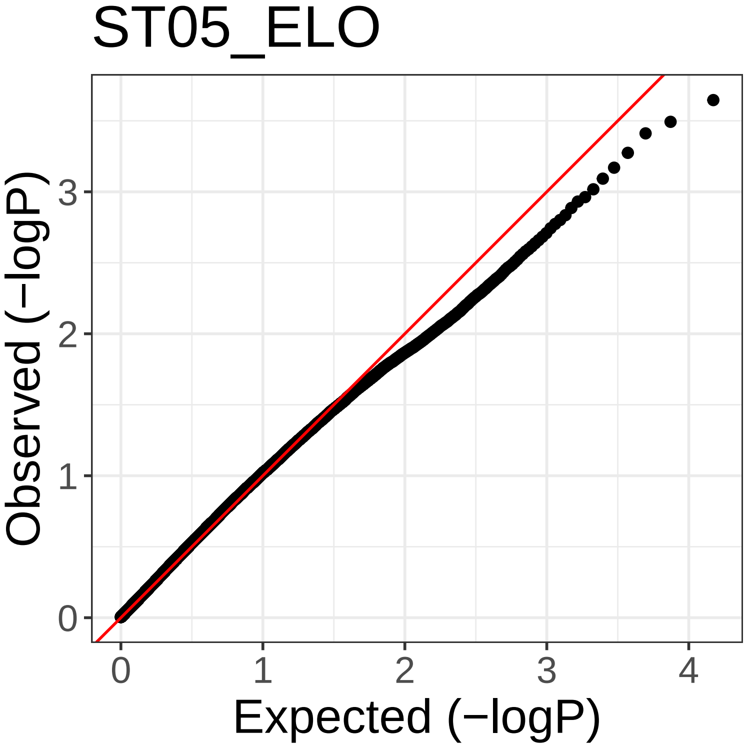

ST05_ELO
Observed (−logP)
Expected (−logP)
0
1
2
3
0
1
2
3
4

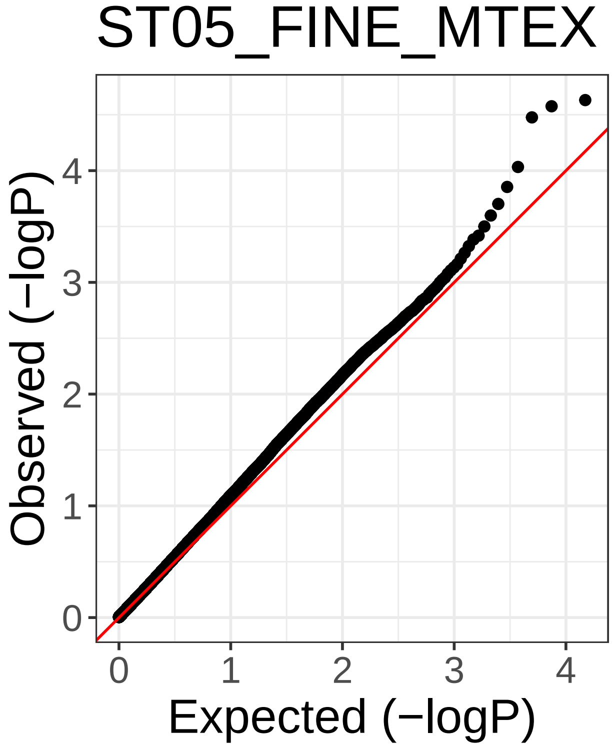
ST05_FINE_MTEX
Observed (−logP)
Expected (−logP)
0
1
2
3
4
0
1
2
3
4

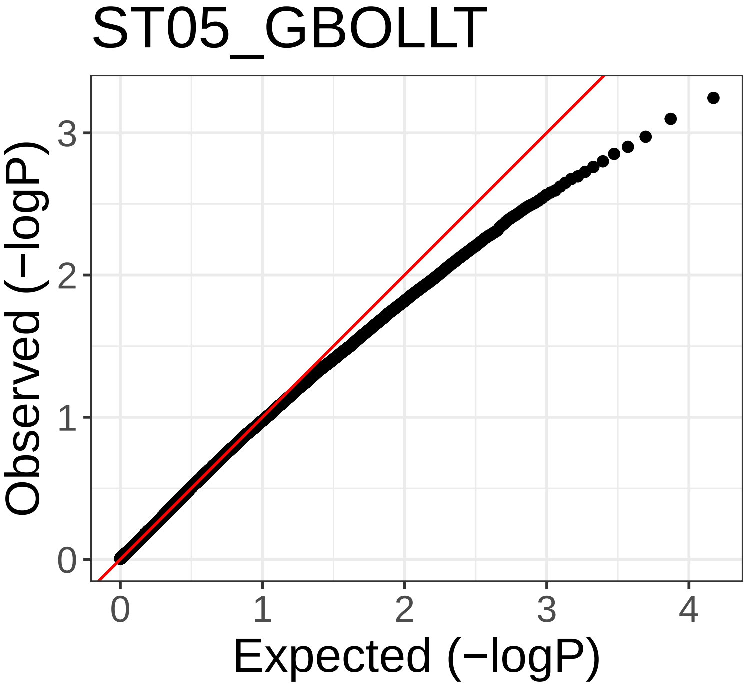
ST05_GBOLLT
Observed (−logP)
Expected (−logP)
0
1
2
3
0
1
2
3
4

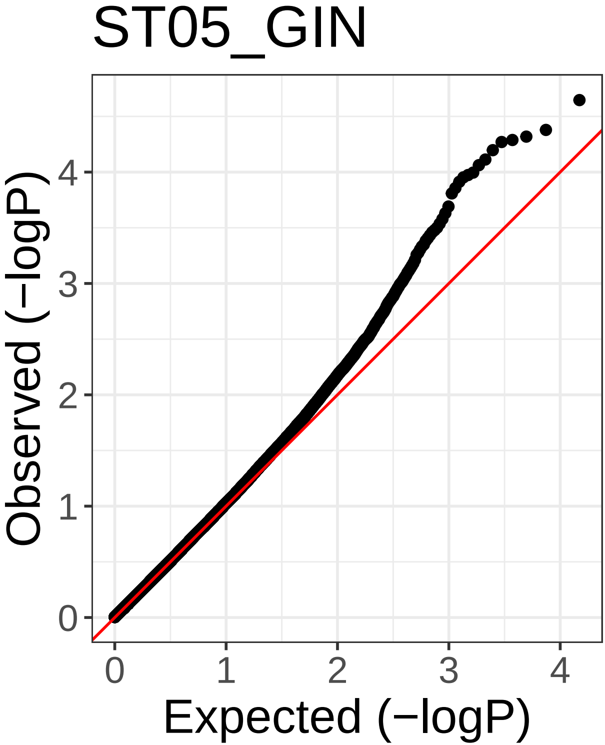
ST05_GIN
Observed (−logP)
Expected (−logP)
0
1
2
3
4
0
1
2
3
4

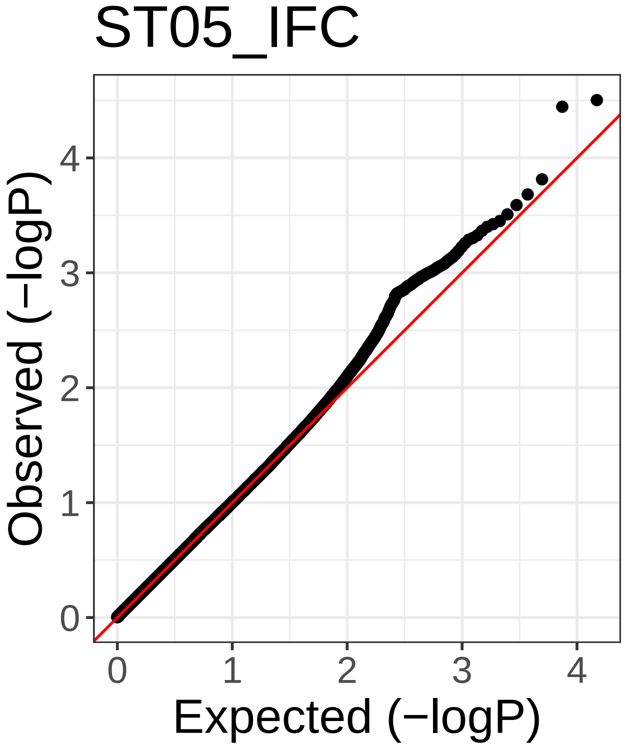
ST05_IFC
Observed (−logP)
Expected (−logP)
0
1
2
3
4
0
1
2
3
4

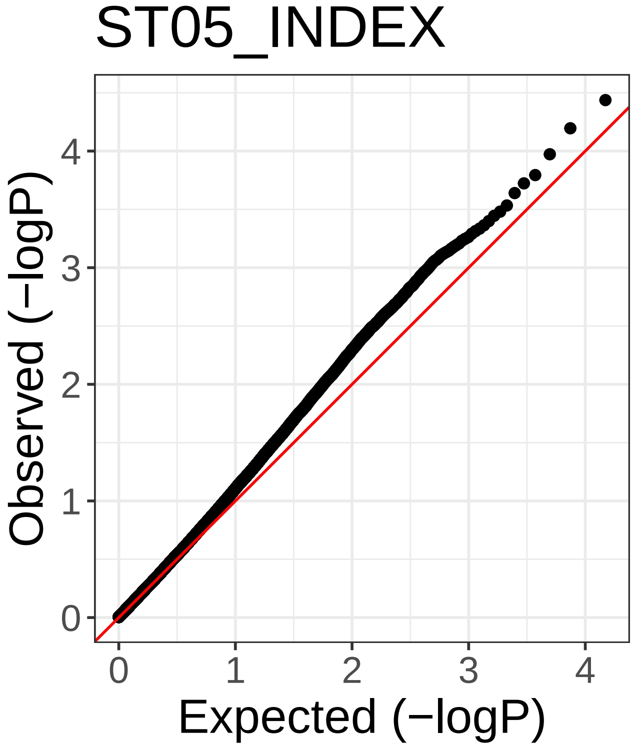

ST05_INDEX
Observed (−logP)
Expected (−logP)
0
1
2
3
4
0
1
2
3
4

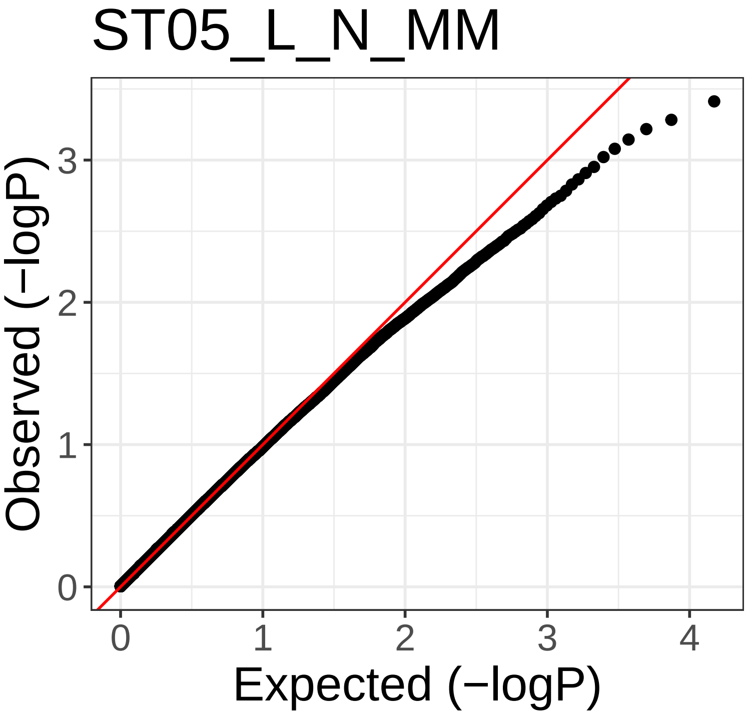

ST05_L_N_MM
Observed (−logP)
Expected (−logP)
0
1
2
3
4

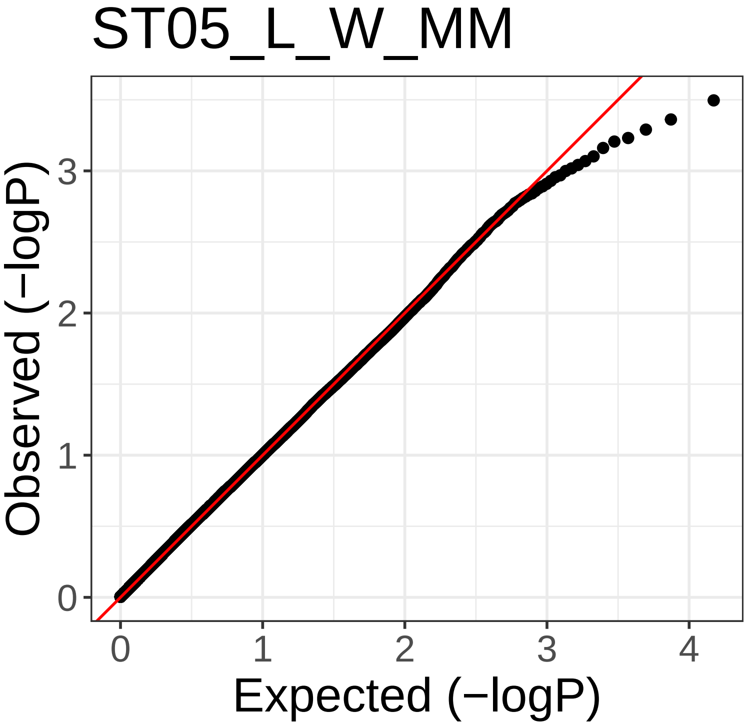

ST05_L_W_MM
Observed (−logP)
Expected (−logP)
0
1
2
3
4

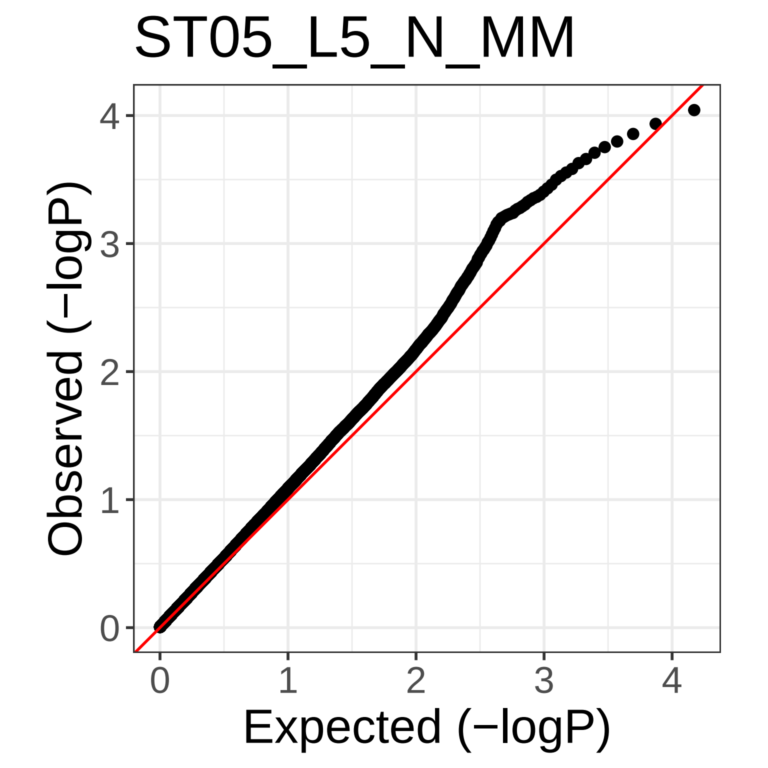
ST05_L5_N_MM
Observed (−logP)
Expected (−logP)
0
1
2
3
4
0
1
2
3
4

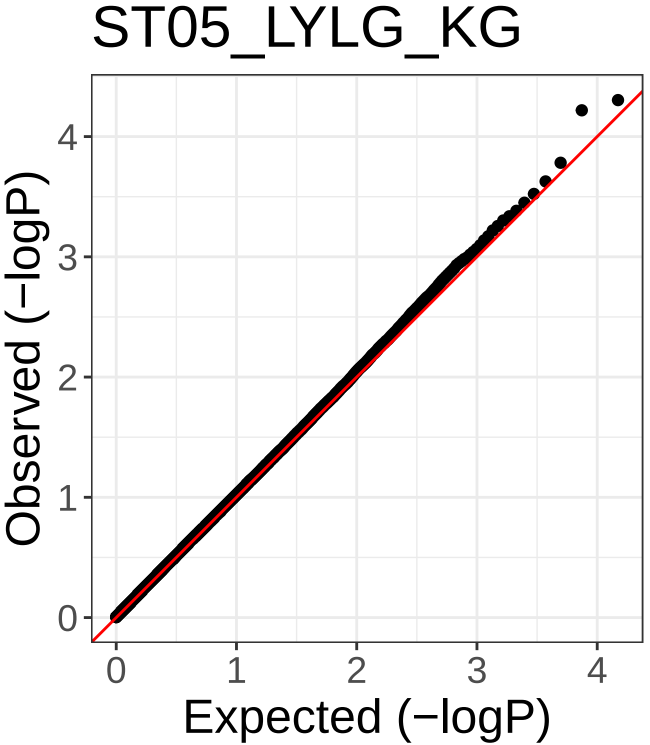
ST05_LYLG_KG
Observed (−logP)
Expected (−logP)
0
1
2
3
4
0
1
2
3
4

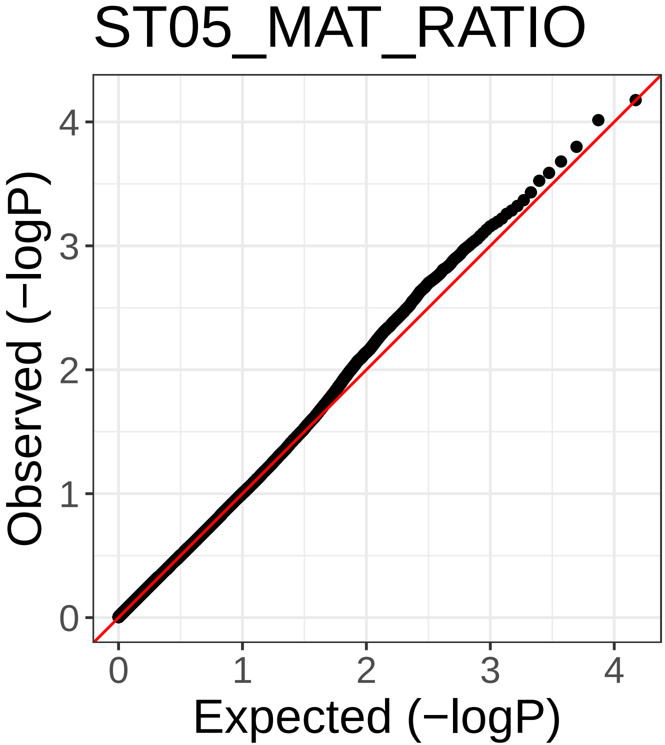

ST05_MAT_RATIO
Observed (−logP)
Expected (−logP)
0
1
2
3
4

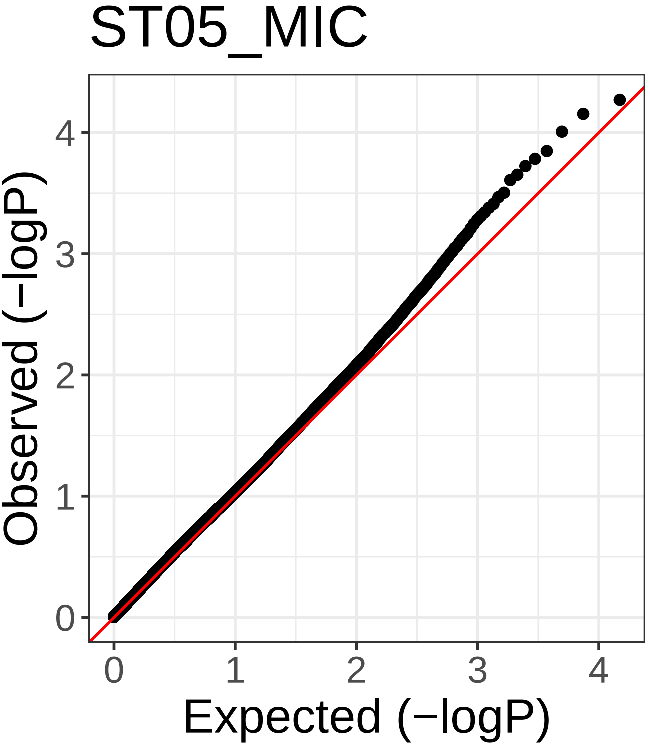

ST05_MIC
Observed (−logP)
Expected (−logP)
0
1
2
3
4
0
1
2
3
4

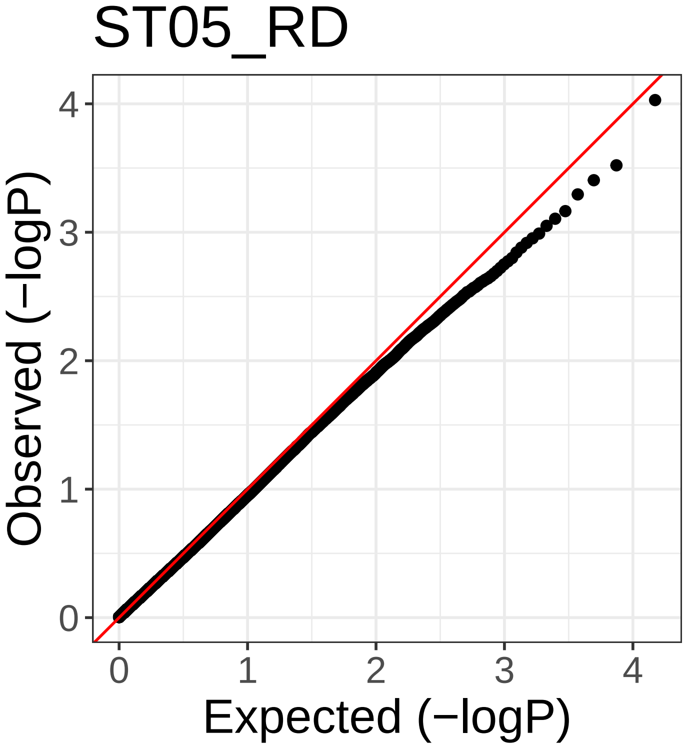
ST05_RD
Observed (−logP)
Expected (−logP)
0
1
2
3
4
0
1
2
3
4

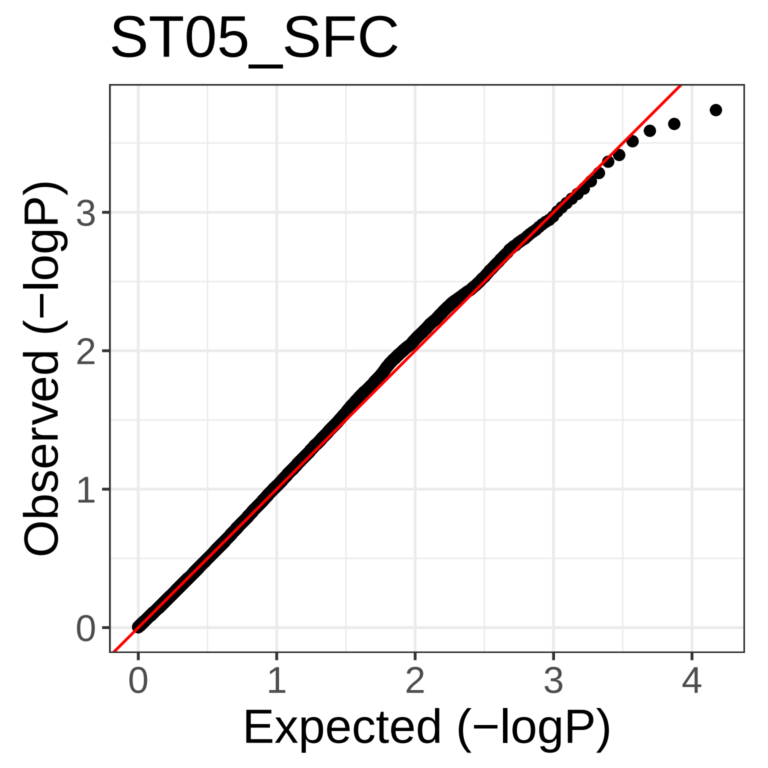

ST05_SFC
Observed (−logP)
Expected (−logP)
0
1
2
3
4

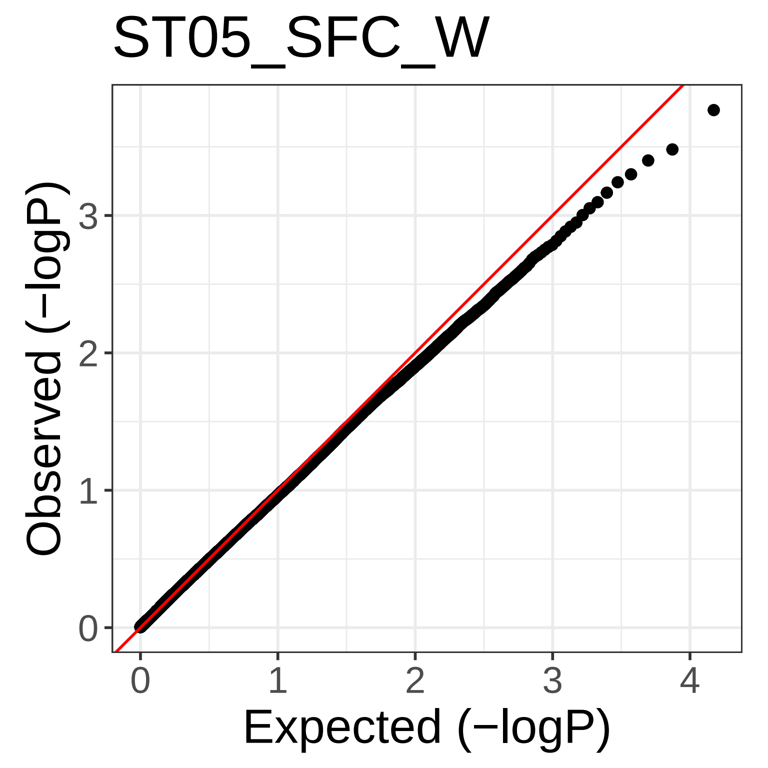
ST05_SFC_W
Observed (−logP)
Expected (−logP)
0
1
2
3
4

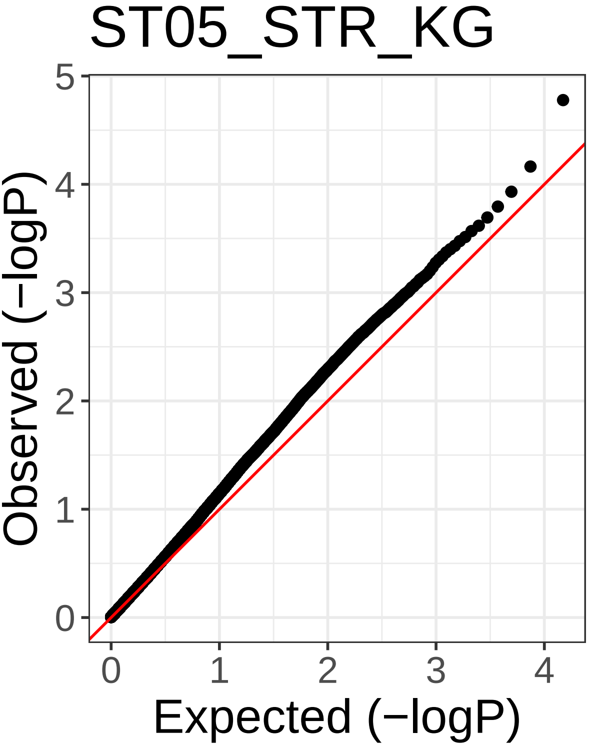

ST05_STR_KG
Observed (−logP)
Expected (−logP)
0
1
2
3
4
5

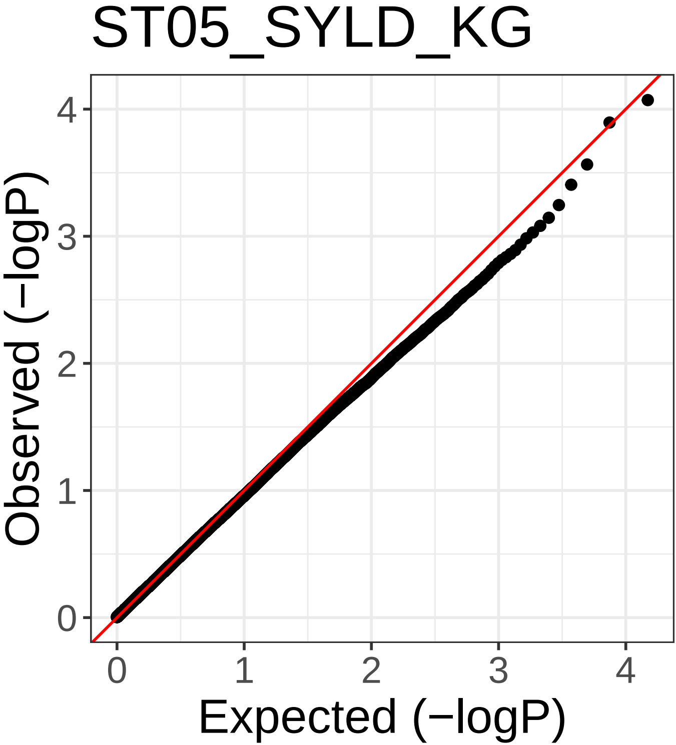

ST05_SYLD_KG
Observed (−logP)
Expected (−logP)
0
1
2
3
4
0
1
2
3
4

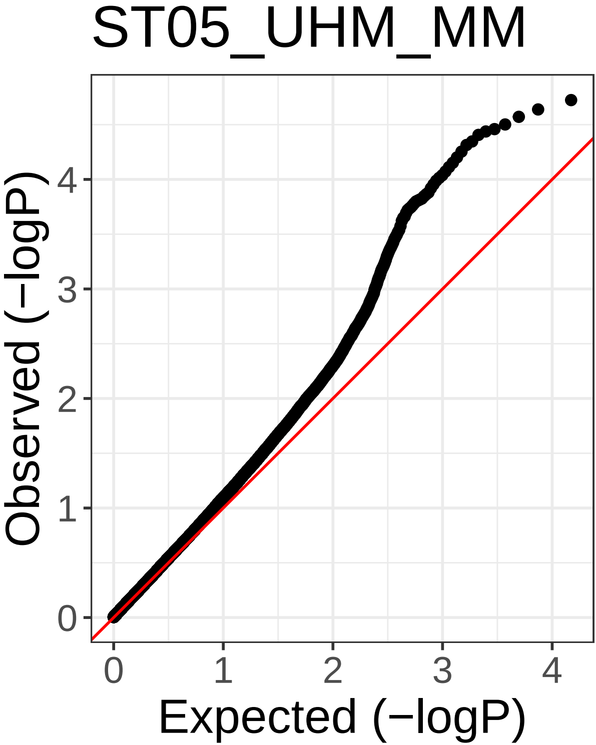

ST05_UHM_MM
Observed (−logP)
Expected (−logP)
0
1
2
3
4
0
1
2
3
4

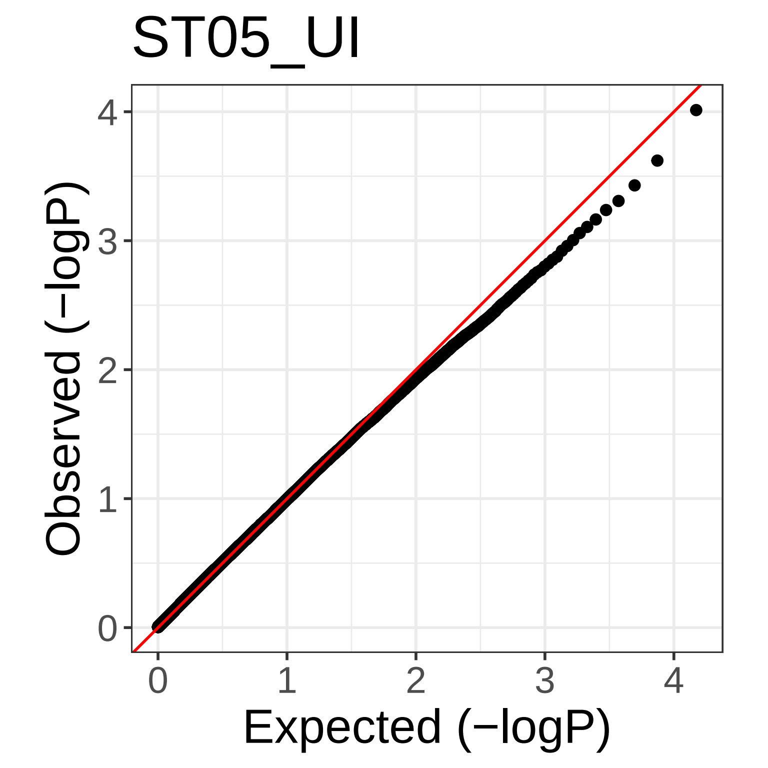

ST05_UI
Observed (−logP)
Expected (−logP)
0
1
2
3
4
0
1
2
3
4

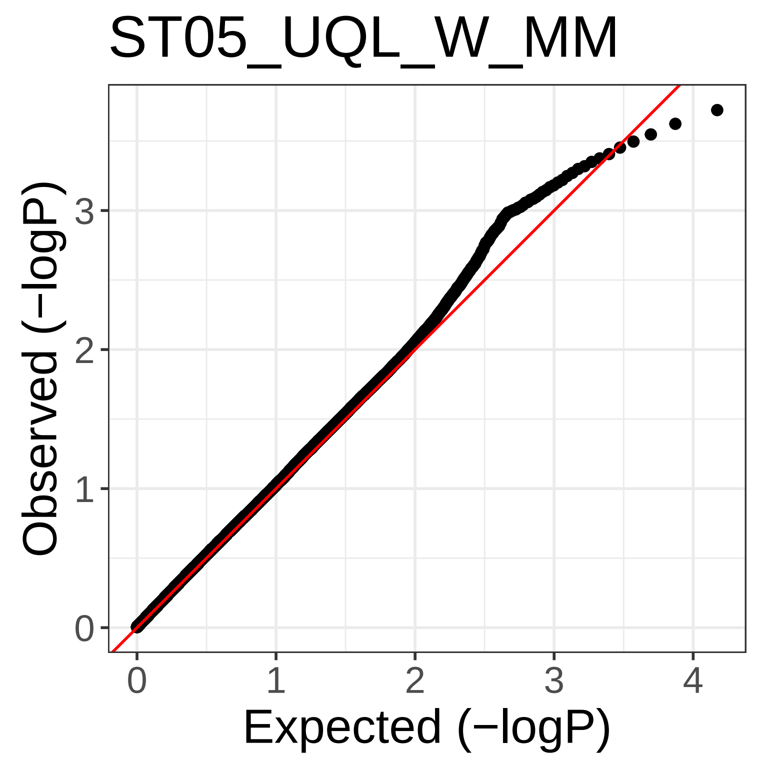
ST05_UQL_W_MM
Observed (−logP)
Expected (−logP)
0
1
2
3
0
1
2
3
4

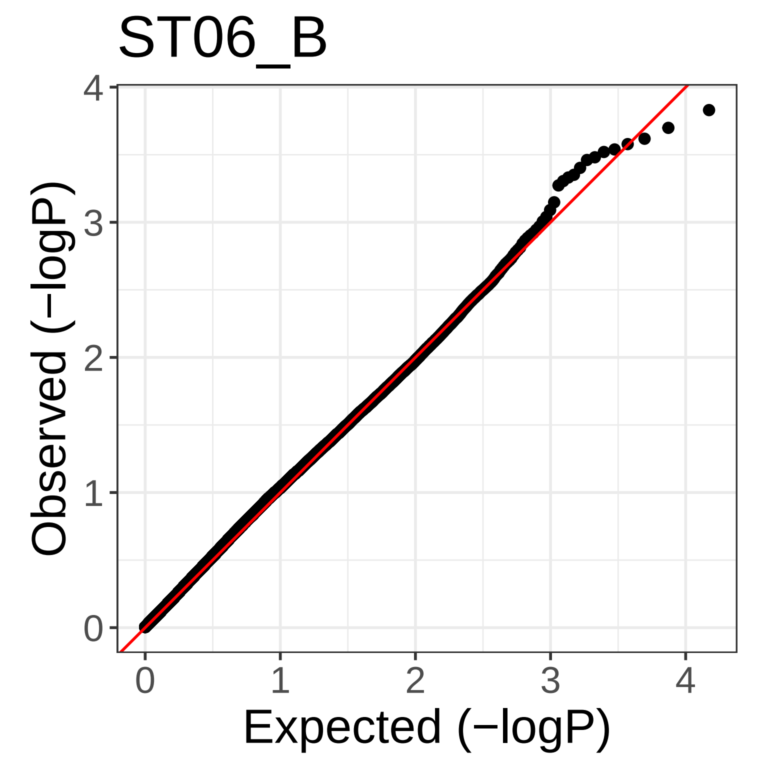
ST06_B
Observed (−logP)
Expected (−logP)
0
1
2
3
4
0
1
2
3
4

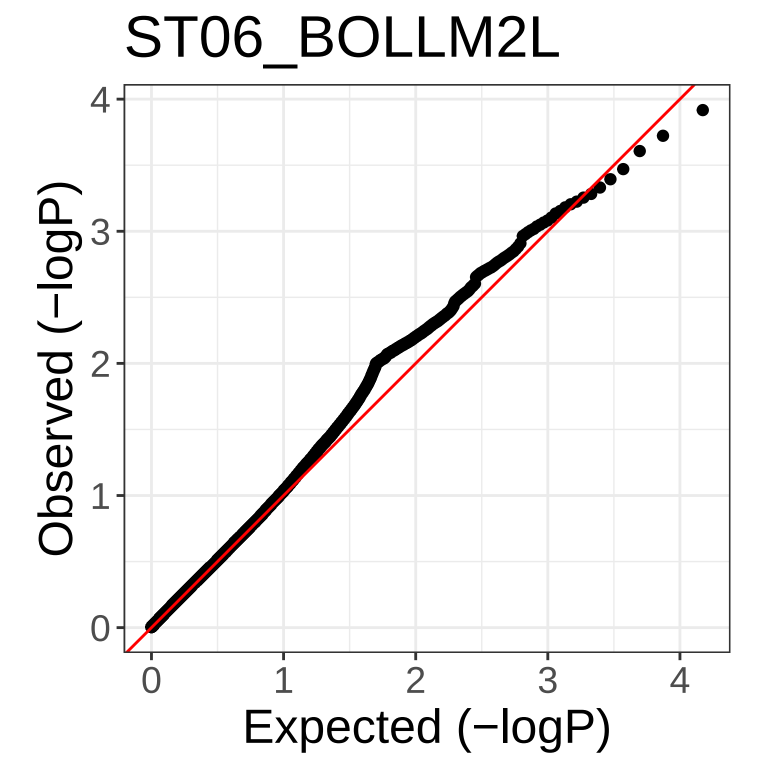

ST06_BOLLM2L
Observed (−logP)
Expected (−logP)
0
1
2
3
4

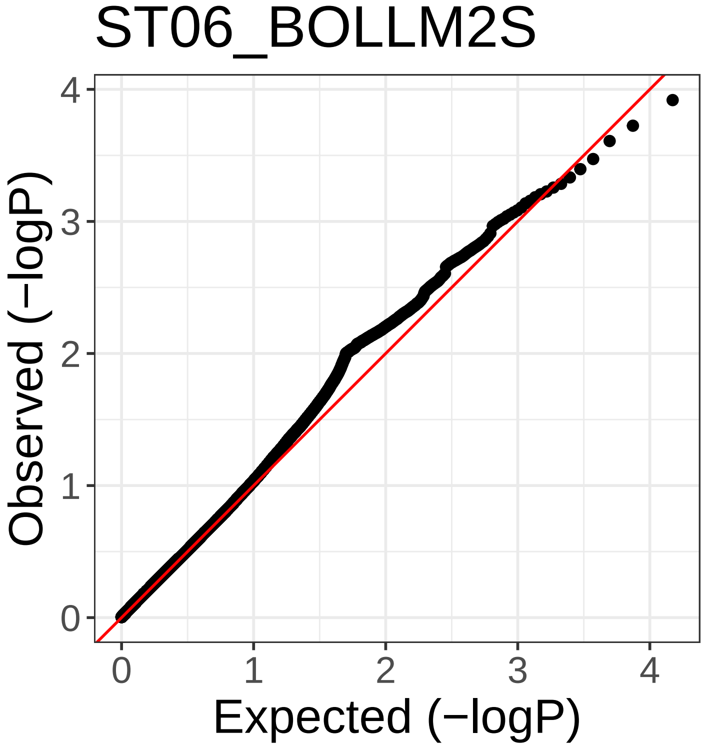

ST06_BOLLM2S
Observed (−logP)
Expected (−logP)
0
1
2
3
4
0
1
2
3
4

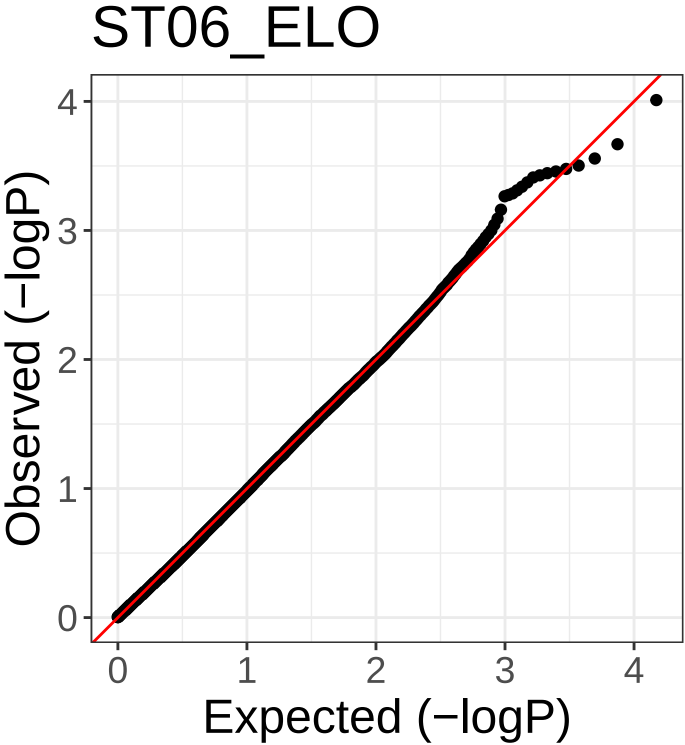
ST06_ELO
Observed (−logP)
Expected (−logP)
0
1
2
3
4
0
1
2
3
4

ST06_FINE_MTEX

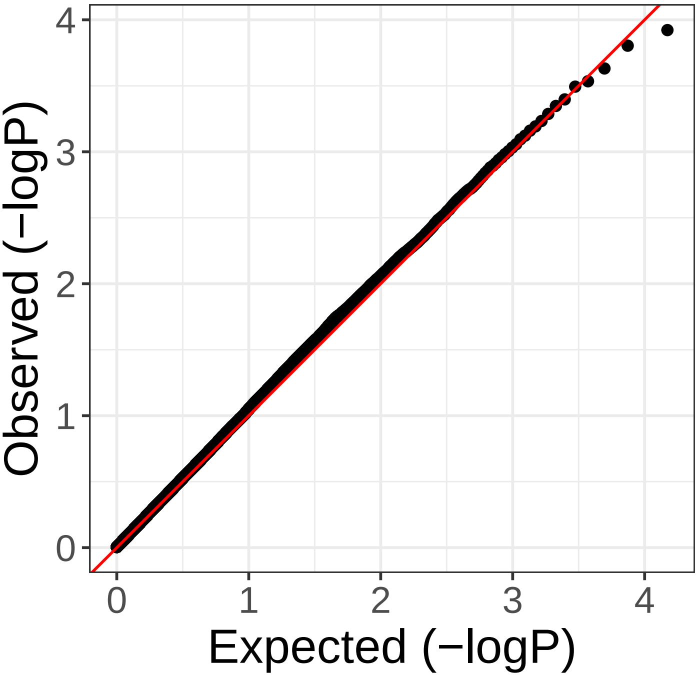

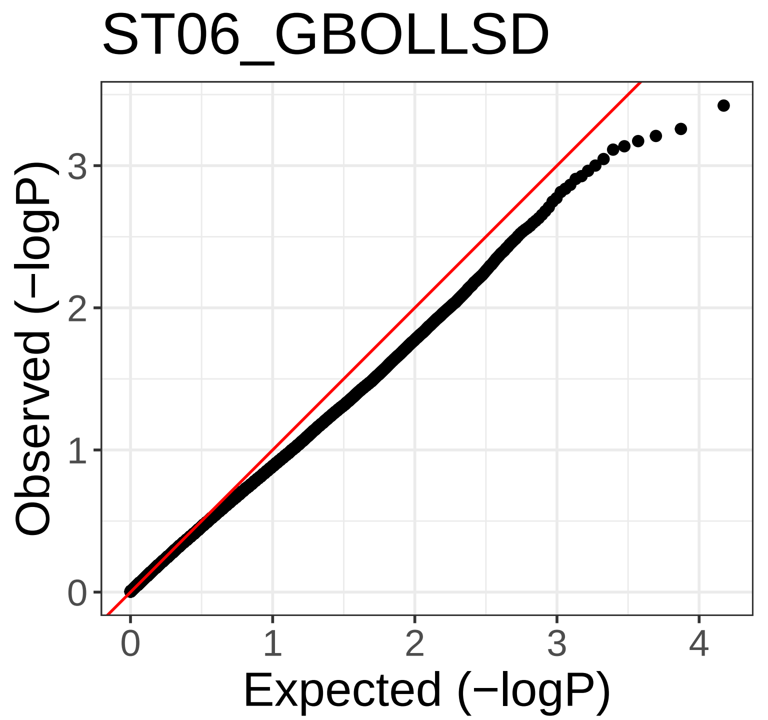
ST06_GBOLLSD
Observed (−logP)
Expected (−logP)
0
1
2
3
4

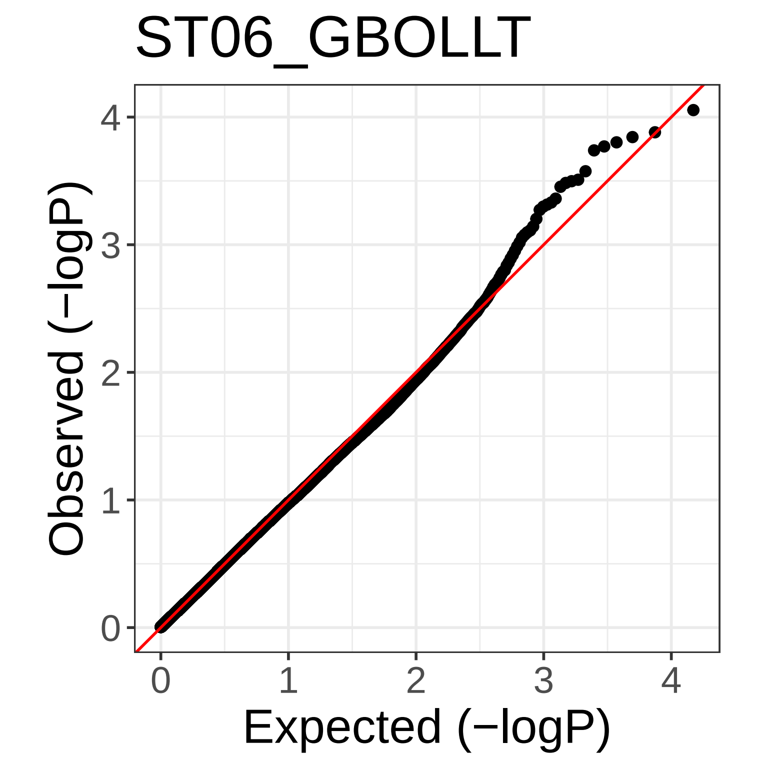
ST06_GBOLLT
Observed (−logP)
Expected (−logP)
0
1
2
3
4
0
1
2
3
4

ST06_GIN

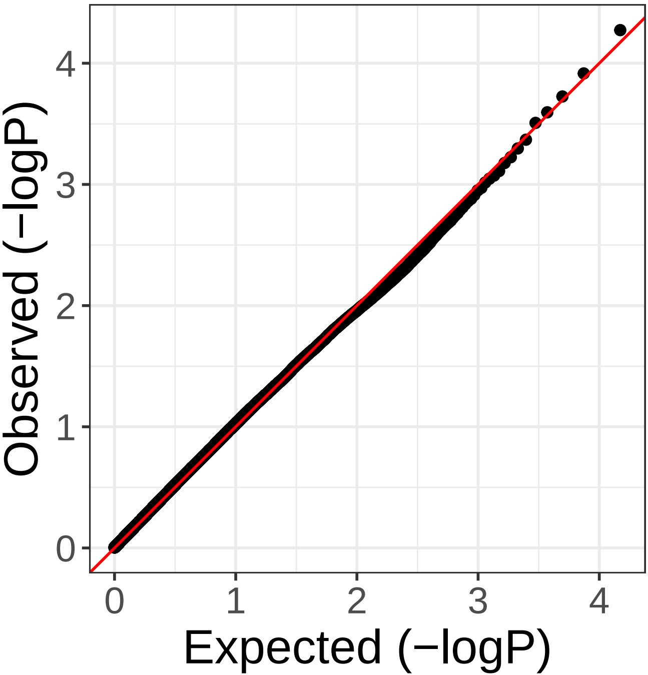

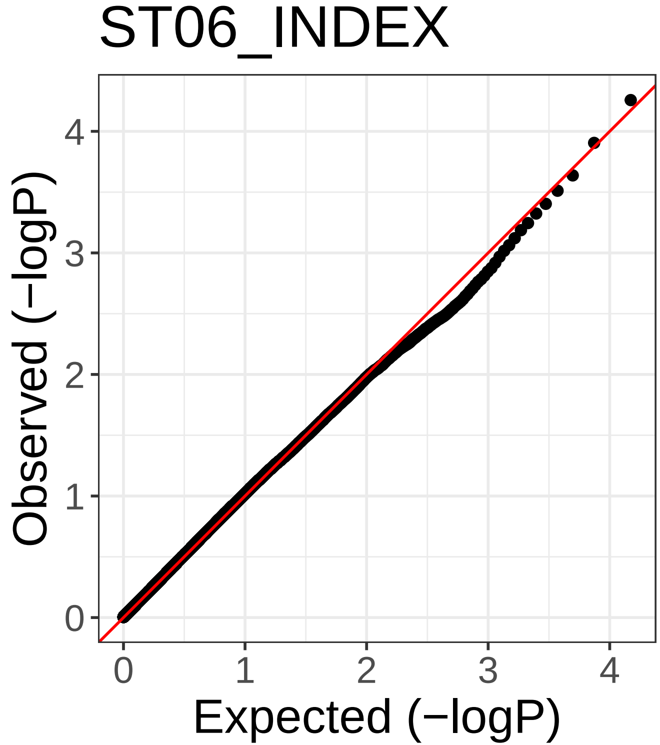
ST06_INDEX
Observed (−logP)
Expected (−logP)
0
1
2
3
4
0
1
2
3
4

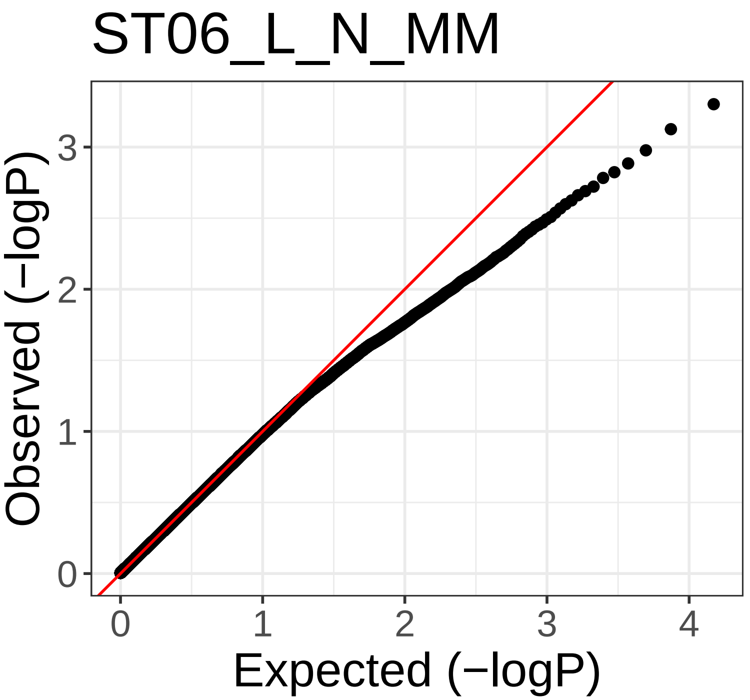
ST06_L_N_MM
Observed (−logP)
Expected (−logP)
0
1
2
3
4

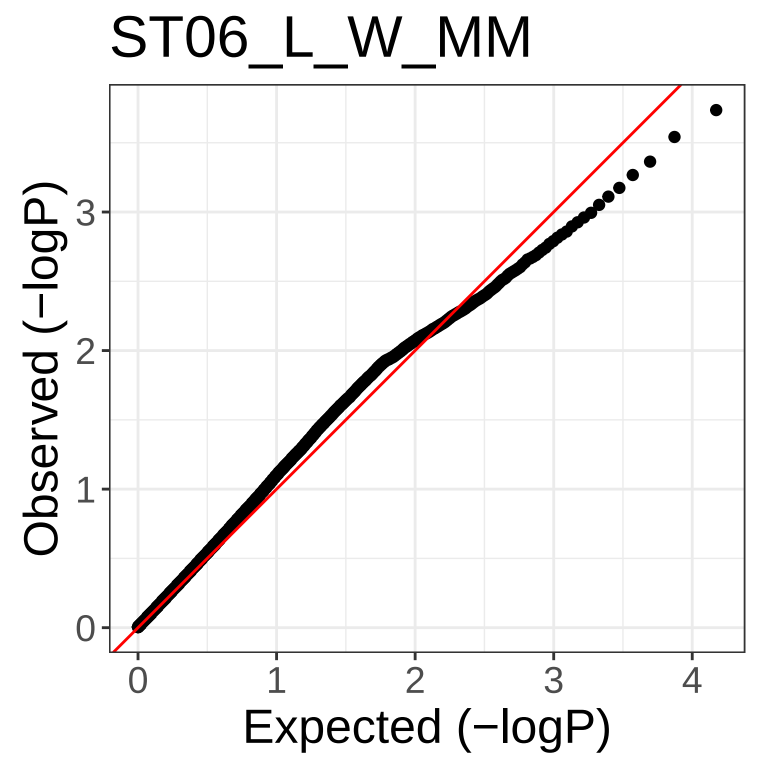
ST06_L_W_MM
Observed (−logP)
Expected (−logP)
0
1
2
3
4

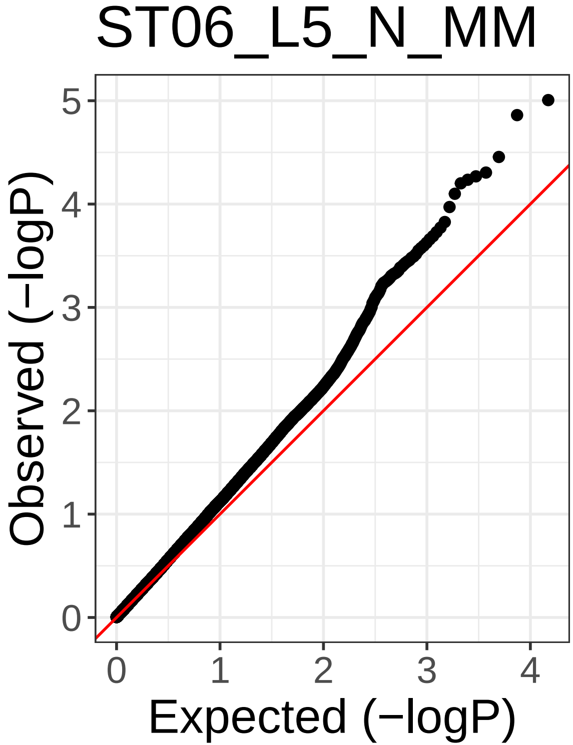
ST06_L5_N_MM
Observed (−logP)
Expected (−logP)
0
1
2
3
4
5
0
1
2
3
4

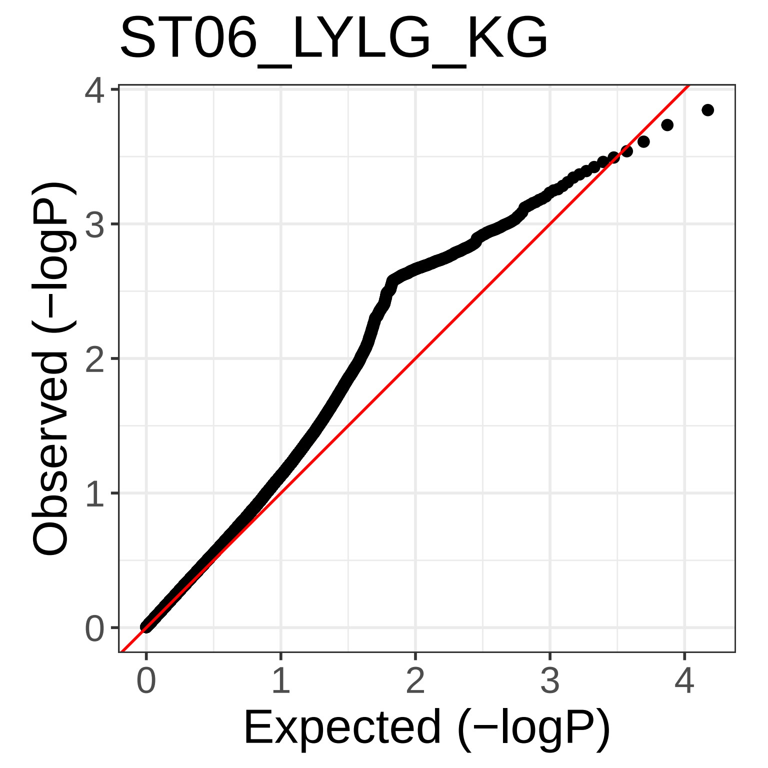
ST06_LYLG_KG
Observed (−logP)
Expected (−logP)
0
1
2
3
4
0
1
2
3
4

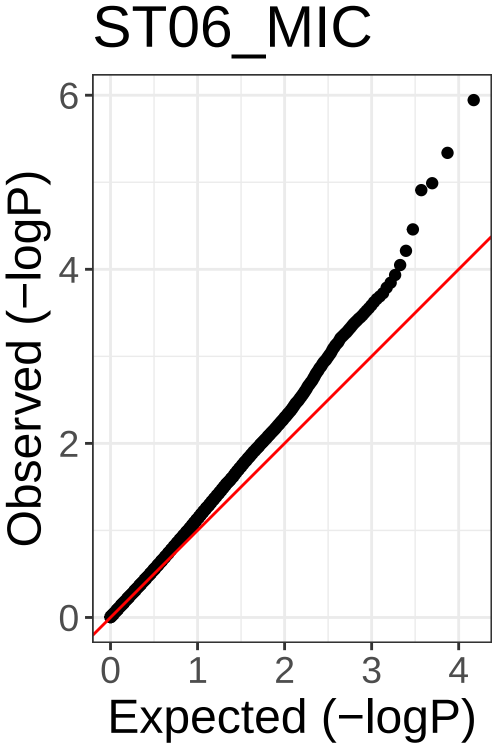
ST06_MIC
Observed (−logP)
Expected (−logP)
0
2
4
6
0
1
2
3
4

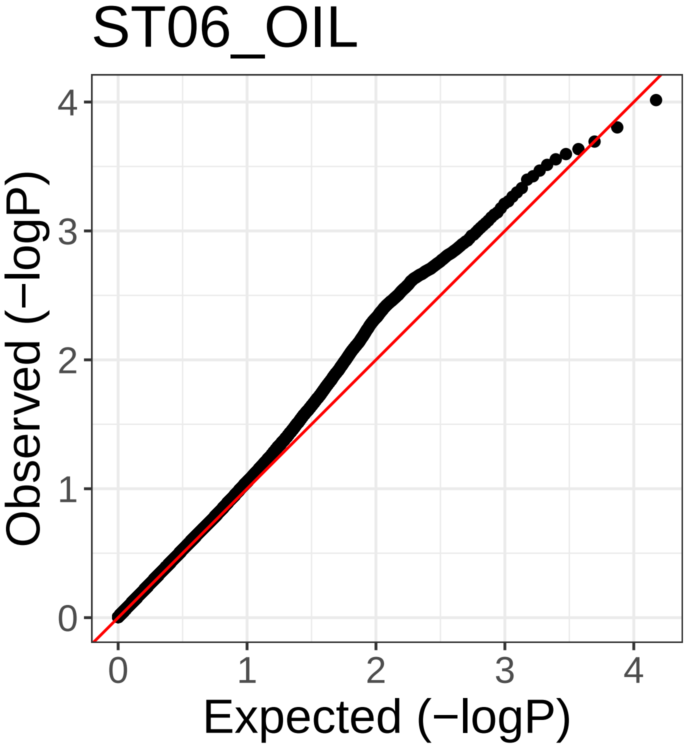

ST06_OIL
Observed (−logP)
Expected (−logP)
0
1
2
3
4
0
1
2
3
4

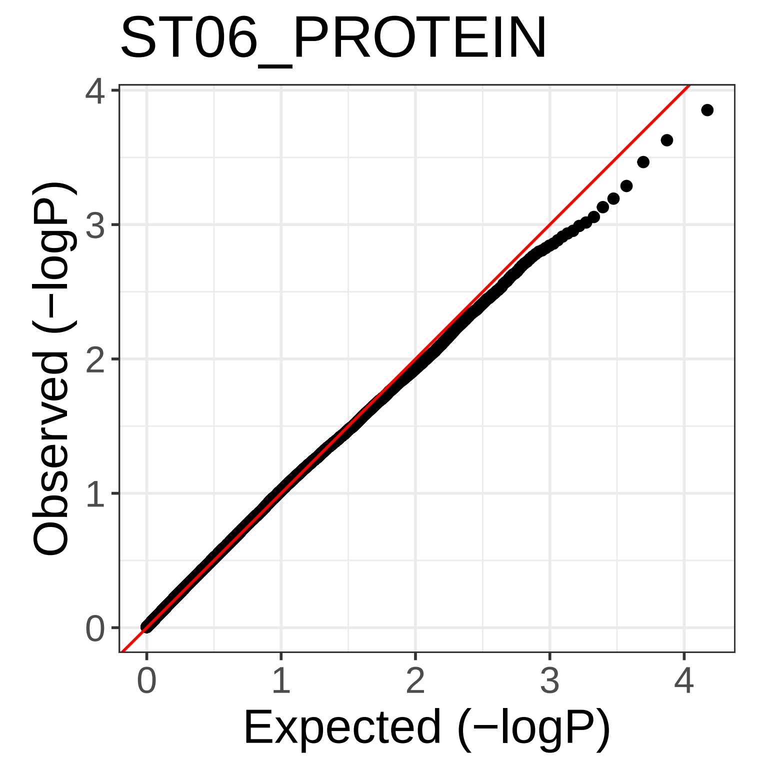

ST06_PROTEIN
Observed (−logP)
Expected (−logP)
0
1
2
3
4
0
1
2
3
4

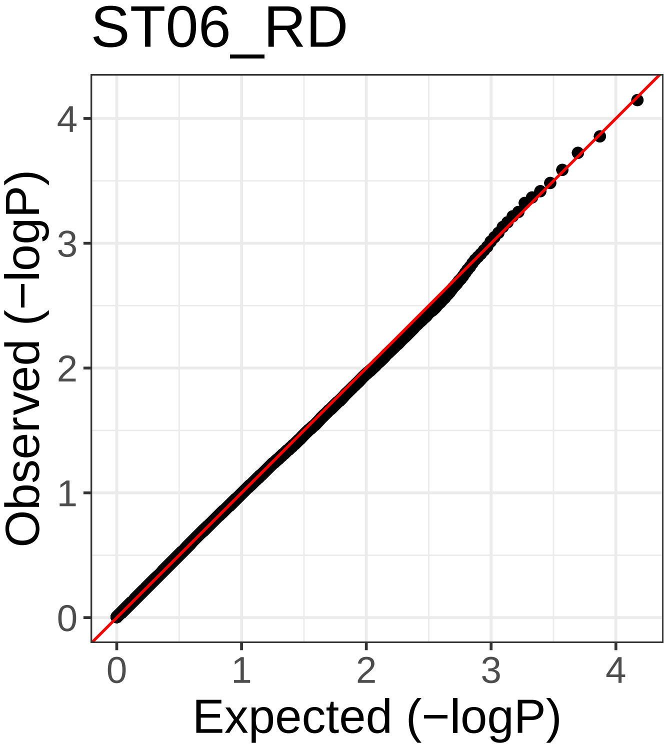
ST06_RD
Observed (−logP)
Expected (−logP)
0
1
2
3
4
0
1
2
3
4

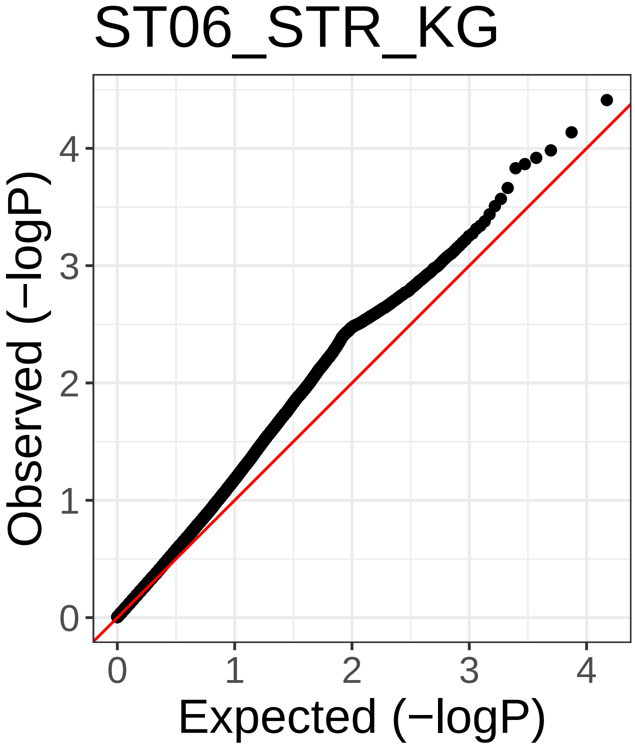

ST06_STR_KG
Observed (−logP)
Expected (−logP)
0
1
2
3
4

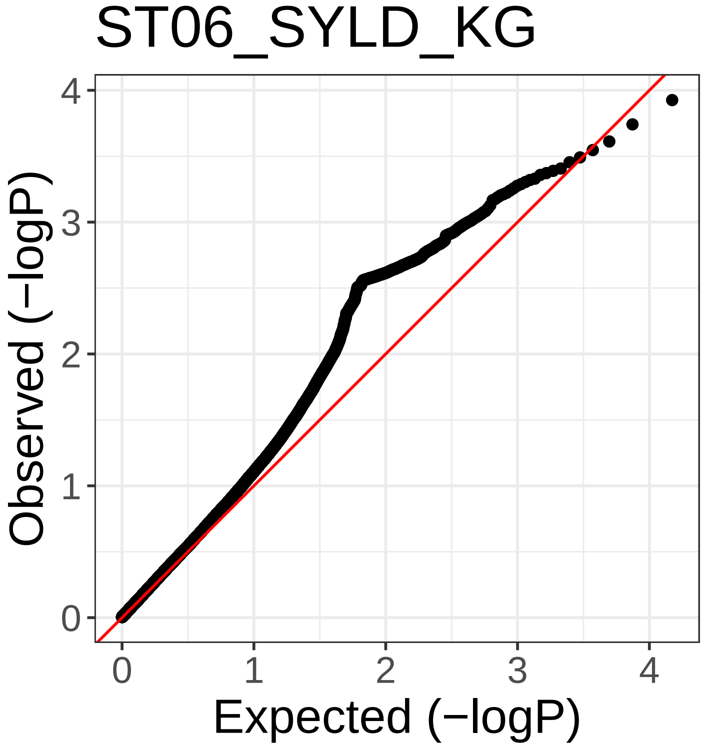

ST06_SYLD_KG
Observed (−logP)
Expected (−logP)
0
1
2
3
4
0
1
2
3
4

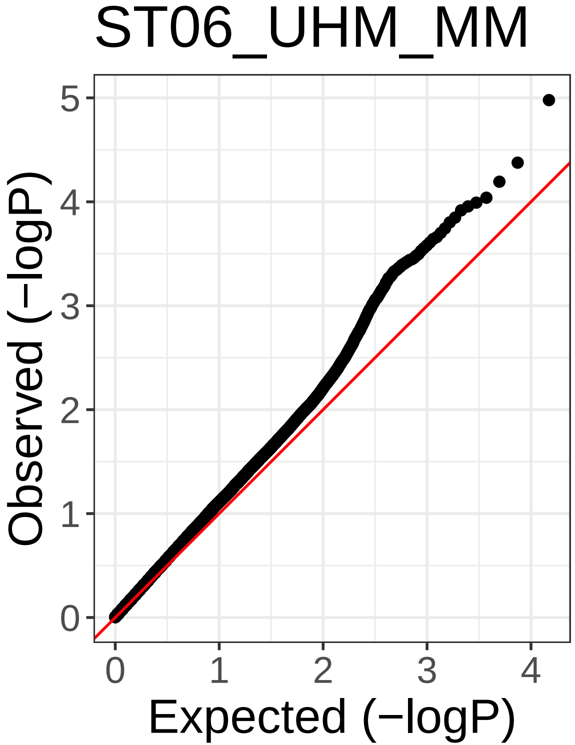

ST06_UHM_MM
Observed (−logP)
Expected (−logP)
0
1
2
3
4
5

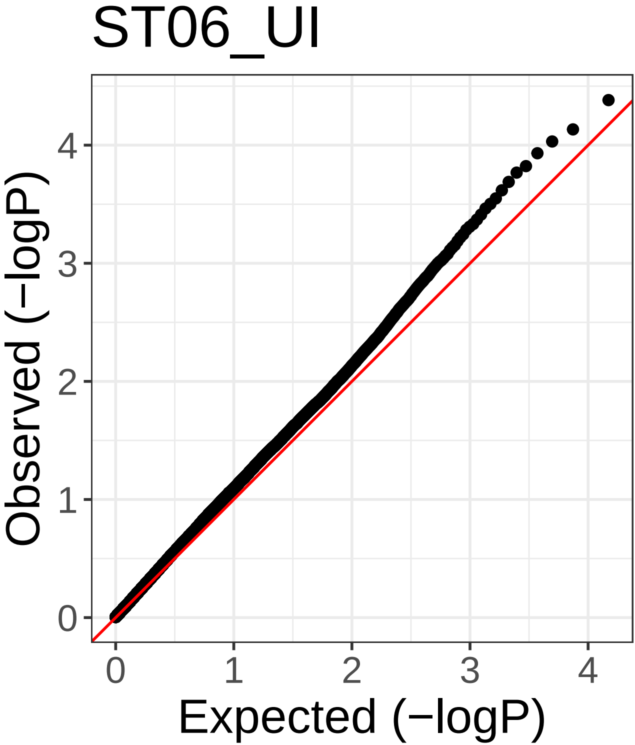
ST06_UI
Observed (−logP)
Expected (−logP)
0
1
2
3
4
0
1
2
3
4

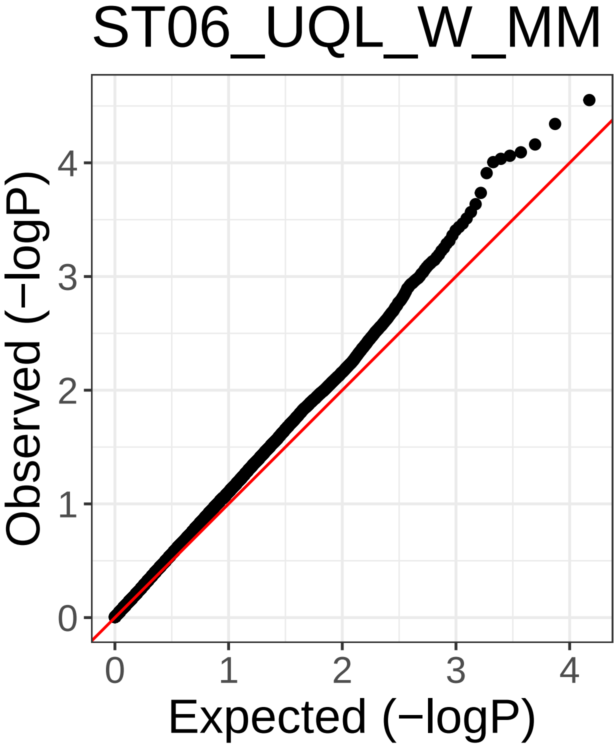

ST06_UQL_W_MM
Observed (−logP)
Expected (−logP)
0
1
2
3
4

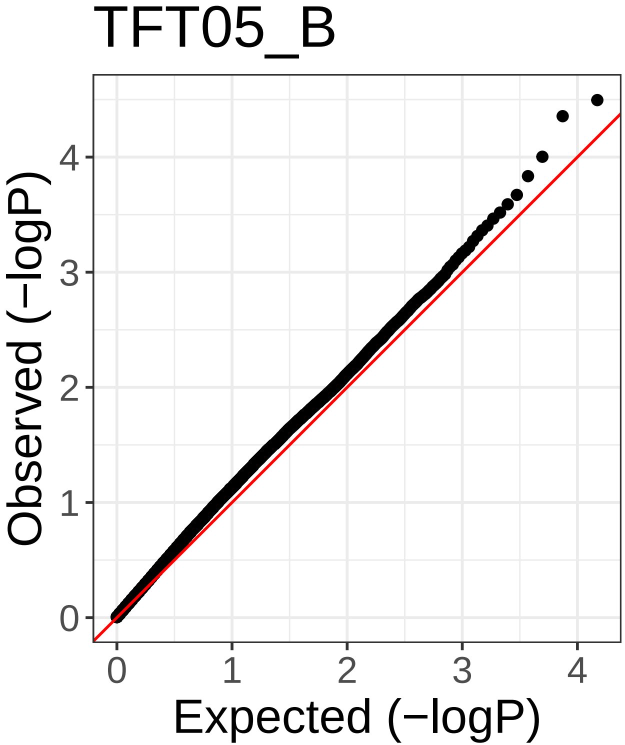
TFT05_B
Observed (−logP)
Expected (−logP)
0
1
2
3
4

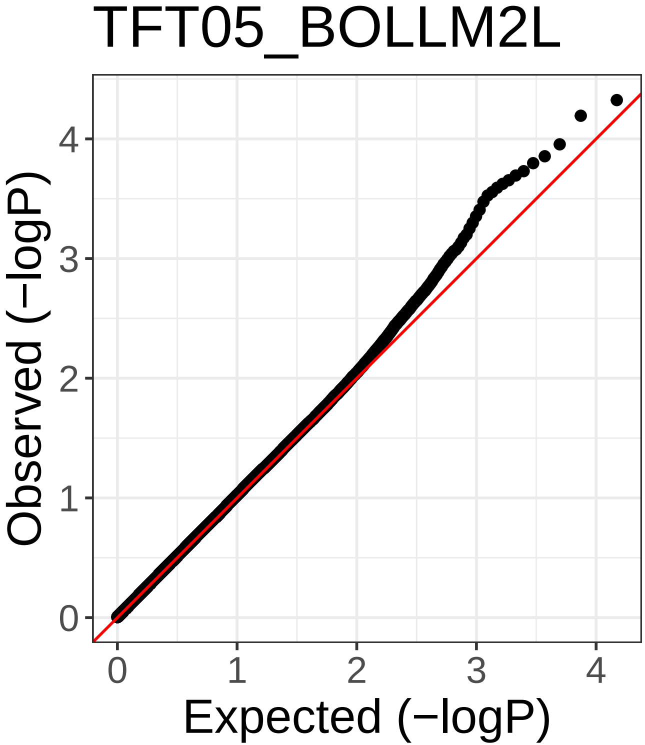
TFT05_BOLLM2L
Observed (−logP)
Expected (−logP)
0
1
2
3
4
0
1
2
3
4

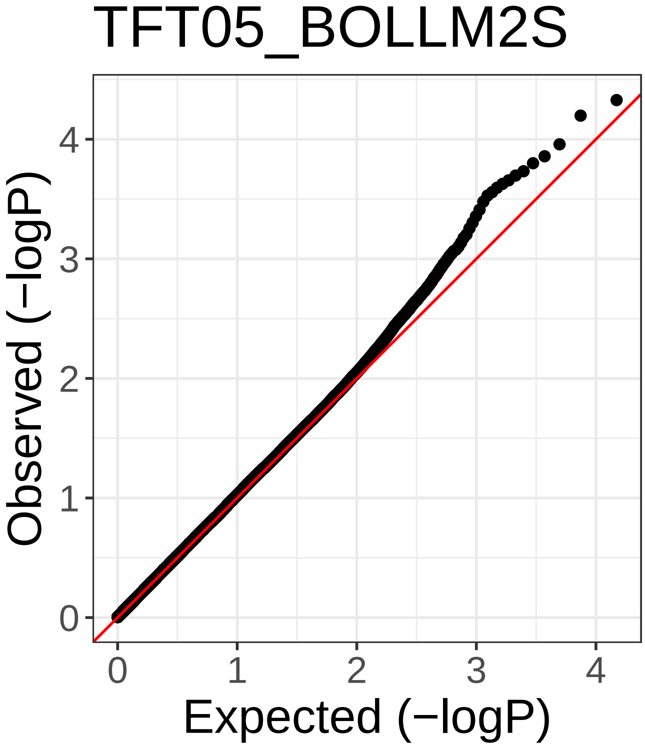

TFT05_BOLLM2S
Observed (−logP)
Expected (−logP)
0
1
2
3
4
0
1
2
3
4

TFT05_ELO

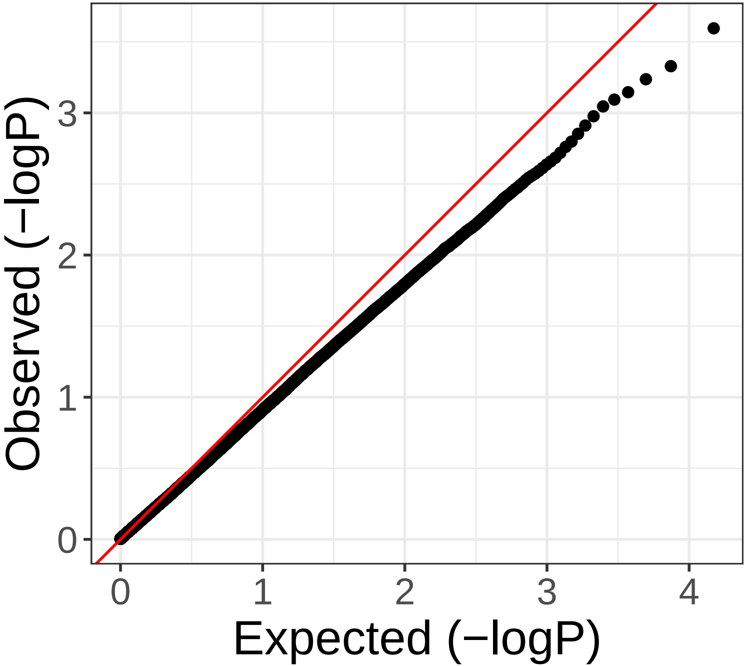

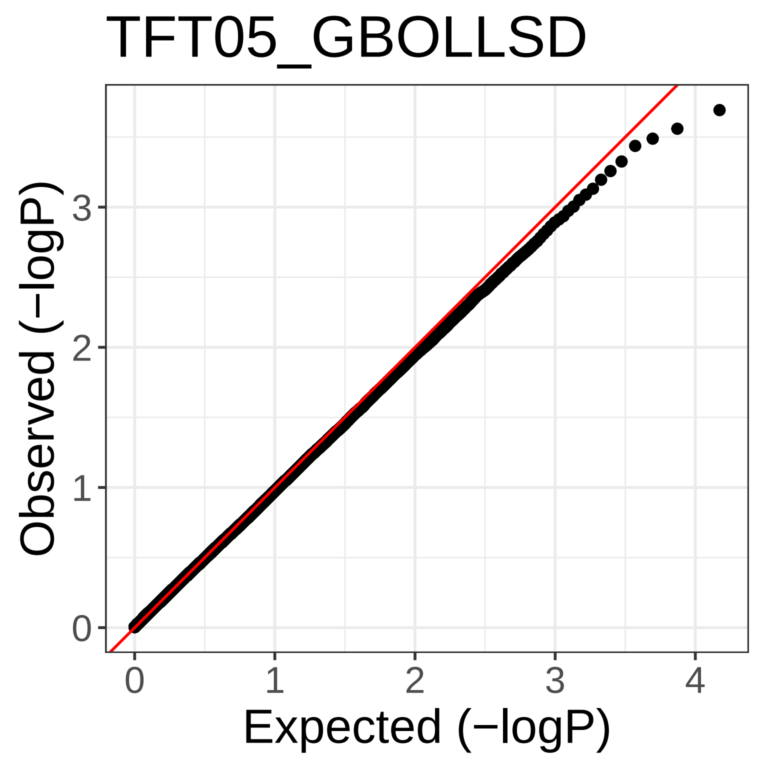

TFT05_GBOLLSD
Observed (−logP)
Expected (−logP)
0
1
2
3
4

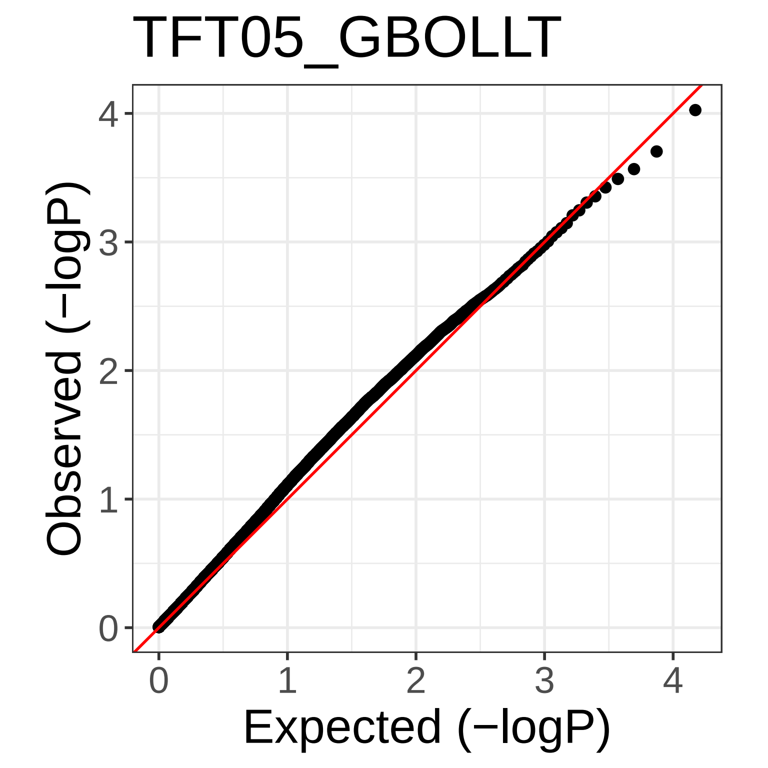

TFT05_GBOLLT
Observed (−logP)
Expected (−logP)
0
1
2
3
4
0
1
2
3
4

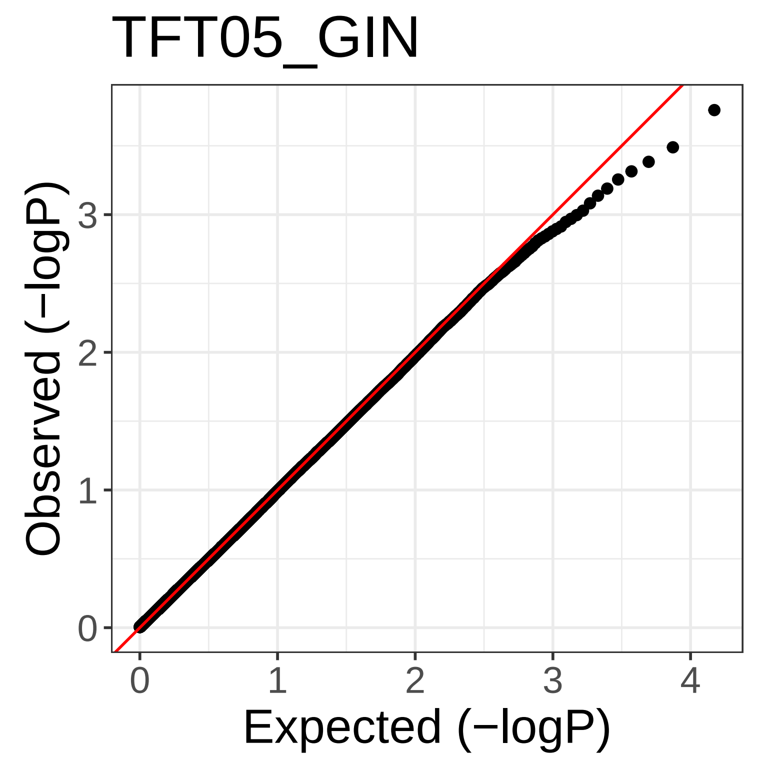

TFT05_GIN
Observed (−logP)
Expected (−logP)
0
1
2
3
4

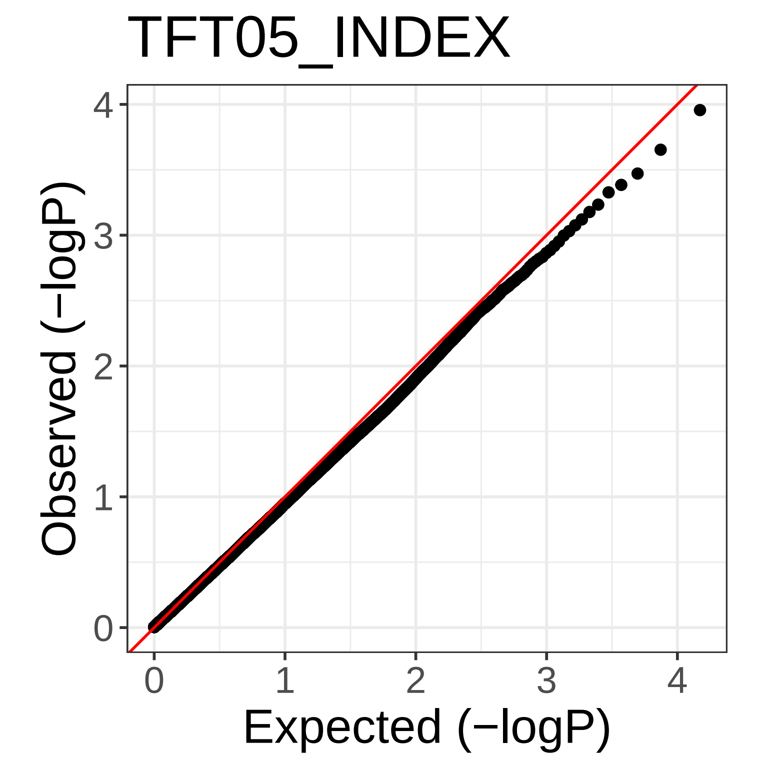

TFT05_INDEX
Observed (−logP)
Expected (−logP)
0
1
2
3
4
0
1
2
3
4

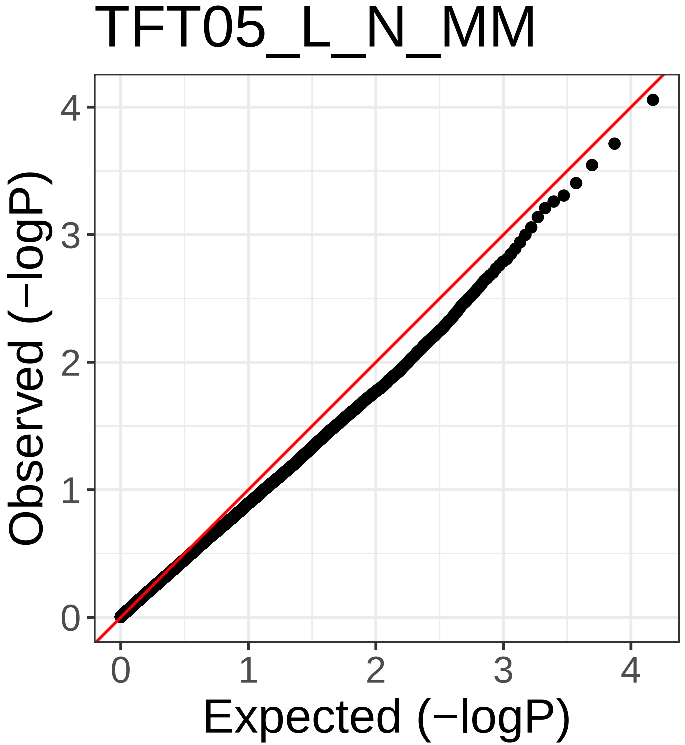
TFT05_L_N_MM
Observed (−logP)
Expected (−logP)
0
1
2
3
4
0
1
2
3
4

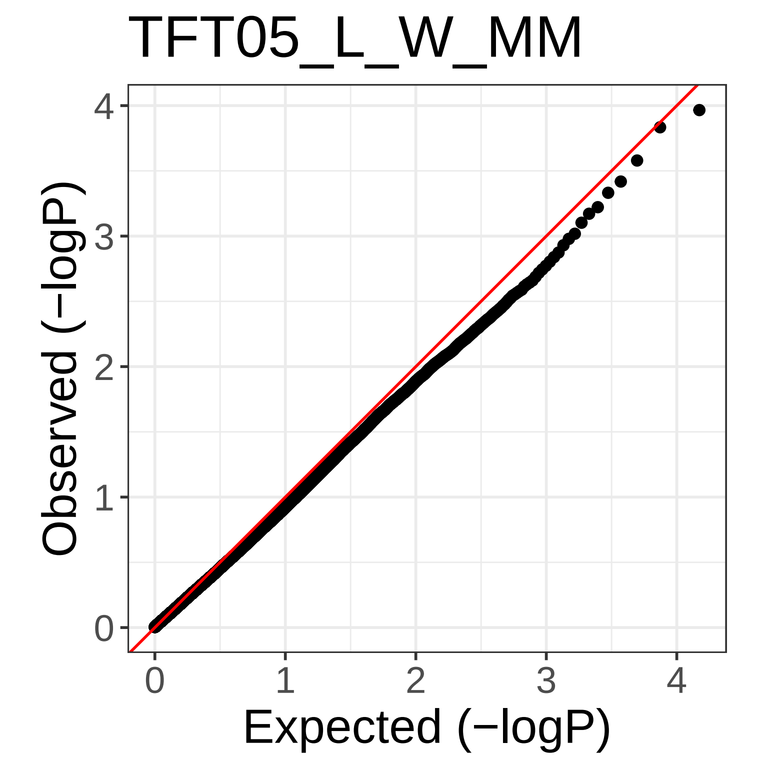
TFT05_L_W_MM
Observed (−logP)
Expected (−logP)
0
1
2
3
4
0
1
2
3
4

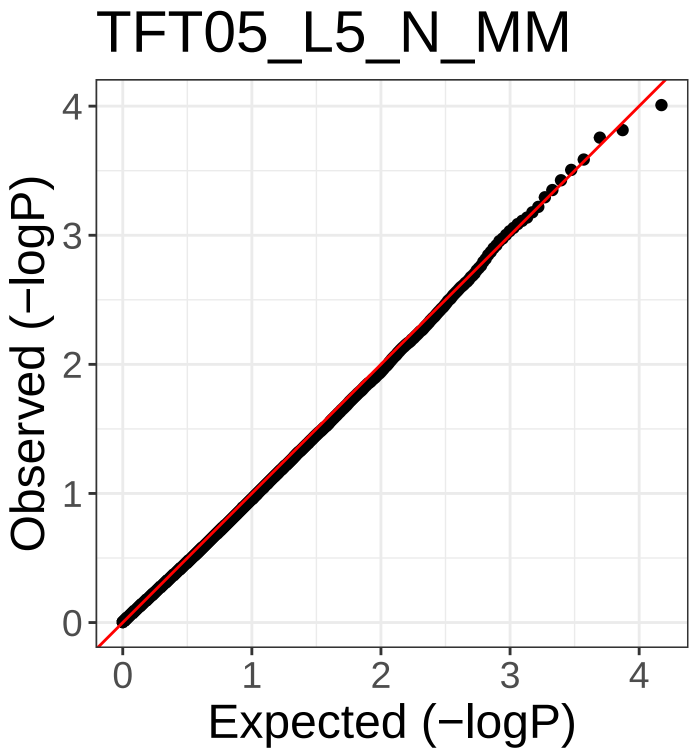

TFT05_L5_N_MM
Observed (−logP)
Expected (−logP)
0
1
2
3
4

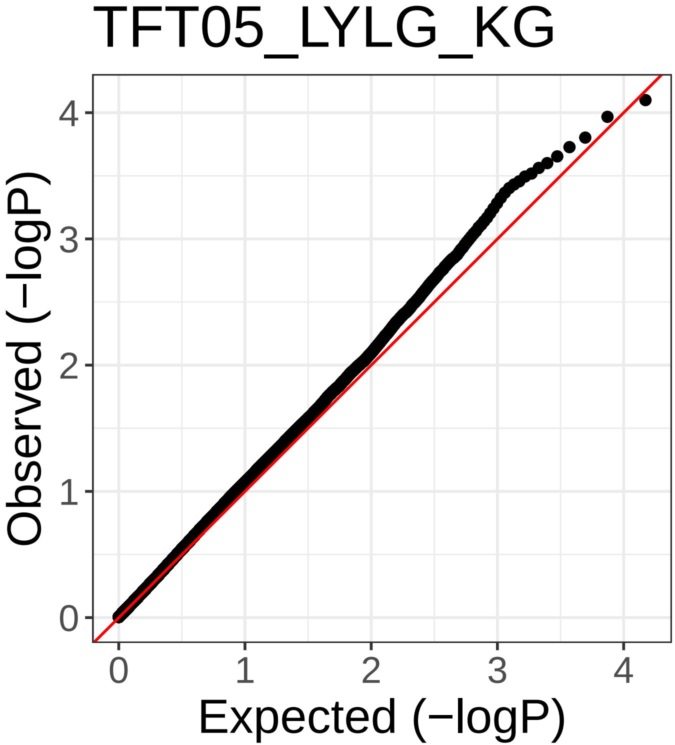
TFT05_LYLG_KG
Observed (−logP)
Expected (−logP)
0
1
2
3
4
0
1
2
3
4

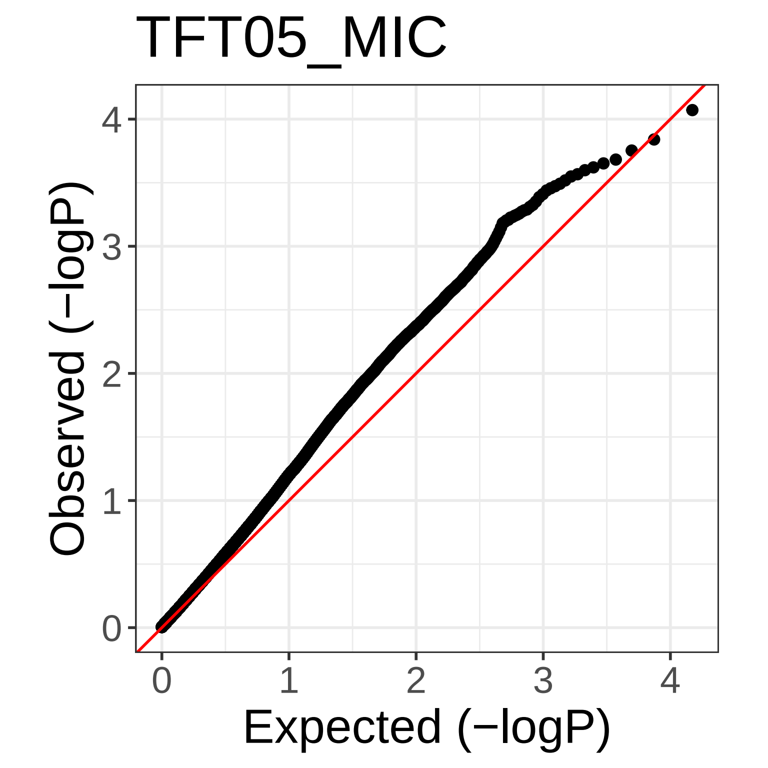

TFT05_MIC
Observed (−logP)
Expected (−logP)
0
1
2
3
4
0
1
2
3
4

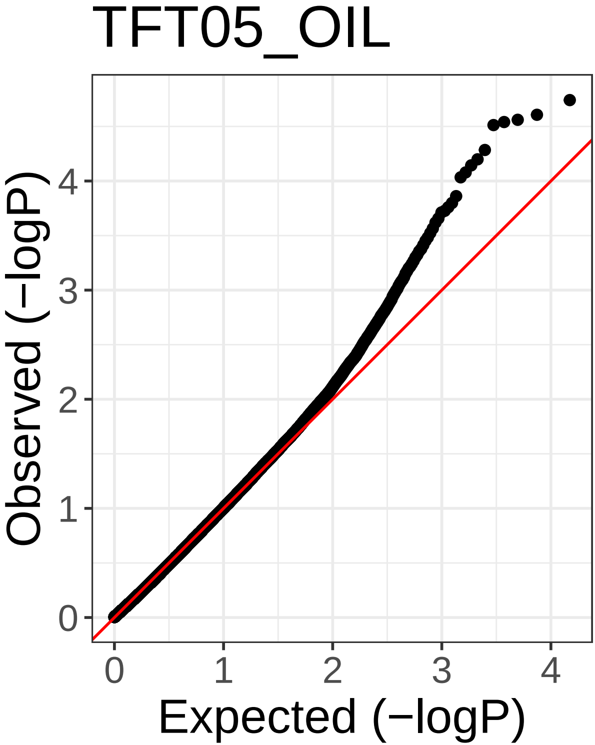
TFT05_OIL
Observed (−logP)
Expected (−logP)
0
1
2
3
4

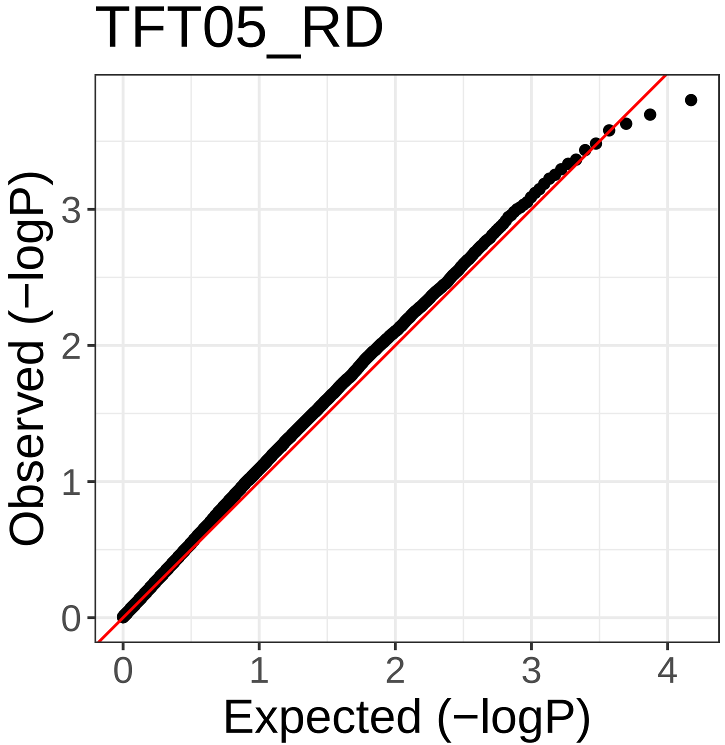
TFT05_RD
Observed (−logP)
Expected (−logP)
0
1
2
3
4

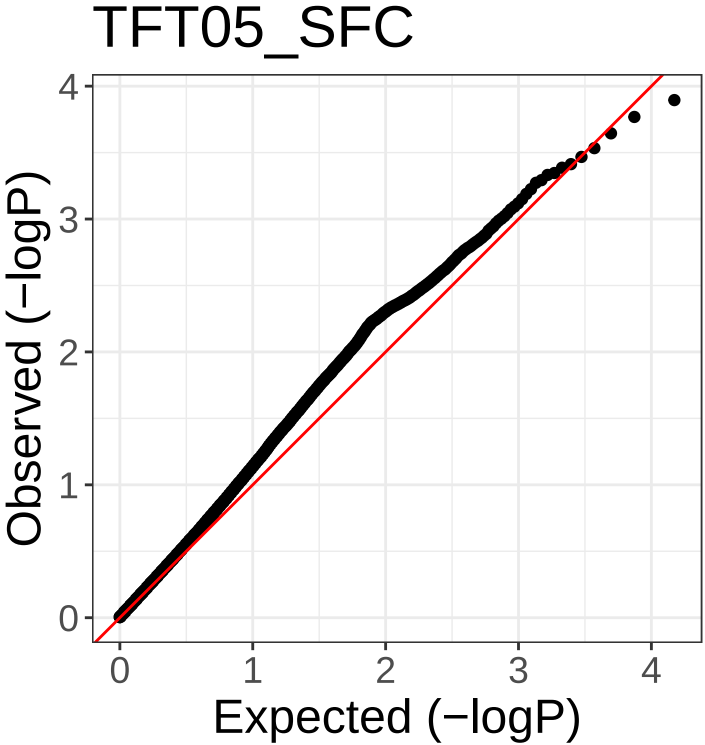
TFT05_SFC
Observed (−logP)
Expected (−logP)
0
1
2
3
4
0
1
2
3
4

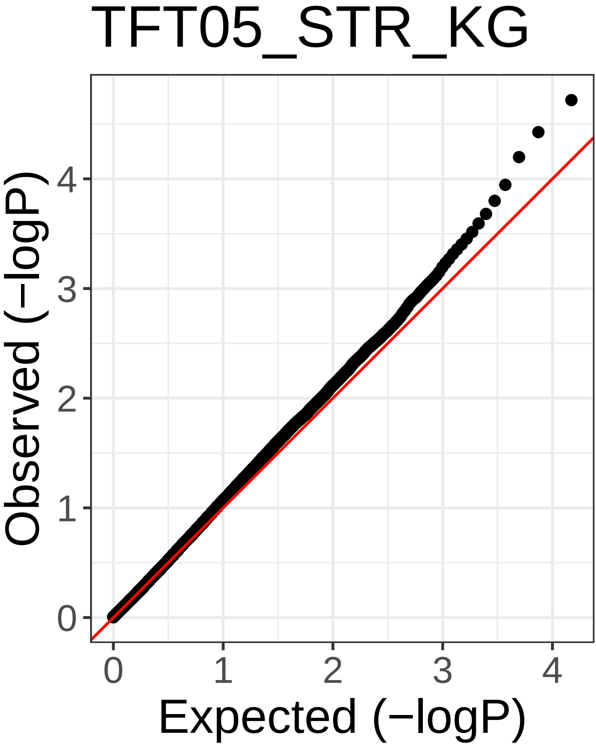
TFT05_STR_KG
Observed (−logP)
Expected (−logP)
0
1
2
3
4
0
1
2
3
4

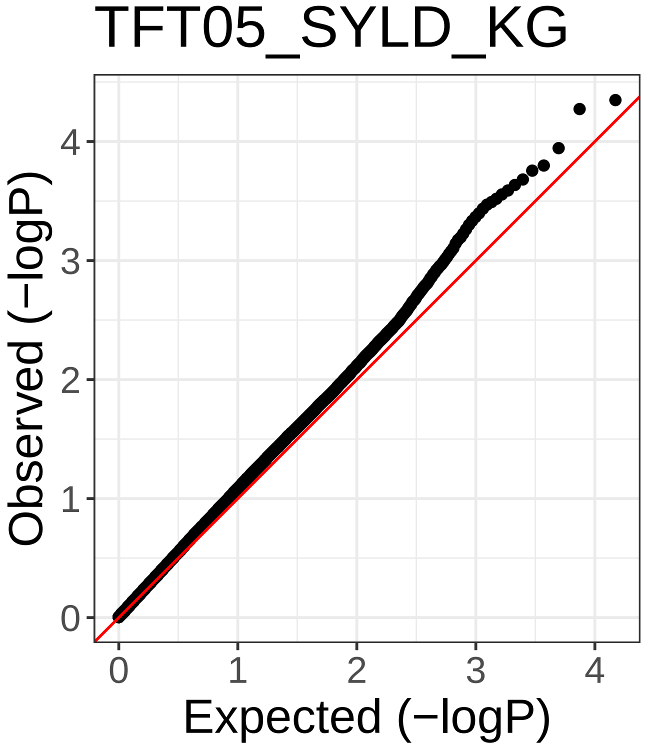

TFT05_SYLD_KG
Observed (−logP)
Expected (−logP)
0
1
2
3
4

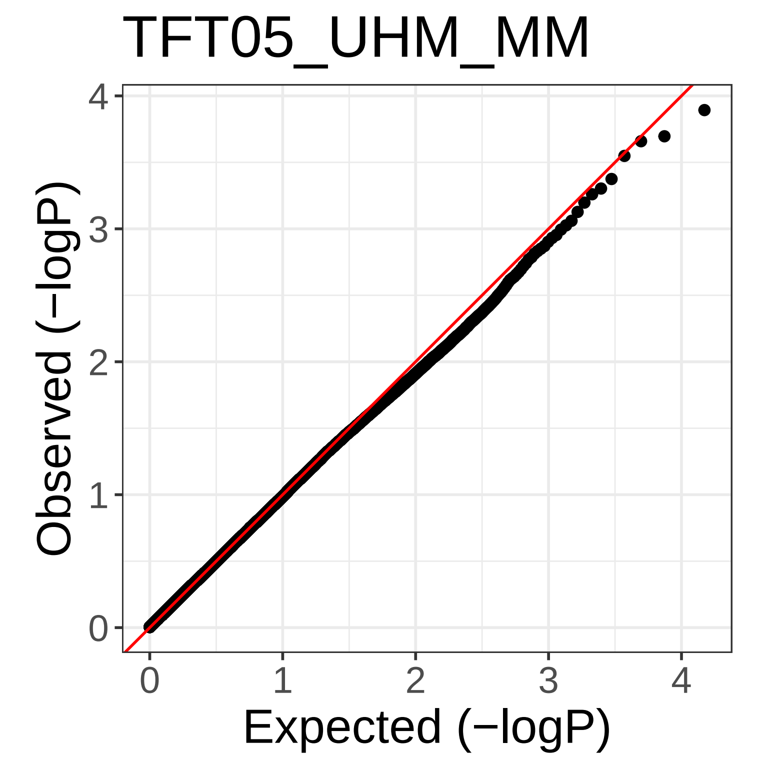
TFT05_UHM_MM
Observed (−logP)
Expected (−logP)
0
1
2
3
4
0
1
2
3
4

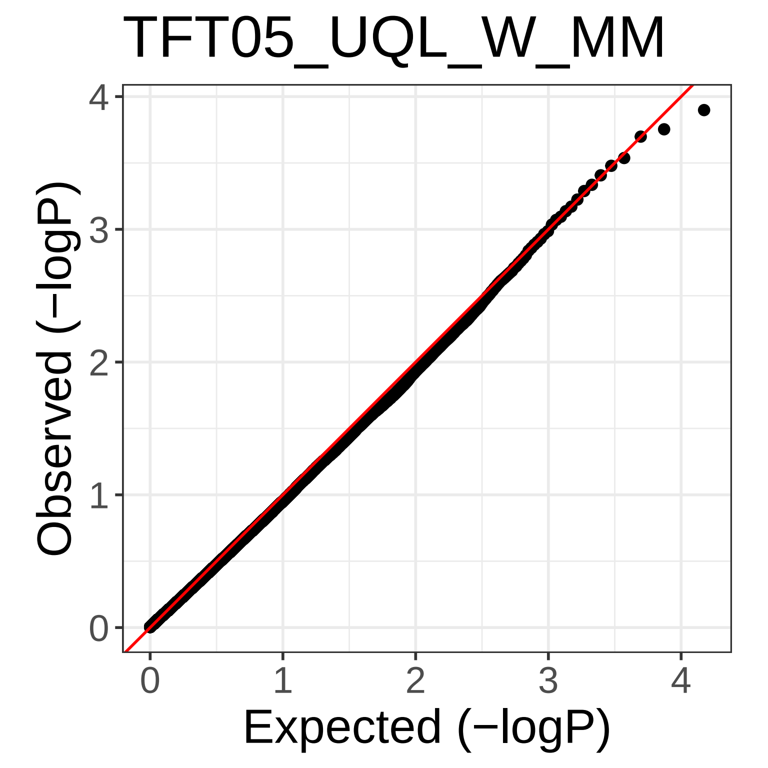

TFT05_UQL_W_MM
Observed (−logP)
Expected (−logP)
0
1
2
3
4
0
1
2
3
4

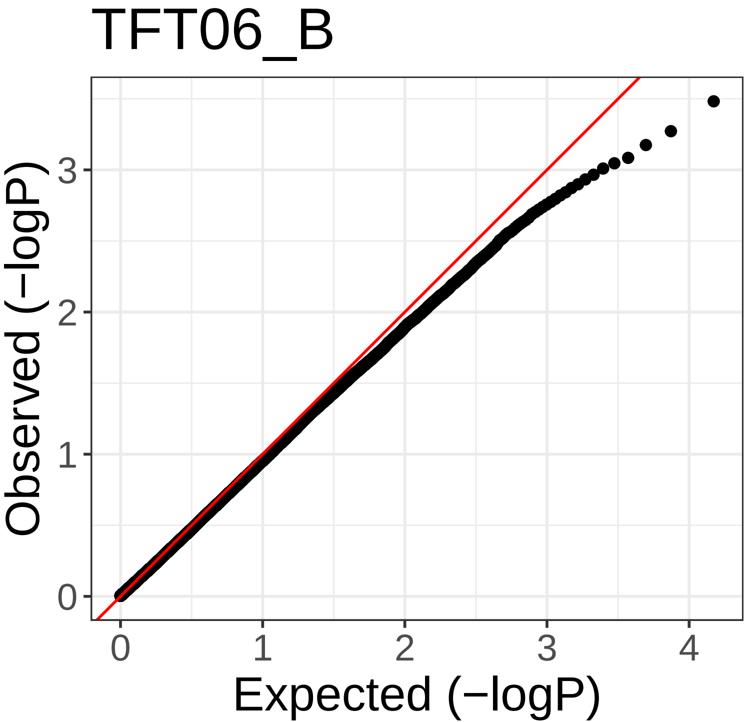
TFT06_B
Observed (−logP)
Expected (−logP)
0
1
2
3
0
1
2
3
4

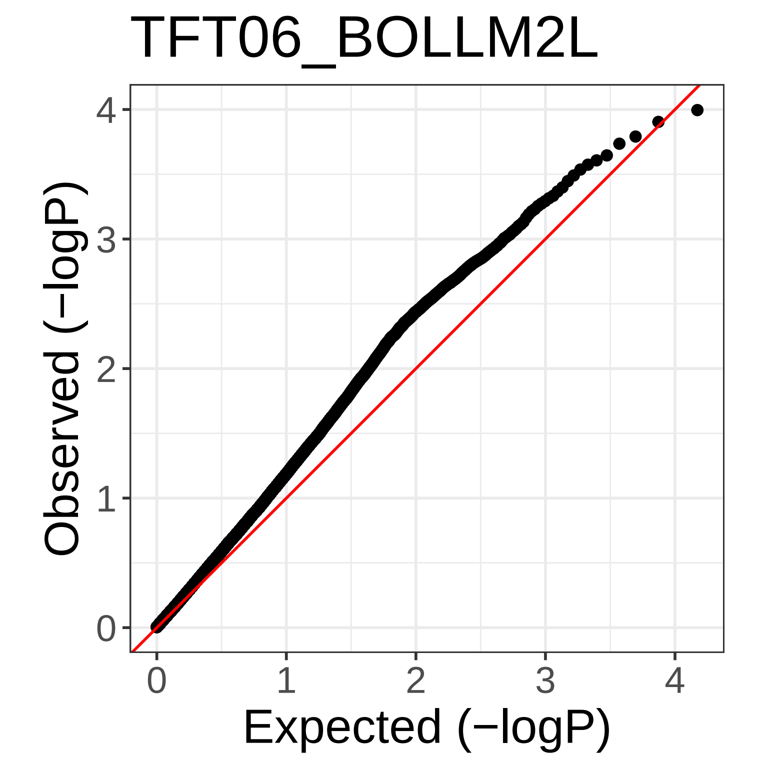
TFT06_BOLLM2L
Observed (−logP)
Expected (−logP)
0
1
2
3
4
0
1
2
3
4

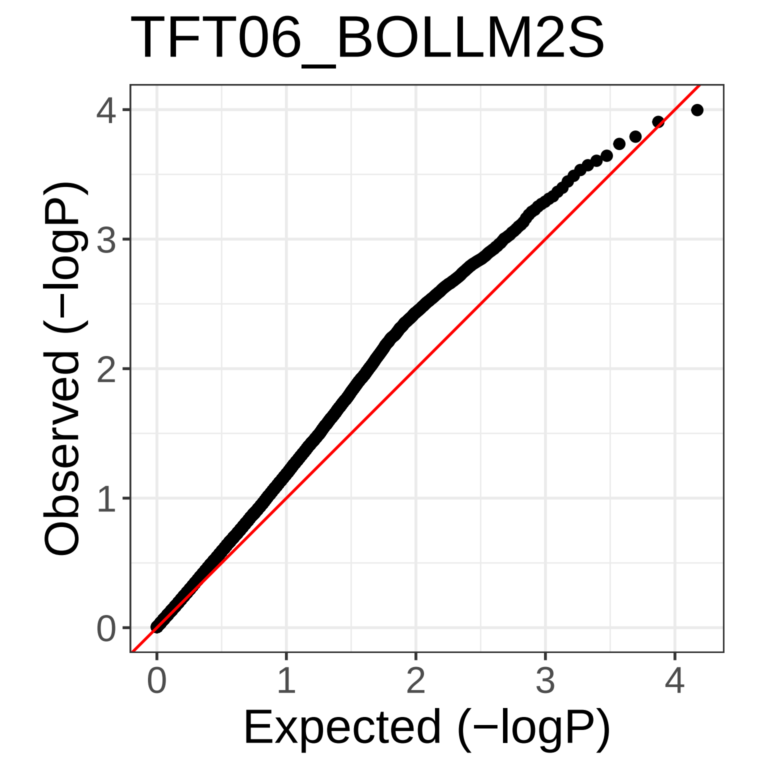

TFT06_BOLLM2S
Observed (−logP)
Expected (−logP)
0
1
2
3
4
0
1
2
3
4

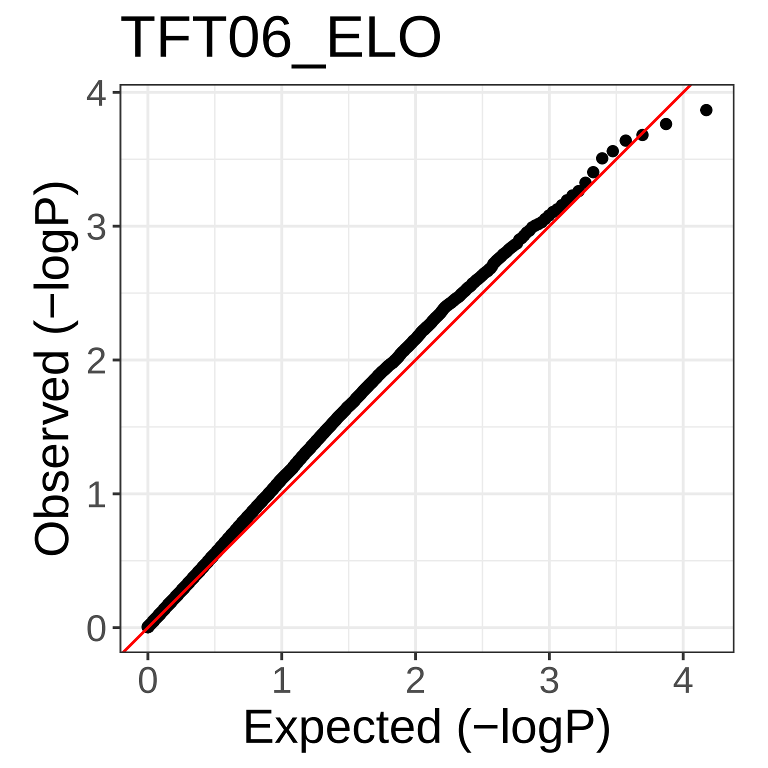
TFT06_ELO
Observed (−logP)
Expected (−logP)
0
1
2
3
4
0
1
2
3
4

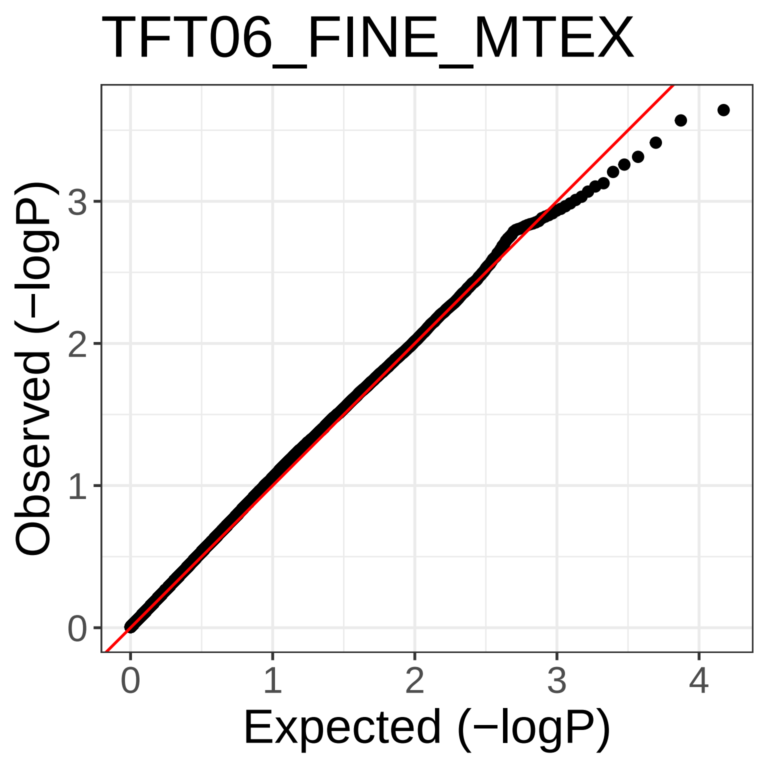
TFT06_FINE_MTEX
Observed (−logP)
Expected (−logP)
0
1
2
3
0
1
2
3
4

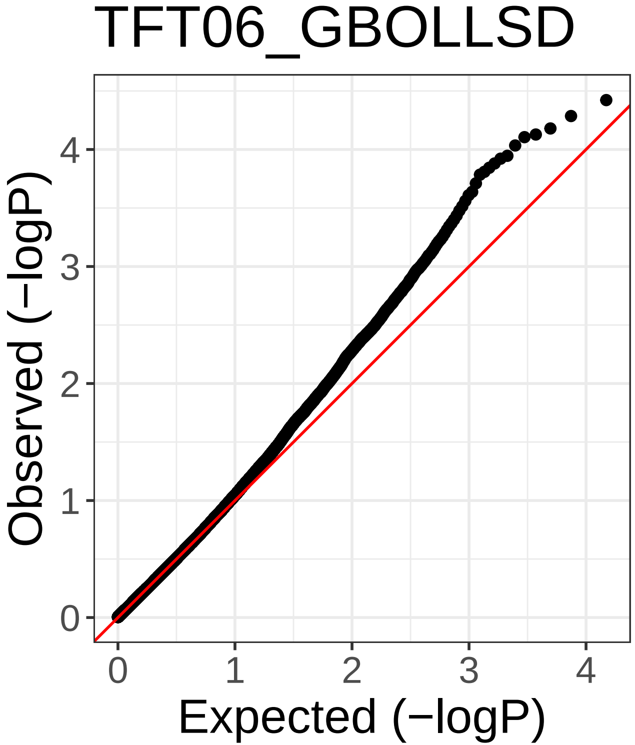
TFT06_GBOLLSD
Observed (−logP)
Expected (−logP)
0
1
2
3
4
0
1
2
3
4

TFT06_GBOLLT

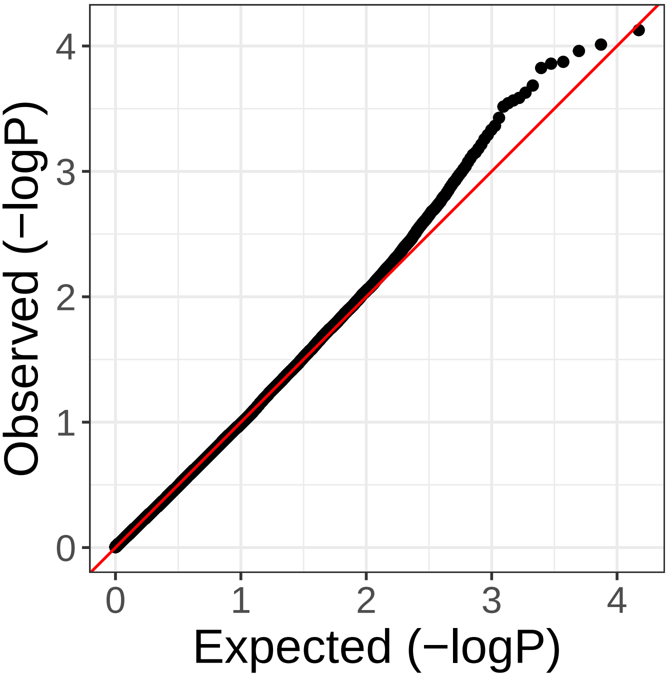

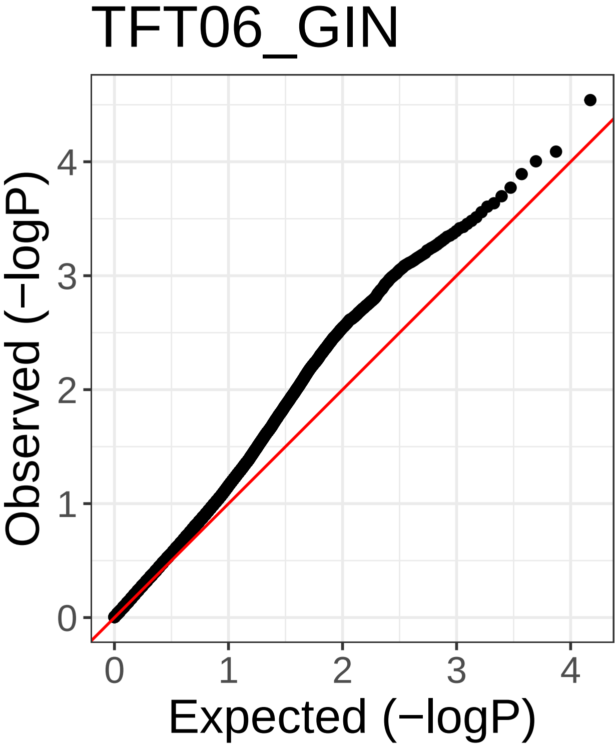

TFT06_GIN
Observed (−logP)
Expected (−logP)
0
1
2
3
4
0
1
2
3
4

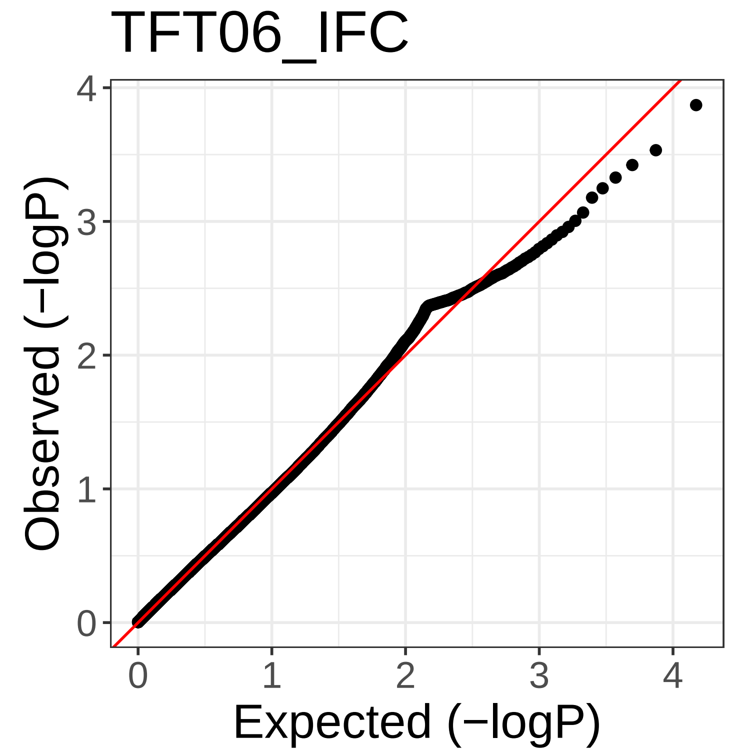

TFT06_IFC
Observed (−logP)
Expected (−logP)
0
1
2
3
4
0
1
2
3
4

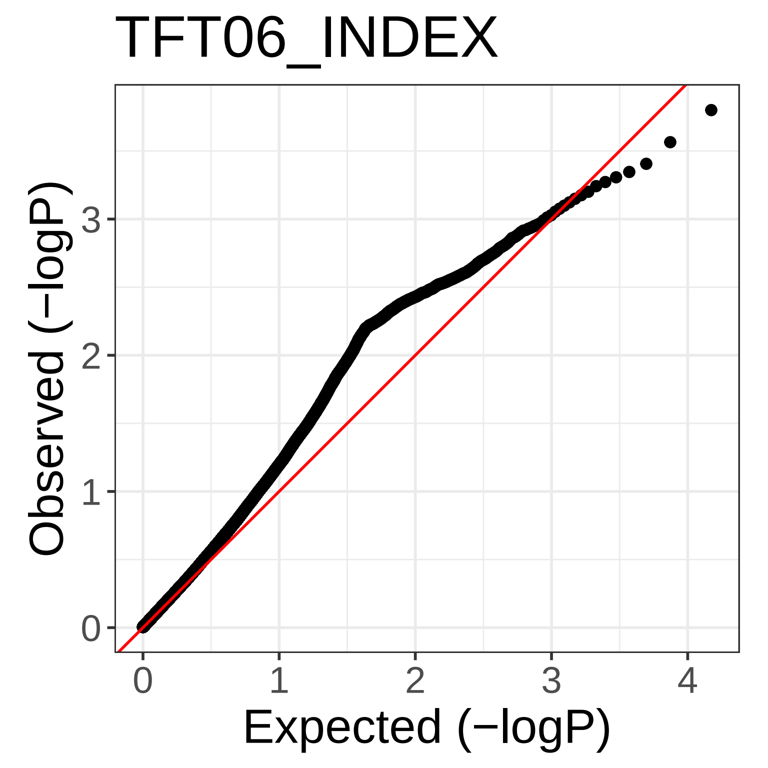
TFT06_INDEX
Observed (−logP)
Expected (−logP)
0
1
2
3
0
1
2
3
4

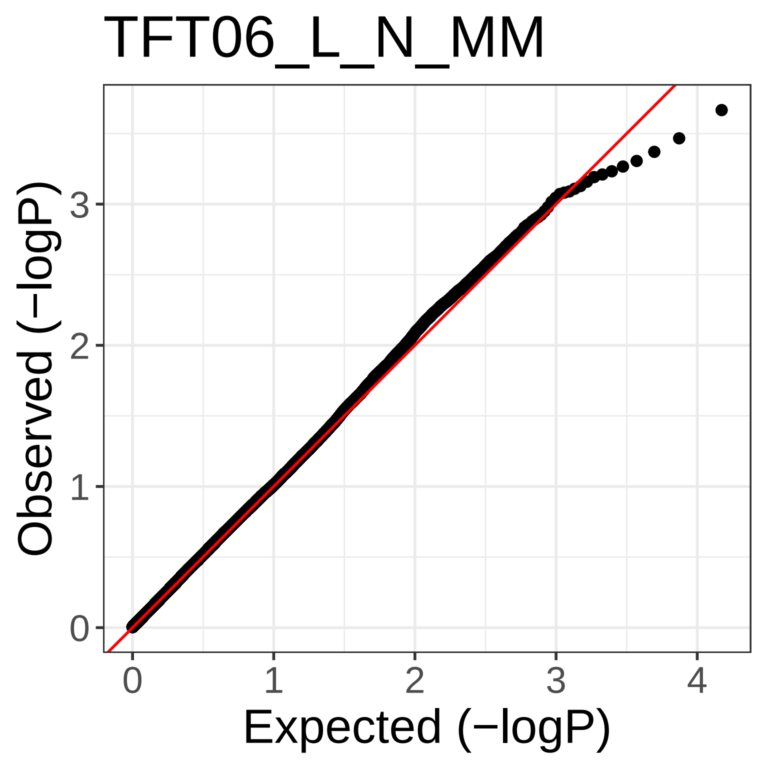

TFT06_L_N_MM
Observed (−logP)
Expected (−logP)
0
1
2
3
4

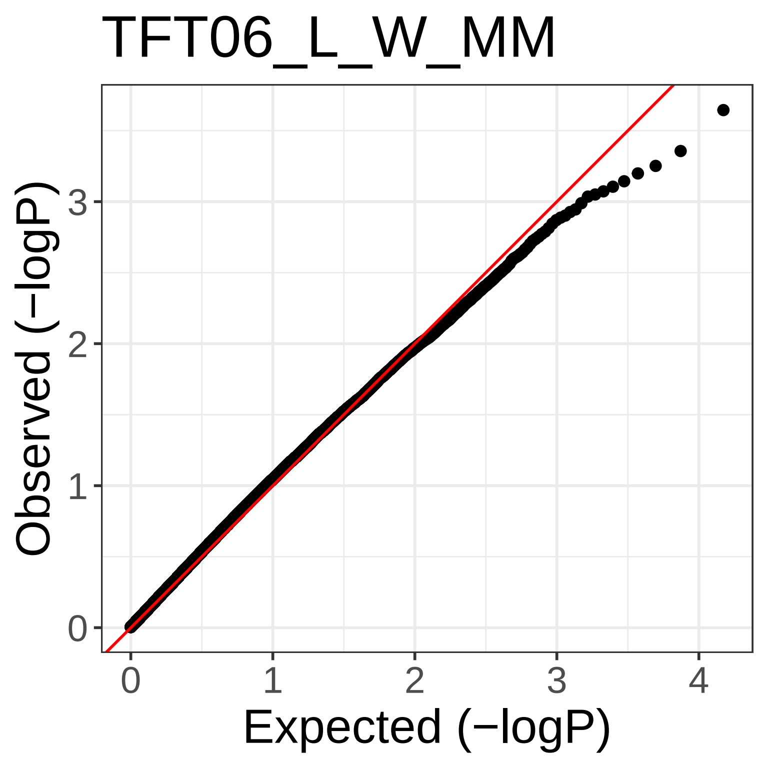
TFT06_L_W_MM
Observed (−logP)
Expected (−logP)
0
1
2
3
4
0
1
2
3

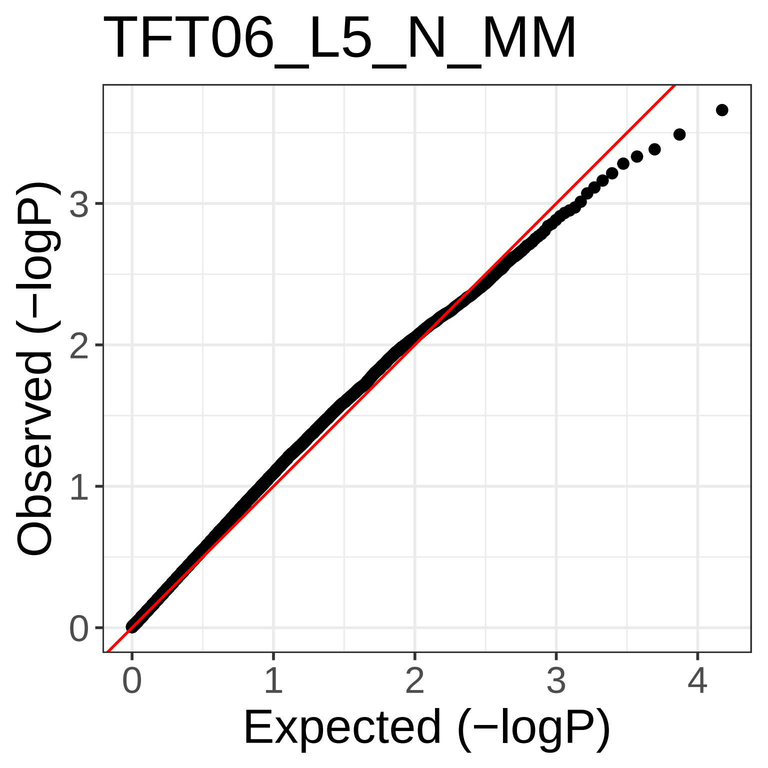
TFT06_L5_N_MM
Observed (−logP)
Expected (−logP)
0
1
2
3
4

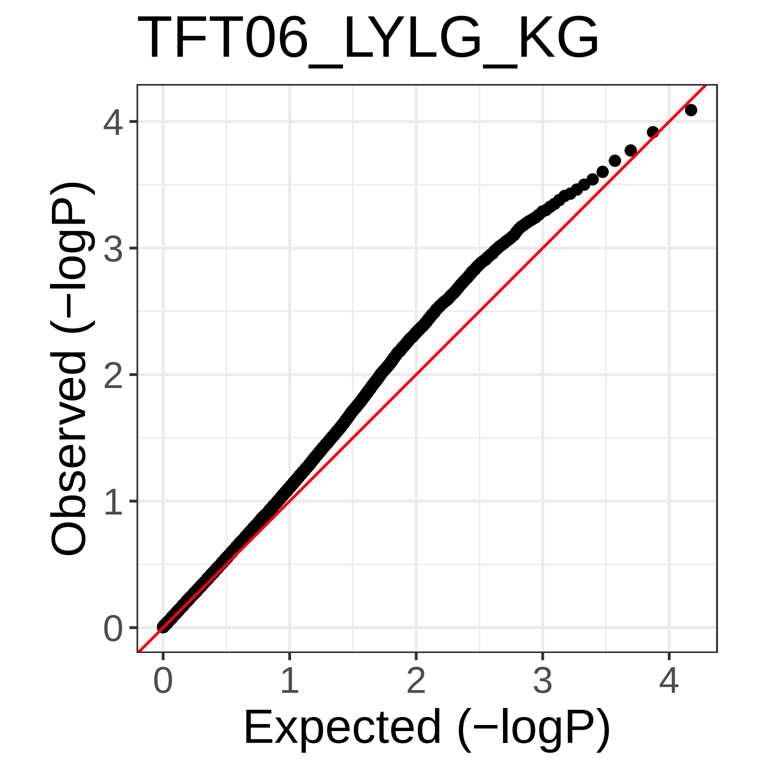
TFT06_LYLG_KG
Observed (−logP)
Expected (−logP)
0
1
2
3
4
0
1
2
3
4

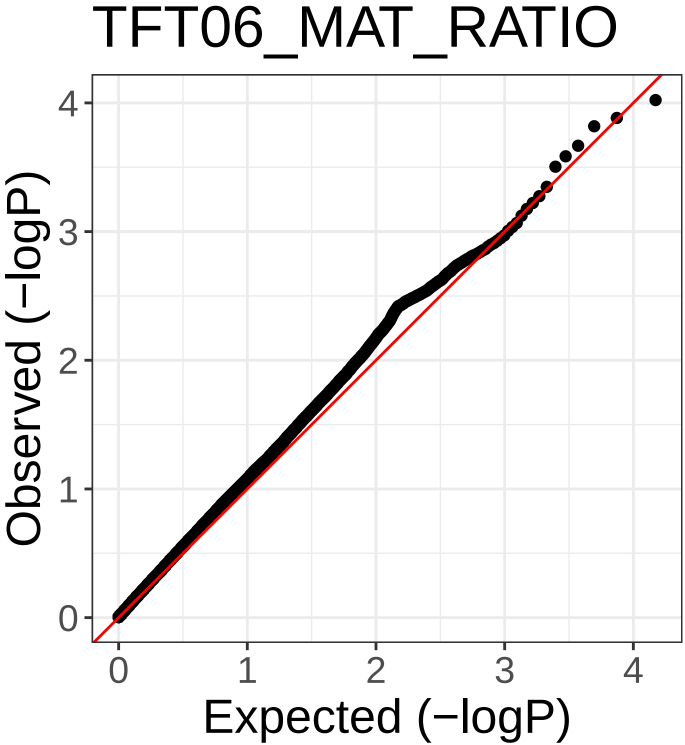

TFT06_MAT_RATIO
Observed (−logP)
Expected (−logP)
0
1
2
3
4
0
1
2
3
4

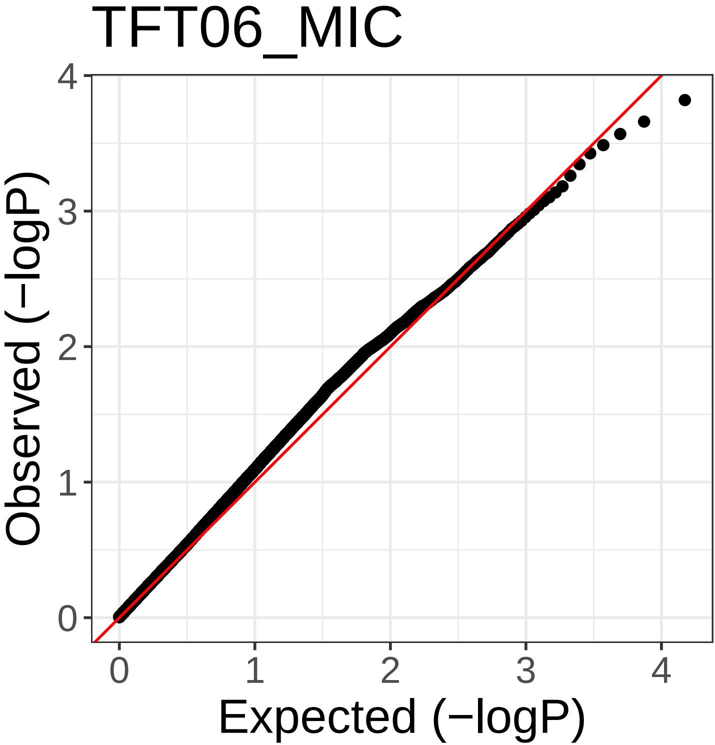
TFT06_MIC
Observed (−logP)
Expected (−logP)
0
1
2
3
4
0
1
2
3
4

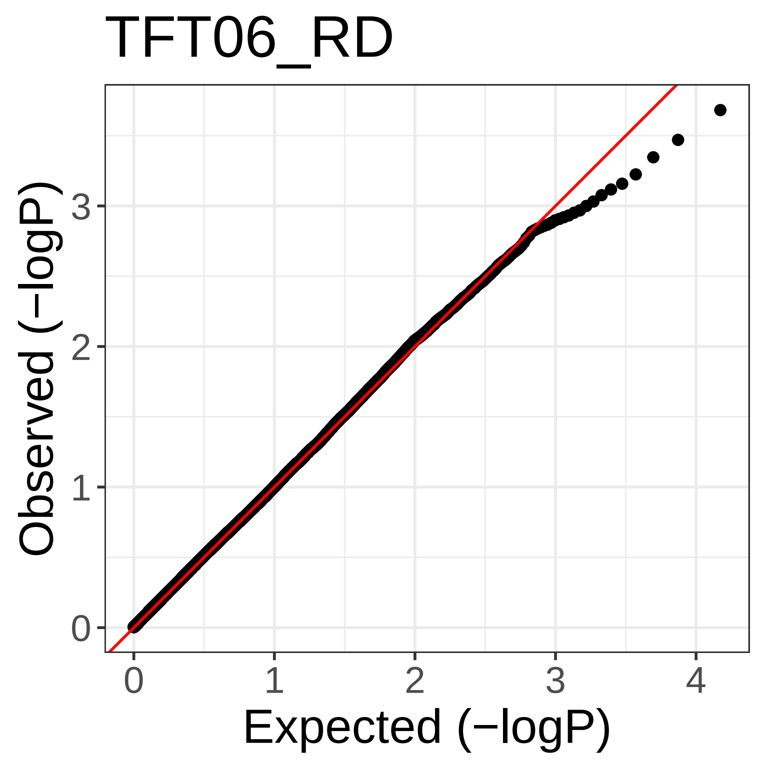
TFT06_RD
Observed (−logP)
Expected (−logP)
0
1
2
3
0
1
2
3
4

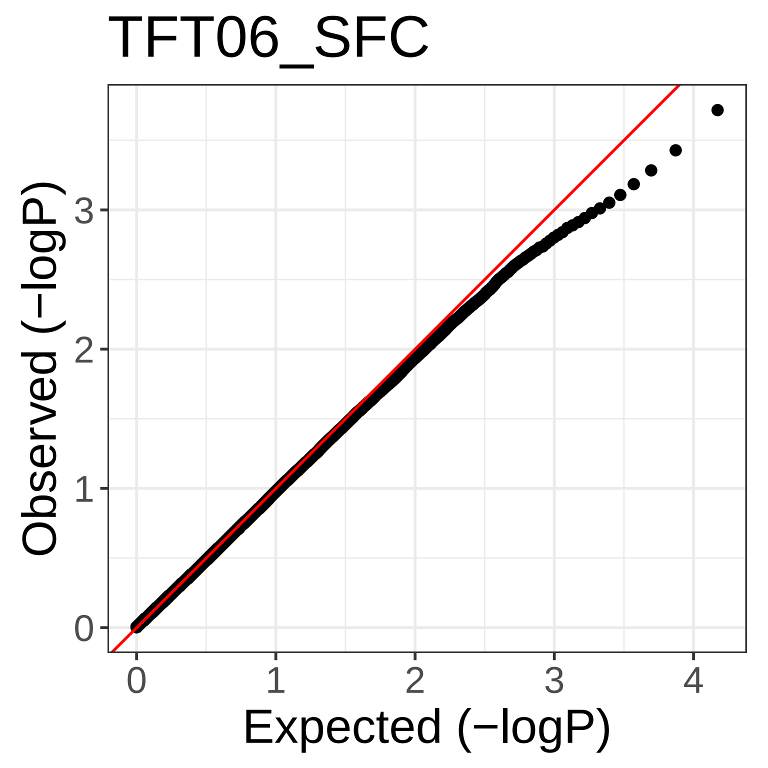
TFT06_SFC
Observed (−logP)
Expected (−logP)
0
1
2
3
0
1
2
3
4

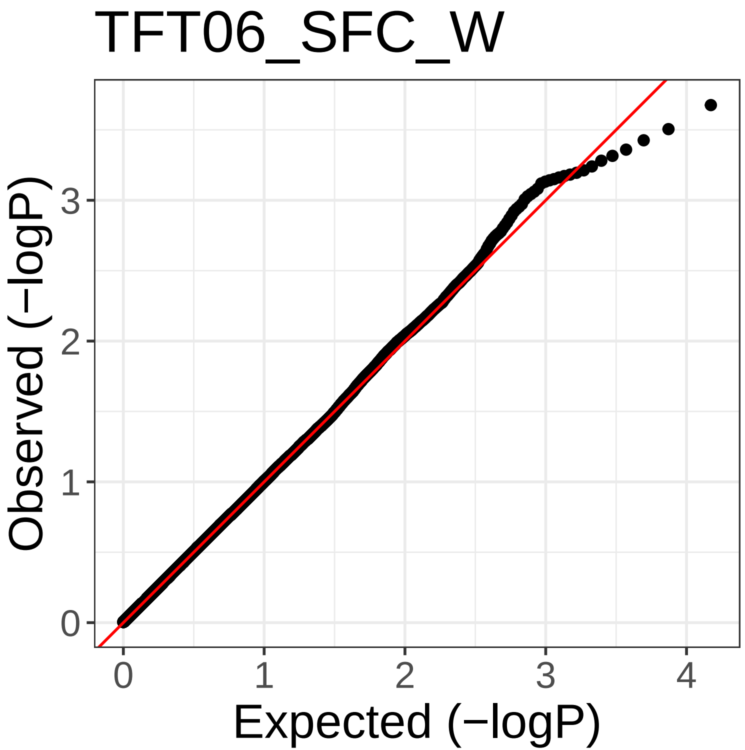
TFT06_SFC_W
Observed (−logP)
Expected (−logP)
0
1
2
3
4

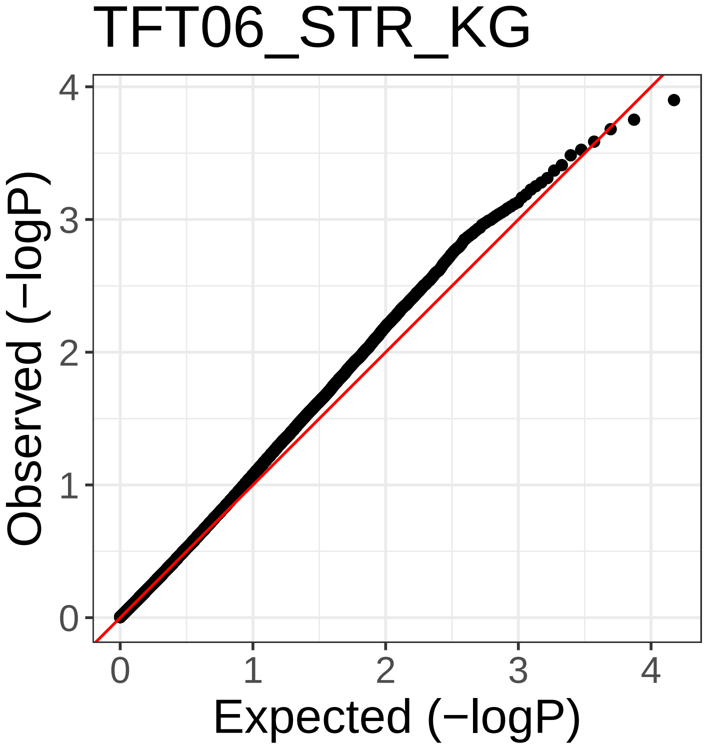
TFT06_STR_KG
Observed (−logP)
Expected (−logP)
0
1
2
3
4
0
1
2
3
4

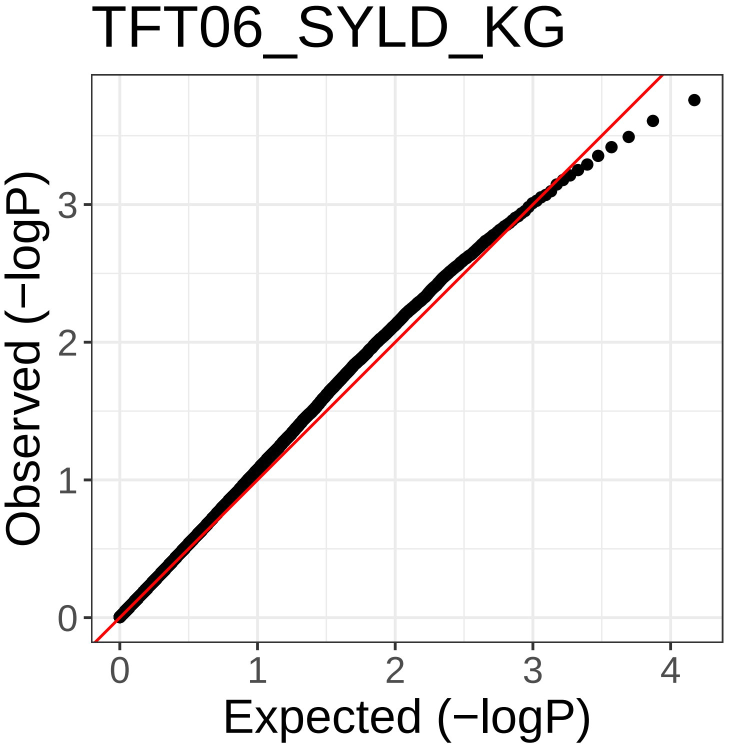
TFT06_SYLD_KG
Observed (−logP)
Expected (−logP)
0
1
2
3
4

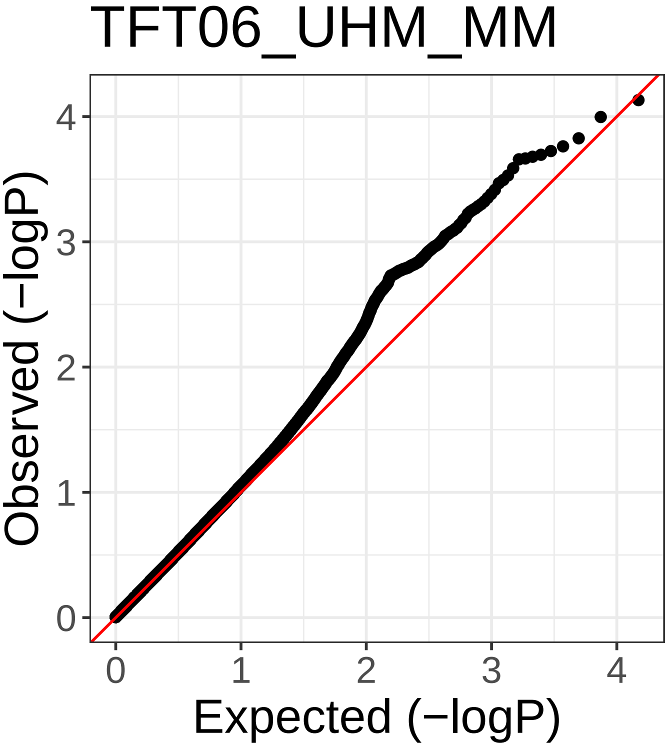
TFT06_UHM_MM
Observed (−logP)
Expected (−logP)
0
1
2
3
4
0
1
2
3
4

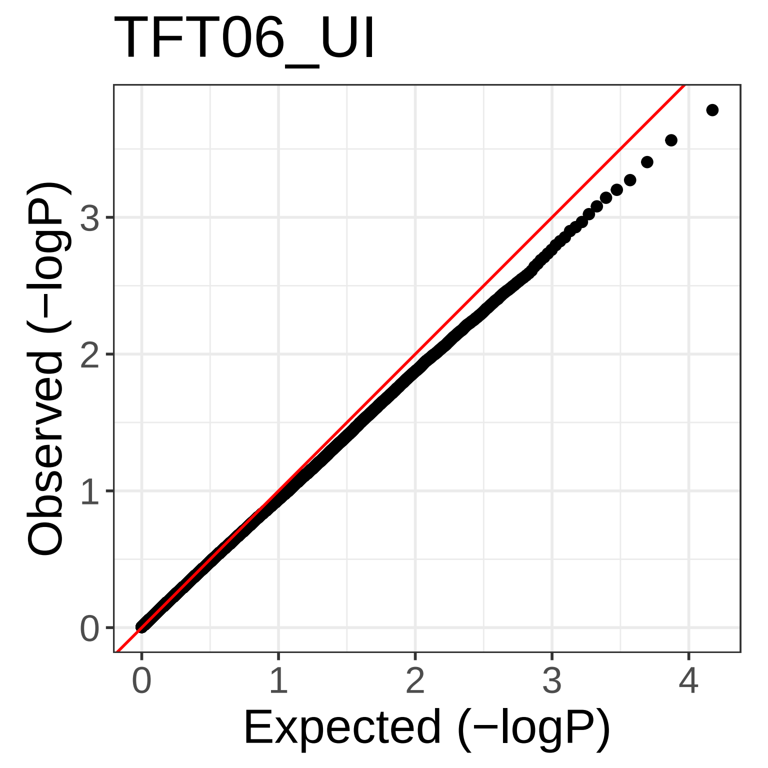
TFT06_UI
Observed (−logP)
Expected (−logP)
0
1
2
3
4

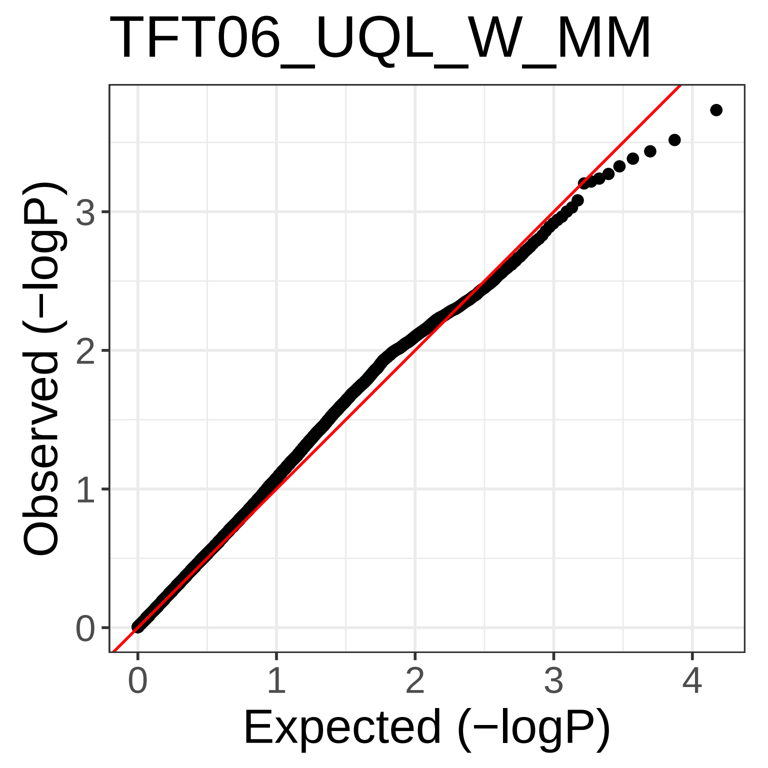
TFT06_UQL_W_MM
Observed (−logP)
Expected (−logP)
0
1
2
3
4
