# Supplementary figures and images for: Outlook for Implementation of Genomics-Based Selection in Public Cotton Breeding Programs
Source: Plants (Basel). 2022 May 29;11(11):1446. doi: 10.3390/plants11111446 (PMC9182660; doi:10.3390/plants11111446)

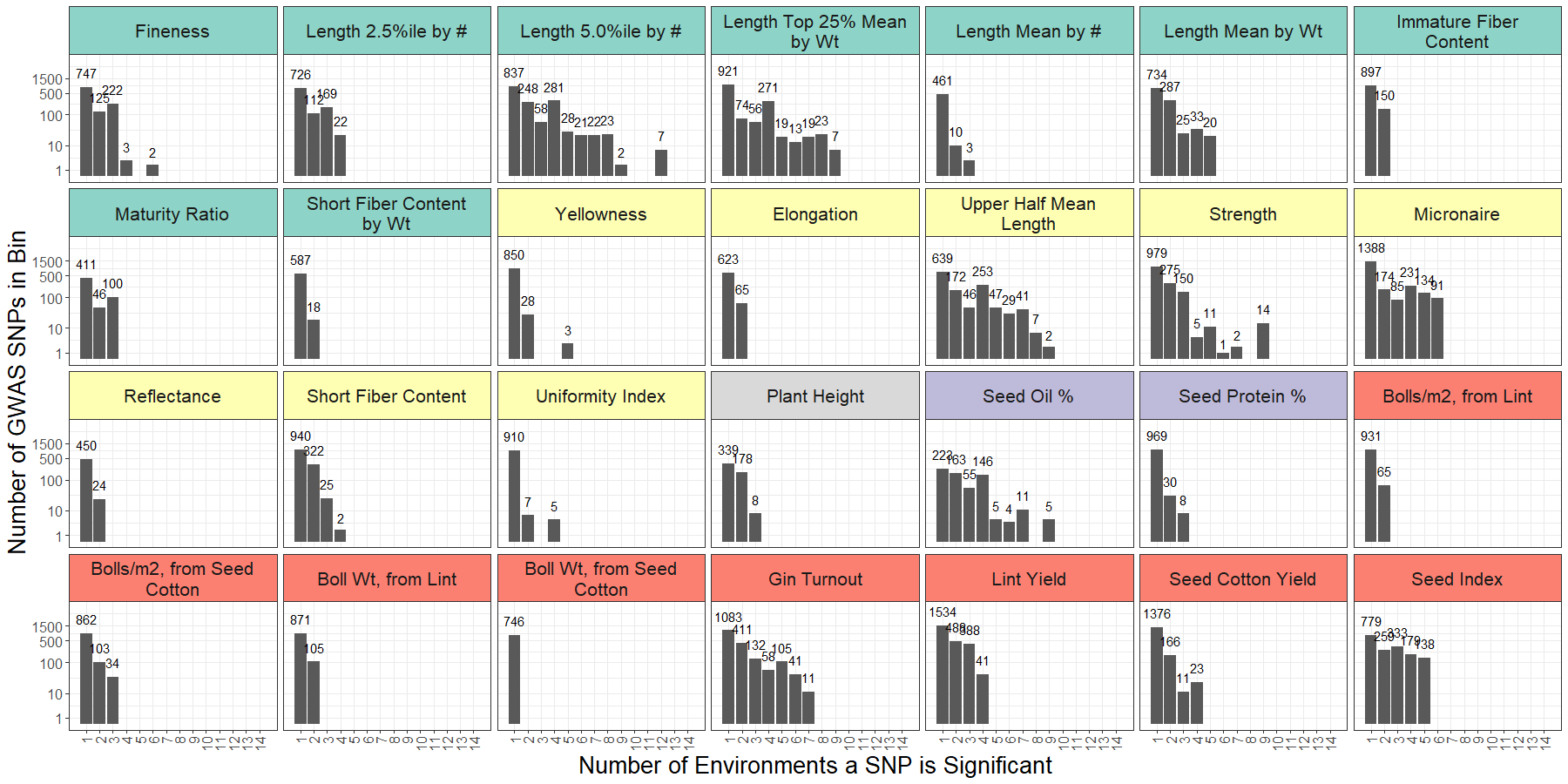

Supplement: Supplementary file 1 [file plants-11-01446-s001.zip › plants-1707252-supplementary/Supplemental_Figure S1.tiff]

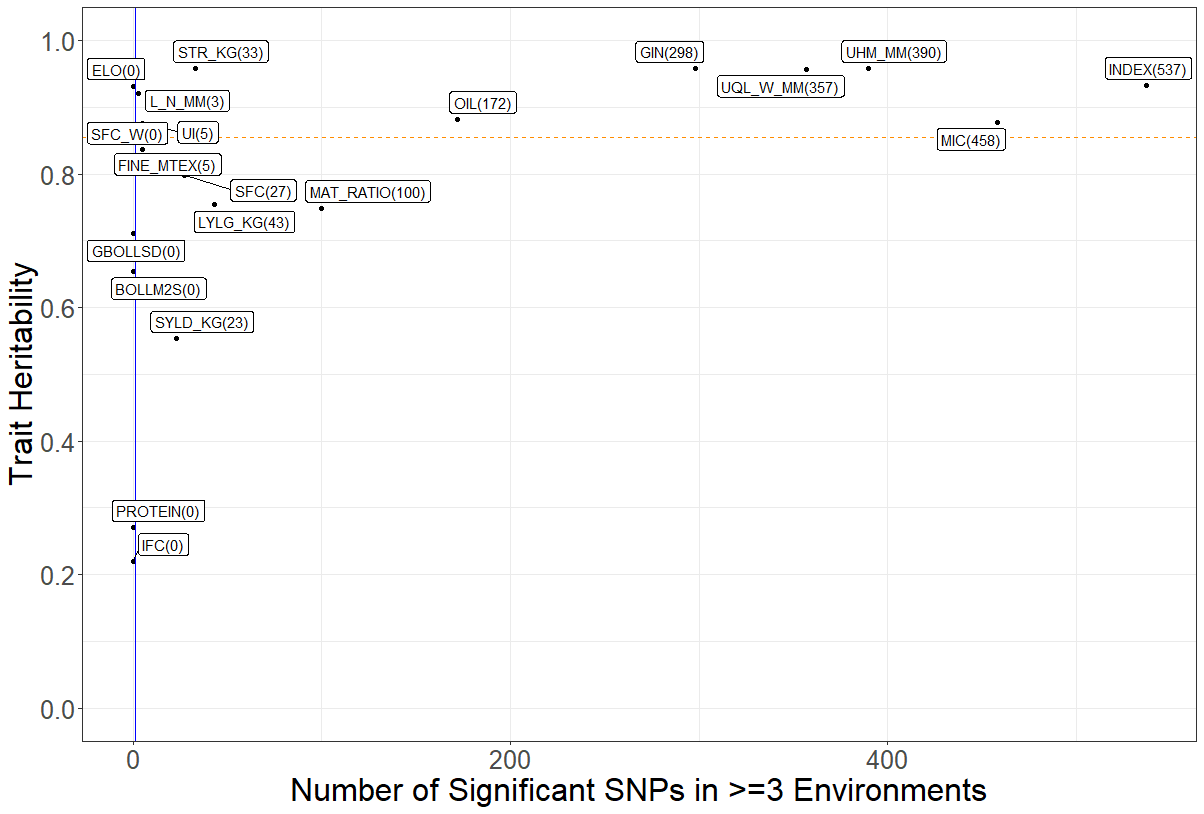

Supplement: Supplementary file 1 [file plants-11-01446-s001.zip › plants-1707252-supplementary/Supplemental_Figure S2.tiff]

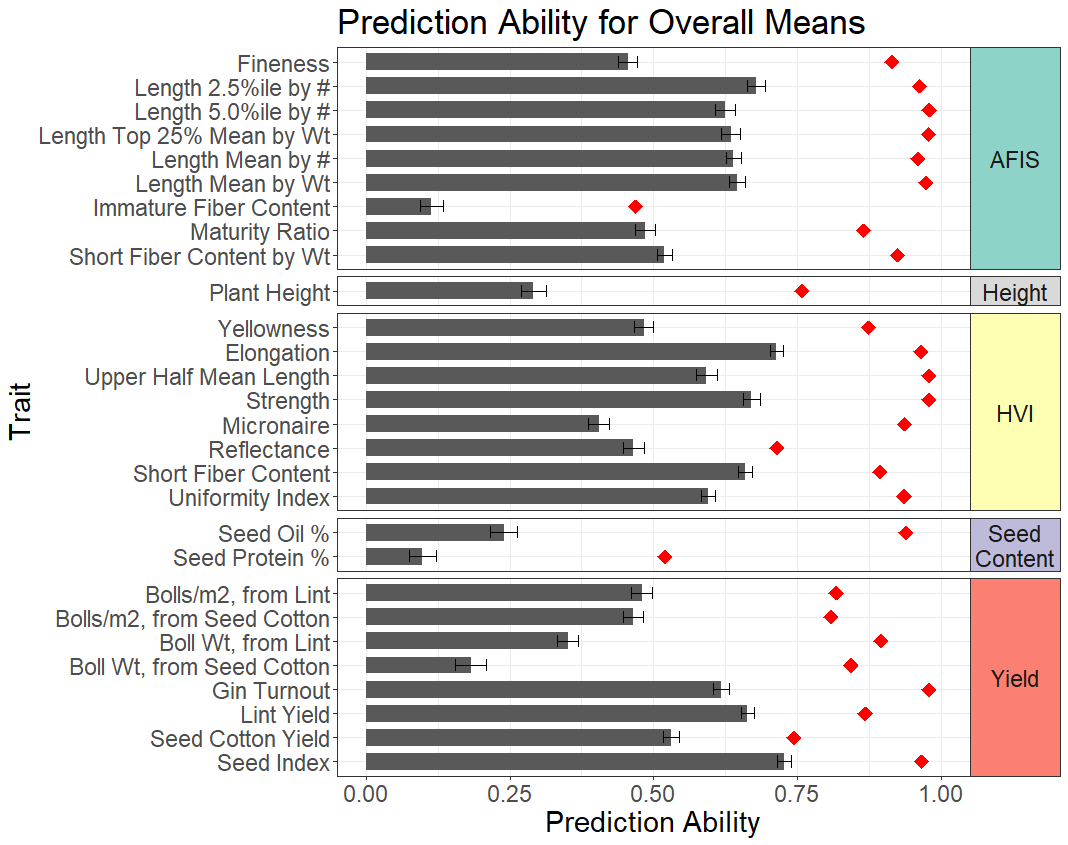

Supplement: Supplementary file 1 [file plants-11-01446-s001.zip › plants-1707252-supplementary/Supplemental_Figure S3.tiff]

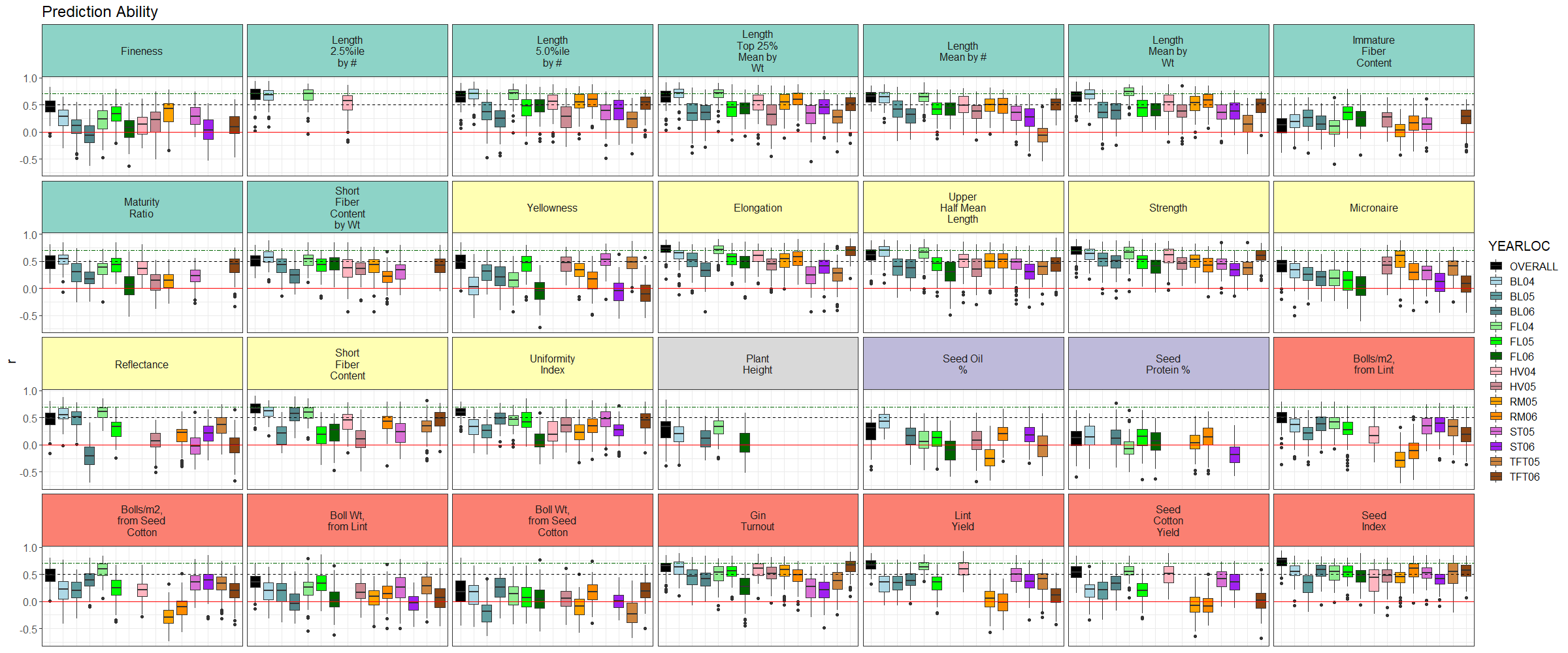

Supplement: Supplementary file 1 [file plants-11-01446-s001.zip › plants-1707252-supplementary/Supplemental_Figure S4.tiff]

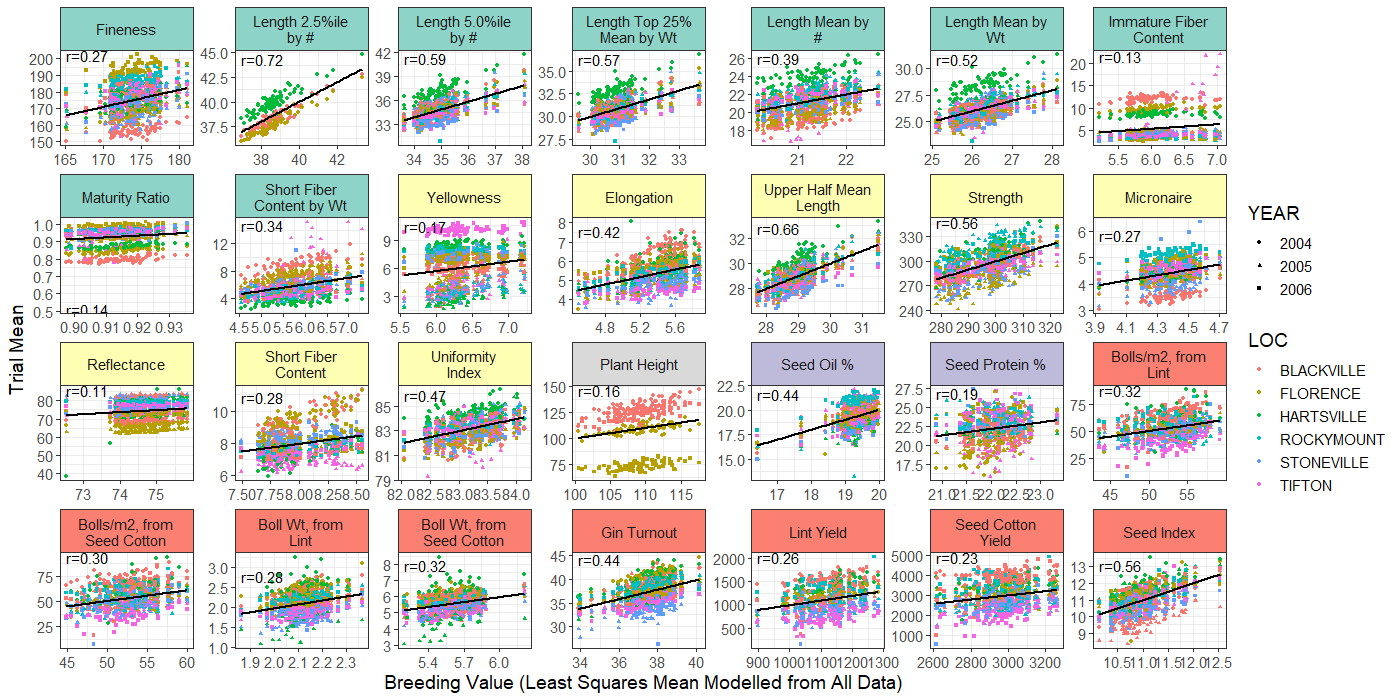

Supplement: Supplementary file 1 [file plants-11-01446-s001.zip › plants-1707252-supplementary/Supplemental_Figure S5.tiff]
